# Covalent Alkynylpyridopyrimidinones Targeting Cysteine 775 of the Epidermal Growth Factor Receptor Overcome Resistance to Current Therapies

**DOI:** 10.1021/acs.jmedchem.5c02924

**Published:** 2025-12-23

**Authors:** Hannah L. Stewart, Cinzia Bordoni, Claire E. Jennings, Islam Al-Khawaldeh, Mathew P. Martin, Richard A. Noble, Nicole Phillips, Sara Pintar, Lisa Prendergast, Huw D. Thomas, Lan-Z. Wang, Jessica E. Watt, Anita Wittner, Agnieszka K. Bronowska, Céline Cano, Martin E. M. Noble, Stephen R. Wedge, Michael J. Waring

**Affiliations:** † Cancer Research Horizons Newcastle Drug Discovery Group, Chemistry, School of Natural and Environmental Sciences, Bedson Building, 5994 Newcastle University, Newcastle upon Tyne NE1 7RU, U.K.; ‡ Cancer Research Horizons Newcastle Drug Discovery G, Translational and Clinical Research Institute, Paul O’Gorman Building, Newcastle University, Newcastle upon Tyne NE2 4HH, U.K.; § Chemistry, School of Natural and Environmental Sciences, Bedson Building, Newcastle University, Newcastle upon Tyne NE1 7RU, U.K.

## Abstract

Inhibitors of epidermal growth factor receptor (EGFR) kinase activity are clinically effective treatments for lung cancers driven by activating mutations in EGFR. Resistance to inhibitors develops over time, frequently through further mutations in the kinase domain. On-target resistance to third-generation inhibitor osimertinib, commonly develops through C797S mutation that prevents covalent binding. There is an urgent need for new treatments for osimertinib-resistant EGFR mutants that retain the advantages of the covalent mechanism. Compounds were designed and synthesized to covalently inhibit EGFR through C775, a further cysteine residue we identified in the orthosteric site. Optimisation of the alkynylpyridopyrimidinone scaffold we discovered led to potent compounds that demonstrate inhibition of EGFR phosphorylation and tumor growth in all EGFR mutant cell lines. The covalent C775 mode-of-action was comprehensively established. This work demonstrates that covalent targeting of C775 is a viable mechanism for the treatment of pan-EGFR mutated cancers, particularly those resistant to current therapies.

## Graphical abstract

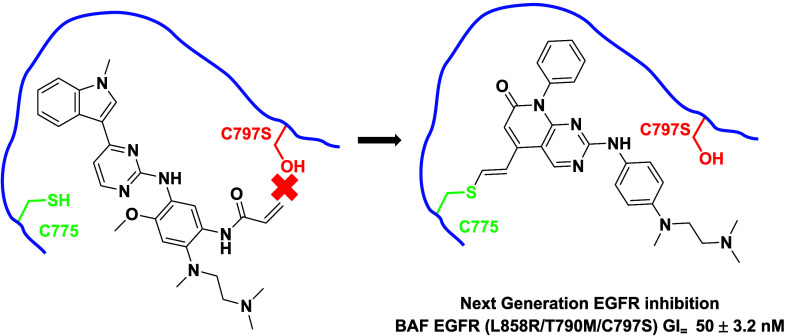

## Introduction

The epidermal growth factor receptor (EGFR) is one of four receptor tyrosine kinases in the erbB family. It mediates downstream cellular signaling in response to binding ligands such as epidermal growth factor (EGF). Ligand binding effects homo- or heterodimerization (with other family members), resulting in autophosphorylation by the kinase domain and subsequent phosphorylation of downstream substrates to propagate signal transduction cascades. EGFR signaling mediates cellular proliferation, survival and suppression of apoptosis. Increased EGFR signaling, particularly through activating mutations in the kinase domain, is a key oncogenic driver, promoting tumor cell proliferation, invasion and metastasis. Nonsmall cell lung cancer (NSCLC) tumors in particular, frequently have oncogenic drivers arising from activating mutations in EGFR, most commonly in-frame deletion of Exon19 (Exon19del) or L858R point mutation.^1^

First-generation EGFR inhibitors, such as erlotinib and gefitinib (Figure 1) are clinically effective in lung cancer patients with the Exon19del or L858R mutations.^2,3^ The success of these treatments arises in part from the reduced ATP affinity of the mutant forms relative to the wild-type, which allows a therapeutic margin to the dose-limiting toxicity associated with WT-EGFR inhibition (skin rash and diarrhea).^4^ Nevertheless, resistance occurs in response to treatment, typically over 10–12 months and most commonly through a mutation of the “gatekeeper” residue (T790M).^5^ The resulting doubly mutated forms of the kinase have reduced affinity for the inhibitors and increased affinity for ATP, such that the inhibitors become less potent and lose their wild-type margin.^6^ Attempts to overcome this resistance focused on the development of covalent inhibitors that target C797. Initial covalent EGFR inhibitors (sometimes termed second-generation inhibitors, such as afatinib and dacomitinib) (Figure 1), were based on the same (anilinoquinazoline) scaffold as first-generation inhibitors and consequentially, despite having increased affinity through the covalent interaction, still suffered from poor wild-type margin.^7^

**Figure 1:**
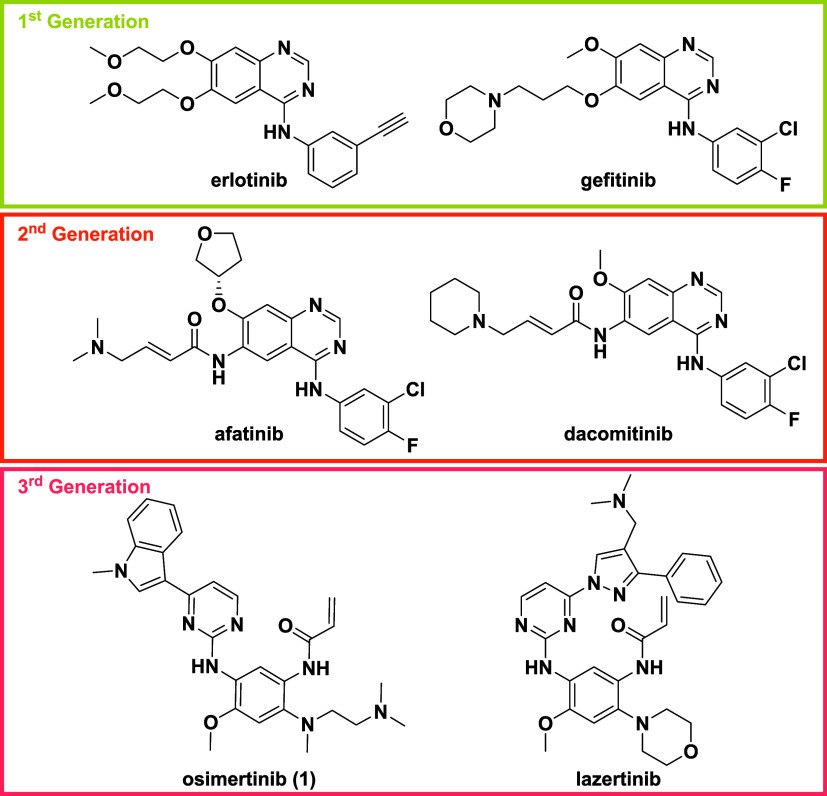
Existing EGFR inhibitors.

Third-generation inhibitors, were developed to inhibit the T790M mutated forms of EGFR, most significantly osimertinib (**1**, Figure 1),^8^ which is active against both the doubly mutated resistant and singly mutated activated forms of EGFR and is now widely used in both first- and second-line NSCLC treatment.^9,10^ Osimertinib is also a covalent inhibitor targeting C797, but is based on an alternative scaffold (anilinopyrimidine) that retains activity against the T790M gatekeeper mutant and wild-type selectivity.^11^ More recently, lazertinib (Figure 1) has also been clinically approved for patients with mutant EGFR driven NSCLC, with comparable activity to osimertinib and improved blood-brain barrier penetration driving its use for those with brain metastases, often given in combination with amivantamab.^12^

Resistance to third-generation therapy develops after both second^13^ and first line^14,15^ treatments (PFS 8.5 and 18.9 months respectively). Often, osimertinib resistance arises from further EGFR mutations that hinder the covalent interaction, most commonly C797S.^16,17^ In the case of second-line osimertinib treatment, this results in a triply mutated protein (e.g., L858R/T790M/C797S), whereas for first-line treatment, resistant isoforms are typically doubly mutated, most predominantly Exon19del/C797S.^18^

All tumors developing resistance to covalent inhibition by osimertinib through further EGFR mutation become intractable to therapy. New inhibitors of these resistant mutants are urgently needed and, because they preclude a covalent interaction with C797, it is highly desirable to develop alternative covalent mechanisms, so that the ability to overcome the high ATP affinity of the EGFR mutants is retained. Furthermore, it is essential that future treatments retain wild-type selectivity.

Examining the structure of EGFR, we identified an alternative cysteine residue in the EGFR ATP-binding pocket (C775) that we reasoned may be amenable to covalent binding. This residue is potentially harder to target than C797, sitting as it does below the gatekeeper residue in a hydrophobic environment,^19^ with only simple C775-bound fragments (Figure 2a) and dual C775–C797 inhibitors (Figure 2b) published at the time of writing.^20,21^

**Figure 2:**
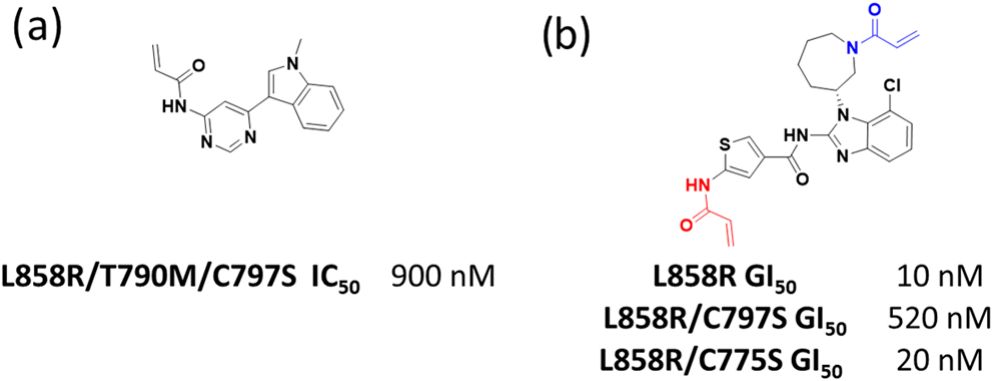
(a) Lead C775-binding fragment with 900 nM binding to TM-EGFR and no WT margin; (b) dual C775–C797 inhibitor, with the C775 warhead highlighted in red and the C797 warhead in blue. GI50 data for growth inhibition of BAF EGFR cell lines demonstrating a much higher binding efficiency for C797.

## Results and Discussion

To identify a suitable chemical scaffold capable of offering a synthetic vector to target C775 we analyzed a series of ligand bound EGFR crystal structures and identified anilinopyrimidine (exemplified by osimertinib, Figure 3a,d),^22^ aminopyrazolopyrimidine (e.g., ibrutinib, Figure 3b,e)^23^ and pyridopyrimidinone (Figure 3c,f)^24^ scaffolds as most promising for derivatization with covalent warheads to trap C775. From the chosen vector, each demonstrated a distance to C775 of 4–7 Å, which we hypothesized could be modified with a covalent warhead, such as an acrylamide, linked to the scaffold directly or via a linker for optimal covalent binding geometry.

**Figure 3:**
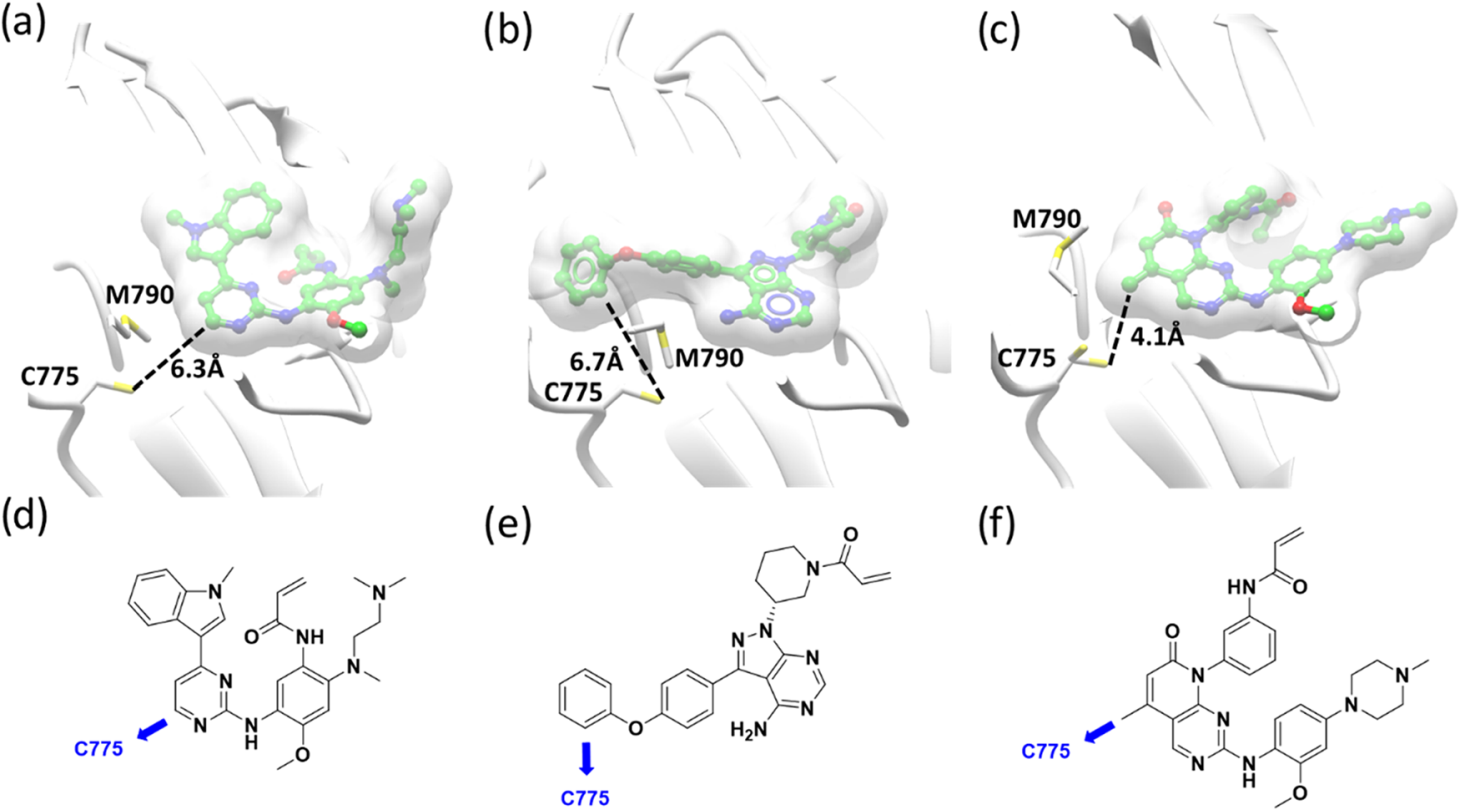
Ligand-bound EGFR structures that could be derivatized with covalent warheads through the vectors shown across distances of 4–7 Å (dashed line) to trap C775 which sits under the methionine gatekeeper. (a) Osimertinib (PDB: 6Z4B); (b) ibrutinib (PDB: 5YU9); (c) pyridopyrimidinone scaffold (PDB: 5GMP); illustration of the structures with the vector to C775 highlighted in blue for (d) osimertinib; (e) ibrutinib; (f) pyridopyrimidinone-based compound.

The published structures were simplified, with the existing C797 covalent warheads and more elaborated pendant groups removed, leading to simple core scaffolds A, B, and C (Table S1). Synthesized compounds (Schemes S1 and S2), with acrylamide and propiolamide warheads were screened through a TR-FRET assay against recombinant, triply mutated EGFR (L858R, T790M, C797S) kinase domain (mLTC-EGFR) using a fluorescent probe displacement assay^25^ and assessed for covalent adduct formation by intact protein mass spectrometry. Disappointingly, although these largely demonstrated covalent adduct formation with a stoichiometry of 1–3, few gave an IC_50_ below 100 μM, with those that did possessing the significantly more reactive propiolamide warhead.^26^

Given the hydrophobic nature of the methionine gatekeeper in mutant EGFR, we considered that the traditional acrylamide warheads may not be tolerated due to their hydrophilic nature. The electron-deficient nature of the pyrimidine ring in scaffold A and pyridopyridmine ring in scaffold C opened the possibility of appending alkynyl or alkenyl groups directly to the scaffolds since their conjugation with the electron-deficient heterocyclic ring systems could render them sufficiently electrophilic.

Pleasingly, compound **2** (Figure 4a and Scheme S3) not only demonstrated low micromolar affinity for mLTC-EGFR, but upon incubation with mLTC-EGFR revealed addition of a single adduct by mass spectrometry and a small amount of double adduct (Figure 4b). Trypsin digest of the modified protein identified C775 as the site of modification, thus verifying that C775 is amenable to protein modification (Figure 4c). However, this compound showed only 2-fold selectivity over the wild-type protein.

**Figure 4:**
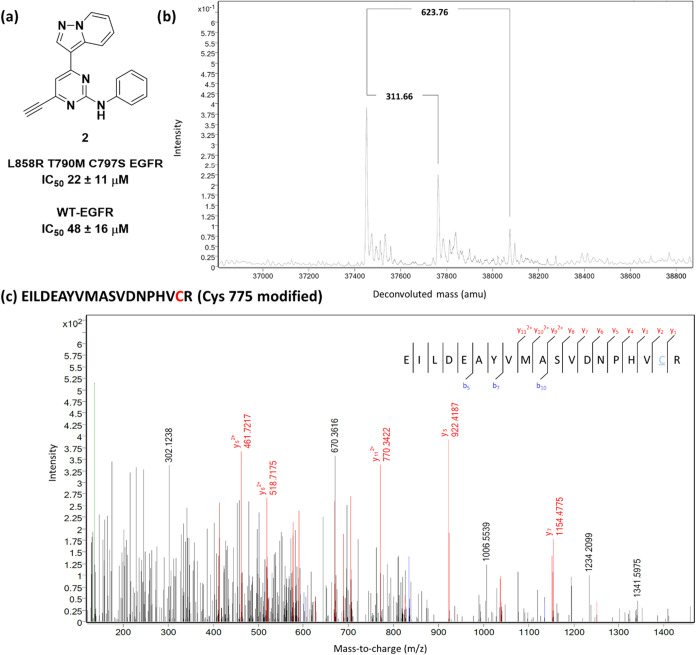
(a) Structure of **2** and IC_50_ values determined by a TR-FRET assay (*n* > 3) against recombinant triply mutated EGFR L858R, T790M, C797S kinase domain (mLTC-EGFR) and wild-type EGFR (WT-EGFR); (b) protein mass spectrometry data showing modulation of triple mutant EGFR by predominantly a single molecule of **2**; (c) protein digest mass spectrometry data showing modification of cysteine 775.

Alkyne-substituted scaffold C derivative **3** (Scheme 3) showed significantly increased potency (nanomolar range) and vastly improved wild-type selectivity (>10-fold) compared to **2** (Figure 5a). Covalent adduct formation was confirmed by protein mass spectrometry (Figure 5b) with a trypsin digest again confirming modification of C775 (Figure 5c).

**Figure 5:**
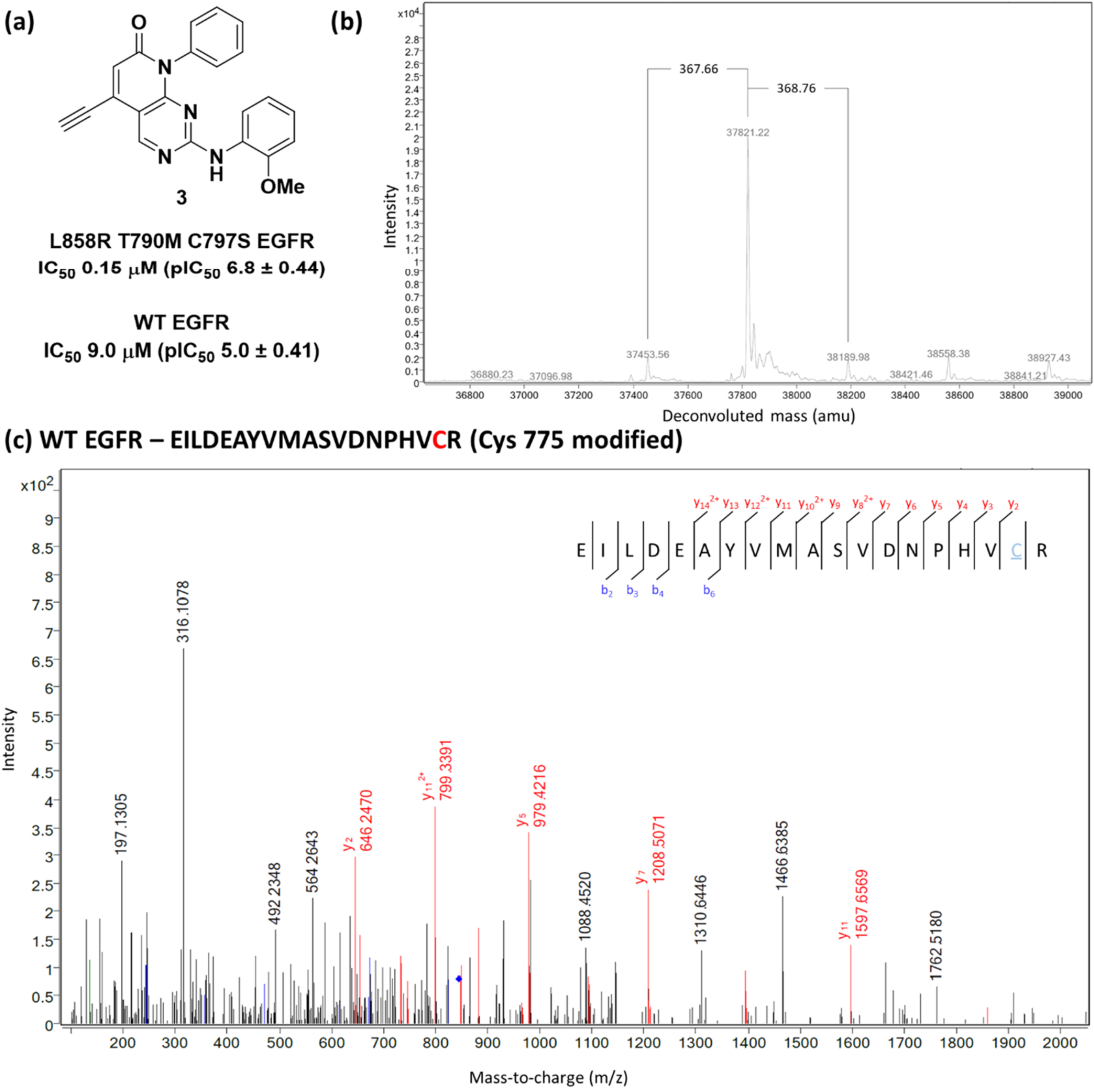
(a) Structure **3**, first lead compound and IC_50_ values determined by a TR-FRET assay (*n* > 3) against recombinant triply mutated EGFR L858R, T790M, C797S kinase domain (mLTC-EGFR) and wild-type EGFR (WT-EGFR); (b) protein mass spectrometry data showing modulation of WT-EGFR by predominantly a single molecule of **3**; (c) protein digest mass spectrometry data showing modification of cysteine 775.

Compound **3** also showed inhibition of pEGFR in H1975 at 10 μM (Western blot, Figure 6a), with an IC_50_ > 3.0 μM by HTRF (Figure 6b). The X-ray crystal structure of **3** bound to WT-EGFR showed the pyridopyrimidinone core retained the two hydrogen-bonds with the peptide backbone of M793 while being held in place through a hydrophobic sandwich between the L844 and A743 of the C and N-terminal lobes, respectively. The crystal structure confirmed the formation of a covalent bond between C775 and the alkene adduct with clear continuous density between the residue and the bound inhibitor and the gatekeeper residue T790 stacking on top of the newly formed covalent bond (Figure 6c).

**Figure 6:**
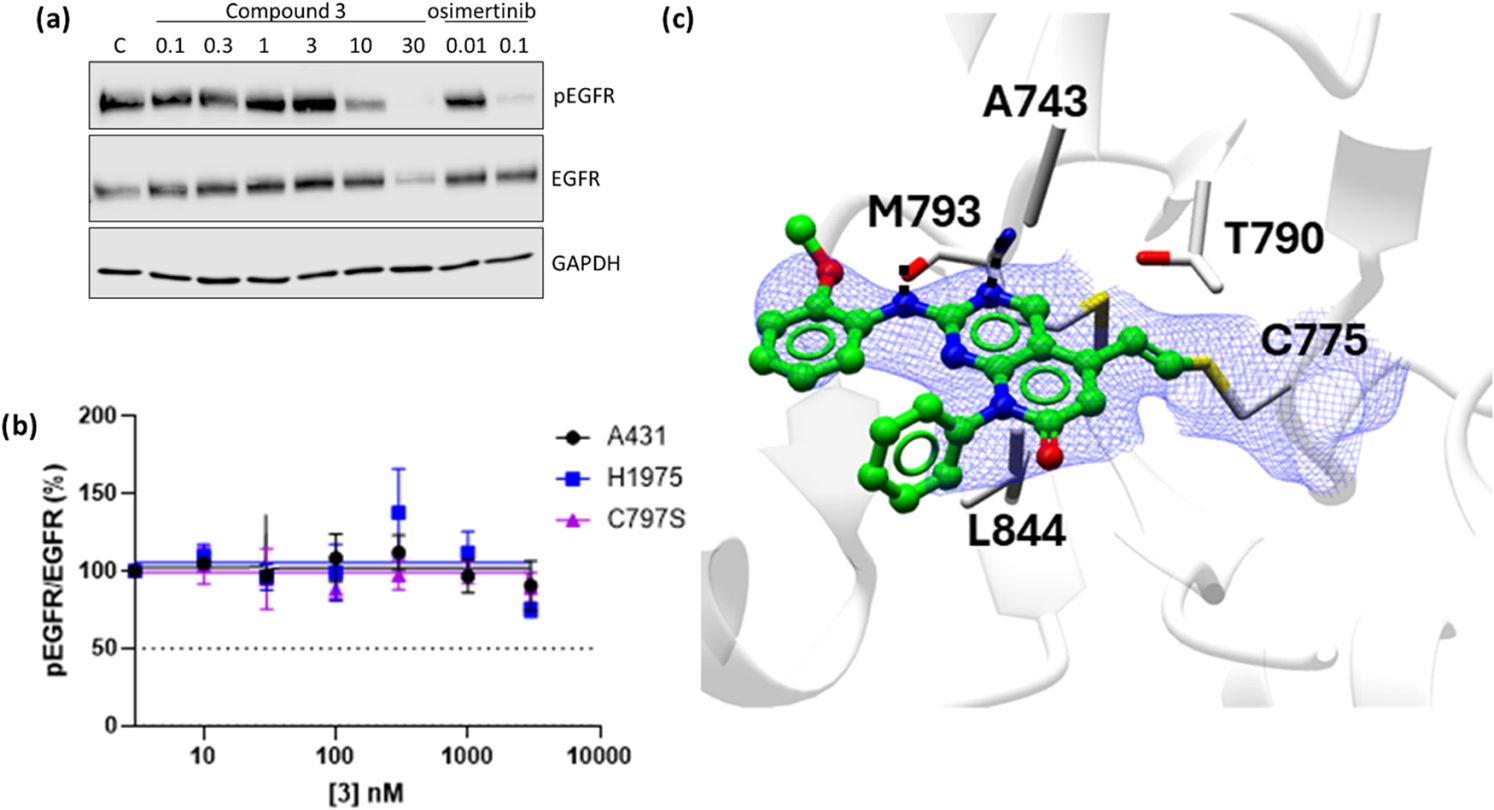
(a) Western blot showing cellular inhibition of EGFR phosphorylation (Tyr1068) in H1975 cells after 4 h; (b) inhibition of cellular EGFR phosphorylation as % of control in H1975 (L858R, T790M), H1975 C797S and A431 (WT-EGFR) cell lines (*n* = 4 ± SEM); (c) crystal structure of **3** (green ball and stick) in complex with wild-type EGFR (white ribbon) showing continuous electron density between C775 (white cylinder) and the alkene linked pyridopyrimidinone core of **3** (blue mesh), contoured a 1.0 I/sigma (0.12 e/Å^3^). Hydrogen bond interactions of the peptide backbone of M793 and **3** (black dash) with hydrophobic sandwich between the L844 and A743 (white ribbon) (PDB: 9H46).

Assessing the time-course of EGFR modification by mass spectrometry showed the expected exponential decay and, even after 24 h, a single covalent adduct was predominant. Over 50% of the protein was modified within 10 min, suggesting reactivity was contributing significantly to potency (Figure S1). The reactivity of the alkynylpyridone in glutathione (GSH) reactivity assays was relatively high (*t*_1/2_ 14 min), comparable to the Phase II EGFR inhibitor canertinib (20 min) but less than osimertinib (60 min), hence the it should be possible to modulate the reactivity into the range populated by clinical covalent compounds with minor structural modifications. An analogue with the covalent group deleted (Compound **S10**, Scheme S4) showed a significant drop in EGFR potency (Table S2, 7.8 μM), establishing that the potency at this stage was dominated by the covalent reactivity.

Replacing the alkyne with an alkene (**S11**, Scheme S5) or methylated alkyne (**S12**, Scheme S5) resulted, as expected, in lower reactivity (GSH *t*_1/2_ 45 min and >380 min respectively) leading to reduced protein modulation by **S11**, little to no modification by **S12** (Figure S2) and a significant drop in EGFR potency (11 μM and >100 μM respectively, Table S3). We therefore looked to increase the noncovalent affinity.

Basic or polar substituents in the *p*-position of the aniline group (Scheme 1) extend into the solvent channel, thus allowing a means of modulating physical properties in a manner that may be tolerated and were explored in the presence and absence of the methoxy substituent (Table 1, compounds **3**, **4**, **5**, **6**, **7**, and **8**). It was necessary to synthesize the desired anilines (**3′**), for the basic amines this entailed an SNAr from the relevant 1-fluoro-4-nitrobenzene (**1′**, R_1_ = H or OMe) before hydrogenation of the nitro group (Scheme 1).

**Scheme 1:**
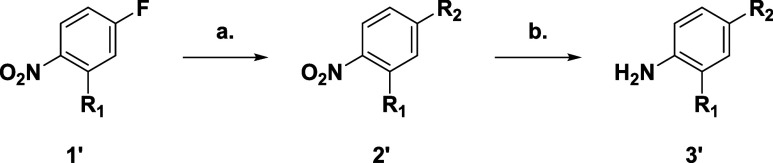
(a) Amine, K_2_CO_3_, DMSO, rt, 12 h, 75–97%; (b) H_2_, Pd/C, EtOH, rt, 12 h, 67–100%

**Table 1:**
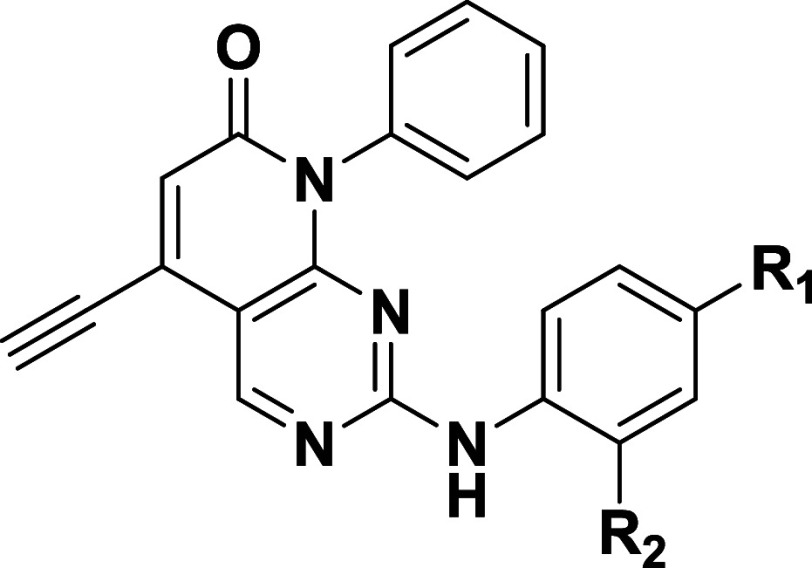

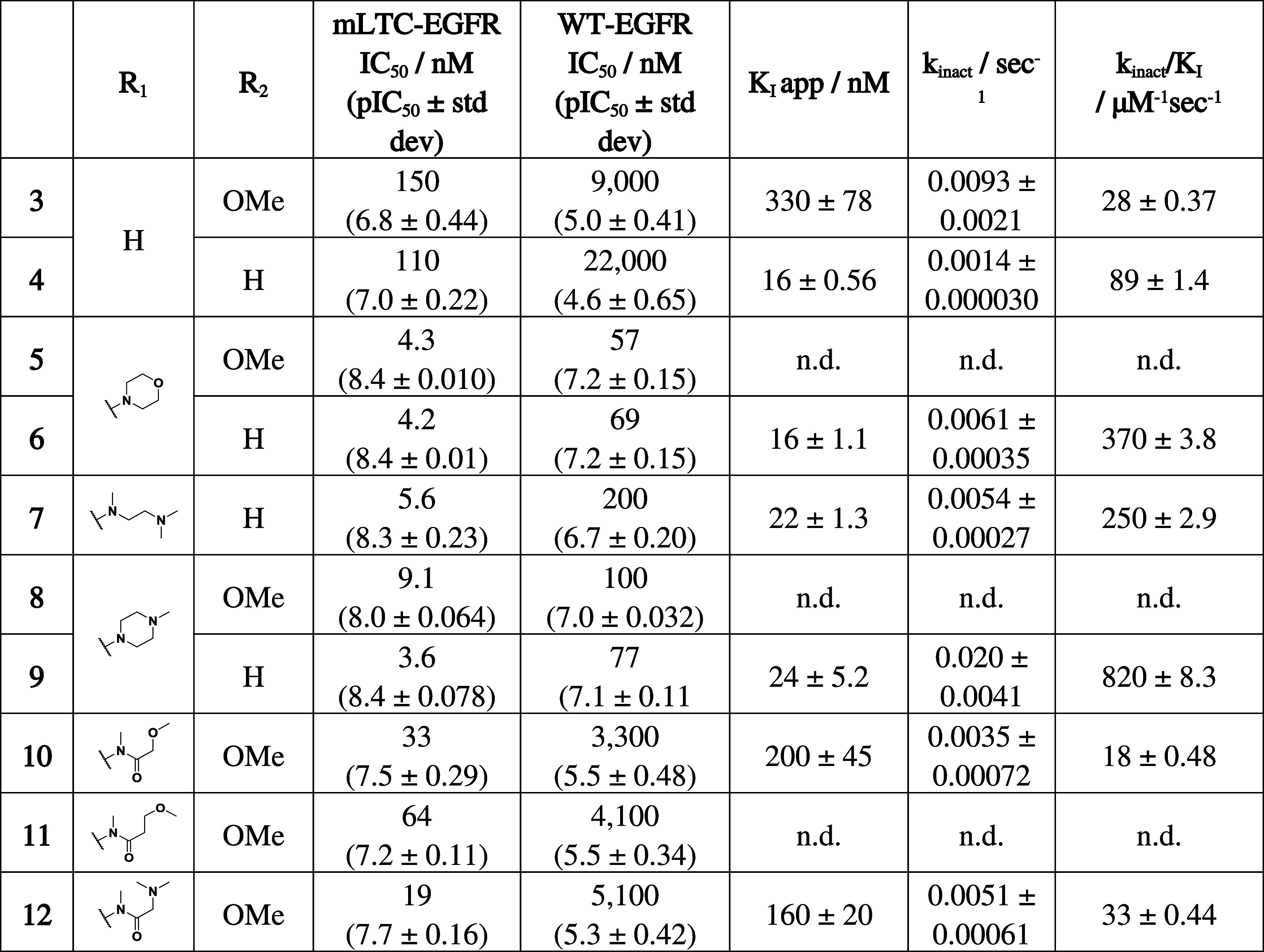
Assay and Kinetic Data for SAR Around the Solvent Channel^a^

a IC_50_ values determined by a TR-FRET assay against recombinant triply mutated EGFR L858R, T790M, C797S kinase domain (mLTC-EGFR) and wild-type EGFR (WT-EGFR). IC_50_ values *n* > 3. Kinetic data determined by AssayQuant against recombinant doubly mutated EGFR L858R, T790M kinase domain (mLT-EGFR).

For the amide solvent channel substituents (Scheme 2), an SNAr reaction between 5-fluoro-2-nitroanisole (**1′**) with methylamine·hydrochloride furnished the amine (**4′**). Acylation gave rise to the desired amides (**5′**, X = OMe or Cl), with the dimethylamino analogue (**6′**) requiring an additional step from the α-chloroamide. Reduction of the nitro group yielded the final 3 anilines (**7′** and **8′**).

**Scheme 2:**
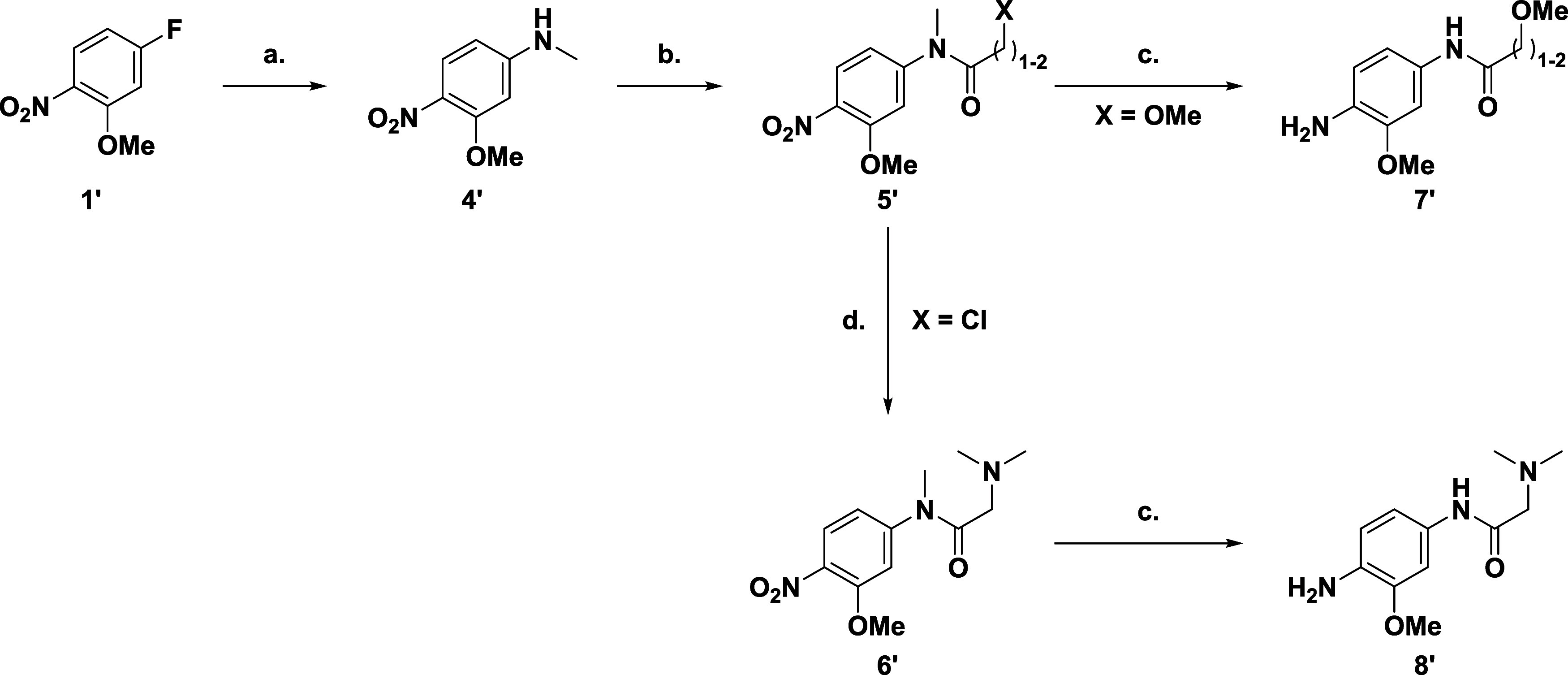
(a) NH_2_Me·HCl, K_2_CO_3_, DMSO, rt, 12 h, 79%; (b) 2-Methoxyacetyl Chloride, DIPEA, DCM, 0 °C-rt, 2 h 100%; 3-Methyoxypropionyl Chloride, Pyridine, MeCN, rt, 12 h, 90%; Chloroacetyl Chloride, EtOAc, 70 °C, 1 h, 64%; (c) H_2_, Pd/C, EtOH, AcOH, rt, 12 h, 52–100%; (d) NHMe_2_, K_2_CO_3_, THF, rt, 12 h, 58%

The core scaffold was synthesized (Scheme 3) from ethyl 4-chloro-2-(methylthio)­pyrimdine-5-carboxylate (**9′**) with an SNAr reaction introducing the sugar pocket group, aniline (Scheme 1). Hydrolysis of the ester (**10′**) gave the acid (**11′**) which was then reacted with benzotriazole to form the activated amide (**12′**). Acylation of the active amide (**12′**) furnished the dicarbonyl (**13′**) from which base catalyzed cyclization yielded the core pyridopyrimidinone scaffold (**14′**) with a 5-hydroxy group acting as a handle for introduction of the warhead. Triflation followed bySonogashira reaction introduced the protected alkyne (**16′**). Finally, oxidation of the methylthiol group gave the activated pyridmine (**17′**) it for subsequent SNAr reaction with the various anilines (Schemes 1 and 2), before deprotection of the TIPS-group unveiled the alkynyl warhead (**19′**).

**Scheme 3:**
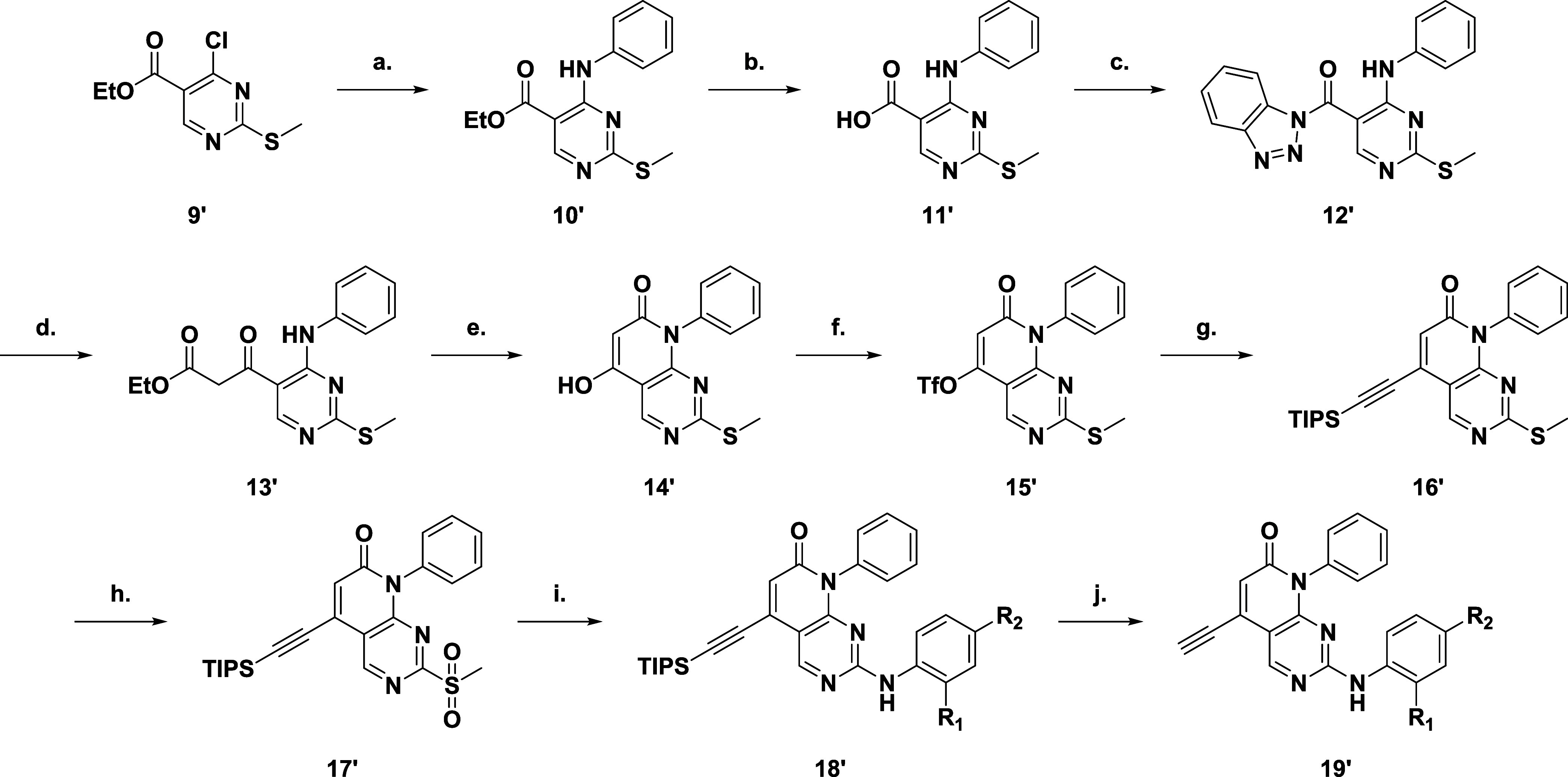
(a) NH_2_Ph, TEA, THF, rt, 12 h, 93%; (b) NaOH, THF, H_2_O, 50 °C, 12 h, 82%; (c) 1*H*-Benzo­[*d*]­[1,2,3]­triazole, EDCI, DCM, rt, 4 h, 85%; (d) EtOAc, LiHMDS, −78 °C-rt, 12 h, 61%; (e) DBU, DIPEA, 120 °C, 1 h, 97%; (f) Tf_2_O, TEA, DCM, 0 °C-rt, 2 h, 55%; (g) TIPSCCH, Pd­(PPh_3_)_2_Cl_2_, CuI, DIPEA, DMF, 80 °C, 3 h, 80%; (h) mCPBA, DCM, rt, 1 h, 89%; (i) Aniline, TFA, MeCN, 85 °C, 12 h 27–74%; (j) KF, DMF, rt, 1–12 h 10–90%

Across the set of 9 analogues exploring the solvent channel, it was found the methoxy substituent had little effect on potency. Morpholine (**5** and **6**), *N* ^1^,*N* ^1^,*N* ^2^-trimethylethane-1,2-diamine (**7**) and piperazine (**8** and **9**) substituents all gave a significant increase in potency to below 10 nM. While amide substituents (**10**, **11** and **12**) showed some improvement in potency over **3**, the more basic side chains were significantly more potent and therefore showed the most promise for further testing.

Given that compound **7** showed the largest wild-type margin it was selected for further evaluation. While the GSH half-life was still short compared to osimertinib and other approved drugs, binding kinetics against doubly mutated L858R, T790M EGFR (mLT-EGFR) were consistent with irreversible binding (*k*_inact_ 0.0054 s^–1^) and the *K*_I_ value with a high level of noncovalent affinity. To further assess the improvement in the noncovalent binding component, compound **S13** lacking the alkynyl warhead was synthesized (Table S2). This demonstrated a greater than 10-fold improvement in potency over **S10** suggesting the inherent noncovalent potency had been significantly increased given that the warhead reactivity was unchanged. Compound **7** demonstrated measurable solubility in phosphate buffer at pH 7.4 (6.5 μM), while microsomal stability (*t*_1/2_ 11 min) would need to be improved (Figure 7a). Despite maintaining a relatively short GSH half-life, **7** formed only a single adduct by intact protein mass spectrometry (Figure 7b). Furthermore, **7** showed excellent selectivity against a panel of 394 kinases, with only 10 showing >75% inhibition at 1.0 μM (Figure S4 and Tables S4 and S5). However, none of these have a homologous cysteine, and so would likely be reversible inhibitors of these kinases.

**Figure 7:**
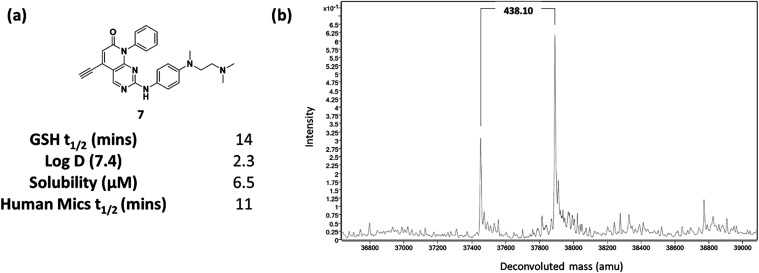
(a) Structure and ADMET data (measured by Cyprotex) for **7**: Log *D*_7.4_ measured using the octanol shake flask method; solubility measured using the turbidimetric method in phosphate buffer at pH 7.4; (b) protein mass spectrum of TM-EGFR showing a single adduct with **7**.

An X-ray crystal structure of **7** bound to mLTC-EGFR (PDB: 9S3X, Figure 8a) was obtained confirming that the hydrogen bonding and hydrophobic interactions of the pyridopyrimidinone core were retained from those observed in the **3**-WT-EGFR structure (PDB: 9H46, Figure 6c). In addition, the **7**-mLTC-EGFR crystal structure confirmed the formation of a **7-**C775 covalent bond and the hydrophobic interactions between the alkene-adduct and the mutant gatekeeper M790. Comparison of the gatekeeper residue in **7** bound to both mLTC-EGFR and WT-EGFR provided a rationale for the enhanced binding affinity of the pyridopyrimidinone series to the mutant form. Cβ and Cγ of the M790 Mutant form gatekeeper were observed to stack on top of the β-carbon of the newly formed alkene covalent bond originating from the alkyne adjacent to the pyridopyrimidinone core, providing additional binding potency beyond that of T790 in WT-EGFR (PDB: 9S3X, 9H42 Figure 8a). Superposition of the crystal structure of **7** with its noncovalent analogue **S13** demonstrated the additive effect of the covalent bond formation. **S13** retained the primary hinge and hydrophobic sandwich interactions of the pyridopyrimidinone core, with a slight perturbation of the noncovalent binding position allowing the core to sit slightly deeper in the pocket (PDB: 9S3X, 9H47 Figure 8b). Additionally, the **7** bound mLTC-EGFR structure showed the activating mutant L858R of the activation loop DFG motif formed a hydrogen bond with Y891 of the C-lobe sandwiched between R836 and D837 of the HRD motif culminating in the overall effect of stabilization of the activation loop (PDB: 9S3X Figure 8c).

**Figure 8:**
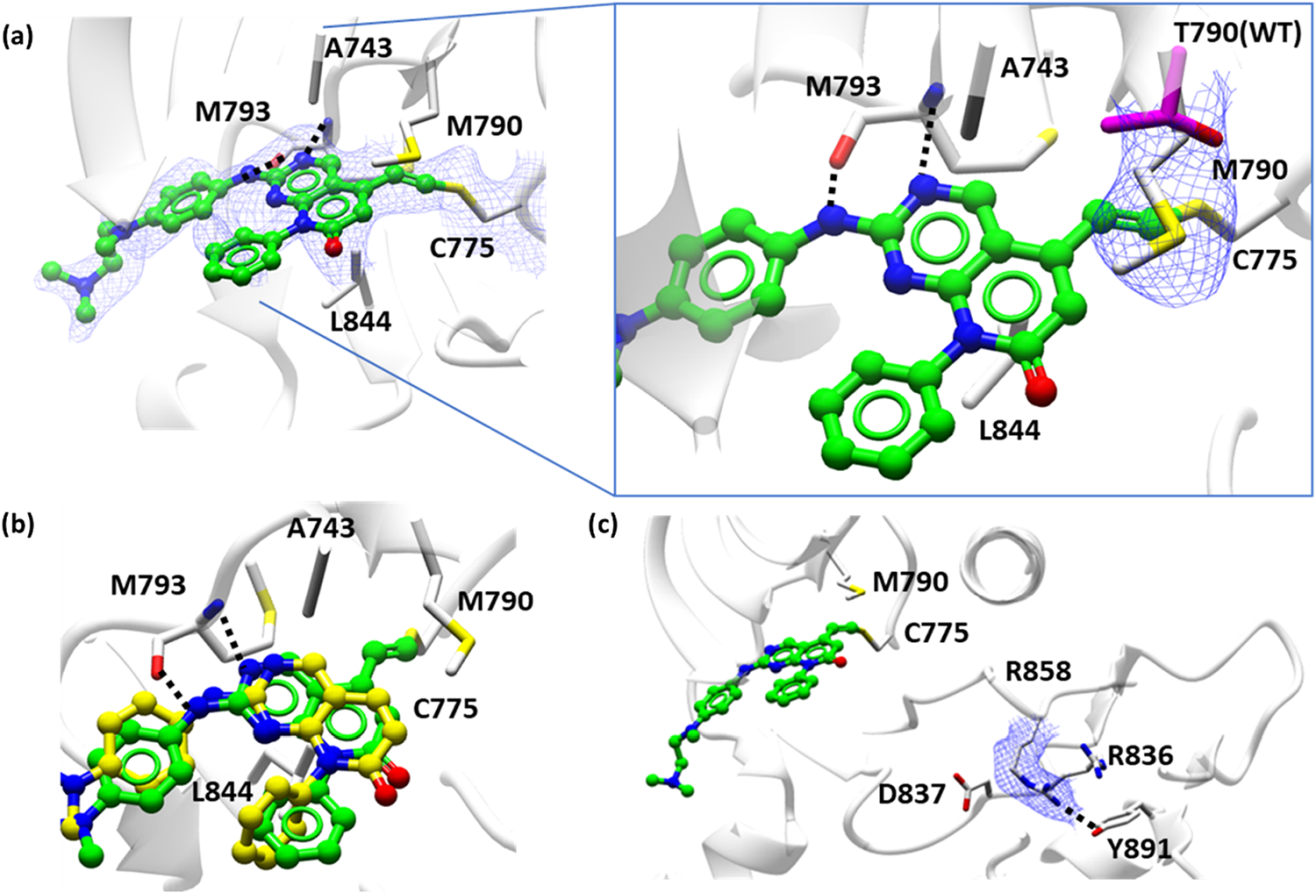
(a) Crystal structure of mLTC-EGFR (white cartoon) bound to **7** (green ball and stick) showing continuous electron density (blue mesh), contoured a 0.7 I/sigma (0.15 e/Å^3^) between C775 (white cylinder) and the alkene linked pyridopyrimidinone core. The hinge region (M793) hydrogen bonding network (black dashes) and hydrophobic sandwich between the L844 and A743 (white cylinder) is retained in the series (PDB: 9S3X). The inset shows electron density (blue mesh), contoured a 0.7 I/sigma (0.15 e/Å^3^) for gatekeeper M790 (white cylinder) of the mutant and T790 (magenta cylinder) of WT-EGFR, showing the enhanced binding of the newly formed alkene covalent bond between the β-carbon stacking with the Cβ and Cγ of the M790 (PDB: 9S3X, 9H42). (b) Crystal structure overlay of covalent adduct **7** (green ball and stick) bound to mLTC-EGFR (white ribbon) with noncovalent analogue **S13** (yellow ball and stick) bound to WT-EGFR; the core scaffold remains engaged at the hinge. (c) Activating mutant L858R white cylinder, electron density in blue mesh, contoured a 0.7 I/sigma (0.15 e/Å^3^) of the activation loops forming a hydrogen bond (black dashes) with Y891 sandwiched in between R836 and D837 (white cylinder) of the HRD motif culminating in the overall effect of stabilization of the activation loop (PDB: 9S3X).

Following these encouraging results, compound **7** was profiled in cellular assays. Western blotting in H1975 cells demonstrated compound **7** to elicit a dose-dependent reduction in EGFR phosphorylation at concentrations ≥100 nM, with a concomitant reduction in ERK1/2 phosphorylation that denotes an impact on signaling downstream of EGFR (Figure 9). Similarly, inhibition of EGFR and ERK1/2 phosphorylation was evident following osimertinib treatment, although at much lower drug concentrations (Figure 9).

**Figure 9:**
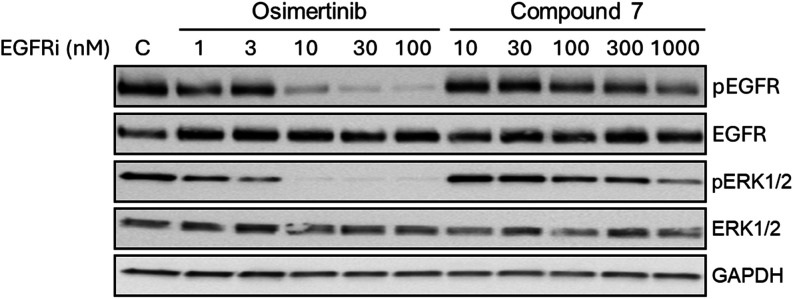
Western blot showing cellular inhibition of EGFR phosphorylation (Tyr1068) and pERK1/2 phosphorylation in H1975 (L858R, T790M) cells following a 4 h exposure to osimertinib, compound **7**, or vehicle denoted as “C”.

The concentration dependence of inhibition of EGFR phosphorylation was demonstrated in an HTRF assay (Figure 10a) in H1975 (L858R, T790M) and CRISPR engineered C797S H1975 cell lines with a >10-fold margin to A431 (WT-EGFR). Cellular target engagement was also established through a nanoBRET assay in L858R/T790M-nanoLuc transfected H293T cells, with an IC_50_ of 324 nM, which is consistent with the inhibition of EGFR phosphorylation in cells (Figure 10b). These data demonstrate that the compounds orthosterically inhibit EGFR kinase activity in adherent tumor cell lines and that the compound series can achieve the necessary selectivity over wild-type EGFR in cells.

**Figure 10:**
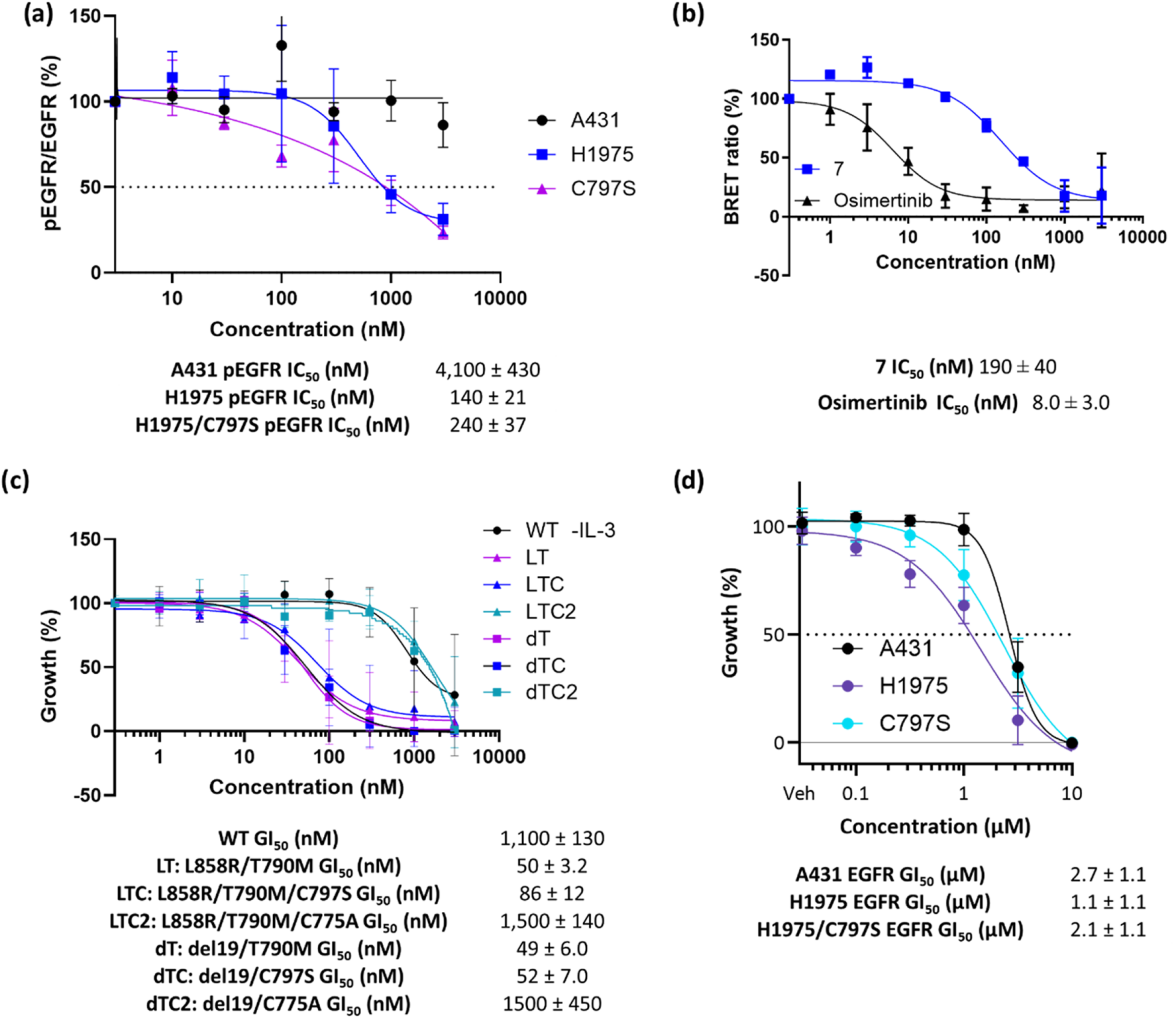
(a) Inhibition of cellular EGFR phosphorylation of EGFR (Tyr1068) as % of control in H1975 (L858R, T790M), H1975 C797S and A431 (WT-EGFR) cell lines (mean ± SD of 4 experiments); (b) NanoBret showing EGFR target engagement for **7** and osimertinib in HEK293T cells transfected with L858R/T790M-nanoLuc reporter EGFR as % of control (mean ± SD of 2 experiments) Data were normalized to vehicle control (0 nM, 100%) and maximal inhibition observed with the positive control compound (0%). Dose–response curves were fitted to normalized data and expressed as a percentage; (c) growth inhibition as % of control against BaF3 EGFR cells lines with L858R/T790M, L858R/T790M/C797S, L858R/T790M/C775A, del19/T790M, del19/T790M/C797S and del19/T790M/C775A EGFR mutations grown in the absence of IL-3 (mean ± SD of 4 experiments); (d) growth inhibition as % of control against H1975 (L858R, T790M), H1975 C797S and A431 (WT-EGFR) cell lines with replenishment of compound every 24 h (mean ± SD of >3 experiments).

With these promising results, **7** was tested in BaF3 EGFR mutant cells lines. Pleasingly, **7** inhibited cell growth at <100 nM in L858R/T790M and L858R/T790M/C797S EGFR mutant lines but showed a significant loss of activity in C775A mutant cell lines, consistent with the compound acting through covalent binding to C775 (Figure 10c). Finally, the compounds were tested in H1975 (L858R, T790M) and CRISPR engineered C797S H1975 cell lines with a 2-fold margin to A431 (WT-EGFR) (Figure 10d).

## Conclusion

This work describes the first compounds that inhibit cellular EGFR phosphorylation exclusively through covalent modification of C775, with selectivity over wild-type EGFR. Given the role of EGFR as a key oncogenic driver in numerous cancers, including nonsmall cell lung cancer tumors, novel inhibitors to target both resistance mutant and activating mutant tumors are needed. By demonstrating the feasibility of targeting C775 and optimizing a cell-active compound, we have identified a novel mode of action for EGFR inhibition, which can be utilized against activating mutant tumors or any of the resistant mutant forms.

## Experimental Section

### Protein Productions

EGFR constructs (Wild type, Triple mutant–C797S, L858R, T790M, crystallography mutant EGFR (T790M, C797S, L858R)) were produced using MultiBac system in SF9 cells grown at double density for 72 h. The cells were collected by centrifuging at 4000*g* for 10 min. Pellets were flash frozen in liquid nitrogen and stored at −70 °C. Buffer A (25 mM Tris-HCl pH 8, 250 mM NaCl, 10% glycerol, 10 mM β-mercaptoEtOH) was supplemented with 5 mM MgCl_2_, 20 mM imidazole, EDTA-free protease inhibitor cocktail (Roche), 0.1 mg/mL DNase, 0.1 mg/mL RNase for lysis. The pellet was unfrozen and resuspended in lysis buffer, sonicated for 5 min at 30% 5 s on, 20 s off and centrifuged at 100,000*g* for 30 min. Supernatant was filtered through 0.45 μm filter. 5 mL of Ni-NTA resin was added to the clarified lysate and incubated for 1 h at 4 °C. The resin was rinsed with wash buffer (buffer A with 20 mM imidazole) and the protein eluted with buffer A with 300 mM imidazole. For ion exchange the protein was dialyzed into a buffer with 25 mM NaCl. Protein was loaded onto Q FF column and eluted with 1 M buffer using a gradient.

### In Vitro TR-FRET Assay

Compounds (dissolved to 100 mM in DMSO) were dispensed into black low volume 384 well assay plates (Corning) over a final concentration range of 100,000, 30,000, 10,000, 3000, 1000, 300, 100, 30, 10, and 3 nM using an Echo 550 (Labcyte). Positive control compound and DMSO as a negative control were dispensed into the first and last well, respectively. Each well was backfilled with DMSO to a final volume of 200 nL, resulting in final assay DMSO concentrations of 1%. 19.8 μL of premixed solution containing final assay concentration of CtermHisTag-mEGFR (Wild type, Triple Mutant–C797S, L858R, T790M) (1.25 nM), Probe (*N*-(4-(4-(3-((4-((3-chloro-4-fluorophenyl)­amino)-7-methoxyquinazolin-6-yl)­oxy)­propyl)­piperazin-1-yl)-4-oxobutyl)-3-(5,5-difluoro-7,9-dimethyl-5*H*-5l4,6l4-dipyrrolo­[1,2-*c*:2′,1′-*f*]­[1,3,2]­diazaborinin-3-yl)­propenamide) (100 nM), and Tb-anti-His Antibody 61HI2TLF (Cisbio Assay) (1:100 dilution) (https://uk.cisbio.eu/media/asset/c/i/cisbio_dd_pi_61hi2tlf-61hi2tla-61hi2tlb.pdf) in a buffer containing 20 mM Tris pH 7.5, 100 mM NaCl, 100 μg/mL bovine serum albumin, was added to each well and incubated with shaking at room temperature for 30 min. Plates were read using a PheraStar FS (BMG Labtech) at Ex337 nm Ex490/520 nm. The data were analyzed using Graphpad Prism/Dotmatics Studies Software. Assays conducted in technical replicate and repeated as a biological duplicate.

### Crystallography

EGFR wild type was crystallized at concentrations around 4.6 mg/mL in 0.085 M Tris pH 7.5–8.2, 110–140 mM sodium acetate, 23.5% PEG 4000, 18% glycerol using microseeding. The mutant EGFR (T790M, C797S, L858R) was crystallized at concentrations around 15 mg/mL in 0.1 M Tris pH 8.5, 150 mM sodium acetate, 25% PEG 400, 20% glycerol using microseeding. Crystals were grown using sitting drop at 20 °C. Compounds were introduced using soaking. Data was collected at Diamond Light Source (Didcot) and processed using CCP4 program suite. **Mass Spectrometry for Covalent Binding Analysis:** Intact protein masses were verified and modification of Cys775 was confirmed using an Agilent 6530 Accurate Mass dual AJS/ESI Q-TOF instrument coupled to an Agilent 1260 Infinity II LC system. Compounds were suspended to 25 mM in 100% DMSO. One μL of compound was added to 2.5 μL of EGFR to achieve the final compound concentration of 1.25 mM in 5% DMSO, in 25 mM Tris pH 8, 150 mM NaCl, 5% Glycerol, before incubating at ambient temperature for 1 h.

**For intact protein analysis**, incubations were quenched by addition of 5 μL of 1.6% formic acid prior to analysis. 1 μL of purified protein (∼1 mg/mL) was injected onto an MS Pac DS-10 Desalter cartridge (Thermo Fisher Scientific), PN: 089170, 2.1 mm × 10 mm for desalting and reversed phase separation at 70 °C. The mobile phase was 0.1% (v/v) formic acid in LC-MS grade water (A) and LC-MS grade acetonitrile (B) with separation performed over 7.5 min. Sample desalting was achieved at 30% B for 2 min at 1 mL/min before reducing the flow rate to 0.2 mL/min for 2 min. Protein elution was achieved at 100% B for 0.5 min and 1 mL/min before re-equilibration at 30% for 1 min. Proteins were detected in positive ion mode using electrospray ionization with a nebulizer pressure of 45 psig, a drying gas flow of 5 L/min, and a source gas temperature of 325 °C. A sheath gas temperature of 400 °C, a gas flow of 11 L/min, a capillary voltage of 3500 V, and a nozzle voltage of 2000 V were also used. Mass spectra were acquired using MassHunter Acquisition software (version B.08.00) over a mass range of 100–3000 *m*/*z* at a rate of 1 spectra/s and 1000 ms/spectrum in the standard mass range (3200 *m*/*z*) at 2 GHz. The instrument had been calibrated over the selected mass range prior to analysis.

**For peptide mapping experiments**, after incubation, 25 μL was diluted with 75 μL of Smart Digest buffer (ThermoScientific), reduced by addition of 5 μL of 0.1 M DTT at 95 °C for 10 min and alkylated by addition of 0.5 M iodoacetamide at room temperature for 30 min, then further reduced by addition of 2.5 μL of 0.1 M DTT. The sample was digested by addition of 2.5 μL Smart Digest Trypsin Protease (Thermo Fisher Scientific) at 70 °C for 2 h. The digests were extracted using HyperSep Spin Tip SPE C18 extraction tips (Thermo Fisher Scientific, PN:60109–412) and 5 μg of digest was injected onto an S7 Acclaim RSLC 120 C18 column (Thermo Fisher Scientific, PN: 068982, 2.1 mm × 100 mm, 2.2 μm, 120 Å) for reversed phase separation at 40 °C and 0.3 mL/min. Mobile phase was 0.1% (v/v) formic acid in LC-MS grade water (A) and acetonitrile (B) with separation performed over a linear gradient of 5–40% B over 15 min, 40–90% B over 3 min followed by a column wash at 90% B for 2 min and equilibration at 5% B for 6 min.

Peptides were detected in positive ion mode using electrospray ionization with nebulizer pressure of 35 psi, drying gas flow of 13 L/min and source gas temperature of 290 °C. Sheath gas temperature of 275 °C and gas flow of 12 L/min, capillary voltage of 4000 V and nozzle voltage of 300 V were also applied. Mass spectra were acquired using MassHunter Acquisition software (version B.08.00) over 100–1700 *m*/*z* range, at a rate of 5 spectra/s and 200 ms/spectrum, using standard mass range mode (3200 *m*/*z*) with extended dynamic range (2 GHz) and collection of both centroid and profile data. MS/MS fragmentation spectra were acquired over 100–3000 *m*/*z* range, at a rate of 3 spectra/s and 333.3 ms/spectrum. Acquired MS and MS/MS spectra were analyzed using Agilent MassHunter BioConfirm software (version B.10.00) against known amino acid sequences for EGFR for confirmation of covalent binding at Cys775. **For the time course study**, 150 μL EGFR (0.15 mg/mL) was incubated with compound 25328 to final concentration of 15 μM in a buffer solution of 20 mM Tris pH 8, 150 mM NaCl, 4% glycerol, 1 mM TCEP, 1% DMSO. The study was performed over a time course of 0, 10, 30, 60, 240, and 1440 min, with 20 μL samples removed and quenched with 1.6% formic acid to achieve a final concentration of 0.4% formic acid solution. Intact protein analysis perform as detailed above.

### Kinetic Analysis

Measurement of *K*_I_, *K*_inact_ and *K*_inact_/*K*_I_ was carried out by AssayQuant Technologies inc.

### ADMET Analysis

Measurement of Log *D*, solubility and human micelle stability was carried out by Cyprotex.

### Cell Culture

A431 cells were purchased from ECACC (Cat No. 85090402), and routinely cultured in DMEM (Sigma-Aldrich, Cat No. D5796) supplemented with 10% FBS (Gibco, Cat No. 10270–106). H1975 cells were purchased from ATCC (Cat No. CRL-5908) and cultured in RPMI (Sigma-Aldrich, Cat No. R8758) supplemented with 10% FBS. Mutant H1975–C797S cells were generated by CRISPR editing and cultured as for the parental line. HEK293T cells were purchased from ATCC (Cat. No. CRL-3216) and were cultured in DMEM (Sigma-Aldrich, Cat No. D6171) supplemented with l-glutamine (2 mM) and 10% FBS.

Murine Ba/F3 cells were purchased from RIKEN (RCB0805)­1–3 and were cultured in RPMI supplemented with 10% FCS and 2 μg/mL puromycin. EGFR WT Ba/F3 additionally required IL-3 (10 μg/mL, Gibco, Cat No, PMC0035).

All cells were maintained at 37 °C with 5% CO_2_.

### Generation of Ba/F3 Mutant Cells

Retroviral constructs for expressing WT or mutated human EGFR were a gift from Matthew Meyerson (Addgene plasmid #11011; http://n2t.net/addgene:11011; RRID:Addgene_11011). pBabe EGFR­(L858R/T790M) was a gift from Matthew Meyerson (Addgene plasmid #32073; http://n2t.net/addgene:32073; RRID:Addgene_32073). pCL-Eco was a gift from Inder Verma (Addgene plasmid #12371; http://n2t.net/addgene:12371; RRID:Addgene_12371).

C797S and C775A mutations were generated by PCR amplification using Addgene plasmid #32073 as a template, mutagenesis primers and CloneAmp Hifi PCR mix from Takara (Cat. No. 639298), followed by DpnI digestion and transformation of Stellar Competent Cells (Takara 636766).

The mutated full-length EGFR cDNAs were confirmed by sequencing.

For retroviral packaging 293T cells were cotransfected with pCL-Eco plasmid and the EGFR expressing plasmids. Viral supernatant was collected 24 h after transfection and used for BaF3 transduction by spinoculation (90 min, 600*g*, at 33 °C) in the presence of Polybrene (8 μg/mL).

Transduced cells were selected in puromycin containing medium (2 μg/mL). Stable cell lines expressing mutant EGFR were cultured in medium with decreasing amount of IL-3 until it was fully removed.

### Western Blotting

#### Lysate Preparation

Cells, seeded on the previous day, were incubated with test compound at the indicated concentrations or DMSO (0.1%) for 4 h. Cells were lysed in Phosphosafe extraction buffer (Merck, Cat. No. 71296) combined with cOmplete Protease Inhibitor Cocktail (Merck, Cat. No. 4693116001) prepared as per the manufacturer’s instructions. Protein concentrations were determined using BCA Protein Assay Kit (Thermo Scientific, Cat. No. 23227). Preparation of lysates and Western blotting was performed twice as independent experiments.

#### Preparation of Samples

Samples were prepared by diluting lysates in PBS to the desired concentration and before adding 4X XT sample buffer. Samples were then denatured at 98 °C for 5 min and then centrifuged to remove the residue. Migration was performed using a 4–20% Criterion TGX Precast Midi Protein Gel, 18 well, 30 μL (Cat. No. 5671094) placed in a Criterion running tank (Cat. No. 1656001) and also using PowerPac HC high-current power supply (Bio-Rad) electrodes. Proteins were transferred to Amersham Protran Premium 0.45 Nitrocellulose membrane (Cat. No. 15269794). The quality of transfer was assessed using Ponceau S Stain (Sigma-Aldrich Cat. No. p7170).

#### Preparation of 10× Tris Glycine Sodium Dodecyl Sulfate (SDS) Running Buffer

Glycine (288 g, 3.84 mol), Trizma base (60.6 g, Sigma-Aldrich, Cat. No. T1503), and SDS (20 g) (Sigma-Aldrich Cat. No. L3771) were added to deuterated water (1.60 L, 88.9 mol) and stirred until dissolved. The mixture was stored at room temperature.

#### Preparation of 1× Tris Glycine SDS Running Buffer

To deuterated water (900 mL, 50 mol) was added 100 mL of the 10× Tris glycine SDS running buffer. It was stored at room temperature.

#### Preparation of the 1× Transfer Buffer

To deuterated water (920 mL, 51 mol) were added 40 mL 25× transfer buffer (Invitrogen Cat. No. LC3675) and methanol (40 mL, 1.0 mol). It was stored at room temperature.

#### Preparation of 10× TBS Solution

Tris-HCl (48.5 g) and NaCl (160 g, 2.70 mol) were added to deuterated water (1.6 L, 88.9 mol) and stirred until dissolved. The solution was then adjusted to pH 7.6 with NaOH, and then the volume was made up to 2 L with additional deuterated water. It was stored at room temperature.

#### Preparation of 1× TBS/T (0.1% v/v) Solution

To 10× TBS solution (100 mL) was diluted with deuterated water (900 mL, 50 mol) and Tween20 (1 mL, Sigma-Aldrich, Cat. No. P5927) was added and stirred until dissolved. It was stored at room temperature.

#### Preparation of Skimmed Milk (5% w/v)

To a falcon tube charged with dried skimmed milk (2.5 g, Marvel) was added 1× TBS/T (0.1 v/v) solution (50 mL).

#### Preparation of Bovine Serum Albumin (BSA) Solution

Bovine serum albumin (BSA) (2.5 g, Sigma-Aldrich) was dissolved in 1× TBS/T (0.1 v/v) solution (50 mL).

#### Preparation of Primary Antibody Solution

Primary antibodies raised to EGFR (CST Cat. No. 4267), phospho-EGFR (CST Cat. No. 3777), phospho-p44/42 MAPK­(ERK1/2) (CST Cat. No. 9101S) and p44/42 MAPK (CST Cat. No. 9102S) were diluted (1:1000) in 5% BSA w/v. Loading control antibodies were prepared in 5% milk w/v with 1× TBS/T (0.1 v/v) solution at a determined optimum concentration.

#### Preparation of Secondary Antibody Solution

Secondary HRP-conjugated antibodies were diluted in 5% milk with w/v in 1× TBS/T (0.1% v/v) solution at a determined optimum concentration.

#### Detection

Proteins were detected using Clarity ECL substrate (Bio-Rad) and imaged using a Licor Odyssey FC.

### HTRF Method

Cells were plated at 20,000 cells per well in 96-well plates and placed at 37 °C with 5% CO_2_. Compounds were reconstituted in DMSO. Once adhered (after 24 h), cells were treated with compounds at a final concentration of 0.1% DMSO in media. Compounds were added to duplicate wells and incubated 37 °C for 3 h with 5% CO_2_. For A431 cells 0.5 h before the end of incubation; 100 ng/mL of EGF (Thermo Fisher, Cat. No. PHG0311) was added to all compound-treated cells as well as control. Compound and media were removed from the cells, and then 50 μL of HTRF lysis buffer was added (lysis buffer was diluted from 4× stock to 1× in deionized (DI) water, with 1% blocking agent also added). Cells were lysed on a plate shaker (2000 rpm) for 30 min at room temperature. pEGFR expression was assessed using the Human Phospho-EGFR (Tyr1068) cellular detection kit (Revvity, Cat. No. 64EG1PEH). Total EGFR expression was monitored using the Human Total EGFR cellular detection kit (Revvity, Cat. No. 64NG1PEH) as per the manufacturer’s instructions. Fluorescence emission was read at two different wavelengths (665 and 620 nm) on a PHERAstar microplate reader (BMG Labtech). Results were calculated as the ratio of pEGFR/total EGFR and then the percentage of 0 μM control.

### Growth Inhibition Assays

For adherent cell assays, each cell line was plated on day 0 in a 96-well plate at a density known to allow for exponential growth over 72 h. After 24 h, the compounds were diluted to the required concentrations in DMEM + 10% FBS media, maintaining a consistent final DMSO concentration of 0.1%. The cells were then incubated for 72 h at 37 °C with 5% CO_2_. Growth inhibition was then assessed using a CyQUANT Direct Cell Proliferation assay (Invitrogen Cat No. C35011) following the manufacturer’s instructions. Fluorescence intensity (ex/em 480/520 nm) values were acquired using a PHERAstar microplate reader reader (BMG Labtech) and values were expressed as a percentage of the average DMSO treated value.

Growth inhibition in Ba/F3 cell lines were assessed in a 384-well format by CellTiter Glo assay (Promega) using the manufacturer’s recommended protocol. Cells were seeded on the day of compound addition and assessed following 72 h incubation including a vehicle only control. Ba/F3 EGFR-WT cells required supplementation with EGF (100 ng/mL, ThermoFisher, Cat No. PHG0311).

### NanoBRET

C-terminal tagged EGFR (L858R, T790M)-NanoLuc vector (Promega, Cat. No. CS1810C788) solution (10 μg/mL) was prepared in Opti-MEM reduced serum media (Gibco, Cat. No. 11058021). Transfection complexes were formed at room temperature by adding TransIT-LT1 (Mirus Bio, Cat. No. MIR2304) to the DNA vector solution (2.8 μL/μg vector). The resulting transfection complex (1 part, volume) was then gently mixed with 10 parts (v/v) of HEK293T cells suspended at a density of 2 × 10^5^ cells/mL in Opti-MEM supplemented with 1% FCS followed by incubation at 37 °C, 5% CO_2_ for 24 h. A 100× DMSO stock of NanoBRET tracer K-5 was diluted into tracer dilution buffer to produce a 20× solution to give a final concentration of 0.25 μM which was previously determined as the optimal concentration. Test compounds were prepared as 10× solutions in Opti-MEM (1% DMSO) and incubated for 2 h before addition of NanoBRET substrate and inhibitor at the manufacturer’s recommended concentrations. Luminescence (acceptor/donor emission) was measured within 15 min of substrate/inhibitor addition using a PHERAstar microplate reader (BMG Labtech). BRET ratios were calculated using luminescence values (610/450 nm). Background subtraction using no-tracer control values. Raw BRET ratios were converted to milliBRET units and multiplied by 1000 which were normalized to vehicle control (100%) and maximal response observed with osimertinib (0%).

### Glutathione Half-Life Assay

The reactivity of compounds, in the presence an excess of glutathione (5.0 mM GSH) at 5 μM was assessed in phosphate buffer at pH 7.4 and 37 °C for 6 h. Compound mixtures of up to 10 compounds and three positive controls (osimertinib, canertinib and afatinib) were prepared in DMSO to a final concentration of 5 μM.

Control sample prepared in 1485 μL of 67 mM PBS at pH 6.7 with 15 μL of the compound mixture. Glutathione samples were prepared in 1335 μL of 67 mM PBS at pH 6.7 with 150 μL of 50 mM GSH and 15 μL of the compound mixture.

The reaction was started by the addition of 15 μL of the compound mixture which contained the three positive controls and up to 10 test compounds at a concentration of 0.5 mM. For example, if 3 compounds were to be tested then the compound mixture was prepared by adding 50 μL of a 10 mM stock solution of each compound in DMSO (150 μl) plus 50 μL of 10 mM DMSO stock solution of each positive control (150 μL) giving a total volume of 300 μL to 700 μL of DMSO to give a mixture of all 6 compounds at 0.5 mM.

After addition of the compound mixture, the reaction was vortex mixed for 5 s and 60 μL removed for the time zero sample and extracted by adding to 60 μL of ice-cold acetonitrile on ice. At time points (5, 10, 15, 30, 60, 90, 120, 180, 240, 360, and 1440 min) the solution was again vortex mixed before removing 60 μL and extracted by adding to 60 μL of ice-cold acetonitrile on ice. Once extracted, samples were placed in a clean dry HPLC vial and the compounds analyzed by LCMS. The reaction was followed by monitoring the loss of the parent by LCMS and the data were fitted to first-order kinetics from which the half-life (*T*_1/2_) was calculated.

#### LCMS Conditions

Separation was carried out using a Zorbax Eclipse Plus C18 2.1 mm × 50 mm 1.8 μm (PN 959757–902) with a mobile phase of 50% (v/v) NH_3_ Acetate pH 5.0. and acetonitrile mixed on the pumps flowing at 400 μL/min. Run times varied between 2 and 4 min depending on the retention of the compounds being studied. The mass spectrometry detection and fragmentation for the compounds was determined by automated compound optimization in Mass Hunter software.

### General Information

Chemicals were purchased from commercial suppliers and used without further purification. Thin layer chromatography (TLC) was performed on aluminum plates coated with 60 F254 silica from Merck. Flash chromatography was carried out using a Biotage SP4, Biotage Isolera, or Varian automated flash system with

Silicycle or GraceResolve normal phase silica gel prepacked columns. Fractions were collected at 254 nm or if necessary, on all wavelengths between 200 and 400 nm. Microwave irradiation was performed in a Biotage Initiator Sixty in sealed vials. Reactions were irradiated at 2.45 GHz and were able to reach temperatures between 60 and 250 °C. Heating was at a rate of 2–5 °C/s, and the pressure was able to reach 20 bar. Final compound purity is >95%.

### Analytical Information

LC-MS analyses were conducted using a Waters Acquity UPLC system with photodiode array (PDA) and evaporating light scattering detector (ELSD). When a 2 min gradient was used, the sample was eluted on an Acquity UPLC BEH C18, 1.7 μm, 2.1 mm × 50 mm, with a flow rate of 0.6 mL/min using 5–95% 0.1% HCOOH in MeCN.

Analytical purity of compounds was determined using Waters XTerra RP18, 5 μm (4.6 mm × 150 mm) column at 1 mL/min using either 0.1% aq. ammonia and MeCN or 0.1% aq. HCOOH and MeCN with a gradient of 5–100% over 15 min.

^1^H NMR spectra were obtained using a Bruker Avance III 500 spectrometer using a frequency of 500 MHz. ^13^C spectra were acquired using the Bruker Avance III 500 spectrometer operating at a frequency of 126 MHz. Multiplicities are indicated by s (singlet), d (doublet), t (triplet), q (quartet), p (pentet), m (multiplet), br (broad) or combinations thereof. Coupling constant values are given in Hz. Homonuclear and heteronuclear two dimensional NMR experiments were used where appropriate to facilitate assignment of chemical shifts. The numbering system used in the assignment of aromatic carbons and hydrogens are done so according to IUPAC nomenclature. All final compounds are >95% pure by HPLC analysis.

### General Procedures

#### General Procedure 1

Methylsulfonyl (1.0 equiv), aniline (1.0–1.5 equiv) and trifluoroacetic acid (1.0–1.5 equiv) in acetonitrile or *n*-butanol (0.1 M) were stirred at 80–110 °C overnight or for the specified time. The reaction mixture was concentrated under reduced pressure.

#### General Procedure 2

TIPS-protected alkyne (1.0 equiv) and potassium fluoride (1.0–20 equiv) in solvent (0.1 M) was stirred at the specified temperature until completion. The solvent was removed under reduced pressure.

#### General Procedure 3

*p*-Fluoronitrophenyl (1.0 equiv), amine (1.0–2.0 equiv) and potassium carbonate (1.5–2.0 equiv) in DMSO (0.6–1.0 M) was stirred at room temperature overnight. The reaction mixture was poured onto water (30 mL) and the resulting precipitate collected by vacuum filtration washing with water and dried in a vacuum over overnight, or extracted with dichloromethane (3 × 50 mL), the combined organic extracts dried (MgSO_4_) and concentrated under reduced pressure.

#### General Procedure 4

To a solution of nitro (1.0 equiv) in ethanol, methanol (0.25 M) or ethanol:acetic acid (1:1, 0.25 M) was added palladium on carbon 5–10 wt % (0.1 equiv). The reaction mixture was stirred at room temperature overnight under an atmosphere of hydrogen. The reaction mixture was filtered through Celite and the filtrate was concentrated under reduced pressure.

### Compound **2**

#### 2-Chloro-4-methoxypyrimdine

To a solution of 2,4-dichloropyrimidine (500 mg, 3.36 mmol, 1.0 equiv) in methanol (1.2 mL, 3.0 M) at 0 °C was added sodium methoxide solution (0.76 mL, 3.36 mmol, 25 wt % in methanol, 1.0 equiv). The reaction mixture was stirred at room temperature overnight then concentrated under reduced pressure. The resulting solid was suspended in diethyl ether and the sodium chloride removed by filtration. The filtrate was concentrated under reduced pressure to yield the title compound as a pale-yellow solid (399 mg, 2.76 mmol, 82% including 15% undesired side product). The product was reacted on without further purification. (ES, *m*/*z*): [M + H]^+^ = 145.0; ^1^H NMR (500 MHz, CDCl_3_) δ = 8.22 (1H, d, *J* = 5.8 Hz), 6.61 (1H, d, *J* = 5.8 Hz), 3.95 (3H, s).

#### 2-Chloro-4-iodo-6-methoxypyrimidine

To a solution of *n*-butyl lithium (0.61 mL, 1.52 mmol, 2.5 M sol. in hexanes, 2.2 equiv) in THF (3.8 mL, 0.4 M) at −30 °C was added 2,2,6,6-tetramethylpiperidine (0.27 mL, 1.59 mmol, 2.3 equiv) dropwise. After 15 min, the reaction mixture was cooled to −78 °C, after which a solution of 2-chloro-4-methoxypyrimdine (100 mg, 0.69 mmol, 1.0 equiv) in THF (0.5 mL, 1.4 M) was added dropwise. After 1 h at −78 °C, a solution of iodine (210 mg, 0.83 mmol, 1.2 equiv) in THF (1 mL, 0.8 M) was added, and stirring continued at −78 °C for 90 min. The reaction mixture was then quenched with a mixture of hydrochloric acid (1 mL, 35% aq. Sol.), ethanol (2 mL) and THF (2 mL) before warming to room temperature. Sodium hydrogen carbonate saturated aqueous solution (20 mL) was added and the mixture concentrated under reduced pressure to remove the THF. The aqueous layer was then extracted with dichloromethane (3 × 50 mL) and the combined organic extracts washed with brine (20 mL), dried (MgSO_4_) and concentrated under reduced pressure. The crude compound was purified by flash column chromatography eluting with ethyl acetate (0–10%) in 40–60 petroleum ether to yield the title compound as a pale orange solid (168 mg, 0.62 mmol, 90%). (ES, *m*/*z*): [M + H]^+^ = 271.0; ^1^H NMR (300 MHz, CDCl_3_) δ = 8.52 (1H, s), 4.01 (3H, s).

#### Ethyl Pyrazolo­[1,5-*a*]­pyridine-3-carboxylate

To a solution of 1-aminopyrdinium (10.0 g, 45.0 mmol, 1.0 equiv) in acetonitrile (60 mL, 0.75 M) was added potassium carbonate (9.33 g, 67.5 mmol, 1.5 equiv) followed by ethyl propiolate (4.56 mL, 45.0 mmol, 1.0 equiv) dropwise. The reaction mixture was stirred at room temperature overnight, then concentrated under reduced pressure. The residue was partitioned between dichloromethane (100 mL) and water (100 mL). The organic phase was washed with brine (50 mL), dried (MgSO_4_) and concentrated under reduced pressure. The crude compound was purified by flash column chromatography to yield the title compound as a red oil (8.62 g, 45.3 mmol, 100%). (ES, *m*/*z*): [M + H]^+^ = 191.1; ^1^H NMR (500 MHz, CDCl_3_) δ = 8.46 (1H, dq, *J* = 6.9, 1.0 Hz), 8.34 (1H, s), 8.10 (1H, dq, *J* = 8.8, 1.0 Hz), 7.35 (1H, ddt, *J* = 8.8, 6.8, 0.9 Hz), 6.89 (1H, td, *J* = 6.9, 1.4 Hz), 4.32 (2H, qd, *J* = 7.1, 0.6 Hz), 1.35 (3H, td, *J* = 7.1, 0.7 Hz); ^13^C NMR (126 MHz, CDCl_3_) δ_C_ = 163.5, 144.9, 140.9, 129.3, 127.3, 119.2, 113.7, 104.0, 60.0, 14.6.

#### Pyrazolo­[1,5-*a*]­pyridine-3-carboxylic Acid

To a solution of ethyl pyrazolo­[1,5-*a*]­pyridine-3-carboxylate (500 mg, 2.63 mmol, 1.0 equiv) in ethanol (9 mL, 0.3 M) was added sodium hydroxide (4.2 mL, 10.5 mmol, 2.5N aq. Sol., 4.0 equiv). The reaction mixture was refluxed for 1 h then concentrated under reduced pressure to remove the ethanol. The remaining aqueous solution was acidified with hydrochloric acid (15% aq. Sol), the resulting precipitate collected by vacuum filtration and washed with water and diethyl ether to yield the title compound as a beige solid (428 mg, 2.63 mmol, 100%) that was reacted on without further purification. (ES, *m*/*z*): [M + H]^+^ = 163.1; ^1^H NMR (500 MHz, CD_3_OD) δ = 8.59–8.54 (1H, m), 8.28 (1H, s), 8.06 (1H, dt, *J* = 8.9, 1.3 Hz), 7.44 (1H, ddd, *J* = 9.0, 6.9, 1.1 Hz), 7.00 (1H, td, *J* = 6.9, 1.5 Hz).

#### Pyrazolo­[1,5-*a*]­pyridine

A solution of ethyl pyrazolo­[1,5-*a*]­pyridine-3-carboxylate (500 mg, 2.63 mmol, 1.0 equiv) in sulfuric acid (0.14 mL, 2.63 mmol, 1.0 equiv) was refluxed for 2 h, then cooled in an ice bath. Sodium hydroxide (210 mg, 5.25 mmol, 50% w/w aq. Sol. 2.0 equiv) was added dropwise, followed by water (20 mL) and sodium hydroxide (20 mL, 2 M aq. Sol.). The aqueous layer was extracted with diethyl ether (3 × 50 mL) and the combined organic layers washed with brine (50 mL), dried (MgSO_4_) and concentrated under reduced pressure. The crude compound was purified by flash column chromatography eluting with ethyl acetate (0–50%) in 40–60 petroleum ether to yield the title compound as a colorless oil (93 mg, 0.78 mmol, 30%). (ES, *m*/*z*): [M + H]^+^ = 119.0; ^1^H NMR (500 MHz, CDCl_3_) δ = 8.41 (1H, dq, *J* = 7.1, 1.1 Hz), 7.88 (1H, d, *J* = 2.3 Hz), 7.47 (1H, dt, *J* = 8.9, 1.3 Hz), 7.02 (1H, ddd, *J* = 8.8, 6.7, 1.1 Hz), 6.67 (td, *J* = 6.8, 1.4 Hz), 6.44 (1H, dd, *J* = 2.3, 1.0 Hz).

#### 3-Iodopyrazolo­[1,5-*a*]­pyridine

To a solution of pyrazolo­[1,5-*a*]­pyridine (286 mg, 2.42 mmol, 1.0 equiv) in acetonitrile (3.0 mL, 0.85 M) was added *N*-iodosuccinimide (599 mg, 2.66 mmol, 1.1 equiv) portionwise. After stirring at room temperature for 1 h, the reaction was quenched with water, then extracted with ethyl acetate (3 × 30 mL). The combined organic layers were washed with sodium hydroxide (20 mL, 2.0 M aq. Sol.), saturated aqueous sodium thiosulfate solution (20 mL), brine (30 mL), dried (MgSO_4_) and concentrated under reduced pressure. The crude compound was purified by flash column chromatography eluting with ethyl acetate (20%) in 40–60 petroleum ether to yield the title compound as a pale-yellow solid (504 mg, 2.07 mmol, 85%). (ES, *m*/*z*): [M + H]^+^ = 245.1; ^1^H NMR (500 MHz, CDCl_3_) δ = 8.48 (1H, dt, *J* = 7.0, 1.1 Hz), 7.98 (1H, s), 7.50 (1H, dt, *J* = 9.0, 1.2 Hz), 7.23 (1H, ddd, *J* = 9.0, 6.8, 1.1 Hz), 6.82 (1H, td, *J* = 6.9, 1.4 Hz).

#### 3-(4,4,5,5-Tetramethyl-1,3,2-dioxaborolan-2-yl)­pyrazolo­[1,5-*a*]­pyridine

To a stirred solution of 3-iodopyrazolo­[1,5-*a*]­pyridine (362 mg, 1.48 mmol, 1.0 equiv) in THF (10 mL, 0.15M) at −10 °C was added dropwise turbo-grignard (0.540 mL, 1.63 mmol, 3.0 M in THF, 1.1 equiv). After 10 min of stirring at −10 °C 2-isopropoxy-4,4,5,5-tetramethyl-1,3,2-dioxaborolane (0.510 mL, 2.52 mmol, 1.7 equiv) was added dropwise and the reaction stirred at −10 °C for 1 h then quenched with saturated aqueous ammonium chloride solution. The aqueous layer was extracted with ethyl acetate (3 × 20 mL) and the combined organic layers were dried (MgSO_4_) and concentrated under reduced pressure. The crude material was purified by flash column chromatography eluting with ethyl acetate (0–10%) in 40–60 petroleum ether to yield the title compound **35** as a pale brown solid (346 mg, 1.41 mmol, 95%). (ES, *m*/*z*): [M + H]^+^ = 245.3; ^1^H NMR (500 MHz, CDCl_3_) δ = 8.54 (1H, dt, *J* = 6.9, 1.1 Hz), 8.26 (1H, s), 7.99 (1H, dt, *J* = 8.9, 1.3 Hz), 7.24 (1H, ddd, *J* = 8.9, 6.7, 1.2 Hz), 6.84 (1H, td, *J* = 6.8, 1.4 Hz), 1.38 (12H, s).

#### 3-(2-Chloro-6-methoxypyrimidin-4-yl)­pyrazolo­[1,5-*a*]­pyridine

3-(4,4,5,5-tetramethyl-1,3,2-dioxaborolan-2-yl)­pyrazolo­[1,5-*a*]­pyridine (538 mg, 2.20 mmol, 1.0 equiv), 2-chloro-4-iodo-6-methoxypyrimidine (894 mg, 3.30 mmol, 1.5 equiv), *tetrakis*(triphenylphosphine)­palladium (0) (76.0 mg, 66.0 μmol, 3 mol %) and cesium carbonate (422 mg, 3.30 mmol, 1.5 equiv) were degassed for 20 min, then dissolved in dioxane (11 mL, 0.2 M) was added and the reaction mixture was heated to 100 °C for 4 h then cooled. The reaction was filtered through Celite washing the filter pad with methanol and concentrated under reduced pressure. The crude compound was purified by flash column chromatography eluting with ethyl acetate (3–10%) in 40–60 petroleum ether to yield the title compound as a solid (493 mg, 1.89 mmol, 86%). (ES, *m*/*z*): [M + H]^+^ = 261.1; ^1^H NMR (500 MHz, CDCl_3_): δ = 8.46 (1H, dt, *J* = 7.0, 1.1 Hz), 8.45 (1H, s), 8.16 (1H, s), 7.55 (1H, dt, *J* = 9.0, 1.2 Hz), 7.23–7.16 (1H, m), 6.81 (1H, td, *J* = 6.9, 1.4 Hz), 4.05 (3H, s).

#### 4-Methoxy-*N*-phenyl-6-(pyrazolo­[1,5-*a*]­pyridin-3-yl)­pyrimidin-2-amine

To a solution of 3-(2-chloro-6-methoxypyrimidin-4-yl)­pyrazolo­[1,5-*a*]­pyridine (200 mg, 0.770 mmol, 1.0 equiv), aniline (70.0 μL, 0.770 mmol, 1.0 equiv) and p-toluene sulfonic acid (0.140 mL, 1.00 mmol, 1.3 equiv) in *iso*-propanol (2.0 mL, 0.4 M) was stirred at 55 °C overnight, then cooled to room temperature and basified with saturated aqueous sodium carbonate solution to pH 8. The aqueous was extracted with dichloromethane (3 × 50 mL) and the combined organic layers washed with brine (10 mL), dried (MgSO_4_) and concentrated under reduced pressure. The crude material was purified by flash column chromatography eluting with ethyl acetate (0–50%) in 40–60 petroleum ether to yield the title compound as a yellow solid (119 mg, 0.380 mmol, 49%). (ES, *m*/*z*): [M + H]^+^ = 318.4; ^1^H NMR (500 MHz, CDCl_3_) δ = 8.51 (1H, d, *J* = 7.0 Hz), 8.16 (1H, s), 7.77–7.66 (2H, m), 7.63 (1H, d, *J* = 8.9 Hz), 7.42–7.35 (2H, m), 7.22–7.15 (2H, m), 7.08 (1H, t, *J* = 7.4 Hz), 6.82 (1H, td, *J* = 6.8, 1.2 Hz), 4.09 (3H, s).

#### 2-(Phenylamino)-6-(pyrazolo­[1,5-*a*]­pyridin-3-yl)­pyrimidin-4-ol

4-methoxy-*N*-phenyl-6-(pyrazolo­[1,5-*a*]­pyridin-3-yl)­pyrimidin-2-amine (92.0 mg, 0.290 mmol, 1.0 equiv) in hydrogen bromide (1 mL, 48% sol. in acetic acid) was heated at reflux for an hour, then cooled and quenched with water (10 mL). The resulting precipitate was collected by vacuum filtration and the dried in the vacuum oven overnight to yield the title compound as a beige solid (64.5 mg, 0.212 mmol, 73%) that was carried forward without further purification. (ES, *m*/*z*): [M + H]^+^ = 304.3; ^1^H NMR (300 MHz, *d* ^6^-DMSO) δ = 8.69 (1H, dt, *J* = 7.0, 1.1 Hz), 8.28 (1H, s), 8.02 (1H, s), 7.79 (1H, dt, *J* = 9.1, 1.3 Hz), 7.66–7.55 (2H, m), 7.47–7.34 (2H, m), 7.27 (1H, ddd, *J* = 9.0, 6.7, 1.1 Hz), 7.22–7.11 (1H, m), 6.93 (1H, td, *J* = 6.8, 1.4 Hz).

#### 4-Chloro-*N*-phenyl-6-(pyrazolo­[1,5-*a*]­pyridin-3-yl)­pyrimidin-2-amine

To a stirred solution of 2-(phenylamino)-6-(pyrazolo­[1,5-*a*]­pyridin-3-yl)­pyrimidin-4-ol (60.0 mg, 0.200 mmol, 1.0 equiv) in toluene (0.20 mL, 1.0 M) was added phosphorus­(V) oxychloride (60.0 μL, 0.600 mmol, 3.0 M) and the reaction mixture heated to 80 °C for 2 h. Once complete, the reaction was cooled in an ice-bath and quenched by the dropwise addition of sodium hydroxide (4 M aq. sol.). The aqueous layer was extracted with dichloromethane (3 × 10 mL) and the combined organic layers were dried (MgSO_4_) and concentrated under reduced pressure. The crude material was purified by flash column chromatography eluting with ethyl acetate (0–100%) in 40–60 petroleum ether to yield the title compound as a yellow oil (36.1 mg, 0.110 mmol, 55%). (ES, *m*/*z*): [M + H]^+^ = 322.3, 324.3; ^1^H NMR (500 MHz, CDCl_3_) δ = 8.55 (1H, dt, *J* = 7.0, 1.1 Hz), 8.48 (1H, s), 8.14 (1H, s), 7.70–7.61 (2H, m), 7.54 (1H, dt, *J* = 8.9, 1.2 Hz), 7.44–7.37 (2H, m), 7.32 (1H, s), 7.24 (1H, ddd, *J* = 9.0, 6.6, 1.1 Hz), 7.13 (1H, tt, *J* = 7.4, 1.1 Hz), 6.89 (1H, d, *J* = 1.3 Hz).

#### 4-Iodo-*N*-phenyl-6-(pyrazolo­[1,5-*a*]­pyridin-3-yl)­pyrimidin-2-amine

To a stirred solution of 2-(phenylamino)-6-(pyrazolo­[1,5-*a*]­pyridin-3-yl)­pyrimidin-4-ol (130 mg, 0.430 mmol, 1.0 equiv) in acetonitrile (1.0 mL, 0.5 M) was added 2,6-lutidine (54.0 μL, 0.470 mmol, 1.1 equiv) followed by trifilic anhydride (79.0 μL, 0.470 mmol, 1.1 equiv) dropwise. Complete triflation was observed after 30 min, as which point sodium iodide (321 mg, 2.14 mmol, 5.0 equiv) was added portionwise followed by trifilic acid (42.0 μL, 0.470 mmol, 1.1 equiv) dropwise. The reaction was stirred at room temperature for 1 h, then diluted with water (10 mL) and quenched with sodium hydroxide (8 N aq. sol.) until pH 10 was achieved. The aqueous layer was extracted with dichloromethane (3 × 20 mL) and the combined organic layers washed with saturated aqueous sodium thiosulfate (20 mL), sodium hydroxide (20 mL, 1 M aq. sol.) and water (20 mL) sequentially, dried (MgSO_4_) and concentrated under reduced pressure. The crude material was purified by flash column chromatography eluting with methanol (0–10%) in dichloromethane to yield the title compound as a yellow solid (146 mg, 0.354 mmol, 82%). (ES, *m*/*z*): [M + H]^+^ = 414.2; ^1^H NMR (300 MHz, CDCl_3_) δ = 8.55 (1H, dt, *J* = 7.0, 1.1 Hz), 8.19 (1H, s), 8.11 (1H, s), 7.82–7.60 (2H, m), 7.47 (1H, dt, *J* = 9.0, 1.2 Hz), 7.44–7.35 (2H, m), 7.24 (1H, ddd, *J* = 8.9, 6.7, 1.1 Hz), 7.17–7.06 (1H, m), 6.88 (1H, td, *J* = 6.9, 1.4 Hz).

#### *N*-Phenyl-4-(pyrazolo­[1,5-*a*]­pyridin-3-yl)-6-((triisopropylsilyl)­ethynyl)­pyrimidin-2-amine

*Bis*(triphenylphosphine)­palladium­(II) dichloride (1.89 mg, 2.90 μmol, 1.0 mol %), copper iodide (0.527 mg, 2.90 μmol, 1.0 mol %) and 4-chloro-*N*-phenyl-6-(pyrazolo­[1,5-*a*]­pyridin-3-yl)­pyrimidin-2-amine (94.0 mg, 0.290 mmol, 1.0 equiv) were degassed for 20 min then dissolved in diisopropylethylamine (1.5 mL, 0.2 M) and DMF (0.73 mL, 0.4 M) and degassed for a further 20 min. Triisopropylsilyl-acetylene (102 μL, 0.435 mmol, 1.5 equiv) was added and the reaction mixture stirred at 55 °C for 2 days. Once cooled, the mixture was quenched with water (20 mL) and extracted with ethyl acetate (3 × 20 mL). The combined organic layers were washed with brine (20 mL), dried (MgSO_4_) and concentrated under reduced pressure. The crude material was purified by flash column chromatography eluting with ethyl acetate (2%) in 40–60 petroleum ether to yield the title compound solid (128 mg, 0.274 mmol, 95%). (ES, *m*/*z*): [M + H]^+^ = 468.5; ^1^H NMR (500 MHz, CDCl_3_) δ = 8.56 (1H, s), 8.53 (1H, dd, *J* = 7.0, 1.2 Hz), 8.19 (1H, s), 7.72–7.67 (2H, m), 7.57 (1H, dt, *J* = 8.9, 1.3 Hz), 7.50 (1H, s), 7.42–7.33 (2H, m), 7.18 (1H, ddd, *J* = 9.0, 6.6, 1.1 Hz), 7.08 (1H, tt, *J* = 7.3, 1.2 Hz), 6.83 (1H, td, *J* = 6.9, 1.4 Hz), 1.09–0.90 (21H, m); ^13^C NMR (126 MHz, CDCl_3_) δ = 158.6, 158.6, 149.2, 141.6, 139.4, 137.9, 129.0, 123.9, 122.7, 119.7, 119.3, 117.7, 112.2, 105.8, 103. 6, 99.6, 18.4, 11.1.

#### 4-Ethynyl-*N*-phenyl-6-(pyrazolo­[1,5-*a*]­pyridin-3-yl)­pyrimidin-2-amine (2)

To a stirred solution of *N*-phenyl-4-(pyrazolo­[1,5-*a*]­pyridin-3-yl)-6-((triisopropylsilyl)­ethynyl)­pyrimidin-2-amine (100 mg, 210 μmol, 1.0 equiv) in THF (4.2 mL, 0.05 M) was added tetrabutylammonium fluoride (315 μL, 315 μmol, 1.5 equiv) dropwise. After 15 min at room temperature, the reaction was concentrated under reduced pressure and the residue dissolved in ethyl acetate (20 mL), washed with brine (10 mL), dried (MgSO_4_) and concentrated under reduced pressure. The crude material was purified by flash column chromatography eluting with ethyl acetate (2%) in 40–60 petroleum ether to yield the title compound pale yellow solid (30.6 mg, 98.3 μmol, 47%). (ES, *m*/*z*): [M + H]^+^ = 312.3; ^1^H NMR (500 MHz, CDCl_3_) δ = 8.63 (1H, s), 8.55 (1H, dt, *J* = 7.1, 1.1 Hz), 8.26 (1H, s), 7.71–7.65 (2H, m), 7.61 (1H, dt, *J* = 9.1, 1.2 Hz), 7.43–7.36 (2H, m), 7.27–7.20 (1H, m), 7.10 (1H, tt, *J* = 7.4, 1.1 Hz), 6.88 (1H, td, *J* = 6.8, 1.3 Hz), 3.31 (1H, s); ^13^C NMR (126 MHz, CDCl_3_) δ = 158.7, 158.5, 147.9, 141.7, 139.1, 137.7, 129.2, 129.1, 124.2, 122.9, 120.0, 119.4, 117.4, 112.4, 105.3, 83.1, 80.9.

### Compound **3**

#### Ethyl 2-(Methylsulfanyl)-4-(phenylamino)­pyrimidine-5-carboxylate

A solution of ethyl 4-chloro-2-(methylsulfanyl)­pyrimidine-5-carboxylate (100 g, 425 mmol, 1.0 equiv), triethylamine (129 g, 1.28 mol, 2.5 equiv) and aniline (59.4 g, 638 mmol, 1.0 equiv) in THF (1.0 L, 0.4 M) was stirred overnight at room temperature. The reaction mixture was diluted with ethyl acetate (250 mL) and water (250 mL). The organic phase was separated and concentrated under reduced pressure. The residue was slurried in 40–60 petroleum ether (100 mL) and filtered. The filter cake was washed with 40–60 petroleum ether and then dried under vacuum to yield the title compound as an off-white solid (115 g, 93%), which was used in next step without any further purification. (ES, *m*/*z*): [M + H]^+^ = 289.9

#### 2-(Methylsulfanyl)-4-(phenylamino)­pyrimidine-5-carboxylic Acid

A solution of ethyl 2-(methylsulfanyl)-4-(phenylamino)­pyrimidine-5-carboxylate (147 g, 507 mmol, 1.0 equiv) and sodium hydroxide (71.0 g, 1.78 mol, 5 equiv) in THF (1.5 L) and water (1.5 L) was stirred at 50 °C overnight. The resulting mixture was concentrated under reduced pressure to remove most of THF. Then the pH value was adjusted to 3–4 with 4 M aqueous hydrochloric acid and either the precipitate was collected by filtration, washing with water or the aqueous solution was extracted with ethyl acetate (3 × 250 mL). The combined organic extracts were concentrated under reduced pressure to afford the title compound as a white solid (110 g, 82%). (ES, *m*/*z*): [M + H]^+^ = 261.9.

#### 5-(1,2,3-Benzotriazole-1-carbonyl)-2-(methylsulfanyl)-*N*-phenylpyrimidin-4-amine

To a stirred solution of carboxylic acid 2-(methylsulfanyl)-4-(phenylamino)­pyrimidine-5-carboxylic acid (30.0 g, 1.0 equiv) and 1*H*-benzo­[*d*]­[1,2,3]­triazole (13.7 g, 1.0 equiv) in dichloromethane (300 mL, 0.3 M) was added EDCI (22.1 g, 1.0 equiv). The resulting mixture was stirred at room temperature for 4 h. The reaction mixture was quenched with water (100 mL), the organic phase was separated and concentrated to dryness under reduced pressure. The residue was purified by flash column chromatography eluting with ethyl acetate (10%) in 40–60 petroleum ether to yield the title compound as a yellow solid (35.6 g, 85% yield). (ES, *m*/*z*): [M + H]^+^ = 363.

#### Ethyl 3-(2-(Methylthio)-4-(phenylamino)­pyrimidin-5-yl)-3-oxopropanoate

To a solution of ethyl acetate (13.8 g, 157 mmol 2.7 equiv) in THF (550 mL, 0.2 M) was slowly added LiHMDS (157 mL, 157 mmol, 1 M in THF, 2.7 equiv) at −78 °C. The mixture was stirred for another 30 min at −78 °C, then a solution of (1*H*-benzo­[*d*]­[1,2,3]­triazol-1-yl)­(2-(methylthio)-4-(phenylamino)­pyrimidin-5-yl)­methanone (22.7 g, 62.7 mmol, 1.0 equiv) in THF (550, 0.2 M) was added to the solution at −78 °C. The resulting mixture was warmed to room temperature and stirred overnight. The reaction was quenched by 1 M aqueous hydrochloric acid then the pH was adjusted to 2 with 6 M aqueous hydrochloric acid and extracted with dichloromethane. The organic layers were combined, dried (Na_2_SO_4_) and concentrated under reduced pressure to yield the title compound as a light yellow solid (12.6 g, 61%). (ES, *m*/*z*): [M + H]^+^ = 332.

#### 5-Hydroxy-2-(methylthio)-8-phenylpyrido­[2,3-*d*]­pyrimidin-7­(8*H*)-one

A solution of ethyl 3-(2-(methylthio)-4-(phenylamino)­pyrimidin-5-yl)-3-oxopropanoate (12.6 g, 38.1 mmol, 1.0 equiv), *N*,*N*-diisopropylethylamine (51.0 mL, 29.2 mmol, 10 equiv) and DBU (6.50 mL, 43.5 mmol, 2.0 equiv) was stirred at 120 °C for 1 h then cooled. The solvent was decanted and the residual thick brown oil was dissolved in water (20 mL). The aqueous solution was acidified to pH 2 with 4 M aqueous hydrochloric acid. The resulting solids were collected by vacuum filtration, washing with water and 40–60 petroleum ether then dried under vacuum to yield the title compound as a light brown solid (10.5 g, 97%). (ES, *m*/*z*): [M + H]^+^ = 286.

#### 2-(Methylthio)-7-oxo-8-phenyl-7,8-dihydropyrido­[2,3-*d*]­pyrimidin-5-yl Trifluoromethanesulfonate

To a stirred solution of 5-hydroxy-2-(methylthio)-8-phenylpyrido­[2,3-*d*]­pyrimidin-7­(8*H*)-one (10.9 g, 38.3 mmol, 1.0 equiv) and triethylamine (5.82 g, 57.5 mmol, 2.0 equiv) in dichloromethane (220 mL, 0.5 M) was added triflic anhydride (16.2 g, 57.5 mmol, 1.5 equiv) dropwise at 0 °C under a nitrogen atmosphere. The resulting mixture was stirred for 2 h at room temperature then concentrated under reduced pressure. The crude material was purified by flash column chromatography eluting with ethyl acetate (0–50%) in 40–60 petroleum ether to yield the title compound as a light yellow solid (8.80 g, 55%). (ES, *m*/*z*): [M + H]^+^ = 418.

#### 2-(Methylthio)-8-phenyl-5-((triisopropylsilyl)­ethynyl)­pyrido­[2,3-*d*]­pyrimidin-7­(8*H*)-one

A solution of 2-(methylthio)-7-oxo-8-phenyl-7,8-dihydropyrido­[2,3-*d*]­pyrimidin-5-yl trifluoromethanesulfonate (2.00 g, 4.80 mmol, 1.0 equiv), bis­(triphenylphosphine)­palladium­(II) dichloride (338 mg, 0.480 mmol, 0.10 equiv), triisopropylsilyl acetylene (1.75 g, 9.60 mmol, 2.0 equiv), copper­(I) iodide (92.0 mg, 0.480 mmol, 0.10 equiv) and *N*,*N-*diisopropylethylamine (10 mL, 3.0 equiv) in DMF (20 mL, 0.2 M). The resulting mixture was stirred for 3 h at 80 °C under a nitrogen atmosphere. The resulting mixture was filtered, the filter cake was washed with dichloromethane (2 × 10 mL).  The crude material was purified by reverse phase flash column chromatography eluting with acetonitrile (25–100%) in water (0.1% ammonium carbonate) to yield the title compound as a light-yellow solid (1.71 g, 80%). (ES, *m*/*z*): [M + H]^+^ = 450.

#### 2-(Methylsulfonyl)-8-phenyl-5-((triisopropylsilyl)­ethynyl)­pyrido­[2,3-*d*]­pyrimidin-7­(8*H*)-one

A solution of 2-(methylthio)-8-phenyl-5-((triisopropylsilyl)­ethynyl)­pyrido­[2,3-*d*]­pyrimidin-7­(8*H*)-one (1.70 g, 3.78 mmol, 1.0 equiv) and *m*-CPBA (1.96 g, 11.4 mmol, 3.0 equiv) in dichloromethane (90 mL, 0.1 M) was stirred at room temperature for 1–2 h. The reaction mixture was quenched with saturated aqueous sodium thiosulfate solution (20 mL) and extracted with dichloromethane (3 × 30 mL). The combined organic extracts were washed with saturated aqueous sodium thiosulfate solution (3 × 20 mL) and saturated aqueous sodium hydrogen carbonate solution (3 × 30 mL). The organic layer was dried (MgSO_4_) and concentrated under reduced pressure to yield the title compound as a yellow solid (1.62 g, 89%). The crude material was carried into the subsequent step without further purification. (ES, *m*/*z*): [M + H]^+^ = 482.

#### 2-((2-Methoxyphenyl)­amino)-8-phenyl-5-((triisopropylsilyl)­ethynyl)­pyrido­[2,3-*d*]­pyrimidin-7­(8*H*)-one

General procedure 1 was applied to 2-(methylsulfonyl)-8-phenyl-5-((triisopropylsilyl)­ethynyl)­pyrido­[2,3-*d*]­pyrimidin-7­(8*H*)-one (1.32 g, 2.74 mmol) with 2-methoxyaniline (338 mg, 2.74 mmol) and trifluoroacetic acid (313 mg, 2.74 mmol) in 2-butanol (10 mL). The reaction mixture was stirred at 110 °C for 16 h. The crude material was purified by reverse phase flash column chromatography eluting with acetonitrile (25–100%) in water (0.1% ammonium carbonate) to yield the title compound as a yellow solid (1.06 g, 74%). (ES, *m*/*z*): [M + H]^+^ = 525.

#### 5-Ethynyl-2-[(2-methoxyphenyl)­amino]-8-phenylpyrido­[2,3-*d*]­pyrimidin-7-one (3)

General procedure 2 was applied to 2-[(2-methoxyphenyl)­amino]-8-phenyl-5-[2-(triisopropylsilyl)­ethynyl]­pyrido­[2,3-*d*]­pyrimidin-7-one (823 mg, 1.56 mmol), potassium fluoride (9.12 g, 156 mmol) in THF and water (22.0 mL, 10:1). The resulting solution was stirred for 48 h at 40 °C. The crude material was purified by reverse phase flash column chromatography eluting with acetonitrile (25–100%) in water (0.1% ammonium carbonate) to yield the title compound as a yellow solid (520 mg, 90%). (ES, *m*/*z*): [M + H]^+^ = 369; ^1^H NMR (500 MHz, CDCl_3_) δ 8.82 (s, 1H), 8.01 (s, 1H), 7.58–7.47 (m, 3H), 7.39 (s, 1H), 7.27–7.19 (m, 2H), 6.85–6.78 (m, 1H), 6.73 (dd, *J* = 8.1, 1.4 Hz, 1H), 6.69 (s, 1H), 6.41 (s, 1H), 3.78 (s, 3H), 3.61 (s, 1H); ^13^C NMR (126 MHz, CDCl_3_) δ 162.57, 158.67, 157.44, 156.80, 147.66, 136.24, 130.00, 129.76, 128.71, 128.63, 127.99, 122.73, 122.42, 120.58, 118.24, 109.65, 105.84, 87.86, 76.61, 55.70.

### Compound **4**

#### 2-((2-Methoxyphenyl)­amino)-8-phenyl-5- ((triisopropylsilyl)­ethynyl)­pyrido­[2,3-*d*]­pyrimidin-7­(8*H*)-one

General procedure 1 was applied to 2-(methylsulfonyl)-8-phenyl-5-((triisopropylsilyl)­ethynyl)­pyrido­[2,3-*d*]­pyrimidin-7­(8*H*)-one (250 mg, 0.52 mmol) with aniline (64 mg, 0.68 mmol), trifluoroacetic acid (60 mg, 0.52 mmol) and 2-butanol (2.0 mL). The crude material was purified by reverse phase flash column chromatography eluting with acetonitrile (10–100%) in water (0.1% ammonium carbonate) to yield the title compound as a yellow solid (150 mg, 55%). (ES, *m*/*z*): [M + H]^+^ = 495.3.

#### 5-Ethynyl-2-((2-methoxyphenyl)­amino)-8-phenylpyrido­[2,3-*d*]­pyrimidin-7­(8*H*)-one (4)

General procedure 2 was applied to 2-((2-methoxyphenyl)­amino)-8-phenyl-5-((triisopropylsilyl)­ethynyl)­pyrido­[2,3-*d*]­pyrimidin-7­(8*H*)-one (150 mg, 0.286 mmol), potassium fluoride (83.0 mg, 1.43 mmol), methanol (10.0 mL) and water (1.0 mL). The crude material was purified by reverse phase flash column chromatography eluting with acetonitrile (10–100%) in water (0.1% ammonium carbonate) then lyophilized to yield the title compound as a yellow solid (30.4 mg, 28%). (ES, *m*/*z*): [M + H]^+^ = 339.1; ^1^H NMR (500 MHz, DMSO) δ 10.21 (s, 1H), 8.85 (s, 1H), 7.64–7.54 (m, 3H), 7.40–7.34 (m, 2H), 7.27 (s, 2H), 6.96 (s, 2H), 6.85 (t, *J* = 7.3 Hz, 1H), 6.70 (s, 1H), 5.14 (s, 1H); ^13^C NMR (126 MHz, DMSO) δ 173.24, 169.39, 162.18, 157.24, 156.95, 152.62, 139.79, 129.78, 129.39, 128.71, 128.59, 121.95, 102.74, 92.15, 77.02, 76.64.

### Compound **5**

#### 1-(3-Methoxy-4-nitrophenyl)-4-methylpiperazine

General procedure 3 was applied to 4-fluoro-2-methoxy-1-nitrobenzene (1.50 g, 8.76 mmol) with morpholine (0.75 mL, 8.76 mmol) and potassium carbonate (2.00 g, 13.1 mmol) in DMSO (18 mL) to yield the title compound as a yellow solid (1.62 g, 6.79 mmol, 78%) which was used without further purification.

(ES, *m*/*z*): [M + H]^+^ = 239.1.

#### 2-Methoxy-4-morpholinoaniline

General procedure 4 was applied to 1-(3-methoxy-4-nitrophenyl)-4-methylpiperazine (1.00 g, 4.20 mmol) with palladium on carbon 5 wt % (223 mg, 0.200 mmol) in ethanol (21.0 mL). The crude material was purified by flash column chromatography eluting with methanol (0–20%) in dichloromethane to yield the title compound as a purple oil (588 mg, 2.82 mmol, 67%).

#### 2-(Methylthio)-8-phenyl-5-((trimethylylsilyl)­ethynyl)­pyrido­[2,3-*d*]­pyrimidin-7­(8*H*)-one

A solution of 2-(methylthio)-7-oxo-8-phenyl-7,8-dihydropyrido­[2,3-*d*]­pyrimidin-5-yl trifluoromethanesulfonate (1.05 g, 2.51 mmol, 1.0 equiv), *bis*(triphenylphsophine)­palladium­(II) dichloride (176 mg, 0.251 mmol, 0.10 equiv), trimethylsilylacetylene (70.0 μL, 5.00 mmol, 2.0 equiv), copper­(I) iodide (48.0 mg, 0.251 mmol, 0.10 equiv) and *N*,*N*-diisopropylethylamine (1.1 mL, 3.0 equiv) in DMF (12.5 mL, 0.2 M). The resulting mixture was stirred for 3 h at 80 °C under a nitrogen atmosphere. The resulting mixture was filtered, the filter cake was washed with dichloromethane (2 × 10 mL).  The crude material was purified by flash column chromatography eluting with ethyl acetate (25–100%) in 40–60 petroleum ether to yield the title compound as a light-yellow solid (798 mg, 87%). (ES, *m*/*z*): [M + H]^+^ = 366.2.

#### 2-(Methylsulfonyl)-8-phenyl-5-((trimethylsilyl)­ethynyl)­pyrido­[2,3-*d*]­pyrimidin-7­(8*H*)-one

A solution of 2-(methylthio)-8-phenyl-5-((trimethylsilyl)­ethynyl)­pyrido­[2,3-*d*]­pyrimidin-7­(8*H*)-one (798 mg, 2.18 mmol, 1.0 equiv) and *m*-CPBA (1.47 mg, 6.54 mmol, 3.0 equiv) in dichloromethane (22.0 mL, 0.1M) was stirred at room temperature for 1–2 h. The reaction mixture was quenched with saturated aqueous sodium thiosulfate solution (20 mL) and extracted with dichloromethane (3 × 30 mL). The combined organic extracts were washed with saturated aqueous sodium thiosulfate solution (3 × 20 mL) and saturated aqueous sodium hydrogen carbonate solution (3 × 30 mL). The organic layer was dried (MgSO_4_) and concentrated under reduced pressure to yield the title compound as a yellow solid (1.05 g, 100%). The crude material was carried into the subsequent step without further purification. (ES, *m*/*z*): [M + H]^+^ = 398.2.

#### 2-((2-Methoxy-4-morpholinophenyl)­amino)-8-phenyl-5-((trimethylsilyl)­ethynyl)­pyrido­[2,3-*d*]­pyrimidin-7­(8*H*)-one

General procedure 1 was applied to 2-(methylsulfonyl)-8-phenyl-5-((triimethylsilyl)­ethynyl)­pyrido­[2,3-*d*]­pyrimidin-7­(8*H*)-one (400 mg, 1.01 mmol) with 2-methoxy-4-morpholinoaniline (336 mg, 1.52 mmol), trifluoroacetic acid (120 μL, 1.52 mmol) and acetonitrile (10 mL). The crude material was purified by flash column chromatography eluting with methanol (0–10%) in dichloromethane to yield the title compound as a red solid (184 mg, 0.350 mmol, 35%). (ES, *m*/*z*): [M + H]^+^ = 526.4.

#### 5-Ethynyl-2-((2-methoxy-4-morpholinophenyl)­amino)-8-phenylpyrido­[2,3-*d*]­pyrimidin-7­(8*H*)-one (5)

A solution of 2-((2-methoxy-4-morpholinophenyl)­amino)-8-phenyl-5-((trimethylsilyl)­ethynyl)­pyrido­[2,3-*d*]­pyrimidin-7­(8*H*)-one (77.0 mg, 0.146 mmol) and potassium carbonate (20.0 mg, 0.146 mmol) in methanol (1.5 mL) was stirred at room temperature for 30 min. The reaction mixture was quenched with water (30 mL), and the resulting solids were collected by vacuum filtration and dried under vacuum at 40 °C overnight to yield the title compound as a red solid (54.5 mg, 0.120 mmol, 82%). (ES, *m*/*z*): [M + H]^+^ = 454.3; ^1^H NMR (500 MHz, DMSO) δ 8.76 (s, 1H), 8.32 (d, *J* = 7.4 Hz, 1H), 7.60–7.50 (m, 2H), 7.34–7.28 (m, 2H), 7.15 (s, 1H), 6.63 (s, 1H), 6.57–6.52 (m, 1H), 5.93 (s, 1H), 5.10 (s, 1H), 3.79–3.71 (m, 8H), 3.34 (s, 3H); ^13^C NMR (126 MHz, DMSO) δ 170.21, 162.18, 157.24, 157.00, 153.29, 148.20, 136.79, 130.11, 129.69, 129.61, 129.36, 128.59, 121.47, 120.14, 106.41, 105.50, 99.85, 92.00, 77.04, 66.58, 56.14, 49.44.

### Compound **6**

#### 4-(4-Nitrophenyl)­morpholine

General procedure 3 was applied to 4-fluoro-1-nitrobenzene (1.55 g, 14.6 mmol) with morpholine (1.27 mL, 14.6 mmol) and potassium carbonate (3.30 g, 21.9 mmol) in DMSO (14.6 mL). The title compound, a bright yellow solid, was carried forward without further purification (2.29 g, 11.0 mmol, 75%). (ES, *m*/*z*): [M + H]^+^ = 209.1

#### 4-Morpholinoaniline

General procedure 4 was applied to 4-(4-nitrophenyl)­morpholine (1.50 g, 7.20 mmol) with palladium on carbon 5 wt % (380 mg, 0.360 mmol) in ethanol (36 mL). The crude material was purified by flash column chromatography eluting with methanol (0–20%) in dichloromethane to yield the title compound as a purple oil (1.28 g, 7.20 mmol, 100%).

#### 2-((4-Morpholinophenyl)­amino)-8-phenyl-5-((triisopropylsilyl)­ethynyl)­pyrido­[2,3-*d*]­pyrimidin-7­(8*H*)-one

General procedure 1 was applied to 2-(methylsulfonyl)-8-phenyl-5-((triisopropylsilyl)­ethynyl)­pyrido­[2,3-*d*]­pyrimidin-7­(8*H*)-one (500 mg, 1.04 mmol) with 4-morpholinoaniline (194 mg, 1.09 mmol) and trifluoroacetic acid (80.0 μL, 1.09 mmol) in acetonitrile (10 mL). The crude material was purified by flash column chromatography eluting with methanol (0–20%) in dichloromethane to yield the title compound as an orange solid (186 mg, 0.320 mmol, 31%). (ES, *m*/*z*): [M + H]^+^ = 580.5.

#### 5-Ethynyl-2-((4-morpholinophenyl)­amino)-8-phenylpyrido­[2,3-*d*]­pyrimidin-7­(8*H*)-one (6)

General procedure 2 was applied to 2-((4-morpholinophenyl)­amino)-8-phenyl-5-((triisopropylsilyl)­ethynyl)­pyrido­[2,3-*d*]­pyrimidin-7­(8*H*)-one (113 mg, 0.190 mmol) with potassium fluoride (226 mg, 3.90 mmol) in DMF (2.0 mL) and methanol (2.0 mL). The crude material was purified by flash column chromatography eluting with methanol (0–20%) in dichloromethane to yield the title compound as a red solid (31.4 mg, 74.1 μmol, 39%). (ES, *m*/*z*): [M + H]^+^ = 424.3; ^1^H NMR (500 MHz, DMSO-*d*_6_) δ 10.07 (s, 1H), 8.79 (s, 1H), 7.60 (d, *J* = 7.2 Hz, 3H), 7.41–7.29 (m, 2H), 7.12 (d, *J* = 8.1 Hz, 2H), 6.64 (s, 1H), 6.53 (d, *J* = 7.6 Hz, 2H), 5.13 (s, 1H), 3.72 (dd, *J* = 5.8, 3.8 Hz, 4H), 2.96 (s, 4H); ^13^C NMR (126 MHz, DMSO) δ 162.21, 157.14, 157.03, 150.71, 144.05, 137.06, 132.18, 130.14, 129.75, 129.40, 128.64, 121.24, 120.02, 115.48, 112.73, 91.97, 77.12, 66.58, 49.45.

### Compound **7**

#### *N* ^1^,*N* ^1^,*N* ^2^-Trimethyl-*N* ^2^-(4-nitrophenyl)­ethane-1,2-diamine

General procedure 3 was applied to 4-fluoro-1-nitrobenzene (3.10 mL, 29.2 mmol) with *N* ^1^,*N* ^1^,*N* ^3^-trimethylethylenediamine (3.70 mL, 29.2 mmol) and potassium carbonate (6.66 g, 43.8 mmol) in DMSO (29 mL). The crude material was purified by flash column chromatography eluting with methanol (0–10%) in dichloromethane to yield the title compound as a yellow solid (6.35 g, 28.4 mmol, 97%). (ES, *m*/*z*): [M + H]^+^ = 224.2.

#### *N* ^1^-(2-(Dimethylamino)­ethyl)-*N* ^1^-methylbenzene-1,4-diamine

General procedure 4 was applied to *N* ^1^,*N* ^1^,*N* ^2^-trimethyl-*N* ^2^-(4-nitrophenyl)­ethane-1,2-diamine (6.36 g, 28.5 mmol) with palladium on carbon 5 wt % (303 mg, 0.285 mmol) in ethanol (142.0 mL). The crude material was purified by flash column chromatography eluting with methanol (0–20%) in dichloromethane to yield the title compound as a purple oil (4.19 g, 21.7 mmol, 76%).

#### 2-((4-((2-(Dimethylamino)­ethyl)­(methyl)­amino)­phenyl)­amino)-8-phenyl-5-((triisopropylsilyl)­ethynyl)­pyrido­[2,3-*d*]­pyrimidin-7­(8*H*)-one

General procedure 1 was applied to 2-(methylsulfonyl)-8-phenyl-5-((triisopropylsilyl)­ethynyl)­pyrido­[2,3-*d*]­pyrimidin-7­(8*H*)-one (974 mg, 2.02 mmol) with *N* ^1^-(2-(dimethylamino)­ethyl)-*N* ^1^-methylbenzene-1,4-diamine (410 mg, 2.12 mmol), trifluoroacetic acid (162 μL, 2.12 mmol) and acetonitrile (20 mL). The crude material was purified by flash column chromatography eluting with methanol (0–10%) in dichloromethane to yield the title compound as a red solid (577.1 mg, 0.969 mmol, 48%). (ES, *m*/*z*): [M + H]^+^ = 595.5.

#### 2-((4-((2-(Dimethylamino)­ethyl)­(methyl)­amino)­phenyl)­amino)-5-ethynyl-8-phenylpyrido­[2,3-*d*]­pyrimidin-7­(8*H*)-one (7)

General procedure 2 was applied to 2-((4-((2-(dimethylamino)­ethyl)­(methyl)­amino)­phenyl)­amino)-8-phenyl-5-((triisopropylsilyl)­ethynyl)­pyrido­[2,3-*d*]­pyrimidin-7­(8*H*)-one (577 mg, 0.973 mmol) with potassium fluoride (1.13 g, 19.4 mmol) in DMF (10 mL). The crude material was purified by flash column chromatography eluting with methanol (0–10%) in dichloromethane to yield the title compound as a red solid (302 mg, 0.689 mmol, 71%). (ES, *m*/*z*): [M + H]^+^ = 439.3; ^1^H NMR (500 MHz, DMSO) δ 9.97 (s, 1H), 8.76 (s, 1H), 7.59 (t, *J* = 7.6 Hz, 2H), 7.52 (t, *J* = 7.4 Hz, 1H), 7.35 (d, *J* = 7.6 Hz, 2H), 7.07 (d, *J* = 9.0 Hz, 2H), 6.61 (s, 1H), 6.28 (d, *J* = 9.1 Hz, 2H), 5.12 (s, 1H), 3.30 (d, *J* = 7.4 Hz, 2H), 2.80 (s, 3H), 2.30 (t, *J* = 7.3 Hz, 2H), 2.20 (s, 6H); ^13^C NMR (126 MHz, DMSO) δ 162.23, 158.80, 157.06, 145.05, 138.76, 137.18, 130.16, 129.68, 129.46, 129.08, 128.41, 120.81, 120.48, 111.95, 104.92, 91.87, 77.17, 55.86, 50.70, 46.09, 38.73

### Compound **8**

#### 1-(3-Methoxy-4-nitrophenyl)-4-methylpiperazine

General procedure 3 was applied to 4-fluoro-3-methoxy-1-nitrobenzene (1.50 g, 8.76 mmol) with 1-methylpiperazine (0.96 mL, 8.76 mmol) and potassium carbonate (1.98 g, 13.1 mmol) in DMSO (18 mL) to yield the title compound as a bright yellow solid (2.03 g, 92%) which was carried forward without further purification.

#### 2-Methoxy-4-(4-methylpiperazin-1-yl)­aniline

General procedure 4 was applied to 1-(3-methoxy-4-nitrophenyl)-4-methylpiperazine (1.00 g, 3.98 mmol) with palladium on carbon 5 wt % (212 mg, 0.199 mmol) in ethanol (20 mL). The crude material was purified by flash column chromatography eluting with methanol (0–20%) in dichloromethane to yield the title compound as a purple oil (872 mg, 3.94 mmol, 99%).

#### 2-((2-Methoxy-4-(4-methylpiperazin-1-yl)­phenyl)­amino)-8-phenyl-5-((trimethylsilyl)­ethynyl)­pyrido­[2,3-*d*]­pyrimidin-7­(8*H*)-one

General procedure 1 was applied to 2-(methylsulfonyl)-8-phenyl-5-((trimethylylsilyl)­ethynyl)­pyrido­[2,3-*d*]­pyrimidin-7­(8*H*)-one (250 mg, 0.629 mmol) with 2-methoxy-4-(4-methylpiperazin-1-yl)­aniline (209 mg, 0.943 mmol), trifluoroacetic acid (70.0 μL, 0.943 mmol) and acetonitrile (6.3 mL). The crude material was purified by flash column chromatography eluting with methanol (0–10%) in dichloromethane to yield the title compound as a red solid (169.8 mg, 0.315 mmol, 50%)

(ES, *m*/*z*): [M + H]^+^ = 539.4

#### 5-Ethynyl-2-(2-methoxy-4-(4-methylpiperazine-1-ylphenyl)­amino)-8-phenylpyrido­[2,3-*d*]­pyrimidin-7­(8*H*)-one (8)

A solution of 2-((2-methoxy-4-(4-methylpiperazin-1-yl)­phenyl)­amino)-8-phenyl-5-((trimethylsilyl)­ethynyl)­pyrido­[2,3-*d*]­pyrimidin-7­(8*H*)-one (49.7 mg, 92.2 μmol) and potassium carbonate (13.0 mg, 92.2 μmol) in methanol was stirred at room temperature overnight. The reaction mixture was concentrated under reduced pressure, suspended in water (20 mL) and extracted with dichloromethane (3 × 20 mL). The combined organic layers were dried (MgSO4) and concentrated under reduced pressure. The crude material was triturated with diethyl ether to yield the title compound as a red solid (18.5 mg, 39.7 μmol, 43%). (ES, *m*/*z*): [M + H]^+^ = 467.3; ^1^H NMR (500 MHz, DMSO) δ 8.75 (s, 1H), 8.26 (s, 1H), 7.56 (q, *J* = 6.8 Hz, 4H), 7.33–7.26 (m, 2H), 7.16–7.11 (m, 1H), 6.61 (s, 1H), 6.51 (d, *J* = 2.7 Hz, 1H), 5.91 (s, 1H), 5.03 (s, 1H), 3.78 (s, 3H), 3.05 (t, *J* = 4.9 Hz, 5H), 2.46 (t, *J* = 5.0 Hz, 5H), 2.24 (s, 3H); ^13^C NMR (126 MHz, DMSO) δ 162.14, 157.22, 156.98, 136.77, 130.18, 129.57, 129.31, 128.52, 121.39, 119.95, 106.71, 105.48, 100.00, 91.82, 76.97, 56.09, 55.11, 49.07, 46.24.

### Compound **9**

#### 2-((4-(4-Methylpiperazin-1-yl)­phenyl)­amino)-8-phenyl-5-((triisopropylsilyl)­ethynyl)­pyrido­[2,3-*d*]­pyrimidin-7­(8*H*)-one

General procedure 1 was applied to 2-(methylsulfonyl)-8-phenyl-5-((triisopropylylsilyl)­ethynyl)­pyrido­[2,3-*d*]­pyrimidin-7­(8*H*)-one (1.07 g, 2.23 mmol) with 4-(4-methylpiperazin-1-yl)­aniline (511 mg, 2.68 mmol), trifluoroacetic acid (205 μL, 2.68 mmol) and acetonitrile (11 mL). The crude material was purified by flash column chromatography eluting with methanol (0–10%) in dichloromethane to yield the title compound as a red solid (650 mg, 1.09 mmol, 29%). (ES, *m*/*z*): [M + H]^+^ = 593.8

#### 5-Ethynyl-2-((4-(4-methylpiperazin-1-yl)­phenyl)­amino)-8-phenylpyrido­[2,3-*d*]­pyrimidin-7­(8*H*)-one (9)

General procedure 2 was applied to 2-((4-(4-methylpiperazin-1-yl)­phenyl)­amino)-8-phenyl-5-((triisopropylsilyl)­ethynyl)­pyrido­[2,3-*d*]­pyrimidin-7­(8*H*)-one (300 mg, 0.507 mmol) with potassium fluoride (294 mg, 5.07 mmol) in DMF (2.5 mL). The crude material was purified by flash column chromatography eluting with methanol (0–10%) in dichloromethane to yield the title compound as a red solid (30.3 mg, 69.5 μmol, 13%). (ES, *m*/*z*): [M + H]^+^ = 437.6; ^1^H NMR (500 MHz, DMSO) δ 10.13 (s, 1H), 8.88 (s, 1H), 7.69 (d, *J* = 7.6 Hz, 3H), 7.46–7.40 (m, 2H), 7.19 (s, 2H), 6.72 (s, 1H), 6.61 (s, 2H), 5.19 (s, 1H), 3.08 (s, 4H), 2.53 (t, *J* = 5.0 Hz, 4H), 2.31 (s, 3H); ^13^C NMR (126 MHz, DMSO) δ 162.2, 157.1, 157.0, 146.8, 137.1, 131.9, 130.2, 129.7, 129.4, 128.6, 126.0, 121.2, 120.0, 115.7, 105.2, 91.9, 77.1, 55.1, 49.1, 46.3.

### Compound (**10**)

#### 3-Methoxy-*N*-methyl-4-nitroaniline

General procedure 3 was applied to 5-fluoro-2-nitroanisole (1.50 g, 8.77 mmol) with methyl amine hydrochloride (1.20 g, 17.5 mmol) and potassium carbonate (2.40 g, 17.5 mmol) in DMSO (6.0 mL). The title compound, a bright yellow solid, was carried forward without further purification (1.25 g, 6.89 mmol, 79%). (ES, *m*/*z*): [M + H]^+^ = 183.0.

#### 2-Methoxy-*N*-(3-methoxy-4-nitrophenyl)-*N*-methylacetamide

A solution of 3-methoxy-*N*-methyl-4-nitroaniline (0.41 g, 2.20 mmol, 1.0 equiv), 2-methoxyacetyl chloride (0.25 mL, 2.70 mmol, 1.2 equiv), *N*,*N*-diisopropylethylamine (0.77 mL, 4.40 mmol, 2.0 equiv) in dichloromethane (4.4 mL, 0.5 M) was stirred at 0 °C for 30 min then warmed to room temperature. After 1 h, additional 2-methoxyacetyl chloride (0.25 mL, 2.70 mmol, 1.2 equiv) was added and the reaction mixture was stirred for 1 h. The reaction mixture was quenched with cold water, extracted with dichloromethane, washed with brine, dried (MgSO_4_), and concentrated under reduced pressure to yield the title compound as a yellow oil (0.59 g, 2.20 mmol, 100%). (ES, *m*/*z*): [M + H]^+^ = 255.1; ^1^H NMR (500 MHz, DMSO-*d*_6_) δ 7.95 (d, *J* = 8.7 Hz, 1H), 7.40 (d, *J* = 2.0 Hz, 1H), 7.12 (dd, *J* = 8.7, 2.1 Hz, 1H), 3.94 (s, 3H), 3.23 (s, 6H).

#### *N*-(4-Amino-3-methoxyphenyl)-2-methoxy-*N*-methylacetamide

General procedure 4 was applied to 2-methoxy-*N*-(3-methoxy-4-nitrophenyl)-*N*-methylacetamide (0.59 g, 2.30 mmol) with palladium on carbon 5 wt % (270 mg, 0.127 mmol) in methanol (9.3.0 mL). The crude material was purified by flash column chromatography eluting with methanol (0–10%) in dichloromethane to yield the title compound as a dark oil (270 mg, 1.19 mmol, 52%). (ES, *m*/*z*): [M + H]^+^ = 225.2.

#### 2-((2-Methoxyphenyl)­amino)-8-phenyl-5-((triisopropylsilyl)­ethynyl)­pyrido [2,3-*d*]­pyrimidin-7­(8*H*)-one

General procedure 1 was applied to 2-(methylsulfonyl)-8-phenyl-5-((triisopropylsilyl)­ethynyl)­pyrido­[2,3-*d*]­pyrimidin-7­(8*H*)-one (350 mg, 0.72 mmol), *N*-(4-amino-3-methoxyphenyl)-2-methoxy-*N*-methylacetamide (163 mg, 0.72 mmol), trifluoroacetic acid (82.0 mg, 0.72 mmol) and 2-butanol (2.7 mL). The crude material was purified by flash column chromatography eluting with methanol (0–10%) in dichloromethane to yield the title compound as a red oil (200 mg, 0.32 mmol, 44%). (ES, *m*/*z*): [M + H]^+^ = 626.5.

#### *N*-(4-((5-Ethynyl-7-oxo-8-phenyl-7,8-dihydropyrido­[2,3-*d*]­pyrimidin-2-yl)­amino)-3-methoxyphenyl)-2-methoxy-*N*-methylacetamide (10)

General procedure 2 was applied to 2-((2-methoxyphenyl)­amino)-8-phenyl-5-((triisopropylsilyl)­ethynyl)­pyrido [2,3-*d*]­pyrimidin-7­(8*H*)-one (140 mg, 0.22 mmol) with potassium fluoride (65.0 mg, 1.10 mmol) in methanol (7.7 mL) and water (0.73.0 mL). The crude material was purified by flash column chromatography eluting with methanol (0–10%) in chloroform. The material was further purified by reverse phase HPLC eluting with acetonitrile (5–95%) in water to yield the title compound as a red solid (10.0 mg, 21.3 μmol, 10%). (ES, *m*/*z*): [M + H]^+^ = 470.3; ^1^H NMR (500 MHz, DMSO) δ 8.83 (s, 1H), 8.56 (s, 1H), 7.58–7.54 (m, 3H), 7.33 (d, *J* = 7.5 Hz, 3H), 6.96 (s, 1H), 6.70 (s, 1H), 6.38 (s, 1H), 5.12 (s, 1H), 3.81 (s, 3H), 3.70 (s, 2H), 3.21 (s, 3H), 3.11 (s, 3H); ^13^C NMR (126 MHz, DMSO) δ 168.7, 162.1, 158.9, 157.38, 156.9, 153.5, 137.8, 136.7, 130.1, 129.7, 129.3, 128.7, 127.3, 122.4, 118.7, 110.3, 106.2, 92.2, 76.9, 70.1, 58.8, 56.6, 37.2, 31.2.

### Compound (**11**)

#### 3-Methoxy-*N*-(3-methoxy-4-nitrophenyl)-*N*-methylpropanamide

A solution of 3-methoxy-*N*-methyl-4-nitroaniline (470 mg, 2.58 mmol) with 3-methoxypropionyl chloride (0.39 mL, 3.57 mmol) and pyridine (0.44 mL, 5.48 mmol) in acetonitrile (5.5 mL) was stirred at room temperature overnight. The crude material was concentrated under reduced pressure, the residue was dissolved in ethyl acetate (20 mL) and washed with saturated aqueous copper­(II) sulfate solution (10 mL). The aqueous layer was extracted with ethyl acetate (2 × 20 mL). The combined organic layers were washed with water (3 × 10 mL), dried (MgSO_4_) and concentrated under reduced pressure to yield the title compound as a yellow solid (620 mg, 2.31 mmol, 90%) which was used without further purification. (ES, *m*/*z*): [M + H]^+^ = 269.2.

#### *N*-(4-Amino-3-methoxyphenyl)-3-methoxy-*N*-methylpropanamide

General procedure 4 was applied to 3-methoxy-*N*-(3-methoxy-4-nitrophenyl)-*N*-methylpropanamide (620 mg, 2.31 mmol) with palladium on carbon 5 wt % (246 mg, 0.231 mmol) in acetic acid (1.0 mL) and ethanol (9.0 mL). The crude material was purified by flash column chromatography eluting with methanol (0–20%) in dichloromethane to yield the title compound as a purple oil (470 mg, 1.97 mmol, 85%). (ES, *m*/*z*): [M + H]^+^ = 239.2.

#### 3-Methoxy-*N*-(3-methoxy-4-((7-oxo-8-phenyl-5-((trimethylsilyl)­ethynyl)-7,8-dihydropyrido­[2,3-*d*]­pyrimidin-2-yl)­amino)­phenyl)-*N*-methylpropanamide

General procedure 1 was applied to 2-(methylsulfonyl)-8-phenyl-5-((trimethylsilyl)­ethynyl)­pyrido­[2,3-*d*]­pyrimidin-7­(8*H*)-one (400 mg, 1.01 mmol) with *N*-(4-amino-3-methoxyphenyl)-3-methoxy-*N*-methylpropanamide (362 mg, 1.52 mmol), trifluoroacetic acid (120 μL, 1.52 mmol) and acetonitrile (10 mL). The crude material was purified by flash column chromatography eluting with methanol (0–10%) in dichloromethane to yield the title compound as a red solid (325 mg, 0.586 mmol, 58%). (ES, *m*/*z*): [M + H]^+^ = 556.5.

#### *N*-(4-((5-Ethynyl-7-oxo-8-phenyl-7,8-dihydropyrido­[2,3-*d*]­pyrimidin-2-yl)­amino)-3-methoxyphenyl)-3-methoxy-*N*-methylpropanamide (11)

A solution of 2-((2-methoxy-4-morpholinophenyl)­amino)-8-phenyl-5-((trimethylsilyl)­ethynyl)­pyrido­[2,3-*d*]­pyrimidin-7­(8*H*)-one (325 mg, 0.586 mmol) and potassium carbonate (81.0 mg, 0.586 mmol) in methanol (6.0 mL) was stirred at room temperature for 1 h. The reaction mixture was quenched with water (30 mL), and the resulting solids collected by vacuum filtration. The crude material was purified by reverse phase flash column chromatography eluting with acetonitrile (5–95%) in water (0.1% formic acid) to yield the title compound as a red solid (82.9 mg, 0.171 mmol, 29%). (ES, *m*/*z*): [M + H]^+^ = 484.4; ^1^H NMR (500 MHz, CDCl_3_) δ 8.84 (s, 1H), 7.97 (s, 1H), 7.58–7.48 (m, 3H), 7.24 (dd, *J* = 6.7, 1.7 Hz, 2H), 6.72 (s, 1H), 6.58 (d, *J* = 2.5 Hz, 1H), 6.21 (s, 1H), 3.79 (s, 3H), 3.64 (s, 1H), 3.56 (t, *J* = 6.5 Hz, 2H), 3.24 (s, 3H), 3.15 (s, 3H), 2.24 (t, *J* = 6.5 Hz, 2H); ^13^C NMR (126 MHz, CDCl_3_) δ 171.1, 162.4, 158.5, 157.5, 156.7, 148.3, 138.1, 136.2, 130.0, 129.8, 128.7, 128.7, 127.6, 123.0, 119.2, 118.6, 109.1, 106.1, 88.3, 68.8, 58.8, 55.9, 37.3, 34.3.

### Compound **12**

#### 2-Chloro-*N*-(3-methoxy-4-nitrophenyl)-*N*-methylacetamide

A solution of 3-methoxy-*N*-methyl-4-nitroaniline (1.42 g, 7.80 mmol, 1.0 equiv) and chloroacetyl chloride (740 μL, 9.36 mmol, 1.2 equiv) in ethyl acetate (16 mL, 0.5 M) was stirred at 70 °C for 1 h, then cooled to room temperature. Upon the addition of 40–60 petroleum ether, the precipitate formed was collected by vacuum filtration to yield the title compound (1.29 g, 4.99 mmol, 64%). (ES, *m*/*z*): [M + H]^+^ = 259.1.

#### 2-(Dimethylamino)-*N*-(3-methoxy-4-nitrophenyl)-*N*-methylacetamide

A solution of 2-chloro-*N*-(3-methoxy-4-nitrophenyl)-*N*-methylacetamide (1.00 g, 3.87 mmol, 1.0 equiv), dimethylamine (13.5 mL, 13.5 mmol, 3.5 equiv) and potassium carbonate (1.87 g, 13.5 mmol, 3.5 equiv) in THF (77 mL, 0.05 M) and water (38 mL, 0.1 M) was stirred at room temperature overnight. The reaction mixture was quenched with saturated aqueous ammonium chloride solution (30 mL) and extracted with dichloromethane (3 × 30 mL). The combined organic layers were dried (MgSO_4_) and concentrated under reduced pressure. The crude material was purified by flash column chromatography eluting with methanol (0–20%) in dichloromethane to yield the title compound (601 mg, 2.25 mmol, 58%). (ES, *m*/*z*): [M + H]^+^ = 268.2

#### *N*-(4-Amino-3-methoxyphenyl)-2-(dimethylamino)-*N*-methylacetamide

General procedure 4 was applied to 2-(dimethylamino)-*N*-(3-methoxy-4-nitrophenyl)-*N*-methylacetamide (600 mg, 2.24 mmol) with palladium on carbon 5 wt % (239 mg, 0.224 mmol) in ethanol (8.1.0 mL) and acetic acid (0.90 mL). The crude material was purified by flash column chromatography eluting with methanol (0–20%) in dichloromethane to yield the title compound as a purple oil (587 mg, 2.83 mmol, 100%).

#### 2-(Dimethylamino)-*N*-(3-methoxy-4-((7-oxo-8-phenyl-5-((triisopropylsilyl)­ethynyl)-7,8-dihydropyrido­[2,3-*d*]­pyrimidin-2-yl)­amino)­phenyl)-*N*-methylacetamide

General procedure 1 was applied to 2-(methylsulfonyl)-8-phenyl-5-((triisopropylsilyl)­ethynyl)­pyrido­[2,3-*d*]­pyrimidin-7­(8*H*)-one (500 mg, 1.04 mmol) with *N*-(4-amino-3-methoxyphenyl)-2-(dimethylamino)-*N*-methylacetamide (226 mg, 1.09 mmol) and trifluoroacetic acid (80.0 μL, 1.09 mmol) in acetonitrile (10 mL). The crude material was purified by flash column chromatography eluting with methanol (0–20%) in dichloromethane to yield the title compound as an orange solid (178 mg, 0.278 mmol, 27%). (ES, *m*/*z*): [M + H]^+^ = 639.5

#### 2-(Dimethylamino)-*N*-(4-((5-ethynyl-7-oxo-8-phenyl-7,8-dihydropyrido­[2,3-*d*]­pyrimidin-2-yl)­amino)-3-methoxyphenyl)-*N*-methylacetamide (12)

General procedure 2 was applied to 2-(dimethylamino)-*N*-(3-methoxy-4-((7-oxo-8-phenyl-5-((triisopropylsilyl)­ethynyl)-7,8-dihydropyrido­[2,3-*d*]­pyrimidin-2-yl)­amino)­phenyl)-*N*-methylacetamide (50.0 mg, 78.3 μmol) with potassium fluoride (91 mg, 1.56 mmol) in DMF (1.0 mL). The crude material was purified by flash column chromatography eluting with methanol (0–20%) in dichloromethane to yield the title compound as a red solid (17.4 mg, 38.5 μmol, 49%). (ES, *m*/*z*): [M + H]^+^ = 483.4; ^1^H NMR (500 MHz, CDCl_3_) δ 8.85 (s, 1H), 7.98 (s, 1H), 7.60–7.45 (m, 3H), 7.42 (d, *J* = 13.6 Hz, 1H), 7.28–7.22 (m, 2H), 6.72 (s, 1H), 6.57 (d, *J* = 2.2 Hz, 1H), 6.19 (s, 1H), 3.81 (s, 3H), 3.65 (s, 1H), 3.14 (s, 3H), 2.83 (s, 2H), 2.26 (s, 6H); ^13^C NMR (126 MHz, CDCl_3_) δ 169.30, 162.41, 158.49, 157.55, 156.74, 148.35, 137.29, 136.21, 129.97, 129.78, 128.72, 128.68, 127.86, 123.16, 119.16, 118.59, 108.75, 106.18, 88.14, 59.79, 56.02, 45.39, 37.54.

## Supplementary Material





## Notes

Supplementary figures, additional experimental details and analytical spectra (PDF)
Molecular formular strings (CSV)

## Abbreviations Used

EGF: epidermal growth factor
EGFR: epidermal growth factor receptor
GST: glutathione
mLTC-EGFR: triply mutated L858R, T790M, C797S EGFR
mLT-EGFR: doubly mutated L858R, T790M EGFR
NSCLC: nonsmall cell lung cancer
TR-FRET: time-resolved Förster-resonance energy transfer
WT-EGFR: wild-type EGFR

## References

[1] Konduri K., Gallant J. N., Chae Y. K., Giles F. J., Gitlitz B. J., Gowen K., Ichihara E., Owonikoko T. K., Peddareddigari V., Ramalingam S. S., Reddy S. K., Eaby-Sandy B., Vavalà T., Whiteley A., Chen H., Yan Y., Sheehan J. H., Meiler J., Morosini D., Ross J. S., Stephens P. J., Miller V. A., Ali S. M., Lovly C. M. (2016). EGFR Fusions
as Novel Therapeutic Targets in Lung Cancer. Cancer Discovery.

[2] Mitsudomi T., Morita S., Yatabe Y., Negoro S., Okamoto I., Tsurutani J., Seto T., Satouchi M., Tada H., Hirashima T., Asami K., Katakami N., Takada M., Yoshioka H., Shibata K., Kudoh S., Shimizu E., Saito H., Toyooka S., Nakagawa K., Fukuoka M. (2010). Articles Gefi
Tinib versus Cisplatin plus Docetaxel in Patients with Non-Small-Cell
Lung Cancer Harbouring Mutations of the Epidermal Growth Factor Receptor
(WJTOG3405): An Open Label, Randomised Phase 3 Trial. Lancet Oncol..

[3] Zhou C., Hu C., Hospital S. X., Zhou C., Wu Y.-L., Chen G., Feng J., Liu X.-Q., Wang C., Zhang S., Wang J., Zhou S., Ren S., Lu S., Zhang L., Hu C., Luo Y., Chen L., Ye M., Huang J., Zhi X., Zhang Y., Xiu Q., Ma J., You C. (2011). Erlotinib
versus Chemotherapy as Fi Rst-Line Treatment
for Patients with Advanced EGFR Mutation-Positive Non-Small-Cell Lung
Cancer (OPTIMAL, CTONG-0802): A Multicentre, Open-Label, Randomised,
Phase 3 Study. Lancet Oncol..

[4] Yun C.-H., Mengwasser K. E., Toms A. V., Woo M. S., Greulich H., Wong K.-K., Meyerson M., Eck M. J. (2008). The T790M Mutation
in EGFR Kinase Causes Drug Resistance by Increasing the Affinity for
ATP. Proc. Natl. Acad. Sci. U.S.A..

[5] Suda K., Onozato R., Yatabe Y., Mitsudomi T. (2009). EGFR T790M
Mutation: A Double Role in Lung Cancer Cell Survival?. J. Thorac. Oncol..

[6] Yun C.-H., Mengwasser K. E., Toms A. V., Woo M. S., Greulich H., Wong K.-K., Meyerson M., Eck M. J. (2008). The T790M Mutation
in EGFR Kinase causes Drug Resistance by Increasing the Affinity for
ATP. Proc. Natl. Acad. Sci. U.S.A..

[7] Park K., Tan E. H., O’Byrne K., Zhang L., Boyer M., Mok T., Hirsh V., Yang J. C. H., Lee K. H., Lu S., Shi Y., Kim S. W., Laskin J., Kim D. W., Arvis C. D., Kölbeck K., Laurie S. A., Tsai C. M., Shahidi M., Kim M., Massey D., Zazulina V., Paz-Ares L. (2016). Afatinib versus Gefitinib
as First-Line Treatment of Patients with EGFR Mutation-Positive Non-Small-Cell
Lung Cancer (LUX-Lung 7): A Phase 2B, Open-Label, Randomised Controlled
Trial. Lancet Oncol..

[8] Finlay M. R. V., Anderton M., Ashton S., Ballard P., Bethel P. A., Box M. R., Bradbury R. H., Brown S. J., Butterworth S., Campbell A., Chorley C., Colclough N., Cross D. A. E., Currie G. S., Grist M., Hassall L., Hill G. B., James D., James M., Kemmitt P., Klinowska T., Lamont G., Lamont S. G., Martin N., McFarland H. L., Mellor M. J., Orme J. P., Perkins D., Perkins P., Richmond G., Smith P., Ward R. A., Waring M. J., Whittaker D., Wells S., Wrigley G. L. (2014). Discovery
of a Potent and Selective EGFR Inhibitor (AZD9291) of Both Sensitizing
and T790M Resistance Mutations That Spares the Wild Type Form of the
Receptor. J. Med. Chem..

[9] Jänne P. A., Yang J. C.-H., Kim D.-W., Planchard D., Ohe Y., Ramalingam S. S., Ahn M.-J., Kim S.-W., Su W.-C., Horn L., Haggstrom D., Felip E., Kim J.-H., Frewer P., Cantarini M., Brown K. H., Dickinson P. A., Ghiorghiu S., Ranson M. (2015). AZD9291 in EGFR Inhibitor–Resistant
Non–Small-Cell
Lung Cancer. N. Engl. J. Med..

[10] Soria J.-C., Ohe Y., Vansteenkiste J., Reungwetwattana T., Chewaskulyong B., Lee K. H., Dechaphunkul A., Imamura F., Nogami N., Kurata T., Okamoto I., Zhou C., Cho B. C., Cheng Y., Cho E. K., Voon P. J., Planchard D., Su W.-C., Gray J. E., Lee S.-M., Hodge R., Marotti M., Rukazenkov Y., Ramalingam S. S. (2018). Osimertinib in Untreated EGFR -Mutated Advanced Non–Small-Cell
Lung Cancer. N. Engl. J. Med..

[11] Finlay M. R. V., Anderton M., Ashton S., Ballard P., Bethel P. A., Box M. R., Bradbury R. H., Brown S. J., Butterworth S., Campbell A., Chorley C., Colclough N., Cross D. A. E., Currie G. S., Grist M., Hassall L., Hill G. B., James D., James M., Kemmitt P., Klinowska T., Lamont G., Lamont S. G., Martin N., McFarland H. L., Mellor M. J., Orme J. P., Perkins D., Perkins P., Richmond G., Smith P., Ward R. A., Waring M. J., Whittaker D., Wells S., Wrigley G. L. (2014). Discovery
of a Potent and Selective EGFR Inhibitor (AZD9291) of Both Sensitizing
and T790M Resistance Mutations That Spares the Wild Type Form of the
Receptor. J. Med. Chem..

[12] Shah D., Shah D., Ndandji S., Kar S. (2025). Lazertinib: A Novel
EGFR-TKI Therapy for Non-Small Cell Lung Cancer. Expert Opin. Drug Metab. Toxicol..

[13] Mok T. S., Wu Y.-L., Ahn M.-J., Garassino M. C., Kim H. R., Ramalingam S. S., Shepherd F. A., He Y., Akamatsu H., Theelen W. S. M. E., Lee C. K., Sebastian M., Templeton A., Mann H., Marotti M., Ghiorghiu S., Papadimitrakopoulou V. A. (2017). Osimertinib or Platinum–Pemetrexed
in EGFR T790M–Positive Lung Cancer. N.
Engl. J. Med..

[14] Soria J.-C., Ohe Y., Vansteenkiste J., Reungwetwattana T., Chewaskulyong B., Lee K. H., Dechaphunkul A., Imamura F., Nogami N., Kurata T., Okamoto I., Zhou C., Cho B. C., Cheng Y., Cho E. K., Voon P. J., Planchard D., Su W.-C., Gray J. E., Lee S.-M., Hodge R., Marotti M., Rukazenkov Y., Ramalingam S. S. (2018). Osimertinib in Untreated EGFR -Mutated Advanced non–Small-Cell
Lung Cancer. N. Engl. J. Med..

[15] Ramalingam S. S., Vansteenkiste J., Planchard D., Cho B. C., Gray J. E., Ohe Y., Zhou C., Reungwetwattana T., Cheng Y., Chewaskulyong B., Shah R., Cobo M., Lee K. H., Cheema P., Tiseo M., John T., Lin M.-C., Imamura F., Kurata T., Todd A., Hodge R., Saggese M., Rukazenkov Y., Soria J.-C. (2020). Overall Survival with Osimertinib
in Untreated, EGFR -Mutated Advanced NSCLC. N. Engl. J. Med..

[16] Thress K. S., Paweletz C. P., Felip E., Cho B. C., Stetson D., Dougherty B., Lai Z., Markovets A., Vivancos A., Kuang Y., Ercan D., Matthews S. E., Cantarini M., Barrett J. C., Jänne P. A., Oxnard G. R. (2015). Acquired EGFR C797S Mutation Mediates Resistance to
AZD9291 in Non–Small Cell Lung Cancer Harboring EGFR T790M. Nat. Med..

[17] Grabe T., Lategahn J., Rauh D. (2018). C797S Resistance: The Undruggable
EGFR Mutation in Non-Small Cell Lung Cancer?. ACS Med. Chem. Lett..

[18] Leonetti A., Sharma S., Minari R., Perego P., Giovannetti E., Tiseo M. (2019). Resistance Mechanisms
to Osimertinib in EGFR-Mutated Non-Small Cell
Lung Cancer. Br. J. Cancer.

[19] Li Z., Lu W., Beyett T. S., Ficarro S. B., Jiang J., Tse J., Kim A. Y. J., Marto J. A., Che J., Jänne P. A., Eck M. J., Zhang T., Gray N. S. (2024). ZNL0325, a Pyrazolopyrimidine-Based
Covalent Probe, Demonstrates an Alternative Binding Mode for Kinases. J. Med. Chem..

[20] Kuki N., Walmsley D. L., Kanai K., Takechi S., Yoshida M., Murakami R., Takano K., Tominaga Y., Takahashi M., Ito S., Nakao N., Angove H., Baker L. M., Carter E., Dokurno P., Le Strat L., Macias A. T., Molyneaux C. A., Murray J. B., Surgenor A. E., Hamada T., Hubbard R. E. (2023). A Covalent
Fragment-Based Strategy Targeting a Novel Cysteine to Inhibit Activity
of Mutant EGFR Kinase. RSC Med. Chem..

[21] Li Z., Jiang J., Ficarro S. B., Beyett T. S., To C., Tavares I., Zhu Y., Li J., Eck M. J., Jänne P. A., Marto J. A., Zhang T., Che J., Gray N. S. (2024). Molecular Bidents with Two Electrophilic Warheads as
a New Pharmacological Modality. ACS Cent. Sci..

[22] Niggenaber J., Heyden L., Grabe T., Müller M. P., Lategahn J., Rauh D. (2020). Complex Crystal Structures of EGFR
with Third-Generation Kinase Inhibitors and Simultaneously Bound Allosteric
Ligands. ACS Med. Chem. Lett..

[23] Wang A., Yan X.-E., Wu H., Wang W., Hu C., Chen C., Zhao Z., Zhao P., Li X., Wang L., Wang B., Ye Z., Wang J., Wang C., Zhang W., Gray N. S., Weisberg E. L., Chen L., Liu J., Yun C.-H., Liu Q. (2016). Ibrutinib
Targets Mutant-EGFR Kinase with a Distinct Binding Conformation. Oncotarget.

[24] Yu L., Huang M., Xu T., Tong L., Yan Xe., Zhang Z., Xu Y., Yun C., Xie H., Ding K., Lu X. (2017). A Structure-Guided
Optimization of
Pyrido­[2,3-d]­Pyrimidin-7-Ones as Selective Inhibitors of EGFRL858R/T790Mmutant
with Improved Pharmacokinetic Properties. Eur.
J. Med. Chem..

[25] Morese P. A., Ahmad A., Martin M. P., Noble R. A., Pintar S., Wang L. Z., Xu S., Lister A., Ward R. A., Bronowska A. K., Noble M. E. M., Stewart H. L., Waring M. J. (2025). Factors
Affecting Irreversible Inhibition of EGFR and Influence of Chirality
on Covalent Binding. Commun. Chem..

[26] Flanagan M. E., Abramite J. A., Anderson D. P., Aulabaugh A., Dahal U. P., Gilbert A. M., Li C., Montgomery J., Oppenheimer S. R., Ryder T., Schuff B. P., Uccello D. P., Walker G. S., Wu Y., Brown M. F., Chen J. M., Hayward M. M., Noe M. C., Obach R. S., Philippe L., Shanmugasundaram V., Shapiro M. J., Starr J., Stroh J., Che Y. (2014). Chemical and Computational Methods for the Characterization of Covalent
Reactive Groups for the Prospective Design of Irreversible Inhibitors. J. Med. Chem..

