# Supplementary material for: Covalent Alkynylpyridopyrimidinones Targeting Cysteine 775 of the Epidermal Growth Factor Receptor Overcome Resistance to Current Therapies
Source: J Med Chem. 2025 Dec 23;69(1):533–52. doi: 10.1021/acs.jmedchem.5c02924 (PMC12794186; doi:10.1021/acs.jmedchem.5c02924)
Supplement: Supplementary file 1 [file jm5c02924_si_001.pdf]

Supporting Information

Covalent alkynylpyridopyrimidinones targeting cysteine 775 of the epidermal growth factor receptor overcome resistance to current therapies

Hannah L. Stewart,[a] Cinzia Bordoni,[a] Claire E. Jennings,[b] Islam Al-Khawaldeh,[a] Mathew P. Martin,[b] Richard A. Noble,[b] Nicole Phillips,[b] Sara Pintar,[b] Lisa Prendergast,[b] Huw D. Thomas,[b] Lan-Z. Wang,[b] Jessica E. Watt,[b] Anita Wittner,[b] Agnieszka K. Bronowska,[c] Céline Cano,[a] Martin E. M. Noble,[b] Stephen R. Wedge[b] and Michael J. Waring[a*]

[a]Cancer Research Horizons Newcastle Drug Discovery Group, Chemistry, School of Natural and Environmental Sciences, Bedson Building, Newcastle University, Newcastle upon Tyne, NE1 7RU, UK.
[b]Cancer Research Horizons Newcastle Drug Discovery G, Translational and Clinical Research Institute, Paul O'Gorman Building, Newcastle University, Newcastle upon Tyne, NE2 4HH, UK
[c] Chemistry, School of Natural and Environmental Sciences, Bedson Building, Newcastle University, Newcastle upon Tyne, NE1 7RU, UK.
Corresponding author email: mike.waring@ncl.ac.uk

Contents

Supplementary Figures and Tables 2
Experimental Details 19
Protein Mass Spectrometry 34
HPLC Spectra 44
NMR Spectra 68
References 92

Supplementary Figures and Tables

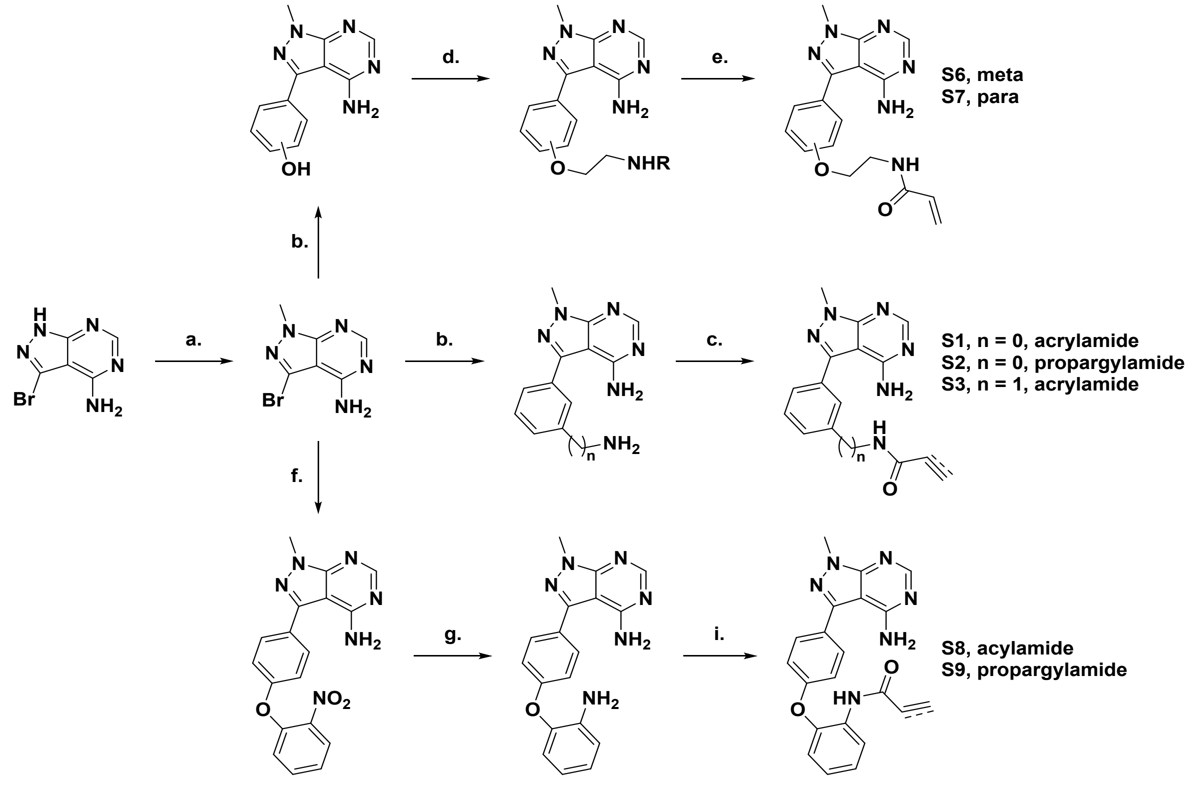

Scheme S1: a. MeOH, PPh_3, DIAD, rt, 2h; b. $RB(OH)_2$, $Pd(dppf)Cl_2$, K_2CO_3, dioxane, H_2O, 100 °C, 2h, 63-89%; c. acroloyl chloride, DIPEA, DCM, 0 °C, rt, 2h, 2-12% or propoiolic acid, HATU, DIPEA, DCM, rt, 2h, 12%; d. $BocHN(CH_2)_2Br$, K_2CO_3, acetone, 60 °C, 2h, 81%; e. i) TFA, DCM, rt, 1h, ii) acroloyl chloride, TEA, DCM , rt, 2h, 11%; f. RBPin, $Pd(dppf)Cl_2$, KOAc, dioxane, H_2O, 44%; g. Zn, NH_4Cl, THF, H_2O, 0 °C-rt, 2h, 73%.

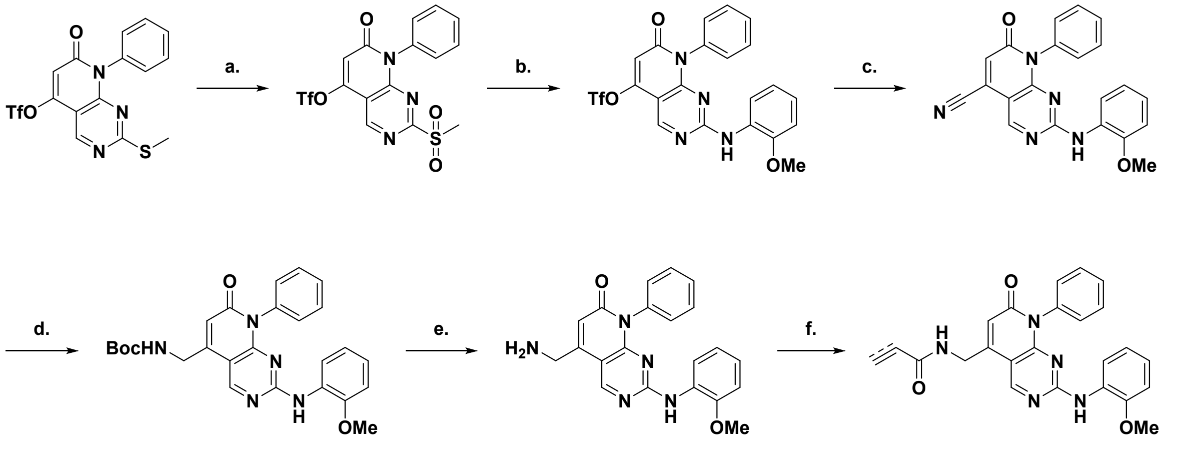

Scheme S2: a. mCPBA, DCM, rt, 4.5h, 74%; b. o-anisidine, TFA, nBuOH, 110 °C, 12h, 63%; c. $Zn(CN)_2$, $Zn(OAc)_2$, $Pd_2(dba)_3$, dppf, DMF, 100 °C, 1h, 40%; d. Pd/C, H_2, Boc_2O, MeOH, rt, 1h, 78%; e. HCl, DCM, rt, 1h, 5%; acroloyl chloride, TEA, DCM, 0 °C, 1h, 10% or i) 3-TMS-propiolic acid, DCC, DCM, rt, 1h, 88%, ii) KF, MeOH, rt, 1h, 16%.

Table S1: Exploration of acrylamide and propargylamide warheads around scaffolds A-C.

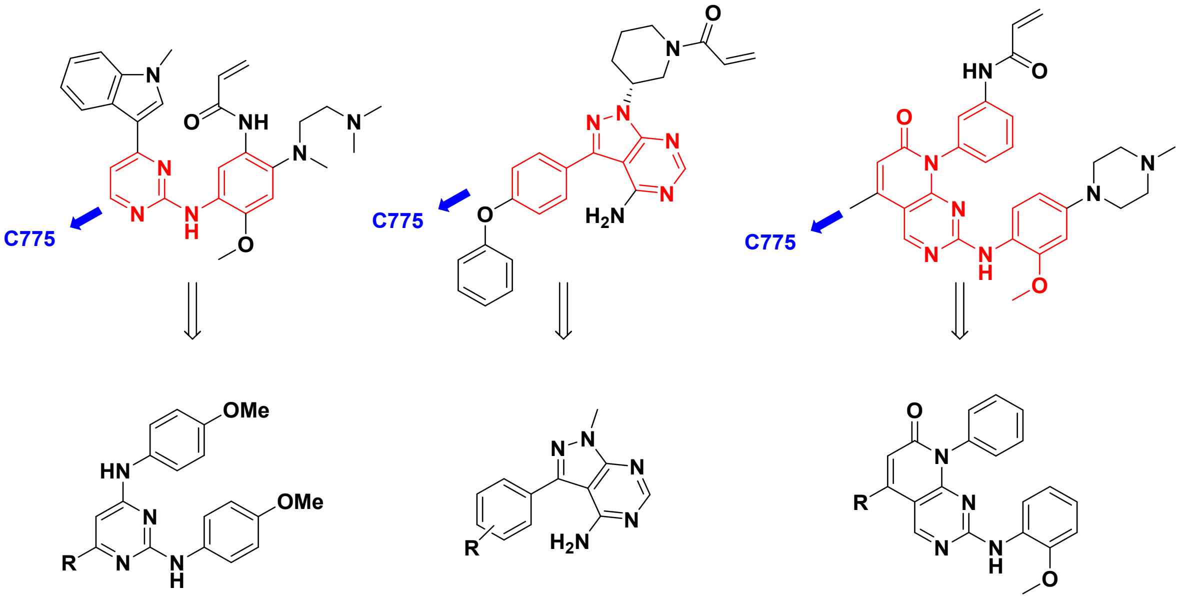

A B C

	R	Meta/Para	Core	mLTC IC_{50} /µM (pIC_{50} ± std dev)	Protein adduct formation
**S1**	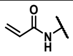	Meta	B	>100 (<4)	1
**S2**	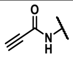	Meta	B	20 (4.7 ± 0.81)	1-3
**S3**	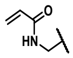	Meta	B	>100 (<4)	0
**S4**		n/a	C	>100 (<4)	0
**S5**	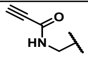	n/a	C	>100 (<4)	1-2
**S6**	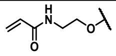	Meta	B	>100 (<4)	n.d.
**S7**	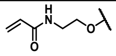	Para	B	>100 (<4)	0
**S8**	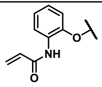	Meta	B	>100 (<4)	0-1
**S9**	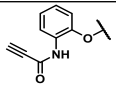	Meta	B	23 (4.6 ± 0.14)	1-4

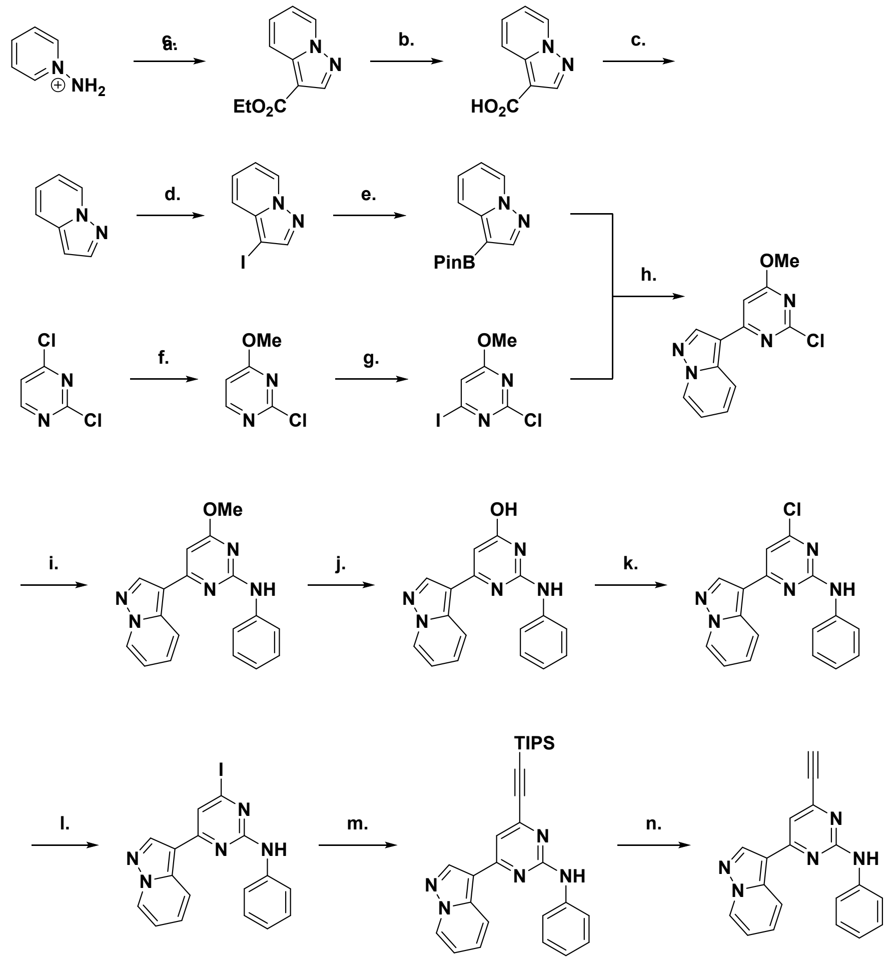

Scheme S3: a. ethyl propiolate, K_2CO_3, MeCN, rt, 12, 100%; b. NaOH, EtOH, reflux, 1h, 100%; c. H_2SO_4, reflux, 2h, 30%; d. NIS, MeCN, rt, 1h, 85%; e. trubo Grignard, ^{i}OPrBPin, THF, -10 °C, 1h, 95%; f. NaOMe, MeOH, rt, 12h, 82%; g. i) ^{n}BuLi, 2,2,6,6-tetramethylpiperidine, THF, -78 °C, 1h, ii) I_2, THF, -78 °C, 2.5h, 90%; h. $Pd(PPh_3)_4$, Cs_2CO_3, dioxane, 100 °C, 4h, 86%; i. aniline, pTsOH, IPA, 55 °C, 12h, 49%; j. HBr (in AcOH), reflux, 1h, 73%; k. $POCl_3$, 80 °C, 2h, 55%; l. i) Tf_2O, 2,6-lutidine, MeCN, rt, 30 mins, ii) NaI, TfOH, rt, 1h, 82%; m. TIPSCCH, $Pd(PPh_3)_2Cl_2$, CuI, DIPEA, DMF, 55 °C, 2 days, 95%; n. TBAF, THF, rt, 15 mins.

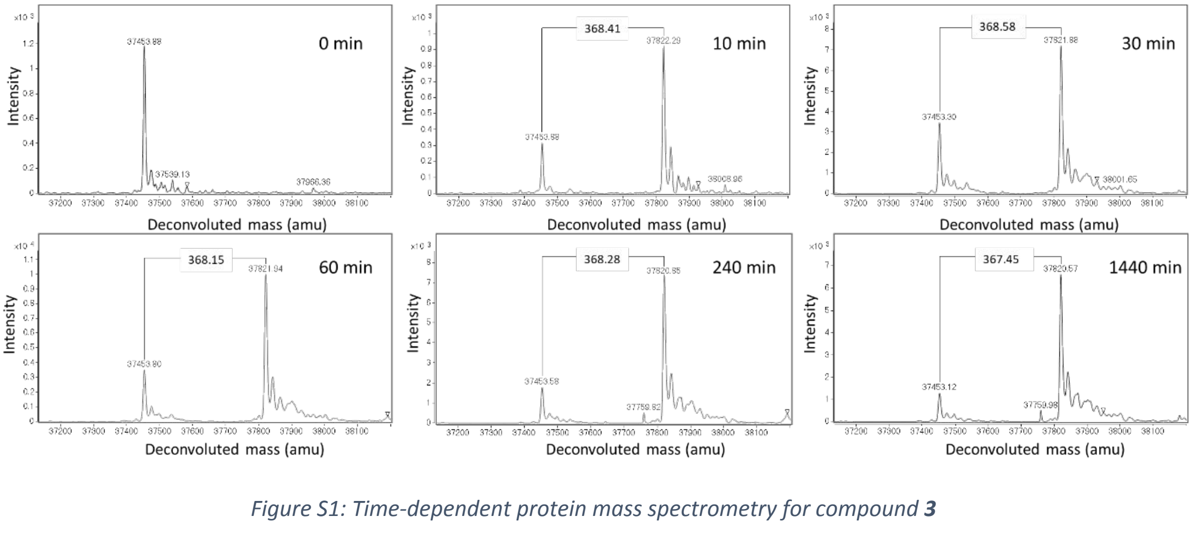

*Figure S1: Time-dependent protein mass spectrometry for compound **3***

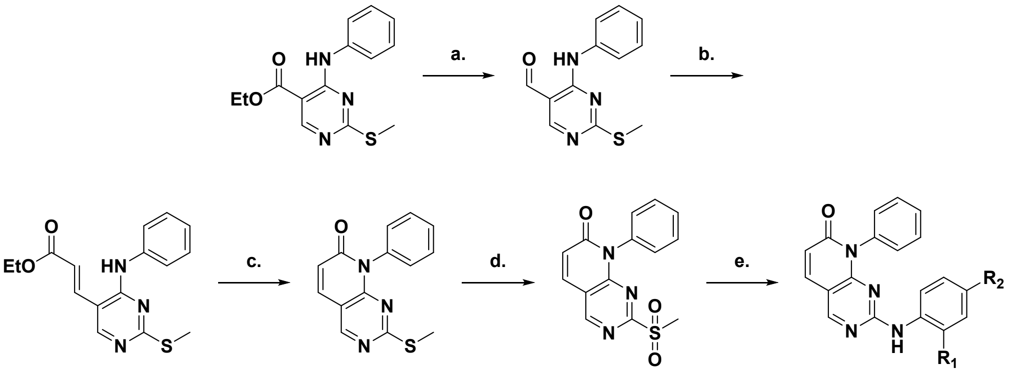

S10, R_1 = OMe, R_2 = H
S13, R_1 = H, R_2 = $NMeCH_2CH_2NMe_2$

Scheme S4: a. i) $LiAlH_4$, THF, 0 °C-rt, 12h, ii) MnO_2, DCM, rt, 12h, 53%; b. $EtO_2CCHPPh_3$, THF, reflux, 2h, 79%; c. DBU, DIPEA, 120 °C, 12h, 45%; c. mCPBA, DCM, rt, 2h, 88%; d. aniline, MeCN, 85 °C, 12h, 42-64%.

Table S2: TR-FRET analysis of non-covalent analogues S10 and 12

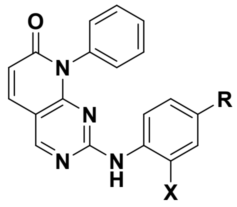

	X	R	mLTC-EGFR IC_{50} (µM) (pIC_{50} ± std dev)	WT-EGFR IC_{50} (µM) (pIC_{50} ± std dev)
**S10**	OMe	H	7.8 (5.1 ± 0.39)	15 (4.8 ± 0.071)
**S13**	H	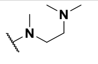	0.63 (6.2 ± 0.14)	7.2 (5.1 ± 0.40)

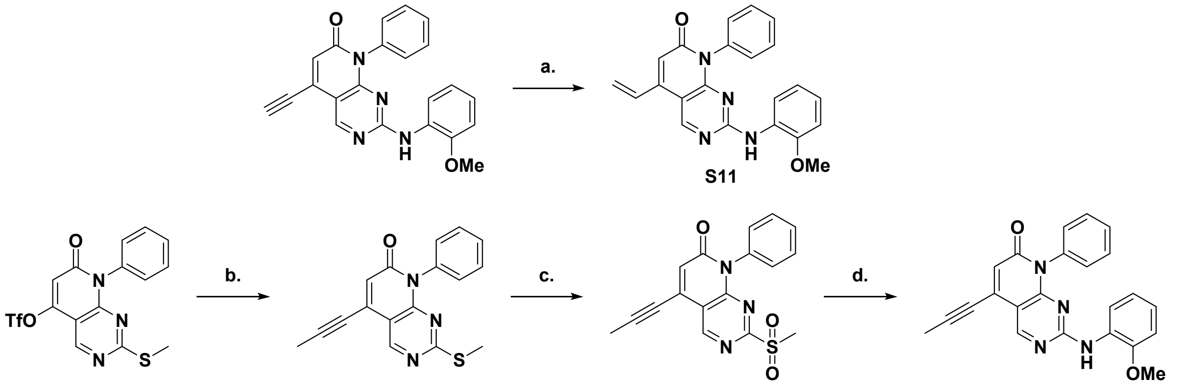

S11

S12

Scheme S5: a. Lindlar Pd, H_2, MeOH, rt, 1h, 25%; b. $MeCCSnBu_3$, $Pd(PPh_3)_4$, dioxane, 100 °C, 12h, 96%; c. mCPBA, DCM, rt, 1h, 82%; d. o-anisidine, TFA, nBuOH, 110 °C, 12h, 14%.

Table S3: TR-FRET analysis of S11 and S12

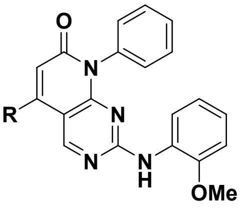

	R	mLTC-EGFR IC_{50} (µM) (pIC_{50} ± std dev)	WT-EGFR IC_{50} (µM) (pIC_{50} ± std dev)
**S11**	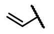	11 (5.0 ± 0.078)	>100 (<4)
**S12**	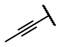	>100 (<4)	>100 (<4)

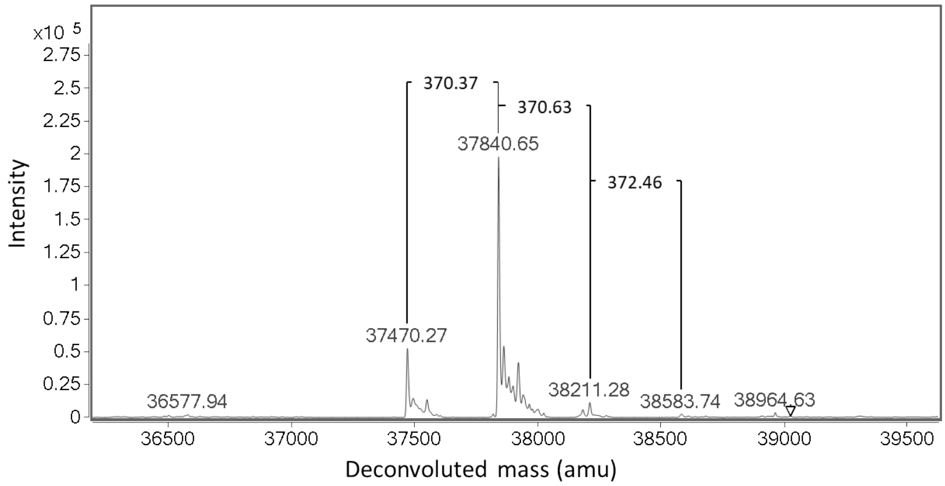

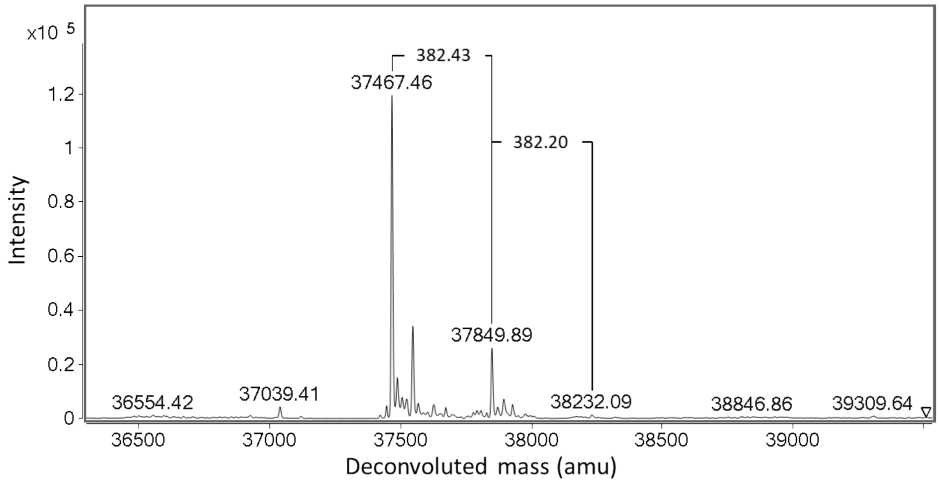

*Figure S2: Protein mass spectrometry for a) **S11** showing a significant amount of single protein; b) **S12** showing mostly unmodulated protein.*

*Table S4: EGFR selectivity of **7** measured at 1.0 µM*

Protein	Percent Control
**EGFR**	31
**EGFR(L858R)**	5
**EGFR(E746-A750del)**	14
**EGFR(T790M)**	40
**EGFR(L858R/T790M)**	2.2

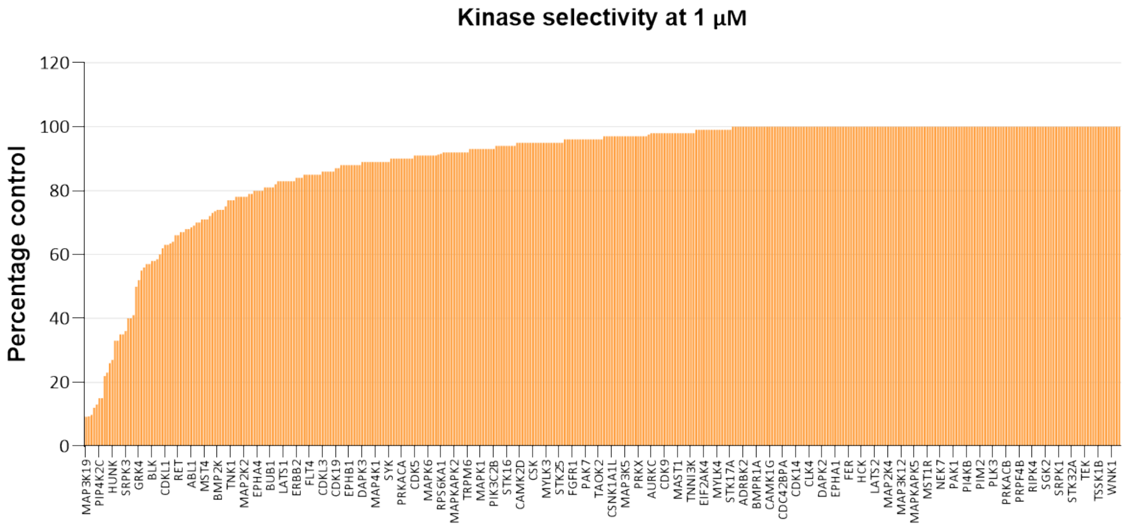

Figure S3: Kinase selectivity panel (456 kinases) measured at 1.0 μM shows only 10 kinases with >75% inhibition. Some kinases labelled for reference, the full dataset is presented in Table S5.

*Table S5: Selectivity of **7** against a panel of 456 measured at 1.0 μM*

Protein	Mean (Percent Control)
**MAP3K19**	9.2
**MAP3K14**	9.3
**NTRK1**	9.8
**MAP3K7**	12
**MAP2K5**	13
**PIP4K2C**	15
**FLT3**	15
**AURKB**	22
**CSF1R**	23
**NEK3**	26
**HUNK**	27
**GSK3B**	33
**NTRK2**	33
**GSK3A**	35
**STK33**	35
**SRPK3**	36
**MINK1**	40
**PIKFYVE**	40
**TNIK**	41
**PDGFRB**	50
**GRK4**	52
**TBK1**	55
**MAP4K4**	56
**LCK**	57
**pknB**	57
**BLK**	58
**HIPK1**	58
**TYK2**	58.5

**PIP5K1A**	60
**MYO3B**	62
**CDKL1**	63
**GAK**	63
**JAK1**	63.5
**AXL**	64
**ERBB3**	66
**RET**	66
**DDR1**	67
**DDR2**	67
**INSR**	68
**STK17B**	68
**ABL1**	68.5
**NTRK3**	69
**ICK**	70
**PDGFRA**	70
**CSNK1E**	71
**MST4**	71
**STK24**	71
**MAPK12**	72
**DCLK1**	73
**BRAF**	73.5
**BMP2K**	74
**MAL13P1.279**	74
**STK38**	74
**NEK10**	75
**CSNK1A1**	77
**TNK1**	77
**TTK**	77
**CDKL2**	78
**DYRK1B**	78
**GRK1**	78
**MAP2K2**	78
**TLK1**	78
**IRAK4**	79
**MAP4K3**	79
**DAPK1**	80
**EPHA4**	80
**KIT**	80
**LIMK1**	80
**ACVR2A**	81
**AURKA**	81
**BUB1**	81
**PIP5K1C**	81
**STK36**	82
**ACVR2B**	83
**CLK1**	83
**LATS1**	83
**MAP2K3**	83
**MAP4K5**	83

**PKMYT1**	83
**WNK2**	83
**ERBB2**	84
**GSG2**	84
**MERTK**	84
**AAK1**	85
**EPHA6**	85
**FLT4**	85
**KIAA0999**	85
**MAP3K1**	85
**MUSK**	85
**PIK3C3**	85
**CDKL3**	86
**CSNK1G3**	86
**IRAK1**	86
**MAP3K13**	86
**RPS6KA2**	86
**CDK19**	87
**PRKCE**	87
**CDPK1**	88
**CSNK1G2**	88
**DMPK**	88
**EPHB1**	88
**ERN1**	88
**KDR**	88
**RPS6KB1**	88
**YES1**	88
**DAPK3**	89
**EPHA5**	89
**EPHB3**	89
**ITK**	89
**LIMK2**	89
**MAP4K1**	89
**MAPK9**	89
**NLK**	89
**NUAK1**	89
**PIP4K2B**	89
**SYK**	89
**DYRK1A**	90
**MAP3K9**	90
**MAP3K10**	90
**MTOR**	90
**PRKACA**	90
**RIPK1**	90
**RPS6KA6**	90
**STK11**	90
**ULK1**	90
**CDK5**	91
**DYRK2**	91
**EPHB2**	91

**FGR**	91
**MAPK3**	91
**MAPK6**	91
**MAPK8**	91
**SgK110**	91
**WNK4**	91
**PIK3CA**	91.3
**RPS6KA1**	91.5
**DCLK2**	92
**EPHB4**	92
**FES**	92
**JAK2**	92
**MAPKAPK2**	92
**MET**	92
**PRKCI**	92
**RPS6KA3**	92
**SIK1**	92
**TRPM6**	92
**ABL2**	93
**CAMKK1**	93
**CDC42BPB**	93
**MAP3K11**	93
**MAPK1**	93
**MAPK10**	93
**MARK2**	93
**NEK4**	93
**NEK5**	93
**PIK3C2B**	93
**CDC2L2**	94
**EPHA8**	94
**ERBB4**	94
**MATK**	94
**STK16**	94
**STK35**	94
**STK38L**	94
**TIE1**	94
**AKT3**	95
**CAMK2D**	95
**CDC42BPG**	95
**CDK3**	95
**CDK18**	95
**CLK3**	95
**CSK**	95
**HIPK4**	95
**INSRR**	95
**IRAK3**	95
**MARK1**	95
**MYLK3**	95
**NEK6**	95
**PHKG1**	95

**PIM3**	95
**SGK1**	95
**STK25**	95
**WEE1**	95
**CABC1**	96
**CAMKK2**	96
**CHUK**	96
**FGFR1**	96
**FGFR2**	96
**MAP3K3**	96
**MAPK15**	96
**PAK3**	96
**PAK7**	96
**PTK2**	96
**RIPK2**	96
**SGK3**	96
**STK32C**	96
**TAOK2**	96
**WEE2**	96
**ACVR1**	97
**CDK15**	97
**CHEK1**	97
**CSNK1A1L**	97
**FLT1**	97
**IKBKB**	97
**JAK3**	97
**LYN**	97
**MAP3K5**	97
**PIK3CG**	97
**PRKCH**	97
**PRKCQ**	97
**PRKD3**	97
**PRKX**	97
**TEC**	97
**TNK2**	97
**ULK3**	97
**FGFR3**	97.5
**AURKC**	98
**BMX**	98
**BRSK2**	98
**CAMK1**	98
**CAMK2G**	98
**CDK9**	98
**CDKL5**	98
**CIT**	98
**MARK3**	98
**MARK4**	98
**MAST1**	98
**PHKG2**	98
**RIOK1**	98

**RIOK3**	98
**RPS6KA5**	98
**TNNI3K**	98
**ULK2**	98
**BRSK1**	99
**CDK4**	99
**CDK11B**	99
**EIF2AK4**	99
**MAP3K6**	99
**MAPK4**	99
**MAPK13**	99
**MAPK14**	99
**MYLK4**	99
**MYO3A**	99
**PRKG1**	99
**ROCK1**	99
**SRMS**	99
**STK17A**	99
**ACVR1B**	100
**ACVRL1**	100
**ADCK4**	100
**ADRBK1**	100
**ADRBK2**	100
**AKT1**	100
**AKT2**	100
**ALK**	100
**ANKK1**	100
**BMPR1A**	100
**BMPR1B**	100
**BMPR2**	100
**BTK**	100
**CAMK1D**	100
**CAMK1G**	100
**CAMK2A**	100
**CAMK2B**	100
**CAMK4**	100
**CASK**	100
**CDC42BPA**	100
**CDK2**	100
**CDK7**	100
**CDK8**	100
**CDK13**	100
**CDK14**	100
**CDK16**	100
**CDK17**	100
**CHEK2**	100
**CLK2**	100
**CLK4**	100
**CSNK1D**	100
**CSNK1G1**	100

**CSNK2A1**	100
**CSNK2A2**	100
**DAPK2**	100
**DCLK3**	100
**DSTYK**	100
**EIF2AK1**	100
**EIF2AK2**	100
**EPHA1**	100
**EPHA2**	100
**EPHA3**	100
**EPHA7**	100
**EPHB6**	100
**FER**	100
**FGFR4**	100
**FRK**	100
**FYN**	100
**GRK7**	100
**HCK**	100
**HIPK2**	100
**HIPK3**	100
**IGF1R**	100
**IKBKE**	100
**LATS2**	100
**LRRK2**	100
**LTK**	100
**MAK**	100
**MAP2K1**	100
**MAP2K4**	100
**MAP2K6**	100
**MAP2K7**	100
**MAP3K2**	100
**MAP3K4**	100
**MAP3K12**	100
**MAP3K15**	100
**MAP4K2**	100
**MAPK7**	100
**MAPK11**	100
**MAPKAPK5**	100
**MELK**	100
**MGC42105**	100
**MKNK1**	100
**MKNK2**	100
**MST1R**	100
**MYLK**	100
**MYLK2**	100
**NEK1**	100
**NEK2**	100
**NEK7**	100
**NEK9**	100
**NEK11**	100

**NUAK2**	100
**OXSR1**	100
**PAK1**	100
**PAK2**	100
**PAK4**	100
**PAK6**	100
**PDPK1**	100
**PI4KB**	100
**PIK3C2G**	100
**PIK3CB**	100
**PIK3CD**	100
**PIM1**	100
**PIM2**	100
**PKN1**	100
**PKN2**	100
**PLK1**	100
**PLK2**	100
**PLK3**	100
**PLK4**	100
**PNCK**	100
**PRKAA1**	100
**PRKAA2**	100
**PRKACB**	100
**PRKCD**	100
**PRKD1**	100
**PRKD2**	100
**PRKG2**	100
**PRPF4B**	100
**PTK2B**	100
**PTK6**	100
**RAF1**	100
**RIOK2**	100
**RIPK4**	100
**ROCK2**	100
**ROS1**	100
**RPS6KA4**	100
**SBK1**	100
**SGK2**	100
**SIK2**	100
**SLK**	100
**SNRK**	100
**SRC**	100
**SRPK1**	100
**SRPK2**	100
**STK3**	100
**STK4**	100
**STK10**	100
**STK32A**	100
**STK32B**	100
**STK39**	100

**TAOK1**	100
**TAOK3**	100
**TEK**	100
**TESK1**	100
**TGFBR1**	100
**TGFBR2**	100
**TLK2**	100
**TSSK1B**	100
**TSSK3**	100
**TXK**	100
**TYRO3**	100
**VRK2**	100
**WNK1**	100
**WNK3**	100
**ZAK**	100
**ZAP70**	100

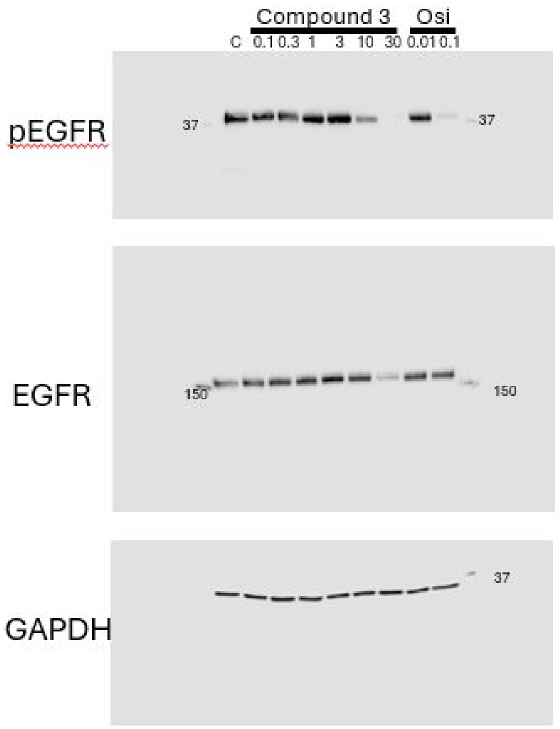

Figure S4: Full Western blot images (from Figure 5) showing cellular inhibition of EGFR phosphorylation (Tyr1068) in H1975 cells after 4 hours

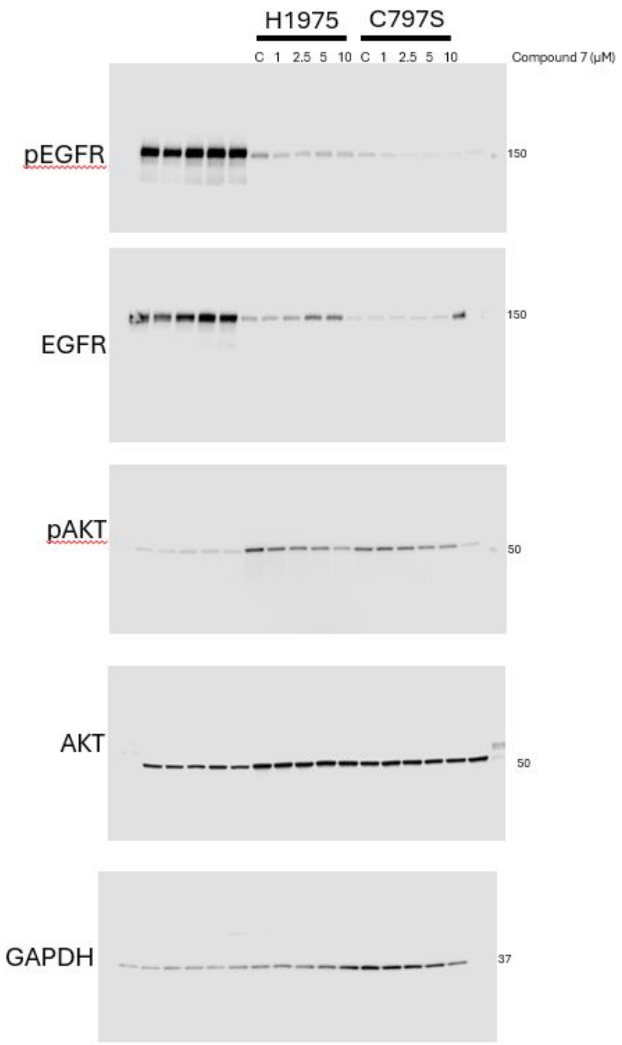

Figure S5: Full Western blot images (from Figure 8) showing cellular inhibition of EGFR phosphorylation (Tyr1068) and AKT phosphorylation in H1975 (L858R, T790M) and CRISPR engineered C797S H1975 cells following 4-hour exposure to compound 7 or vehicle denoted as 'C'.

Table S6: X-ray data collection and refinement statistics

	WT EGFR complex **3** (pdb 9H46)	WT EGFR complex **7** (pdb 9H42)	WT EGFR complex **13** (pdb 9H47)	T790M, C797S L858R mutant EGFR complex **7** (pdb 9S3X)
**Data collection**				
Space group	I23	I23	I23	I23
Unit cell (Å)	a=b=c=144.70	a=b=c=144.89	a=b=c=145.59	a=b=c=143.18
Resolution (Å)<br>(highest resolution shell)	45.76-3.16<br>(3.38-3.16)	45.82-2.60<br>(2.72-2.60)	46.04-2.99<br>(3.17-2.99)	50.62-2.40<br>(2.49-2.40)
Total observations	16741<br>(3055)	636659 (80268)	431268 (68936)	795256 (85592)
Unique	8781 (1580)	15709 (1903)	10543 (1673)	19240 (1999)
R_{merge}	0.029(0.407)	0.175(6.477)	0.451(6.905)	0.169(5.998)
Mean I/σ(I)	15.0 (1.7)	19.2 (0.8)	9.4 (0.8)	16.7 (0.8)
Multiplicity	1.9 (1.9)	40.5 (42.2)	40.9 (41.2)	41.3 (42.8)
Completeness %	100 (100)	100 (100)	100 (100)	100 (100)
CC(1/2)	1.00 (0.712)	1.00 (0.335)	1.00 (0.386)	1.00 (0.564)
**Refinement**				
Number of atoms (B-factor)<br>protein<br>other	 4,670 (117.6)<br>44 (154.1)<br>31 (134.5)	 5,173 (100.3)<br>57 (123.0)	 5,173 (105.0)<br>54 (122.7)	 2599 (84.4)<br>33 (139.6)<br>211 (82.7)
R_{work} (highest resolution shell)	0.212	0.184	0.200	0.189
R_{free} (highest resolution shell)	0.256	0.245	0.244	0.246
Rmsd bonds (Å)	0.0060	0.0073	0.0068	0.0065
Rmsd angles (°)	1.343	1.899	1.729	1.722

The structures have been deposited in the PDB with accession codes 9H46, 9H42, 9H47 & 9S3X.

Experimental Details

Compound S1

3-Bromo-1-methylpyrazolo[3,4-d]pyrimidin-4-amine

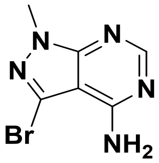

A solution of 3-bromo-1H-pyrazolo[3,4-d]pyrimidin-4-amine (2.00 g, 9.35 mmol, 1.0 eq.), triphenylphosphine (3.00 g, 11.4 mmol, 1.2 eq.) in methanol (10 mL) was treated with DIAD (3.00 g, 14.8 mmol, 1.6 eq.) was stirred for 2 hours at room temperature. The reaction mixture was concentrated under reduced pressure. The residue was purified by flash column chromatography, eluting with ethyl acetate (16%) in 40-60 petroleum ether to afford the title compound as a white solid.

3-(3-Aminophenyl)-1-methylpyrazolo[3,4-d]pyrimidin-4-amine

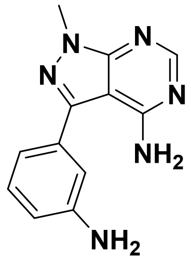

To a stirred solution of 3-bromo-1-methylpyrazolo[3,4-d]pyrimidin-4-amine (300 mg, 1.32 mmol, 1.0 eq.) and m-aminophenylboronic acid (300 mg, 2.19 mmol, 1.7 eq.) in dioxane (5.0 mL) and water (1.0 mL) was added potassium carbonate (400 mg, 2.89 mmol, 2.2 eq.) and [1,1′-bis(diphenylphosphino)ferrocene]dichloropalladium(II) (50.0 mg, 68.0 µmol, 0.050 eq.). After stirring for 2 hours at 100 °C under a nitrogen atmosphere, the resulting mixture was concentrated under reduced pressure. The residue was purified by flash column chromatography, eluting with ethyl acetate (18%) in 40-60 petroleum ether to afford the title compound as a light-yellow solid (200 mg, 63%).

N-(3-{4-Amino-1-methylpyrazolo[3,4-d]pyrimidin-3-yl}phenyl)prop-2-enamide (S1)

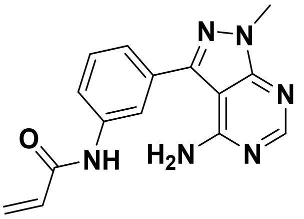

To a solution of 3-(3-aminophenyl)-1-methylpyrazolo[3,4-d]pyrimidin-4-amine (200 mg, 0.832 mmol, 1.0 eq.) in dichloromethane (3.0 mL) was added diisopropylethylamine (200 mg, 1.55 mmol, 1.9 eq.) followed by acryloyl chloride (100 mg, 1.11 mmol, 1.3 eq.) at 0°C. The resulting mixture was stirred for 2 hours at room temperature then quenched with saturated aqueous sodium hydrogen carbonate. The organic layer was separated, dried (Na_2SO_4) and concentrated under reduced pressure. The residue was purified by reverse phase flash column chromatography eluting with acetonitrile (10-50%) in water (0.1% formic acid), to yield the title compound as a white solid (30.0 mg, 12%).

(ES, *m/z*): $[M+H]^+$ = 295.20

^{1}H NMR (500 MHz, DMSO) δ 10.32 (s, 1H), 8.19 (s, 1H), 7.99 (t, *J* = 1.9 Hz, 1H), 7.61 (ddd, *J* = 8.0, 2.2, 1.1 Hz, 1H), 7.43 (t, *J* = 7.9 Hz, 1H), 7.32 (dt, *J* = 7.8, 1.3 Hz, 1H), 6.40 (dd, *J* = 17.0, 10.2 Hz, 1H), 6.23 (dd, *J* = 17.0, 2.0 Hz, 1H), 5.73 (dd, *J* = 10.1, 2.0 Hz, 1H), 3.89 (s, 2H).

^{13}C NMR (126 MHz, $CDCl_3$) δ = 163.90, 158.48, 156.23, 154.79, 143.67, 139.88, 133.86, 132.19, 130.21, 127.70, 123.75, 120.01, 119.70, 97.64, 33.97.

Compound S2

N-(3-{4-amino-1-methylpyrazolo[3,4-d]pyrimidin-3-yl}phenyl)prop-2-ynamide (S2)

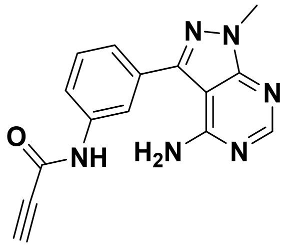

A mixture of 3-(3-aminophenyl)-1-methylpyrazolo[3,4-d]pyrimidin-4-amine (200 mg, 0.832 mmol, 1.0 eq.), diisopropylethylamine (200 mg, 1.55 mmol, 1.9 eq.), HATU (400 mg, 1.05 mmol, 1.3 eq.) and propiolic acid (70.0 mg, 0.999 mmol, 1.2 eq.) in dichloromethane (4.0 mL) was stirred for 2 hours at room temperature. The residue was purified by reverse phase flash column chromatography eluting with acetonitrile (10-50%) in water (0.1% trifluoroacetic acid) to yield the title compound as a white solid (30.0 mg, 12%).

(ES, *m/z*): $[M+H]^+$ = 293.10

^{1}H NMR (500 MHz, DMSO) δ 11.04 (s, 1H), 8.26 (s, 1H), 7.97 (t, *J* = 1.9 Hz, 1H), 7.63 (d, *J* = 7.8 Hz, 1H), 7.50 (t, *J* = 7.9 Hz, 1H), 7.41 (d, *J* = 7.6 Hz, 1H), 4.47 (d, *J* = 3.4 Hz, 1H), 3.96 (s, 3H)

^{13}C NMR (126 MHz, DMSO) δ 158.46, 156.24, 154.78, 150.38, 143.50, 139.09, 133.92, 130.23, 124.49, 120.33, 120.00, 97.62, 78.75, 77.97, 33.98.

Compound S3

3-[3-(Aminomethyl)phenyl]-1-methylpyrazolo[3,4-d]pyrimidin-4-amine

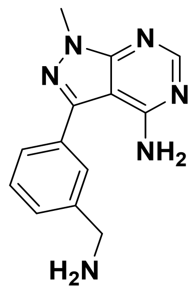

To a stirred solution of 3-bromo-1-methylpyrazolo[3,4-d]pyrimidin-4-amine (200 mg, 0.877 mmol, 1.0 eq.) and 3-(aminomethyl)phenylboronic acid (200 mg, 1.33 mmol, 1.5 eq.) in dioxane (4.0 mL) and water (1.0 mL) was added potassium carbonate (260 mg, 1.88 mmol, 2.2 eq.) and [1,1'-bis(diphenylphosphino)ferrocene]dichloropalladium(II) (60.0 mg, 82.0 µmol, 0.090 eq.) . After stirring for 2 hours at 100 °C under a nitrogen atmosphere, the resulting mixture was concentrated under reduced pressure. The residue was purified by flash column chromatography, eluting with ethyl acetate (15%) to afford the title compound as a light-yellow solid (200 mg, 89%).

N-[(3-{4-Amino-1-methylpyrazolo[3,4-d]pyrimidin-3-yl}phenyl)methyl]prop-2-enamide (S3)

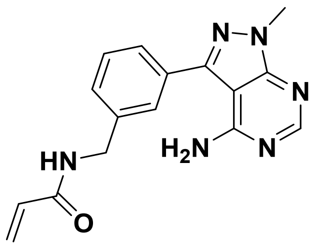

A solution of 3-[3-(aminomethyl)phenyl]-1-methylpyrazolo[3,4-d]pyrimidin-4-amine (200 mg, 0.786 mmol, 1.0 eq.), diisopropylethylamine (200 mg, 1.55 mmol, 2.0 eq.) in dichloromethane (3.0 mL) was treated with acryloyl chloride (100 mg, 1.11 mmol, 1.4 eq.) was stirred for 2 hours at room temperature. The residue was purified by reverse phase flash column chromatography eluting with acetonitrile (10-50%) in water (0.1% formic acid) to yield the title compound as a white solid (30.0 mg, 12%).

(ES, *m/z*): $[M+H]^+$ = 309.20

^{1}H NMR (500 MHz, DMSO) δ 8.64 (t, *J* = 6.0 Hz, 1H), 8.19 (s, 1H), 7.52 – 7.41 (m, 3H), 7.31 (dt, *J* = 7.6, 1.6 Hz, 1H), 6.21 (dd, *J* = 17.1, 10.2 Hz, 1H), 6.08 (dd, *J* = 17.2, 2.2 Hz, 1H), 5.56 (dd, *J* = 10.2, 2.1 Hz, 1H), 4.37 (d, *J* = 6.0 Hz, 2H), 3.89 (s, 3H)

^{13}C NMR (126 MHz, DMSO) δ 165.18, 158.48, 156.22, 154.75, 143.76, 140.65, 133.39, 132.01, 129.69, 128.20, 127.66, 127.18, 126.10, 97.64, 42.73, 33.95.

Compound S6

3-{4-Amino-1-methylpyrazolo[3,4-d]pyrimidin-3-yl}phenol

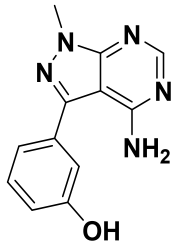

To a stirred solution of 3-bromo-1-methylpyrazolo[3,4-d]pyrimidin-4-amine (500 mg, 2.19 mmol, 1.0 eq.) and 3-hydroxyphenylboronic acid (500 mg, 3.63 mmol, 1.6 eq.) in dioxane (5.0 mL) and water (1.0 mL) was added potassium carbonate (520 mg, 3.76 mmol, 1.7 eq.) and [1,1′-bis(diphenylphosphino)ferrocene]dichloropalladium(II) (120 mg, 0.164 mmol, 0.070 eq.). After stirring for 2 hours at 100 °C under a nitrogen atmosphere, the resulting mixture was concentrated under reduced pressure. The residue was purified by flash column chromatography, eluting with ethyl acetate (15%) in 40-60 petroleum ether to afford the title compound as a light-yellow solid (400 mg, 75%).

tert-Butyl N-[2-(3-{4-amino-1-methylpyrazolo[3,4-d]pyrimidin-3-yl}phenoxy)ethyl]carbamate

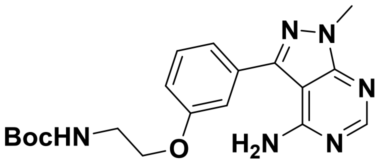

A solution of 3-{4-amino-1-methylpyrazolo[3,4-d]pyrimidin-3-yl}phenol (100 mg, 0.415 mmol, 1.0 eq.), potassium carbonate (120 mg, 0.868 mmol, 2.1 eq.), tert-butyl N-(2-bromoethyl)carbamate (120 mg, 0.535 mmol, 1.3 eq.) and acetone (2.0 mL) was stirred for 2 hours at 60 °C. The reaction mixture was concentrated under reduced pressure. The residue was purified by flash column chromatography eluting with ethyl acetate (10%) in 40-60 petroleum ether to afford the title compound as a white solid (130 mg, 81%).

N-[2-(3-{4-Amino-1-methylpyrazolo[3,4-d]pyrimidin-3-yl}phenoxy)ethyl]prop-2-enamide (S6)

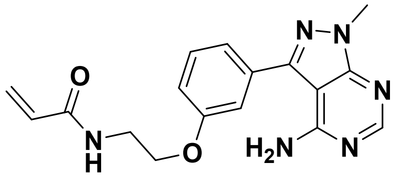

A solution of tert-butyl N-[2-(3-{4-amino-1-methylpyrazolo[3,4-d]pyrimidin-3-yl}phenoxy)ethyl]carbamate (100 mg, 0.260 mmol, 1.0 eq.) in dichloromethane (2.0 mL) was treated with trifluoroacetic acid (1.0 mL). The

resulting solution was stirred for 1 hour at room temperature and then concentrated under reduced pressure. The residue was dissolved in dichloromethane (3.0 mL). The pH was adjusted to 8-9 with triethylamine then acryloyl chloride (20.0 mg, 0.221 mmol, 0.85 eq.) and the reaction stirred for 2 hours at room temperature. The reaction mixture was purified by reverse phase flash column chromatography eluting with acetonitrile (10-50%) in water (0.1% trifluoroacetic acid) to afford the title compound as a white solid (10.0 mg, 11%).

(ES, *m/z*): $[M+H]^+$ = 339.30

^{1}H NMR (500 MHz, DMSO) δ 8.34 (d, *J* = 5.0 Hz, 1H), 8.19 (s, 1H), 7.39 (t, *J* = 7.9 Hz, 1H), 7.17 (d, *J* = 7.7 Hz, 1H), 7.14 – 7.10 (m, 1H), 7.00 (dd, *J* = 8.1, 2.9 Hz, 1H), 6.20 (dd, *J* = 17.1, 10.2 Hz, 1H), 6.04 (dd, *J* = 17.2, 2.2 Hz, 1H), 5.53 (dd, *J* = 10.1, 2.2 Hz, 1H), 4.04 (t, *J* = 5.6 Hz, 3H), 3.89 (s, 3H), 3.48 (q, *J* = 5.5 Hz, 2H).

^{13}C NMR (126 MHz, DMSO) δ 165.35, 159.24, 158.54, 156.21, 154.70, 143.71, 134.72, 132.04, 130.79, 125.78, 121.02, 115.50, 114.53, 97.72, 66.82, 38.75, 33.95.

Compound (S7)

4-{4-Amino-1-methylpyrazolo[3,4-d]pyrimidin-3-yl}phenol

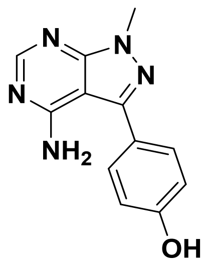

To a stirred solution of 3-bromo-1-methylpyrazolo[3,4-d]pyrimidin-4-amine (1.00 g, 4.38 mmol, 1.0 eq.) and 4-hydroxyphenylboronic acid (1.00 g, 7.25 mmol, 1.65 eq.) in dioxane (20 mL) and water (5.0 mL) was added potassium carbonate (1.30 g, 9.40 mmol, 2.15 eq.) and [1,1'-bis(diphenylphosphino)ferrocene]dichloropalladium(II) (200 mg, 0.273 mmol, 0.06 eq.) . After stirring for 2 hours at 100 °C under a nitrogen atmosphere, the resulting mixture was concentrated under reduced pressure. The crude was purified by flash column chromatography, eluting with ethyl acetate (50%) in 40-60 petroleum ether to afford the title compound as a white solid (1.00 g, 94%).

tert-Butyl N-[2-(4-{4-amino-1-methylpyrazolo[3,4-d]pyrimidin-3-yl}phenoxy)ethyl]carbamate

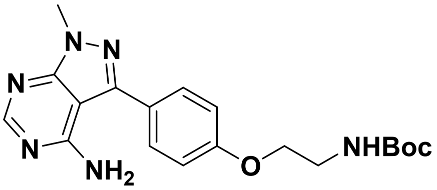

A mixture of 4-{4-amino-1-methylpyrazolo[3,4-d]pyrimidin-3-yl}phenol (100 mg, 0.415 mmol, 1 eq.), potassium carbonate (120 mg, 0.868 mmol, 2.1 eq.) and tert-butyl N-(2-bromoethyl)carbamate (120 mg, 0.535 mmol, 1.3 eq.) in acetone (2.0 mL) was stirred for 2 hours at 60 °C. The reaction mixture was diluted with ethyl acetate (20 mL) and washed with water (20 mL). The organic layer was separated and concentrated under reduced pressure. The crude material was purified by flash column chromatography, eluting with ethyl acetate (10%) in 40-60 petroleum ether to afford the title compound as a white solid (130 mg, 81%).

N-[2-(4-{4-amino-1-methylpyrazolo[3,4-d]pyrimidin-3-yl}phenoxy)ethyl] prop-2-enamide (S7)

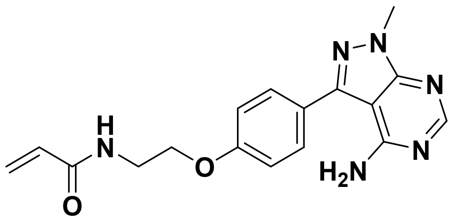

A solution of tert-butyl N-[2-(4-{4-amino-1-methylpyrazolo[3,4-d]pyrimidin-3-yl}phenoxy)ethyl]carbamate (100 mg, 0.260 mmol, 1 eq.) in dichloromethane (2.0 mL) was treated with trifluoroacetic acid (1.0 mL) for 1 hour at room temperature under nitrogen atmosphere then concentration to dryness under reduced pressure. The residue was dissolved in dichloromethane (3.0 mL) then triethylamine was added followed by the dropwise addition of acryloyl chloride (20.0 mg, 0.221 mmol, 0.85 eq.) at room temperature. The resulting mixture was stirred for 2 hours at room temperature. The residue was purified by reverse-phase flash column chromatography eluting with acetonitrile (10-50%) in water (0.1% trifluoroacetic acid) to yield the title compound as a white solid (10.0 mg, 11%).

(ES, *m/z*): $[M+H]^+$ = 339.25

^{1}H NMR (500 MHz, DMSO) δ 8.43 (t, *J* = 5.4 Hz, 1H), 8.25 (s, 1H), 7.61 – 7.55 (m, 2H), 7.15 – 7.09 (m, 2H), 6.28 (dd, *J* = 17.1, 10.2 Hz, 1H), 6.12 (dd, *J* = 17.0, 2.2 Hz, 1H), 5.61 (dd, *J* = 10.1, 2.2 Hz, 1H), 4.15 – 4.08 (m, 3H), 3.93 (s, 3H), 3.55 (q, *J* = 5.6 Hz, 2H).

^{13}C NMR (126 MHz, DMSO) δ 177.84, 165.37, 159.14, 158.60, 156.17, 154.64, 143.71, 139.96, 132.03, 130.00, 125.83, 115.59, 97.66, 49.07, 38.78, 33.86.

Compound S8

4-Bromophenyl methanesulfonate

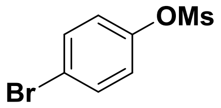

To a stirred solution of 4-bromophenol (10.0 g, 57.8 mmol, 1.0 eq.) and triethylamine (8.77 g, 86.7 mmol, 1.5 eq.) in dichloromethane (100 mL) was added methane sulfonyl chloride (7.94 g, 69.4 mmol, 1.2 eq.) dropwise at 0 °C under nitrogen atmosphere. The resulting mixture was stirred for 2 hours at room temperature then diluted with water (200 mL) and extracted with dichloromethane (2 x 50 mL). The combined organic layers were washed with brine (2 x 30 mL), dried (Na_2SO_4) and concentrated under reduced pressure. The residue was purified by flash column chromatography, eluting with ethyl acetate (18%) in 40-60 petroleum ether to afford the title compound as an off-white solid (11.0 g, 76%).

1-Bromo-4-(2-nitrophenoxy)benzene

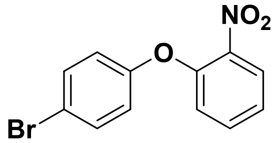

To a stirred solution of 4-bromophenyl methanesulfonate (11.0 g, 43.8 mmol, 1.0 eq.) and *O*-fluoronitrobenzene (6.49 g, 46.0 mmol, 1.1 eq.) in DMSO (100 mL) was added cesium carbonate (18.6 g, 56.9 mmol, 1.3 eq.). The resulting mixture was stirred for 4 hpurs at 80°C then diluted with water (300 mL) and extracted with ethyl acetate (3 x 50 mL). The combined organic layers were washed with brine (2 x 30 mL), dried (Na_2SO_4) and concentrated under reduced pressure. The residue was purified by flash column chromatography, eluting with ethyl acetate (30%) in 40-60 petroleum ether to afford the title compound as a light-yellow solid (9.00 g, 70%).

4,4,5,5-Tetramethyl-2-[4-(2-nitrophenoxy)phenyl]-1,3,2-dioxaborolane

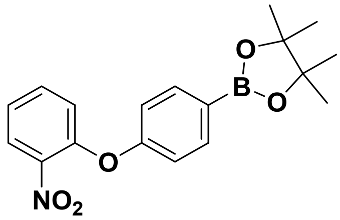

To a stirred solution of 1-bromo-4-(2-nitrophenoxy)benzene (5.00 g, 17.0 mmol, 1.0 eq.) and bis(pinacolato)diboron (5.18 g, 20.4 mmol, 1.2 eq.) in dioxane (50 mL) was added potassium acetate (3.34 g, 34.0 mmol, 2.0 eq.) and [1,1'-bis(diphenylphosphino)ferrocene]dichloropalladium(II) (620 mg, 0.850 mmol, 0.050 eq.). After stirring overnight at 100 °C under a nitrogen atmosphere, the mixture was filtered, washing with ethyl acetate (3 x 30 mL). The filtrate was concentrated under reduced pressure. The residue was purified by flash column chromatography, eluting with ethyl acetate (50%) in 40-60 petroleum ether to afford the title compound as a light-yellow solid (3.00 g, 52%).

1-Methyl-3-[4-(2-nitrophenoxy)phenyl]pyrazolo[3,4-d]pyrimidin-4-amine

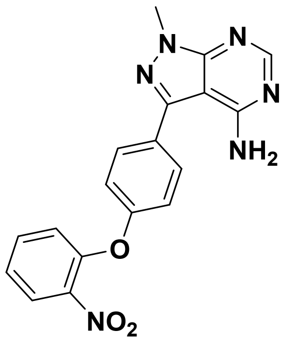

To a solution of 4,4,5,5-tetramethyl-2-[4-(2-nitrophenoxy)phenyl]-1,3,2-dioxaborolane (1.50 g, 4.40 mmol, 1.0 eq.) and 3-bromo-1-methylpyrazolo[3,4-d]pyrimidin-4-amine (1.10 g, 4.84 mmol, 1.1 eq.) in dioxane (10 mL) and water (2.0 mL) was added potassium acetate (860 mg, 8.79 mmol, 2.0 eq.) and [1,1'-bis(diphenylphosphino)ferrocene]dichloropalladium(II) (160 mg, 0.220 mmol, 0.050 eq.). After stirring overnight at 100 °C under a nitrogen atmosphere. The mixture was cooled to room temperature then filtered, the filter cake was washed with ethyl acetate (3 x 10 mL) and the filtrate was concentrated under reduced pressure. The residue was purified by flash column chromatography, eluting with ethyl acetate (50%) in 40-60 petroleum ether to afford the title compound as a yellow solid (700 mg, 44%).

3-[4-(2-Aminophenoxy)phenyl]-1-methylpyrazolo[3,4-d]pyrimidin-4-amine

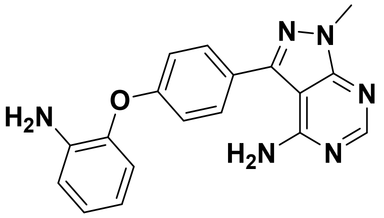

To a stirred solution of 1-methyl-3-[4-(2-nitrophenoxy)phenyl]pyrazolo[3,4-d]pyrimidin-4-amine (300 mg, 0.828 mmol, 1.0 eq.) and ammonium chloride (443 mg, 8.28 mmol, 10 eq.) in THF (4.0 mL) and water (1.0 mL) was added zinc (271 mg, 4.14 mmol, 5.0 eq.) in portions at 0 °C under nitrogen atmosphere. The resulting mixture was stirred for 2 hours at room temperature then filtered, washing with THF (2 x 5 mL). The filtrate was diluted with water (10 mL) and extracted with ethyl acetate (3 x 10 mL), dried (Na_2SO_4) and concentrated under reduced pressure to yield the title compound as a light brown oil (200 mg, 73%), which was used in the next step directly without further purification.

N-[2-(4-{4-Amino-1-methylpyrazolo[3,4-d]pyrimidin-3-yl}phenoxy)phenyl]prop-2-enamide (S8)

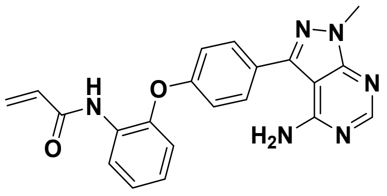

To a stirred solution of 3-[4-(2-aminophenoxy)phenyl]-1-methylpyrazolo[3,4-d]pyrimidin-4-amine (100 mg, 0.301 mmol, 1.0 eq.) and diisopropylethylamine (77.8 mg, 0.602 mmol, 2.0 eq.) in dichloromethane (1.0 mL) was added acryloyl chloride (27.2 mg, 0.301 mmol, 1.0 eq.) dropwise at 0 °C. The resulting mixture was stirred for 2 hours at room temperature then concentrated under reduced pressure. The residue was purified by reverse phase flash column chromatography eluting with acetonitrile (20-40%) in water (0.1% ammonium carbonate), to yield the title compound as a white solid (2.50 mg, 2%).

(ES, *m/z*): $[M+H]^+$ = 387.10

^1H NMR (500 MHz, DMSO) δ 9.73 (s, 1H), 8.25 (s, 1H), 8.09 (d, *J* = 8.2 Hz, 1H), 7.65 (d, *J* = 8.5 Hz, 2H), 7.16 (dd, *J* = 14.2, 6.5 Hz, 4H), 7.10 – 7.04 (m, 1H), 6.62 (dd, *J* = 17.0, 10.2 Hz, 1H), 6.27 – 6.19 (m, 1H), 5.73 – 5.67 (m, 1H), 3.95 (s, 3H)

^{13}C NMR (126 MHz, DMSO) δ 163.96, 160.35, 158.56, 157.51, 156.19, 154.70, 149.29, 143.37, 132.19, 130.26, 130.20, 128.47, 127.33, 125.72, 124.40, 119.73, 119.32, 97.72, 33.90.

Compound S9

N-[2-(4-{4-Amino-1-methylpyrazolo[3,4-d]pyrimidin-3-yl}phenoxy)phenyl]prop-2-ynamide (S9)

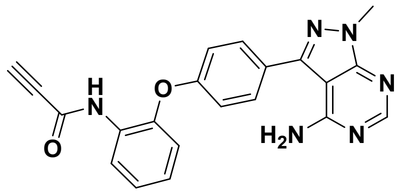

A solution of 3-[4-(2-aminophenoxy)phenyl]-1-methylpyrazolo[3,4-d]pyrimidin-4-amine (100 mg, 0.301 mmol, 1.0 eq.), propiolic acid (21.1 mg, 0.301 mmol, 1.0 eq.), HATU (137 mg, 0.361 mmol, 1.2 eq.) and diisoporpylethylamine (58.3 mg, 0.452 mmol, 1.5 eq.) in DMF (1.0 mL) was stirred for 2 hours at room temperature. The reaction mixture was purified by reverse phase flash column chromatography eluting with acetonitrile (25-43%) in water (0.1% ammonium carbonate) to yield the title compound as a light brown solid (10.0 mg, 8%).

(ES, *m/z*): $[M+H]^+$ = 385.05

^1H NMR (500 MHz, DMSO) δ 10.38 (s, 1H), 8.19 (s, 1H), 7.64 – 7.55 (m, 3H), 7.23 – 7.09 (m, 2H), 7.08 – 7.00 (m, 3H), 4.26 (s, 1H), 3.88 (s, 3H)

^{13}C NMR (126 MHz, DMSO) δ 158.57, 157.38, 156.23, 154.70, 150.73, 149.30, 143.40, 130.27, 128.55, 128.47, 127.40, 126.61, 124.39, 119.96, 119.38, 97.69, 78.63, 77.87, 33.92.

Compound S4

2-(Methylsulfonyl)-7-oxo-8-phenyl-7,8-dihydropyrido[2,3-d]pyrimidin-5-yltrifluoromethanesulfonate

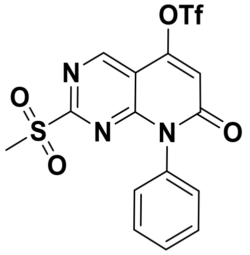

A solution of 2-(methylthio)-7-oxo-8-phenyl-7,8-dihydropyrido[2,3-d]pyrimidin-5-yl trifluoromethanesulfonate (600 mg,1.44 mmol, 1.0 eq.) and m-CPBA (744 mg, 4.32 mmol, 70% wt, 3.0 eq.) in dichloromethane (15mL) was stirred at room temperature for 4.5 hours. The reaction mixture was washed with saturated aqueous sodium thiosulfate solution solution, dried (Na_2SO_4) and concentrated under reduced pressure to yield the title compound as a yellow solid (480 mg, 74%), which was used in next step directly.

2-((2-Methoxyphenyl)amino)-7-oxo-8-phenyl-7,8-dihydropyrido[2,3-d]pyrimidin-5-yl trifluoromethanesulfonate

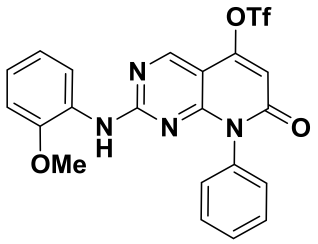

A solution of 2-(methylsulfonyl)-7-oxo-8-phenyl-7,8-dihydropyrido[2,3-d]pyrimidin-5-yl trifluoromethanesulfonate (480 mg, 1.07 mmol, 1.0 eq.), 2-methoxyaniline (131 mg, 1.07 mmol, 1.0 eq.), trifluoroacetic acid (122 mg, 1.07 mmol, 1.0 eq.) in 2-butanol (4.0 mL) was stirred at 110 ^oC overnight. The reaction mixture was cooled to room temperature then purified by reverse phase flash column chromatography eluting with acetonitrile (10-100%) in water (0.1% ammonium carbonate) to yield the title compound as a yellow solid (330 mg, 63%).

2-((2-Methoxyphenyl)amino)-7-oxo-8-phenyl-7,8-dihydropyrido[2,3-d]pyrimidine-5-carbonitrile

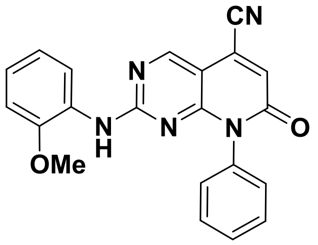

A solution of 2-((2-methoxyphenyl)amino)-7-oxo-8-phenyl-7,8-dihydropyrido[2,3-d]pyrimidin-5-yltrifluoromethanesulfonate (2.20 g, 4.40 mmol 1.0 eq.) and zinc cyanide (520 mg, 4.40 mmol 1.0 eq.), zinc acetate (32.0 mg, 0.176 mmol 0.040 eq.) tris(dibenzylideneacetone)dipalladium(0)-chloroform adduct (120 mg, 0.132 mmol 0.030 eq.), 1,1'-bis(diphenylphosphino)ferrocene (183 mg, 0.330 mmol 0.075 eq.) in DMF (4.0 mL). was stirred at 100 ^oC for 1 hour then concentrated under reduced pressure. The crude product was purified by flash column chromatography to yield the title compound as a yellow oil (650 mg, 40%).

tert-Butyl ((2-((2-methoxyphenyl)amino)-7-oxo-8-phenyl-7,8-dihydropyrido[2,3-d]pyrimidin-5-yl)methyl)carbamate

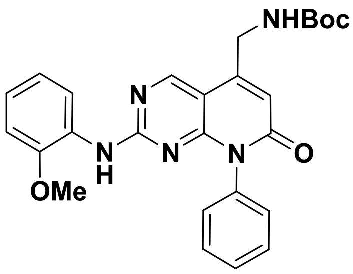

A solution of 2-((2-methoxyphenyl)amino)-7-oxo-8-phenyl-7,8-dihydropyrido[2,3-d]pyrimidine-5-carbonitrile (650 mg, 1.76 mmol, 1.0 eq.) and palladium on carbon (6.50 mg), boc-anhydride (655 mg, 3.52 mmol, 2.0 eq.) in methanol (10 mL) was stirred under hydrogen at room temperature for 1 hour at 1 atm. The reaction mixture was filtered through a Celite pad and the filtrate concentrated under reduced pressure to yield the title compound as a yellow solid (400 mg, 78%).

(ES, *m/z*): $[M+H]^+$ = 474.5

5-(Aminomethyl)-2-((2-methoxyphenyl)amino)-8-phenylpyrido[2,3-d]pyrimidin-7(8H)-one

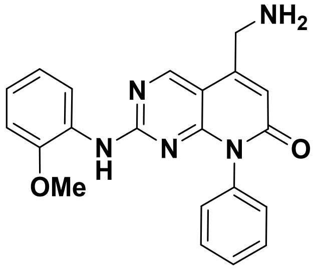

To a stirred solution of tert-butyl ((2-((2-methoxyphenyl)amino)-7-oxo-8-phenyl-7,8-dihydropyrido[2,3-d]pyrimidin-5-yl)methyl)carbamate (5.00 g, 17.2 mmol, 1.0 eq.) in dichloromethane (5.0 mL) was added hydrogen chloride (4M in ethyl acetate, 5.0 mL). The resulting solution was stirred for 1 hour at room temperature then concentrated under reduced pressure to yield the title compound as a reddish yellow solid (350 mg, 5%).

N-(3-(2-((2-Methoxyphenyl)amino)-7-oxo-8-phenyl-7,8-dihydropyrido [2,3-d]pyrimidin-5-yl)benzyl)acrylamide (S4)

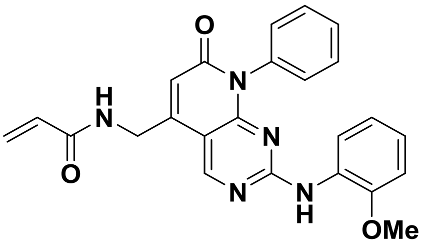

To a stirred solution of 5-(aminomethyl)-2-((2-methoxyphenyl)amino)-8-phenylpyrido[2,3-d]pyrimidin-7(8H)-one (60.0 mg, 0.161 mmol, 1.0 eq.) and dichloromethane (2.0 mL) was added triethylamine (150 mg, 0.483 mmol, 3.0 eq.) dropwise at 0 ^oC followed by acryloyl chloride (14.0 mg, 0.161 mmol, 1.0 eq.). The resulting mixture was stirred for 1 hour at room temperature. The resulting solution was washed with water (5.0 mL) then dried (Na_2SO_4) and concentrated under reduced pressure. The residue was purified by reverse phase flash column chromatography eluting with acetonitrile (10-100%) in water (0.05% ammonium carbonate). The collected fractions were combined and lyophilized to dryness to yield the title compound as a white solid (6.60 mg, 10%).

(ES, *m/z*): $[M+H]^+$ = 428.5

^{1}H NMR (500 MHz, DMSO) δ 8.97 (s, 1H), 8.79 (t, *J* = 6.0 Hz, 1H), 8.27 (s, 1H), 7.62 – 7.51 (m, 3H), 7.39 (d, *J* = 7.6 Hz, 1H), 7.35 – 7.29 (m, 2H), 6.95 (d, *J* = 1.6 Hz, 0H), 6.89 (t, *J* = 7.4 Hz, 1H), 6.45 (s, 1H), 6.36 (dd, *J* = 17.0, 10.2 Hz, 1H), 6.30 (d, *J* = 1.3 Hz, 1H), 6.20 (dd, *J* = 17.1, 2.1 Hz, 1H), 5.71 (dd, *J* = 10.2, 2.2 Hz, 1H), 4.68 (d, *J* = 4.7 Hz, 2H), 3.82 (s, 3H).

^{13}C NMR (126 MHz, DMSO) δ 167.95, 165.42, 162.64, 156.99, 156.54, 147.46, 137.17, 131.61, 129.71, 129.44, 128.57, 128.01, 126.69, 120.40, 115.51, 110.92, 105.34, 100.08, 88.81, 83.12, 56.26, 38.65.

Compound S5

N-((2-((2-methoxyphenyl)amino)-7-oxo-8-phenyl-7,8-dihydropyrido[2,3-d]pyrimidin-5-yl)methyl)-3-(trimethylsilyl)propiolamide

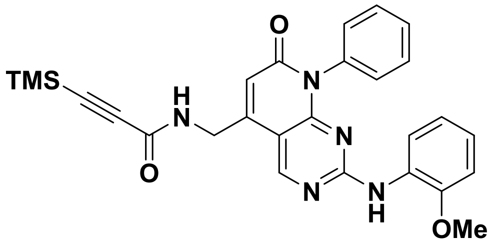

To a stirred solution of 5-(aminomethyl)-2-((2-methoxyphenyl)amino)-8-phenylpyrido[2,3-d]pyrimidin-7(8H)-one (60.0 mg, 0.161 mmol, 1.0 eq.), DCC (10.0 mg, 0.161 mmol, 1.0 eq.) in dichloromethane (4.0 mL) was added 3-(trimethylsilyl)propiolic acid (12.0 mg, 0.161 mmol, 1.0 eq.) and the resulting mixture was stirred at room temperature for 1 hour. The resulting solution was washed with water (5.0 mL) then dried (Na_2SO_4) and concentrated under reduced pressure to yield the crude title compound as a yellow oil (70.0 mg, 88%), which was used in next step directly.

N-((2-((2-Methoxyphenyl)amino)-7-oxo-8-phenyl-7,8-dihydropyrido[2,3-d]pyrimidin-5-yl)methyl)propiolamide (S5)

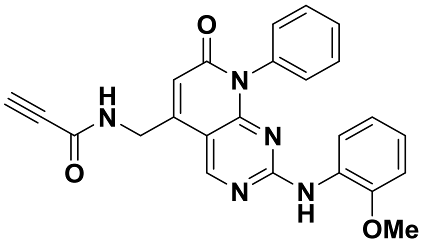

A solution of N-((2-((2-methoxyphenyl)amino)-7-oxo-8-phenyl-7,8-dihydropyrido[2,3-d]pyrimidin-5-yl)methyl)-3- (trimethylsilyl)propiolamide (60.0 mg, 0.121 mmol, 1.0 eq.) and potassium fluoride (10.0 mg, 0.174 mmol, 1.4 eq.) in methanol (2.0 mL) was stirred at room temperature for 1 hour. The reaction mixture was diluted with dichloromethane (10 mL), washed with water (5.0 mL) and concentrated under reduced pressure. The residue was purified by reverse phase flash column chromatography eluting with acetonitrile (5-95%) in water (0.05% ammonium carbonate) to yield the title compound as a white solid (8.10 mg, 16%).

(ES, *m/z*): $[M+H]^+$ = 307.2

^1H NMR (500 MHz, DMSO) δ 9.52 (t, *J* = 5.9 Hz, 1H), 9.00 (s, 1H), 8.36 (d, *J* = 20.5 Hz, 1H), 7.68 – 7.57 (m, 3H), 7.44 (d, *J* = 7.6 Hz, 1H), 7.42 – 7.36 (m, 2H), 7.05 – 6.99 (m, 1H), 6.96 (t, *J* = 7.6 Hz, 1H), 6.51 (s, 1H), 6.33 (d, *J* = 1.3 Hz, 1H), 4.69 (d, *J* = 5.0 Hz, 2H), 4.38 (s, 1H), 3.88 (s, 3H).

^{13}C NMR (126 MHz, DMSO) δ 176.84, 174.31, 162.57, 158.57, 156.98, 156.51, 152.43, 146.54, 137.14, 136.10, 129.71, 129.44, 128.58, 127.99, 120.40, 115.46, 110.92, 105.19, 82.13, 79.71, 79.65, 79.44, 79.18, 77.29, 59.36, 56.25, 40.58, 40.49, 40.42, 40.32, 40.25, 40.16, 40.08, 39.99, 39.91, 39.82, 39.66, 39.58, 39.49.

Compound S10

2-(methylthio)-4-(phenylamino)pyrimidine-5-carbaldehyde

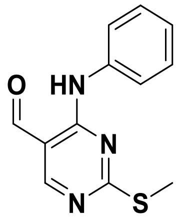

To a stirred solution of ethyl 2-(methylthio)-4-(phenylamino)pyrimidine-5-carboxylate (1.00 g, 3.46 mmol, 1.0 eq.) in THF (6.9 mL, 0.5 M) at 0 °C was added lithium aluminium hydride (6.90 mL, 6.90 mmol, 1.0 M sol. in THF, 2.0 eq.) dropwise. The reaction was stirred at 0 °C for 30 minutes, then warmed to room temperature and stirred overnight. Once complete, the reaction was quenched by the dropwise addition of ethyl acetate until cessation of bubbling followed after 1 hour by the addition of saturated aqueous Rochelle salts solution (20 mL). After stirring at room temperature overnight, the aqueous was extracted with dichloromethane (3 x 50 mL). The combined organic layers were washed with brine (30 mL), dried ($MgSO_4$) and concentrated under reduced pressure to yield the crude (2-(methylthio)-4-(phenylamino)pyrimidin-5-yl)methanol.

(ES, *m/z*): $[M+H]^+$ = 248.1

The crude (2-(methylthio)-4-(phenylamino)pyrimidin-5-yl)methanol was dissolved in dichloromethane (4.0 mL, 0.25 M) and manganese (IV) dioxide (740 mg, 8.50 mmol, 10 eq.) was added to the stirred solution. The reaction was stirred at room temperature overnight, then filtered through celite, washing the filter pad with dichloromethane (20 mL) and concentrated under reduced pressure. The crude material was purified by flash column chromatography eluting with ethyl acetate (5 %) in 40-60 petroleum ether to yield the title compound as a colourless oil (467 mg, 1.84 mmol, 53%).

(ES, *m/z*): $[M+H]^+$ = 246.1

^{1}H NMR (500 MHz, $CDCl_3$): δ 10.63 (1H, s), 9.79 (1H, s), 8.4 (1H, s), 7.73 (2H, d, *J* = 7.6 Hz), 7.39 (2H, t, *J* = 7.6 Hz), 7.18 (1H, tt, *J* = 7.3, 1.1 Hz), 2.58 (3H, s).

Ethyl (*E*)-3-(2-(methylthio)-4-(phenylamino)pyrimidin-5-yl)acrylate

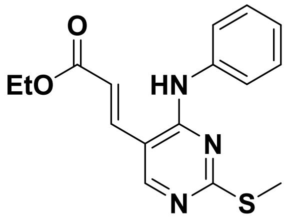

A solution of 2-(methylthio)-4-(phenylamino)pyrimidine-5-carbaldehyde (99.0 mg, 0.404 mmol, 1.0 eq.) and ethyl (triphenylphosphoranylidene) acetate (171 mg, 0.490 mmol, 1.2 eq.) in THF (2.0 mL, 0.2 M) was stirred at relux for 2 hours. Once complete, the reaction was cooled to room temperature, filtered and concentrated under reduced pressure. The crude material was purified by flash column chromatography eluting with ethyl acetate (5 %) in 40-60 petroleum ether to yield the title compound as a white solid (101 mg, 0.319 mmol, 79%).

(ES, *m/z*): $[M+H]^+$ = 316.1

^{1}H NMR (500 MHz, $CDCl_3$): δ = 8.32 (1H, s), 7.67 (1H, d, *J* = 15.9 Hz), 7.60 (2H, d, *J* = 7.7 Hz), 7.39 (2H, t, *J* = 7.7 Hz), 7.18 (1H, t, *J* = 7.7 Hz), 6.86 (1H, s), 6.42 (1H, d, *J* = 15.9 Hz), 4.30 (2H, q, *J* = 7.1 Hz), 2.52 (3H, s), 1.37 (3H, t, *J* = 7.1 Hz)

2-(methylthio)-8-phenylpyrido[2,3-*d*]pyrimidin-7(8*H*)-one

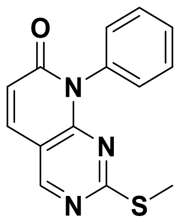

A solution of ethyl (*E*)-3-(2-(methylthio)-4-(phenylamino)pyrimidin-5-yl)acrylate (216 mg, 0.687 mmol, 1.0 eq.) and DBU (0.210 mL, 1.37 mmol, 2.0 eq.) in diisopropylethylamine (1.7 mL, 0.4 M) and THF (0.46 mL, 1.5 M) was stirred at 120 °C overnight then cooled to room temperature and quenched with water (10 mL). The aqueous was acidifed to pH 2 using hydrochloric acid solution (6 M aq. sol.) and extracted with dichloromethane (3 x 20 mL). The combined organic layers were washed with brine (10 ml), dried ($MgSO_4$) and concentrated under reduced pressure. The crude material was purified by flash column chromatography eluting with methanol (2-10 %) in dichloromethane to yield the title compound as a white solid (84.0 mg, 0.312 mmol, 45%).

(ES, *m/z*): $[M+H]^+$ = 270.1

^1H NMR (500 MHz, $CDCl_3$): δ = 8.68 (1H, s), 7.73 (1H, d, *J* = 9.6 Hz), 7.57 (2H, t, *J* = 7.6 Hz), 7.50 (1H, t, *J* = 7.3 Hz), 7.27 (2H, s), 6.77 (1H, d, *J* = 9.6 Hz), 2.17 (3H, s).

2-(methylsulfonyl)-8-phenylpyrido[2,3-*d*]pyrimidin-7(8*H*)-one

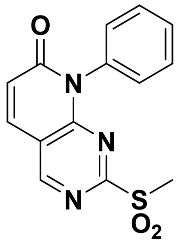

A solution of 2-(methylthio)-8-phenylpyrido[2,3-*d*]pyrimidin-7(8*H*)-one **5** (84.0 mg, 0.312 mmol, 1.0 eq.) and mCPBA (162 mg, 0.936 mmol, 3.0 eq.) in dichloromethane (6.0 mL, 0.05 M) was stirred at room temperature for 2 hours, then quenched with saturated aqueous sodium hydrogen carbonate solution (20 mL). The aqueous was extracted with dichloromethane (3 x 20 mL) and the combined organic layers were washed dried ($MgSO_4$) and concentrated under reduced pressure to yield the title compound **6** as a yellow solid (83.2 mg, 0.276 mmol, 88%) which was carried forward without further purification.

(ES, *m/z*): $[M+H]^+$ = 302.2

^1H NMR (500 MHz, $CDCl_3$): δ = 9.10 (1H, s), 7.90 (1H, d, *J* = 9.6 Hz), 7.60 (1H, dd, *J* = 8.3, 6.6 Hz), 7.55 (1H, t, *J* = 7.4 Hz), 7.27 (2H, d, *J* = 7.5 Hz), 7.04 (1H, d, *J* = 9.6 Hz), 3.04 (3H, s).

2-((2-methoxyphenyl)amino)-8-phenylpyrido[2,3-*d*]pyrimidin-7(8*H*)-one (S10)

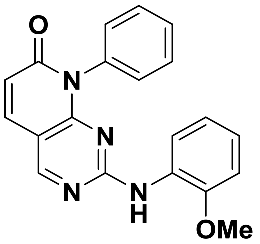

A solution of 2-(methylsulfonyl)-8-phenylpyrido[2,3-d]pyrimidin-7(8H)-one (79.0 mg, 0.261 mmol, 1.0 eq.) and o-anisidine (30.0 µL, 0.261 mmol, 1.0 eq.) in acetonitrile (1.3 mL, 0.2 M) was stirred at 85 °C overnight. The reaction mixture was concentrated under reduced pressure. The crude material was purified by flash column

chromatography eluting with ethyl acetate (20-100%) in 40-60 petroleum ether in yield the title compound as a white solid (38.3 mg, 0.111 mmol, 42%).

(ES, *m/z*): $[M+H]^+$ = 345.3

1H NMR (500 MHz, CDCl3) δ 8.52 (s, 1H), 7.96 (s, 1H), 7.60 – 7.48 (m, 5H), 7.29 – 7.23 (m, 2H), 6.81 (td, J = 8.1, 1.7 Hz, 1H), 6.73 (dd, J = 8.1, 1.5 Hz, 1H), 6.53 (d, J = 9.3 Hz, 1H), 6.43 (s, 1H), 3.79 (s, 3H).

13C NMR (126 MHz, CDCl3) δ 163.55, 158.40, 157.96, 157.06, 147.63, 136.55, 136.42, 129.72, 128.76, 128.53, 128.14, 122.25, 120.60, 118.99, 118.21, 109.64, 106.40, 55.71.

Compound S11

2-((2-Methoxyphenyl)amino)-8-phenyl-5-vinylpyrido[2,3-d]pyrimidin-7(8H)-one (S11)

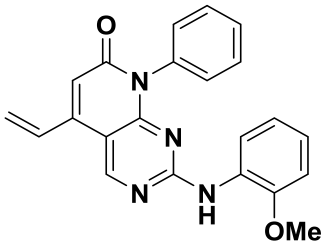

A solution of 5-ethynyl-2-((2-methoxyphenyl)amino)-8-phenylpyrido[2,3-d]pyrimidin-7(8H)-one (200 mg, 0.541 mmol, 1.0 eq.), Lindlar palladium catalyst (40.0 mg) in methanol (5.0 mL) was stirred at room temperature for 1 hour under a hydrogen balloon. The reaction mixture was filtered through a Celite pad and the filtrate was concentrated under reduced pressure. The reaction mixture was purified by reverse phase flash column chromatography eluting with acetonitrile (10-50%) in water (0.1% trifluoroacetic acid), then lyophilized to dryness to yield the title compound as an off-white solid (50.0 mg, 25%).

(ES, *m/z*): $[M+H]^+$ = 371

^{1}H NMR (500 MHz, MeOD) δ 8.84 (s, 1H), 7.52 (dddd, *J* = 8.8, 7.1, 5.0, 1.8 Hz, 4H), 7.35 (s, 1H), 7.25 – 7.18 (m, 2H), 7.13 (ddd, *J* = 17.2, 11.0, 0.9 Hz, 1H), 6.82 – 6.74 (m, 2H), 6.53 (d, *J* = 0.9 Hz, 1H), 6.37 – 6.31 (m, 1H), 6.00 (dd, *J* = 17.3, 1.0 Hz, 1H), 5.67 (dd, *J* = 11.0, 0.9 Hz, 1H), 3.77 (d, *J* = 1.1 Hz, 3H).

^{13}C NMR (126 MHz, MeOD) δ 164.16, 161.39, 156.91, 155.58, 146.63, 136.66, 129.48, 129.36, 128.67, 128.62, 128.33, 127.48, 122.29, 120.10, 122.47, 115.01, 112.91, 109.56, 105.46, 54.94.

Compound S12

2-(Methylthio)-8-phenyl-5-(prop-1-yn-1-yl)pyrido[2,3-d]pyrimidin-7(8H)-one

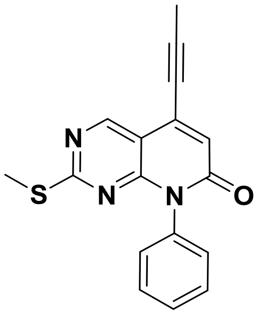

A solution of 2-(methylthio)-7-oxo-8-phenyl-7,8-dihydropyrido[2,3-d]pyrimidin-5-yl trifluoromethanesulfonate (500 mg, 1.20 mmol 1.0 eq.) and tributyl(prop-1-yn-1-yl)stannane (594 mg, 1.80 mmol 1.5 eq.) and tetrakis(triphenylphosphine)palladium (139 mg, 0.120 mmol 0.10 eq.) in dioxane (10 mL) was stirred at 100 °C overnight. The mixture was concentrated under reduced pressure. The crude product was purified by flash column chromatography to yield the title compound as a yellow solid (353 mg, 96%).

2-(Methylsulfonyl)-8-phenyl-5-(prop-1-yn-1-yl)pyrido[2,3-d]pyrimidin-7(8H)-one

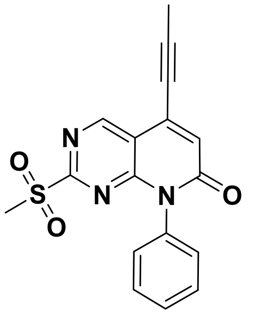

A solution of 2-(methylthio)-8-phenyl-5-(prop-1-yn-1-yl)pyrido[2,3-d]pyrimidin-7(8H)-one (353 g, 1.15 mmol, 1.0 eq.), m-CPBA (614 mg, 3.45 mmol, 3.0 eq.) in dichloromethane (10 mL) was stirred at room temperature for 1 hour. The reaction solution was washed by saturated aqueous sodium hydrogen carbonate and saturated aqueous sodium thiosulfate, dried (Na_2SO_4) and concentrated under reduced pressure. The crude product was purified by flash column chromatography to yield the title compound as a yellow solid (319 mg, 82%).

2-((2-Methoxyphenyl)amino)- 8-phenyl-5-(prop-1-yn-1-yl)pyrido[2,3-d]pyrimidin-7(8H)-one (S12)

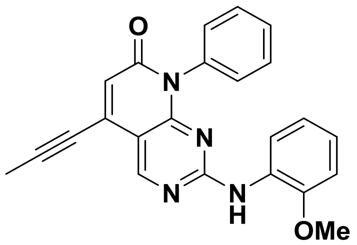

A solution of 2-(methylsulfonyl)-8-phenyl-5-(prop-1-yn-1-yl)pyrido[2,3-d]pyrimidin-7(8H)-one (319 mg, 0.941 mmol, 1.0 eq.), 2-methoxyaniline (116 mg, 0.941 mmol, 1.0 eq.) and trifluoroacetic acid (91.0 mg, 0.941 mmol, 1.0 eq.) in 2-butanol (5.0 mL) was stirred at 110 ^{o}C overnight. The reaction mixture was cooled to room temperature and purified by reverse phase flash column chromatography directly eluting with acetonitrile (10-100%) in water (0.1% ammonium carbonate) to yield the title compound as an off white solid (50.0 mg, 14%).

(ES, *m/z*): $[M+H]^+$ = 383

^{1}H NMR (500 MHz, $CDCl_3$) δ 8.89 (s, 1H), 8.06 (s, 1H), 7.67 – 7.55 (m, 3H), 7.52 (s, 1H), 7.43 – 7.33 (m, 2H), 6.94 – 6.87 (m, 1H), 6.82 (dd, *J* = 8.1, 1.5 Hz, 1H), 6.65 (s, 1H), 6.51 (s, 1H), 3.88 (s, 3H), 2.26 (s, 3H).

^{13}C NMR (126 MHz, $CDCl_3$) δ 162.97, 158.59, 157.64, 156.75, 147.62, 136.44, 131.91, 129.69, 128.79, 128.51, 128.15, 122.23, 121.19, 120.59, 118.18, 109.62, 106.39, 98.16, 73.40, 55.69, 4.91.

Compound S13

2-((4-((2-(dimethylamino)ethyl)(methyl)amino)phenyl)amino)-8-phenylpyrido[2,3-d]pyrimidin-7(8H)-one (S13)

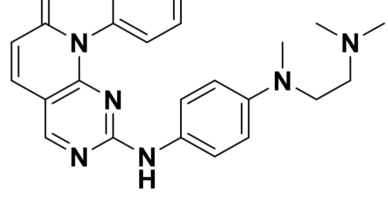

A solution of 2-(methylsulfonyl)-8-phenylpyrido[2,3-d]pyrimidin-7(8H)-one (200 mg, 0.664 mmol, 1.0 eq.), N^1-(2-(dimethylamino)ethyl)-N^1-methylbenzene-1,4-diamine (167 mg, 0.863 mmol, 1.3 eq.) and trifluoroacetic acid

(66.0 µL, 0.863 mmol, 1.3 eq.) in acetonitrile (6.6 mL, 0.1 M) was stirred at 80 °C overnight. The reaction mixture was concentrated under reduced pressure. The crude material was purified by flash column chromatography eluting with methanol (0-3%) in dichloromethane to yield the title compound (176 mg, 0.425 µmol, 64%).

(ES, *m/z*): $[M+H]^+$ = 415.3

^{1}H NMR (500 MHz, MeOD) δ 8.55 (s, 1H), 7.77 (d, *J* = 9.5 Hz, 1H), 7.51 (d, *J* = 7.9 Hz, 3H), 7.20 (dd, *J* = 7.4, 2.0 Hz, 2H), 7.06 – 6.95 (m, 2H), 6.42-6.25 (m, 2H), 6.36 (d, *J* = 9.5 Hz, 1H), 3.31 (t, *J* = 7.2 Hz, 2H), 2.76 (s, 3H), 2.51 (t, *J* = 7.6 Hz, 2H), 2.32 (s, 6H).

^{13}C NMR (126 MHz, MeOD) δ 164.56, 158.59, 156.71, 149.01, 137.98, 136.77, 129.24, 129.20, 128.72, 128.09, 119.72, 116.40, 114.26, 112.79, 106.01, 54.51, 48.83, 43.15, 38.60.

Protein Mass Spectrometry

Scaffold A

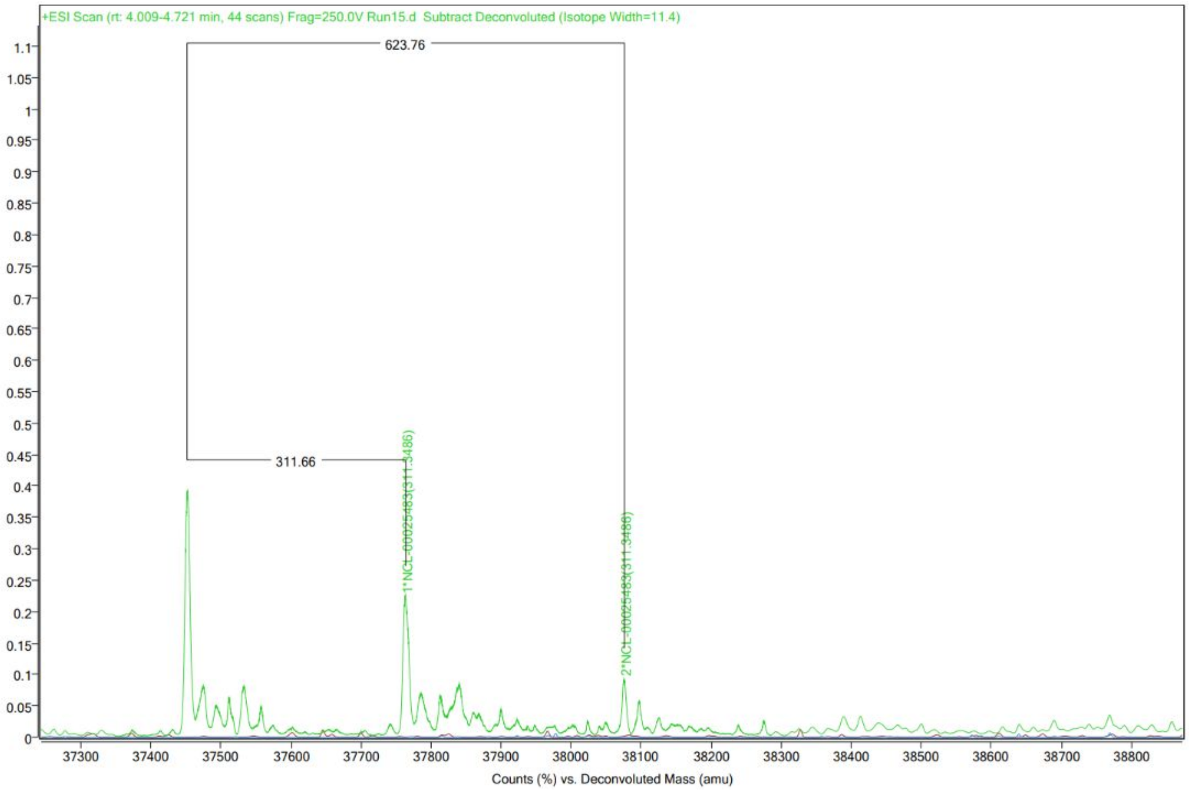

*Figure S4: Protein mass spectrum for **2***

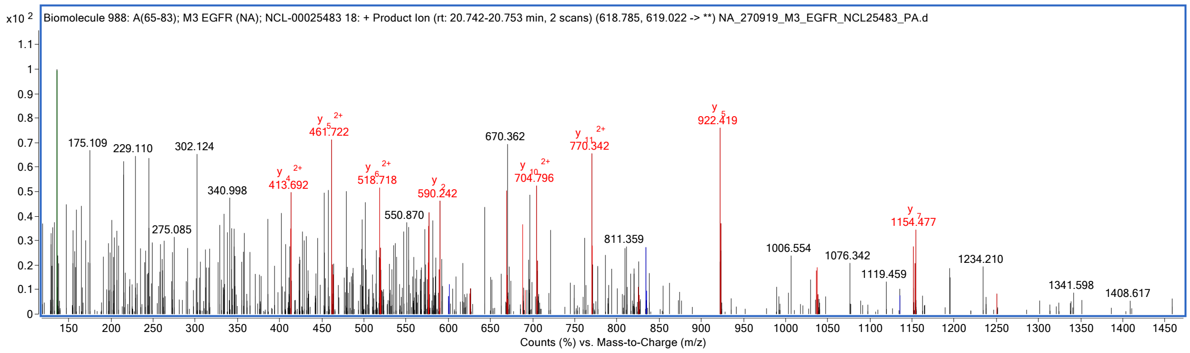

*Figure S5: Pepsin digest for EGFR-**2** adduct demonstrates C775 modification - EILDEAYVMASVDNPHVCR*

Scaffold B

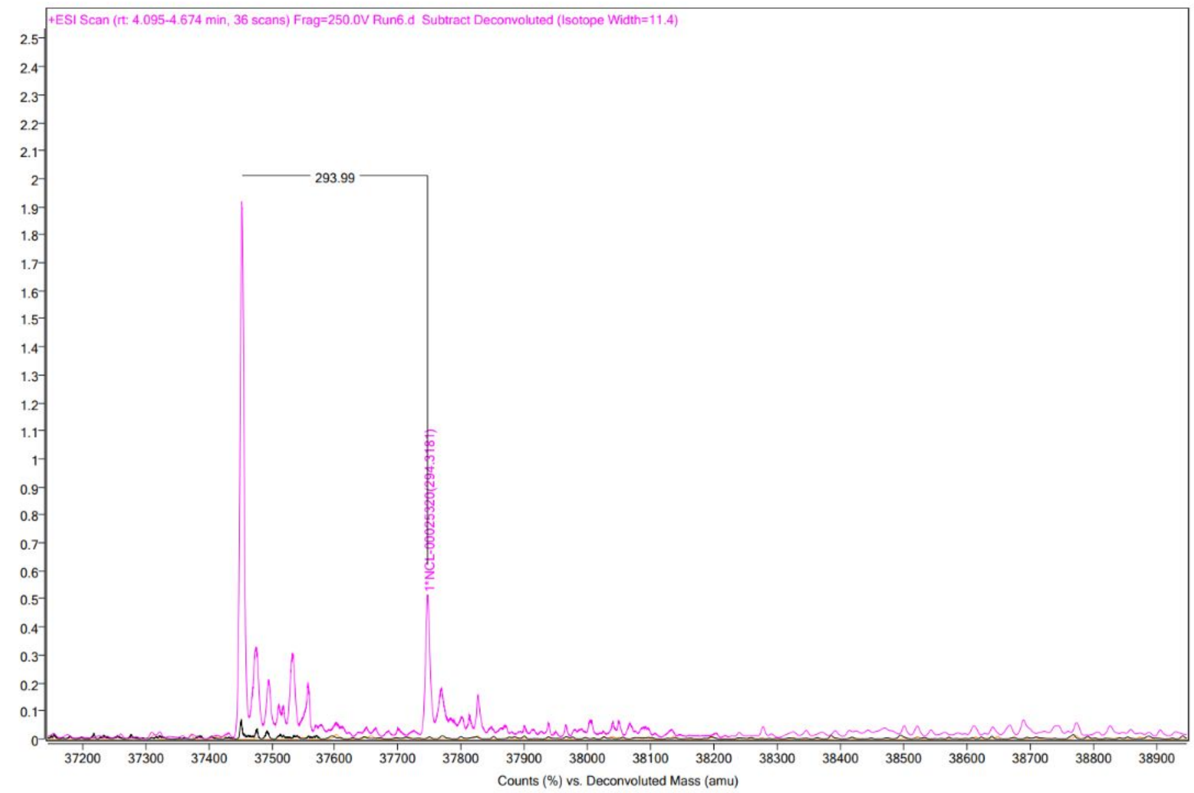

*Figure S6: Protein mass spectrum for **S1***

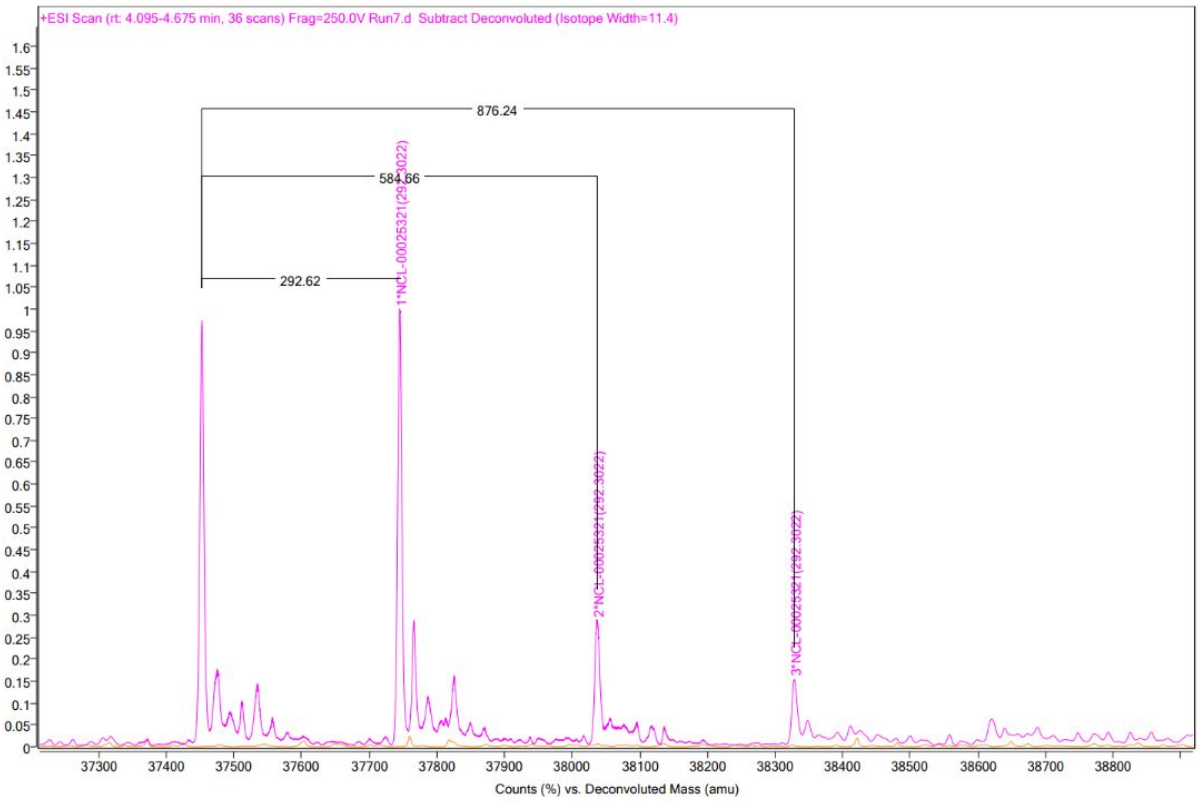

*Figure S7: Protein mass spectrum for **S2***

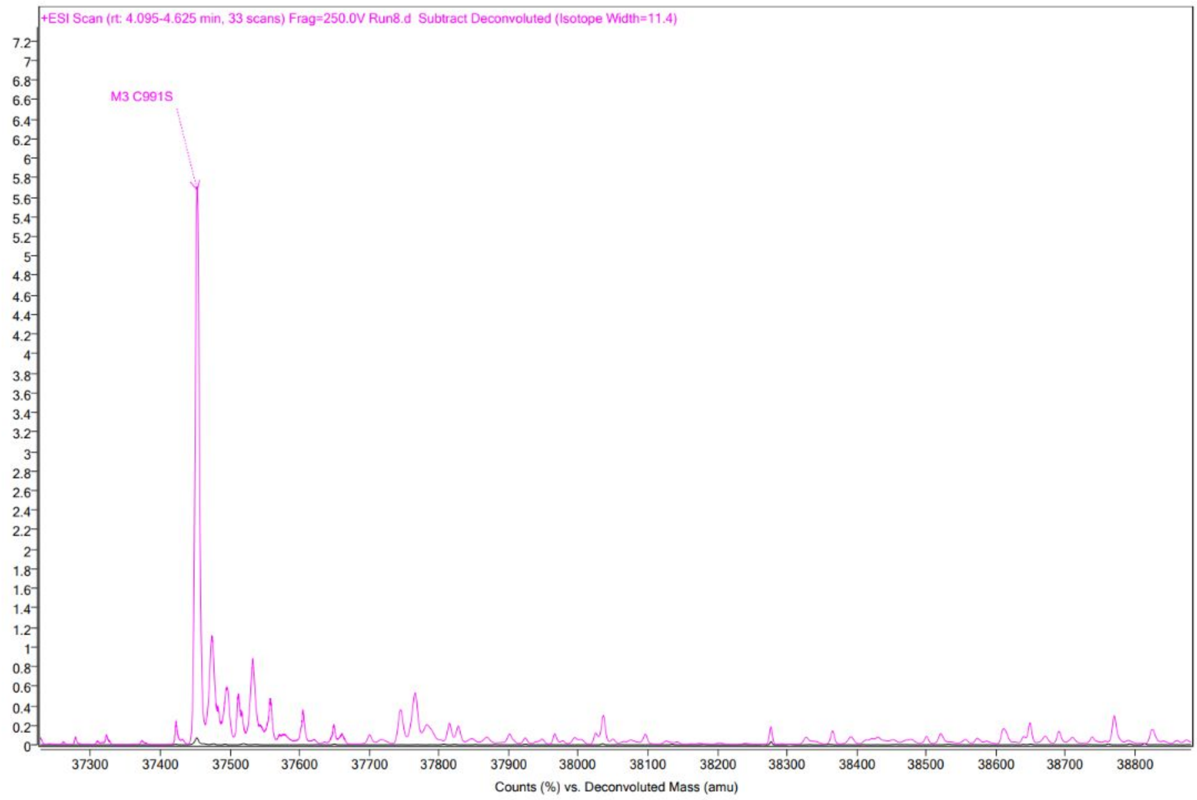

Figure S8: Protein mass spectrum for ***S3***

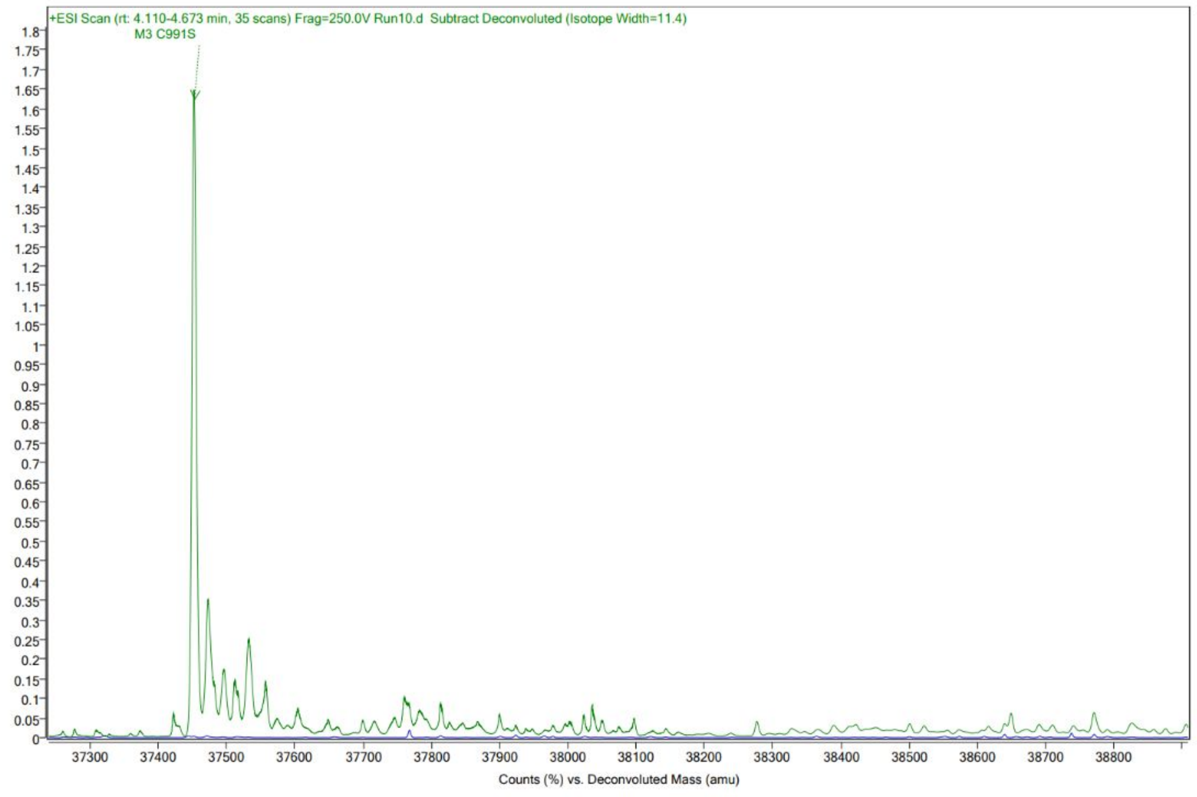

Figure S9: Protein mass spectrum for ***S7***

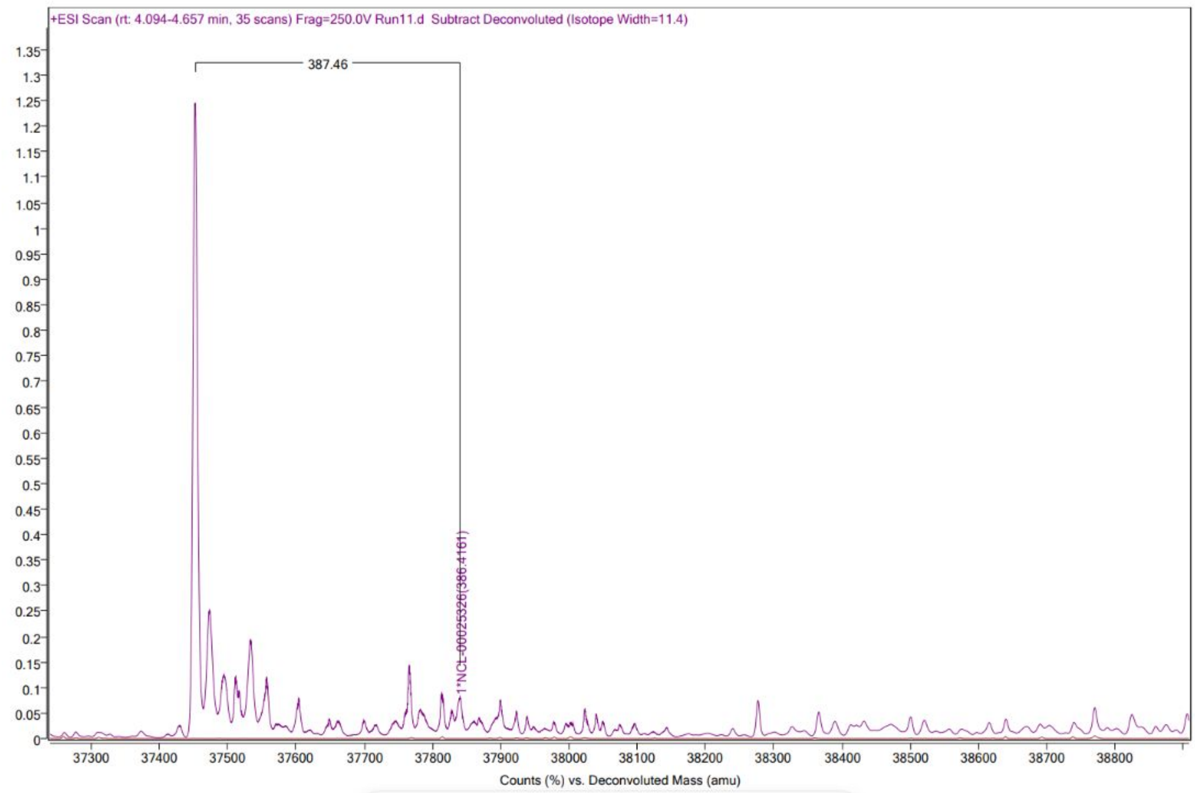

*Figure S90: Protein mass spectrum for **S8***

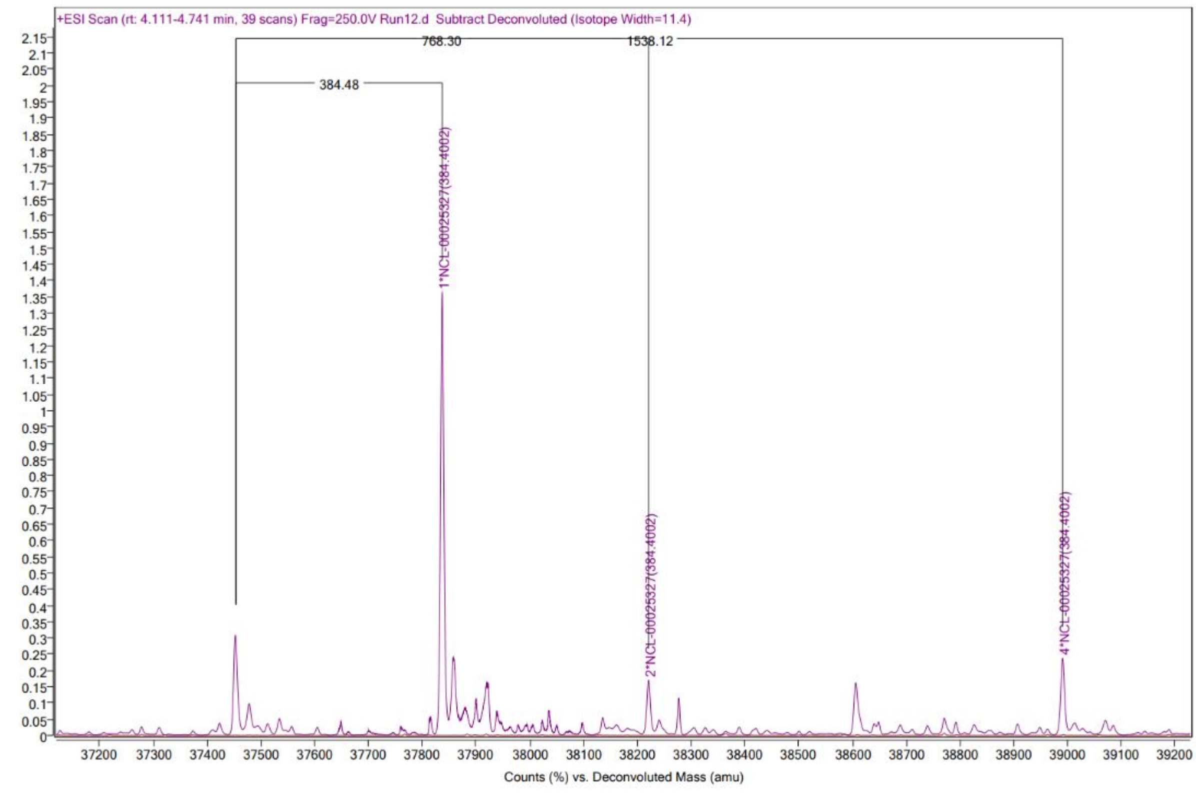

*Figure S101: Protein mass spectrum for **S9***

Scaffold C

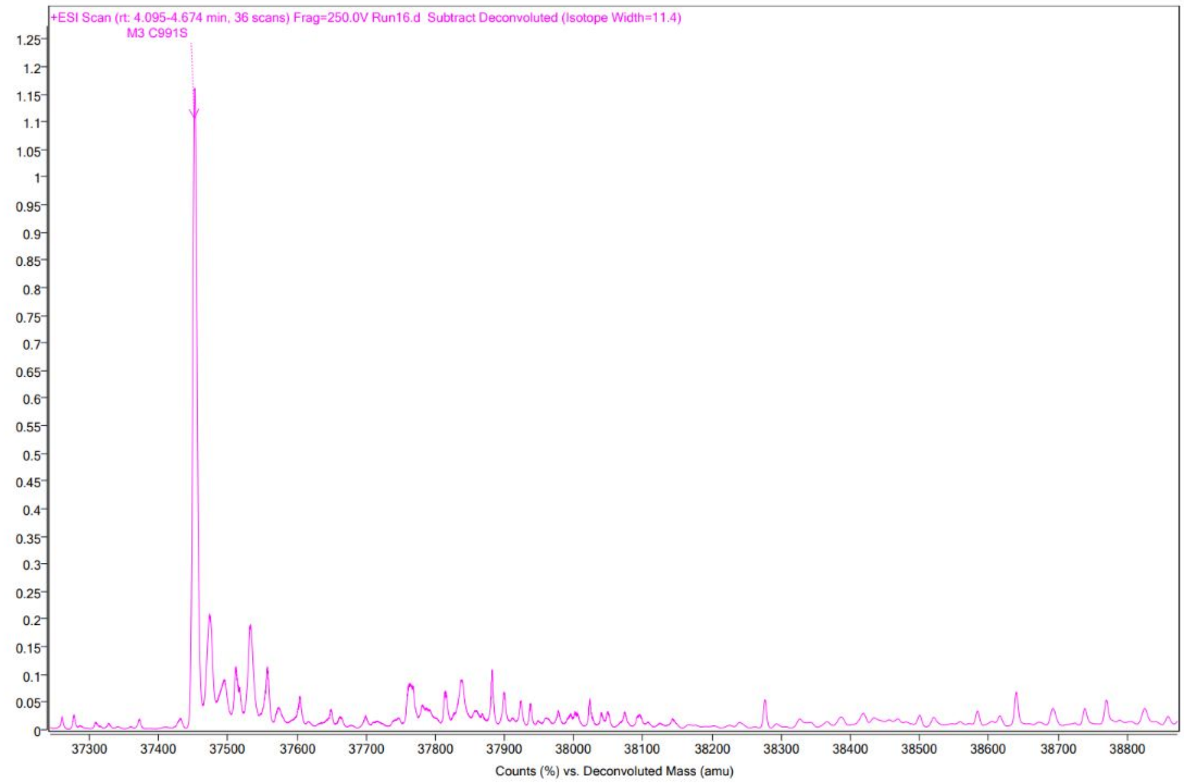

*Figure S12: Protein mass spectrum for **S4***

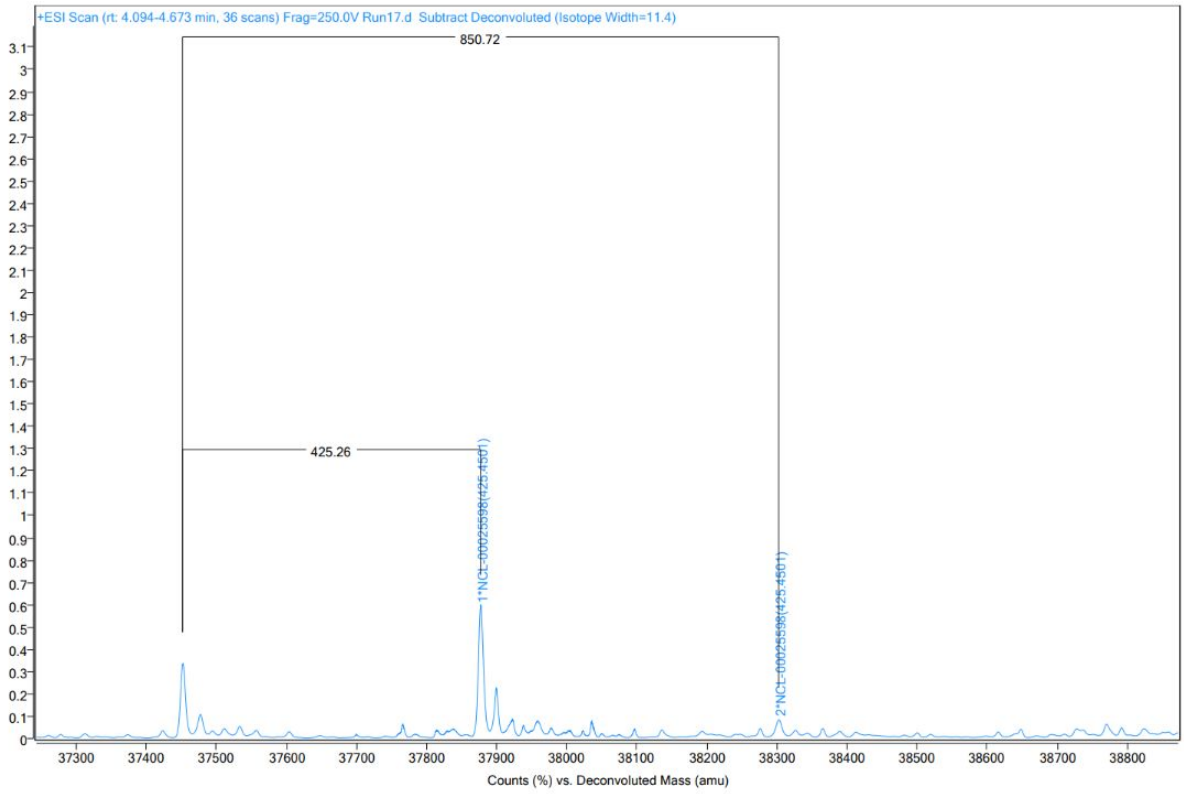

*Figure S13: Protein mass spectrum for **S5***

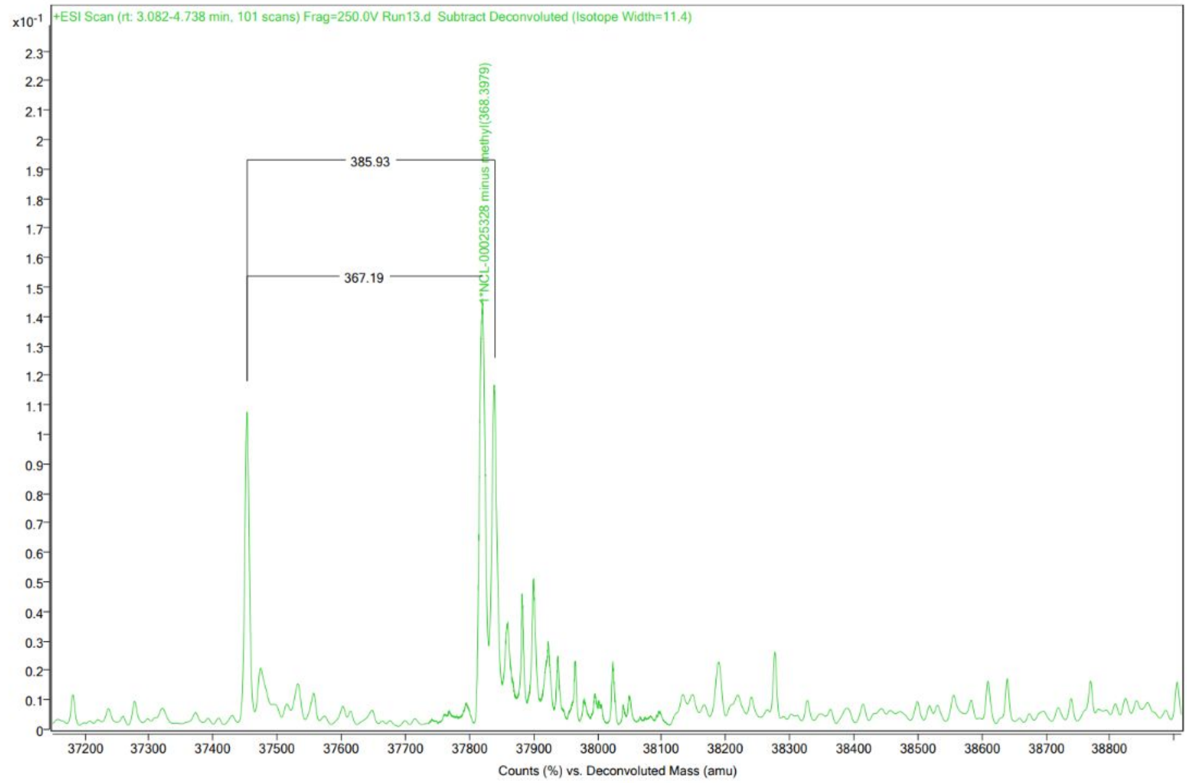

*Figure S114: Protein mass spectrum for **3***

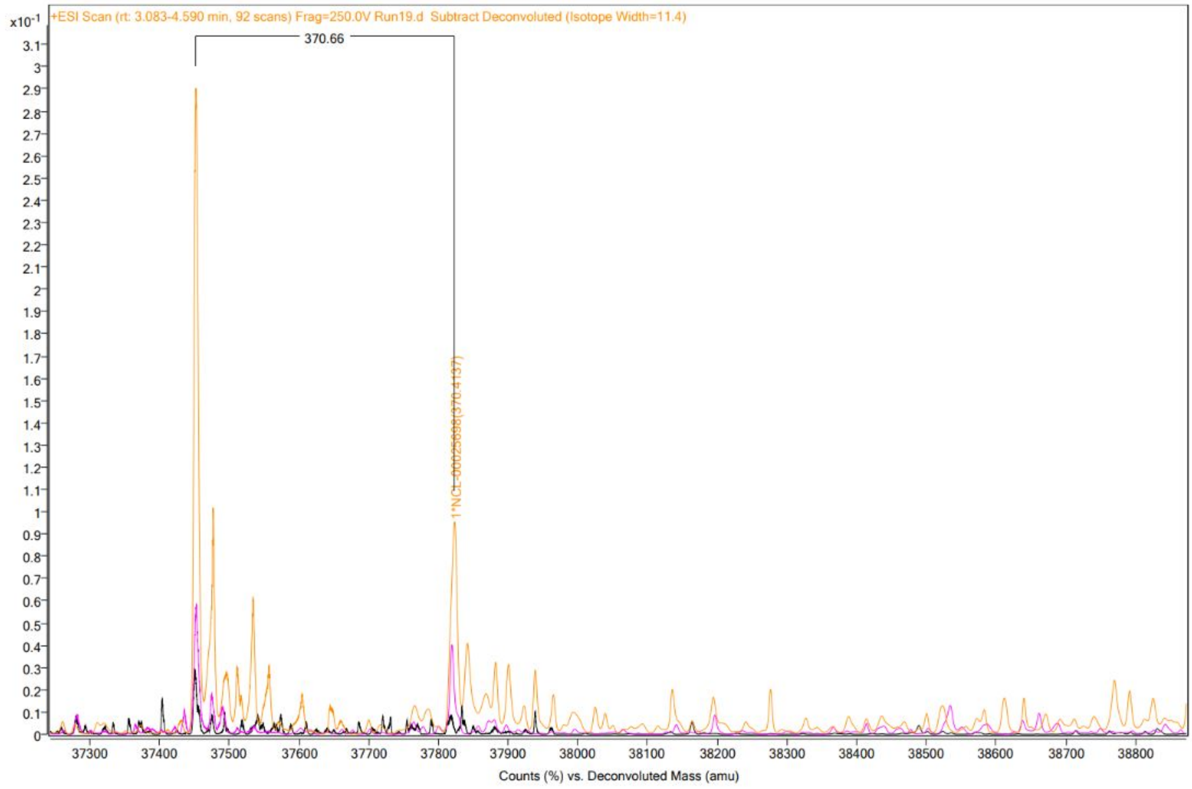

*Figure S15: Protein mass spectrum for **S11***

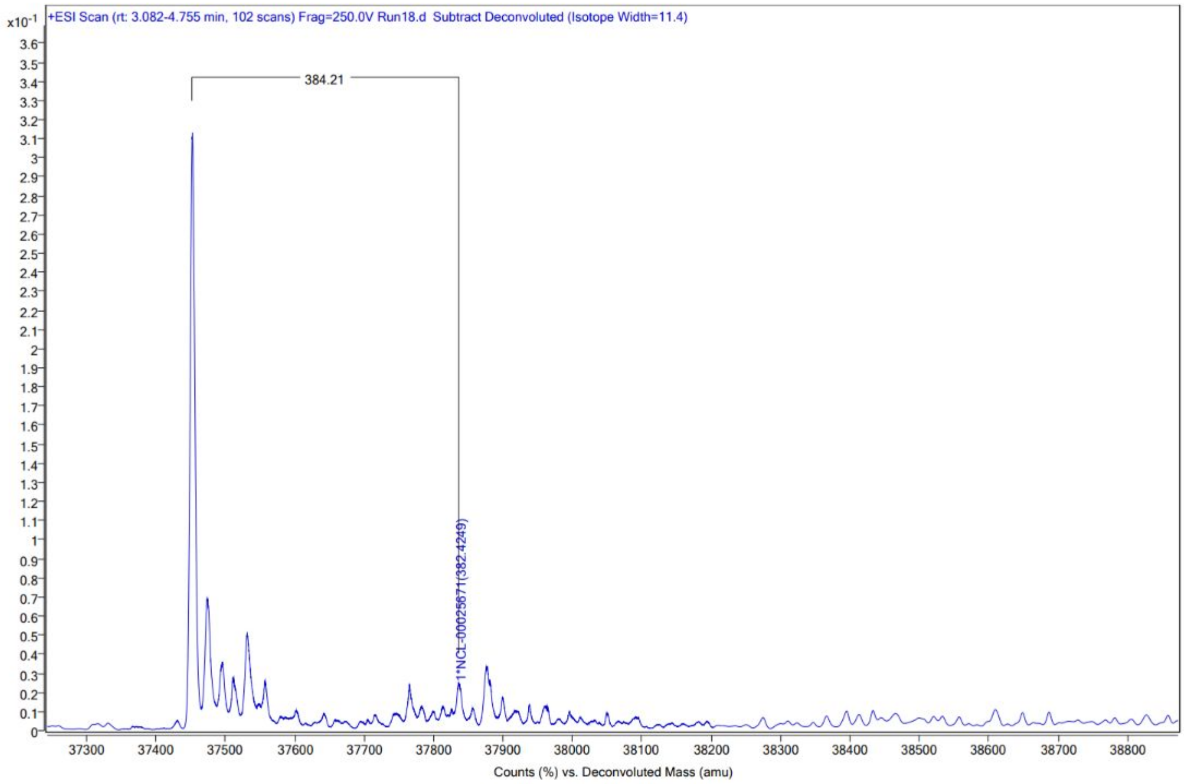

*Figure S16: Protein mass spectrum for **S12***

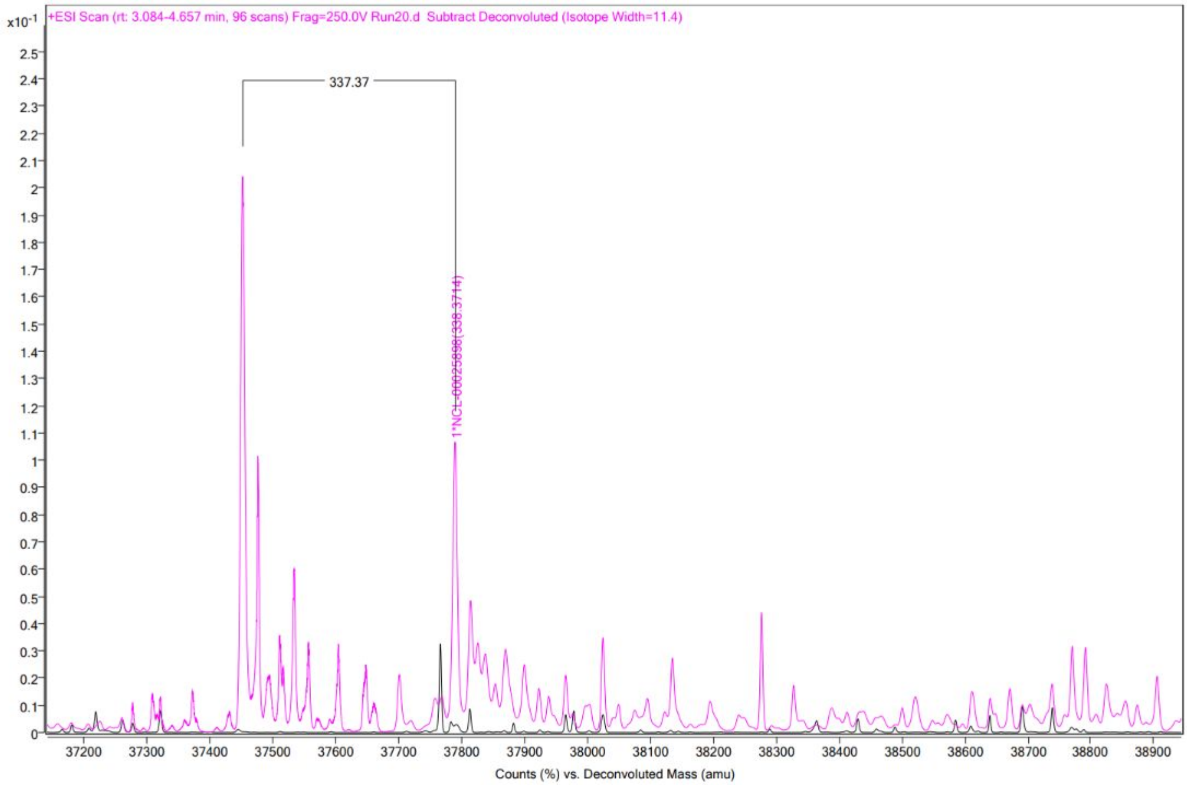

*Figure S17: Protein mass spectrum for **4***

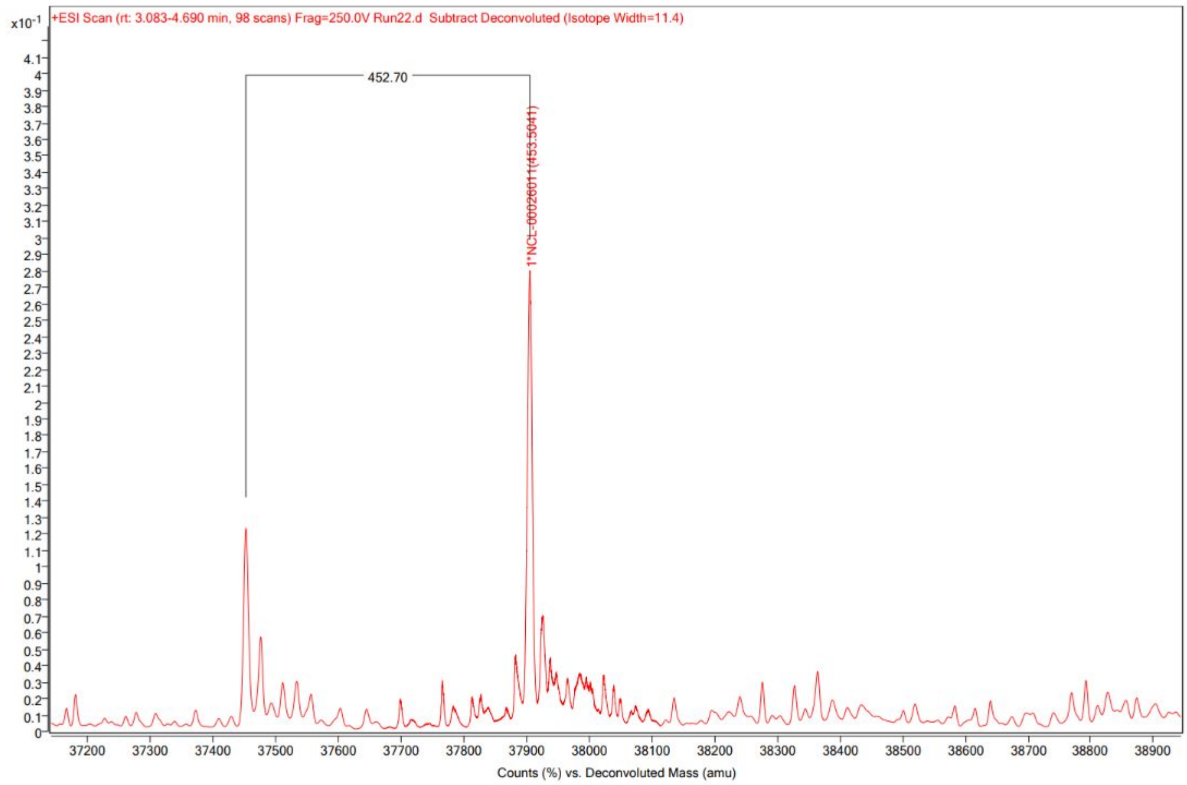

*Figure S18: Protein mass spectrum for **5***

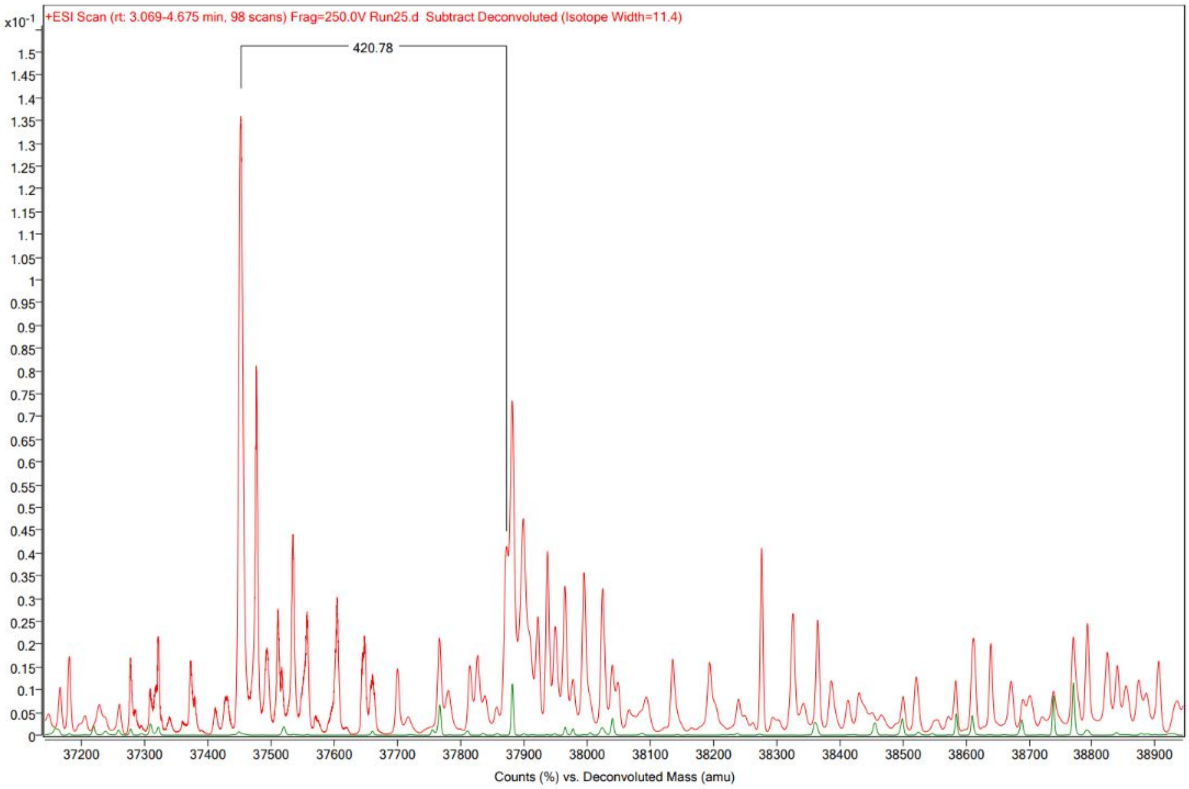

*Figure S19: Protein mass spectrum for **6***

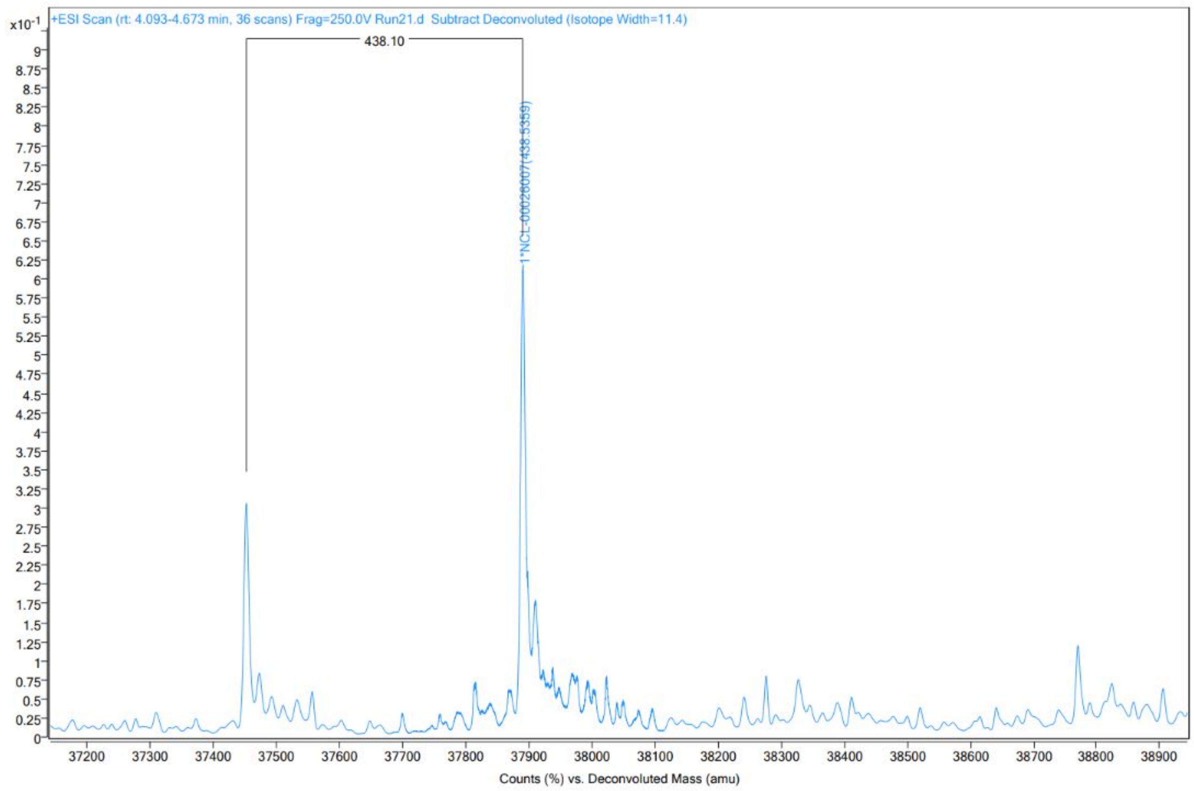

Figure S20: Protein mass spectrum for ***7***

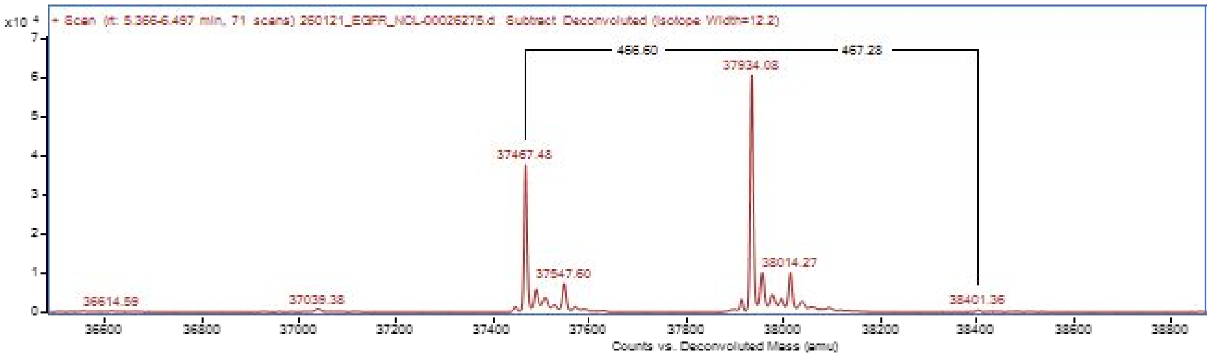

Figure S2112: Protein mass spectrum for ***8***

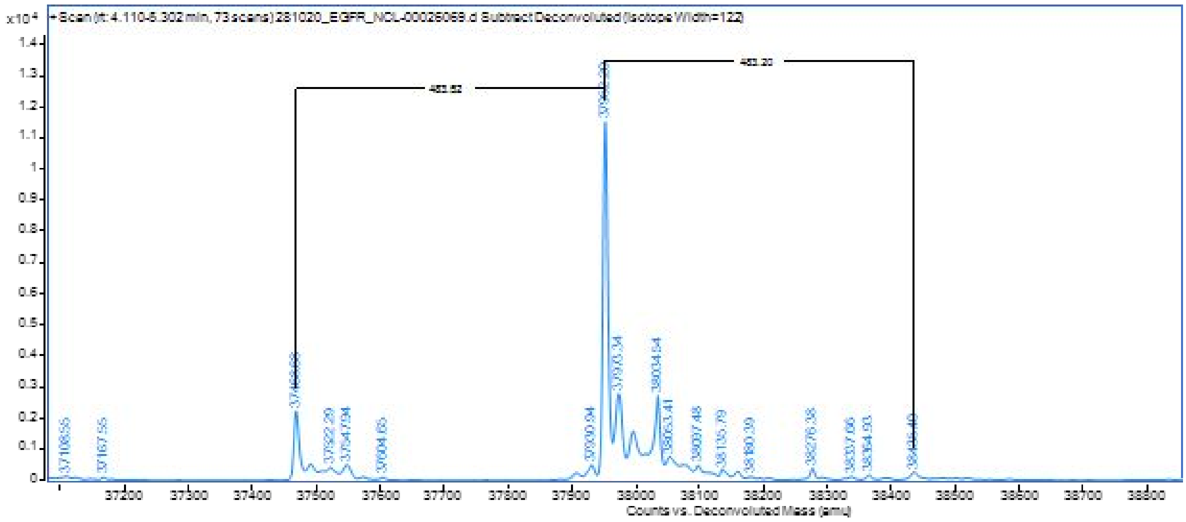

*Figure S22: Protein mass spectrum for **10***

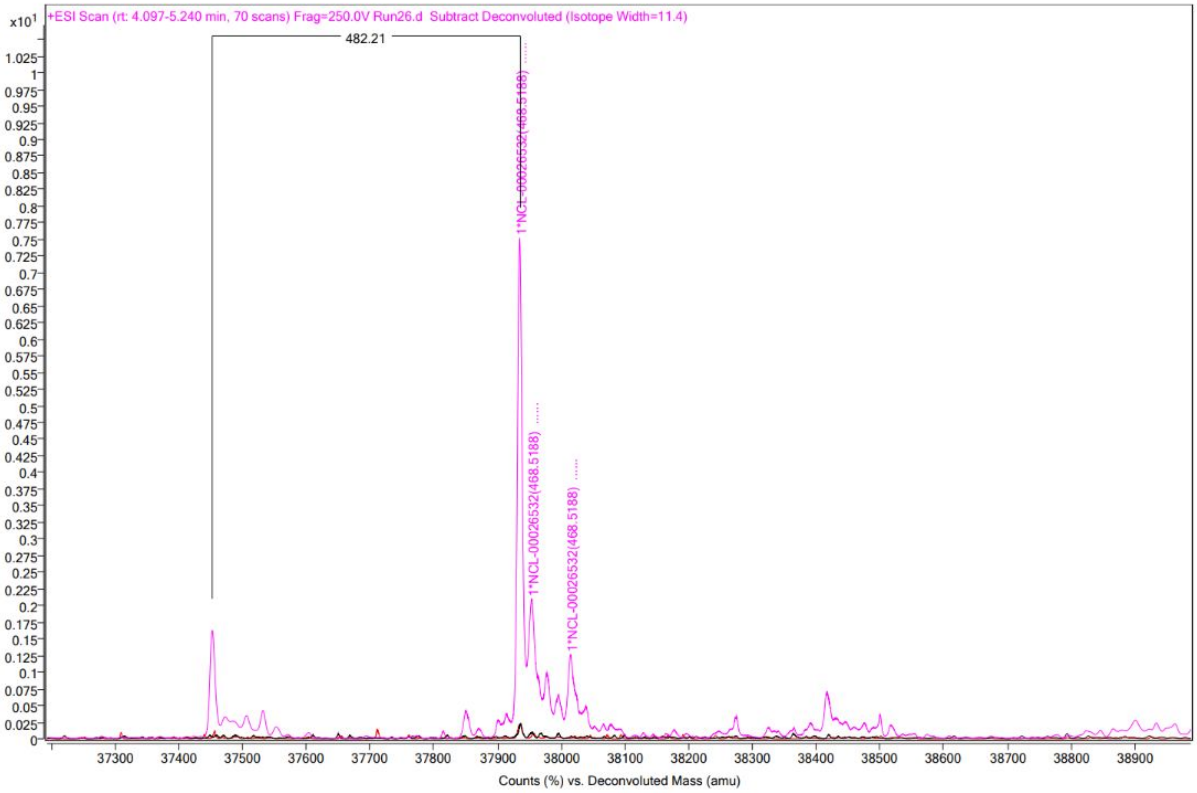

*Figure S23: Protein mass spectrum for **11***

HPLC Spectra

Scaffold A

4-ethynyl-*N*-phenyl-6-(pyrazolo[1,5-a]pyridin-3-yl)pyrimidin-2-amine (2)

Acidic – 100%

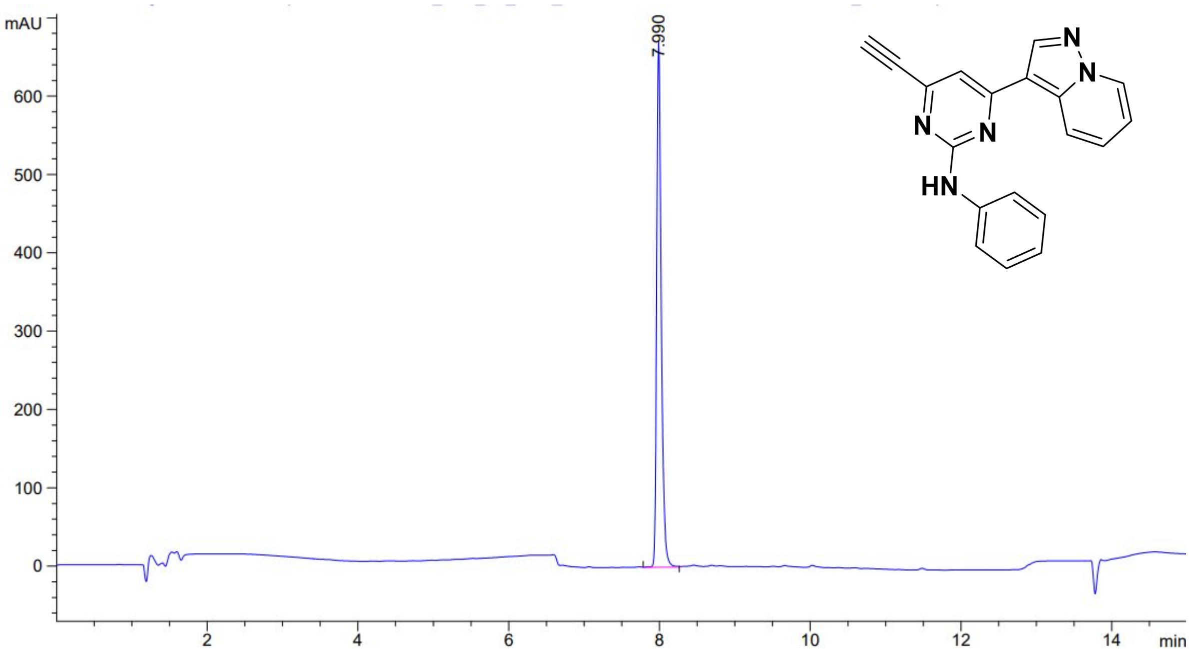

Basic – 100%

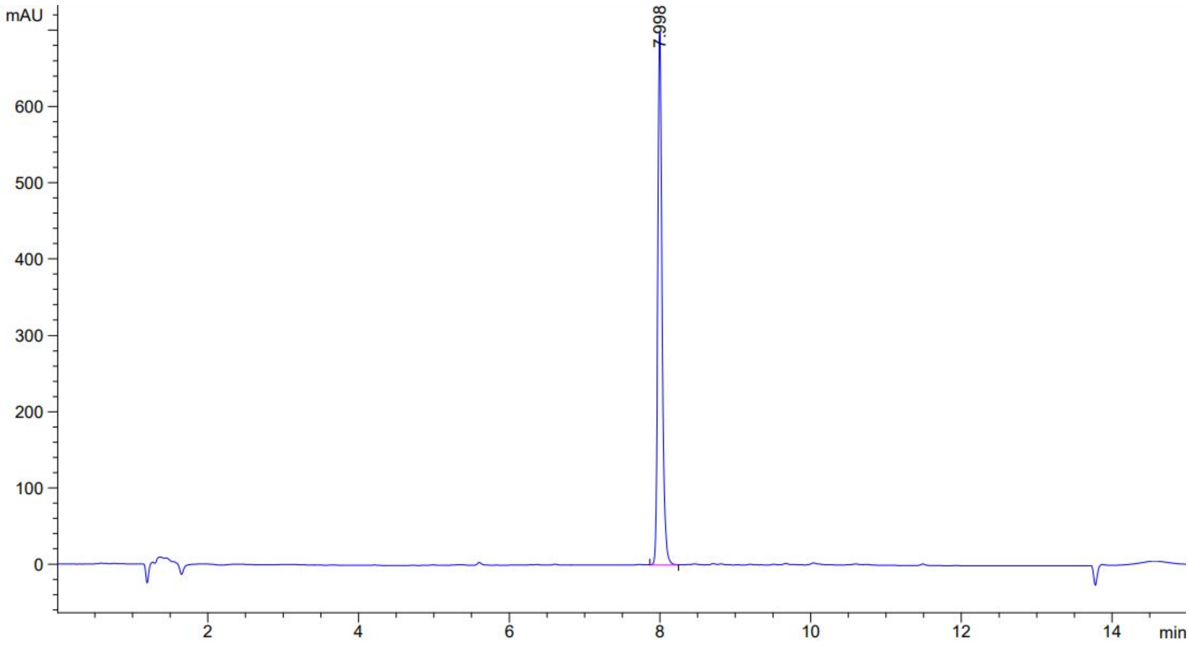

Scaffold B

N-(3-{4-Amino-1-methylpyrazolo[3,4-d]pyrimidin-3-yl}phenyl)prop-2-enamide S1

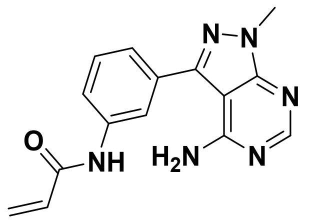

Acidic – 99.6%

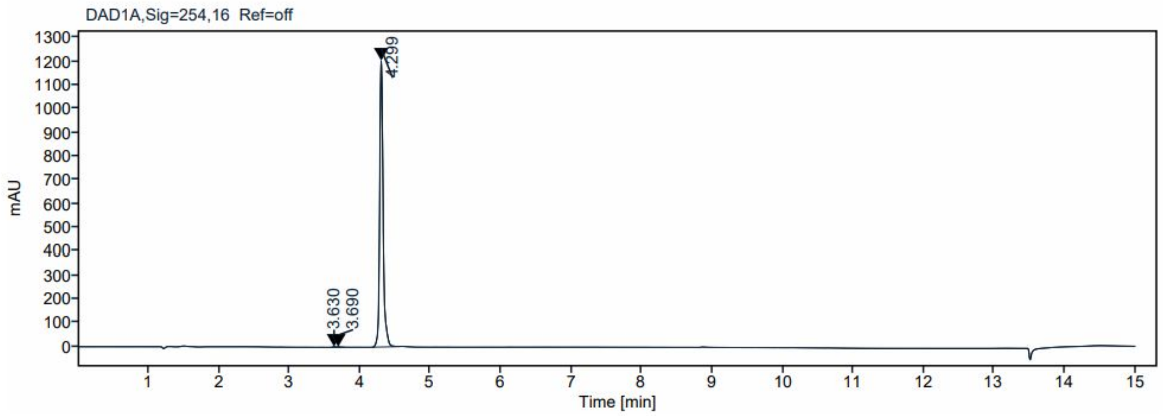

Basic – 99.8%

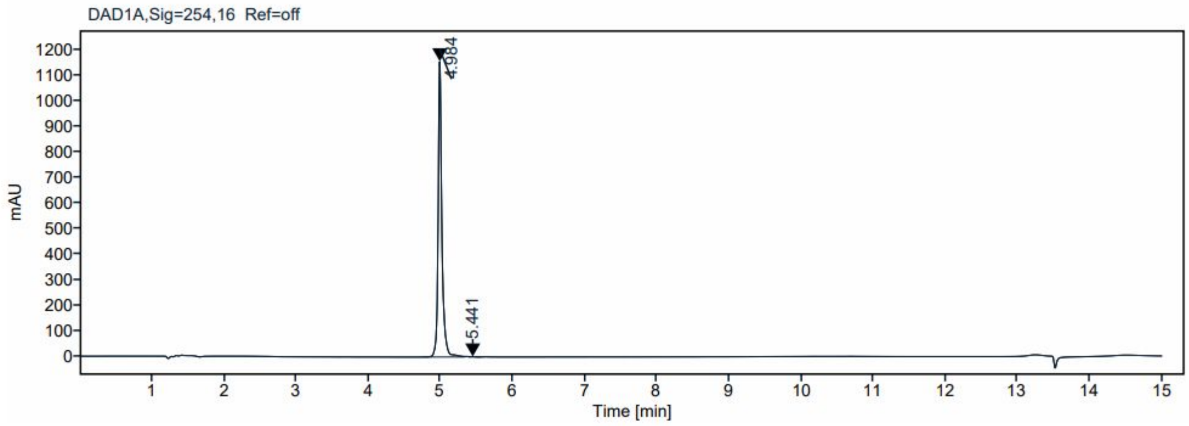

N-(3-{4-amino-1-methylpyrazolo[3,4-d]pyrimidin-3-yl}phenyl)prop-2-ynamide (S2)

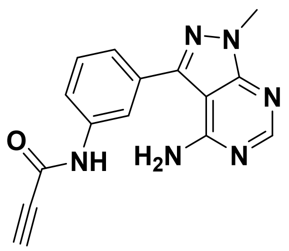

Acidic – 94.5%

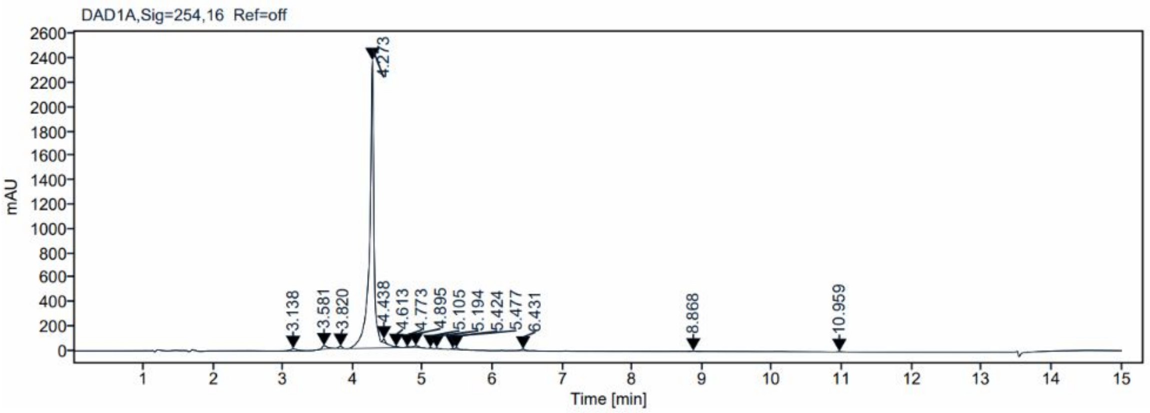

Basic – 95.4%

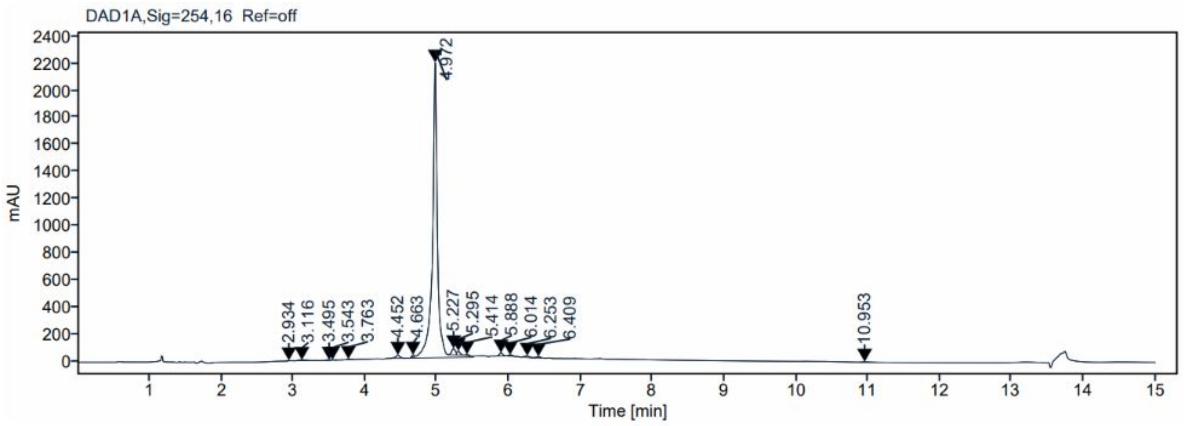

N-[(3-{4-Amino-1-methylpyrazolo[3,4-d]pyrimidin-3-yl}phenyl)methyl]prop-2-enamide (S3)

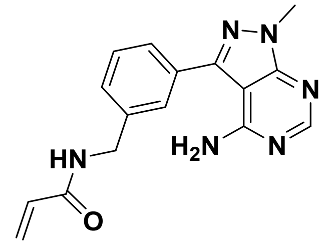

Acidic – 96.7%

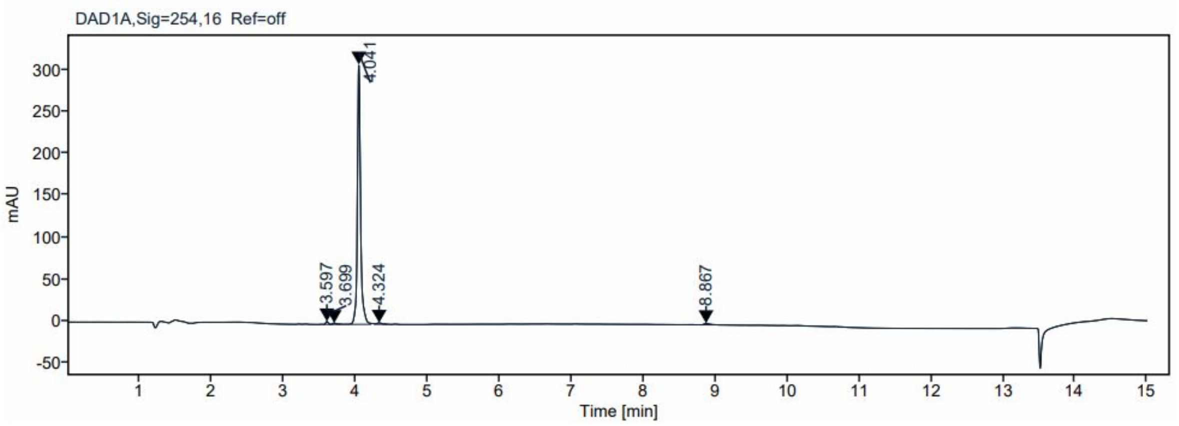

Basic – 95.0%

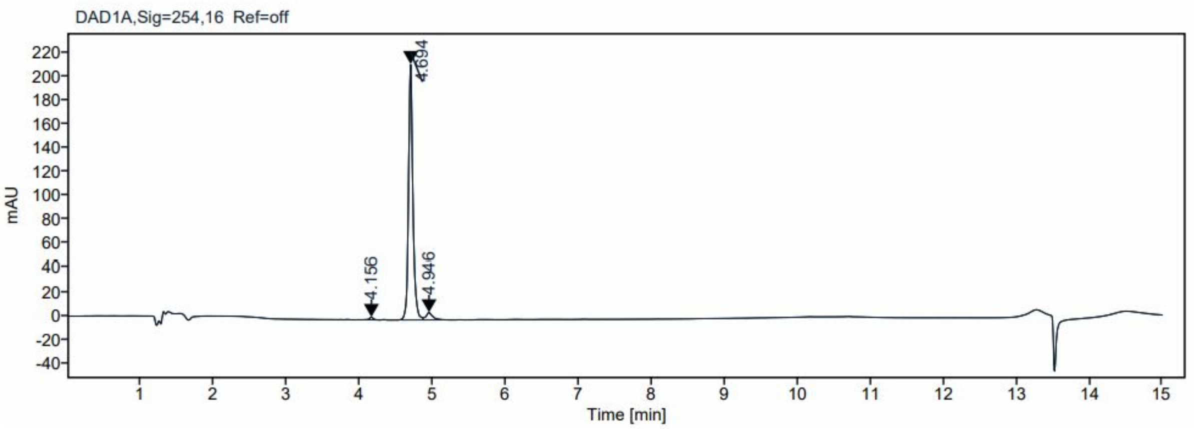

N-[2-(3-{4-Amino-1-methylpyrazolo[3,4-d]pyrimidin-3-yl}phenoxy)ethyl]prop-2-enamide (S6)

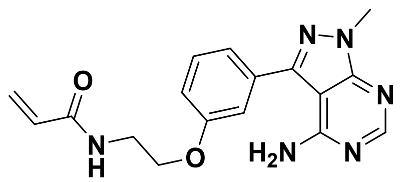

Acidic – 100%

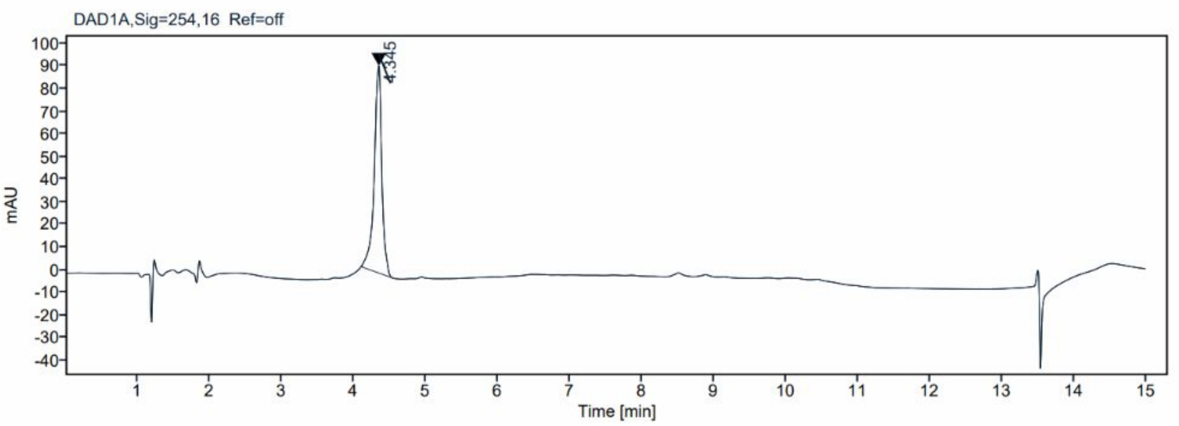

Basic – 100%

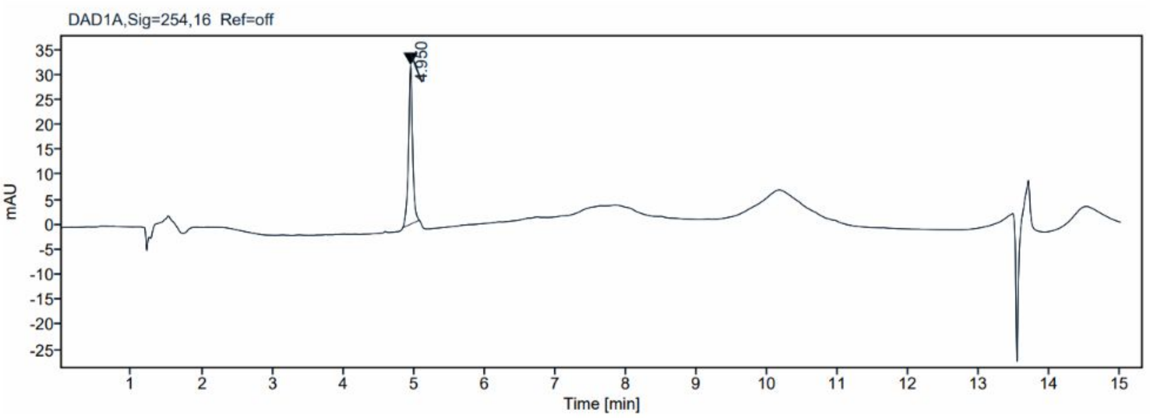

***N*-(2-(4-(4-amino-1-methyl-1*H*-pyrazolo[3,4-*d*]pyrimidin-3-yl)phenoxy)ethyl)acrylamide (S7)**

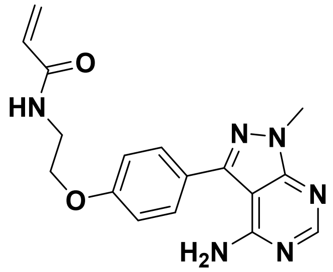

Acidic – 100%

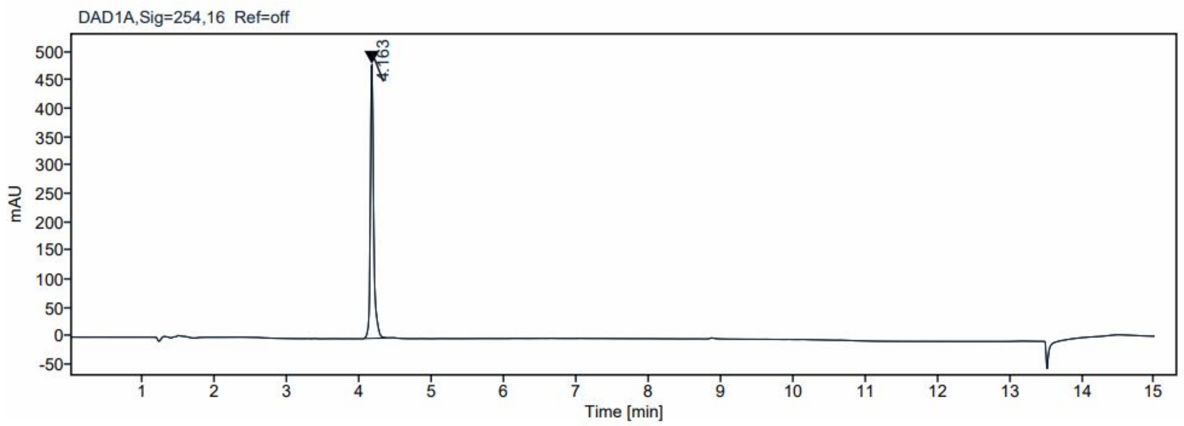

Basic – 98.6

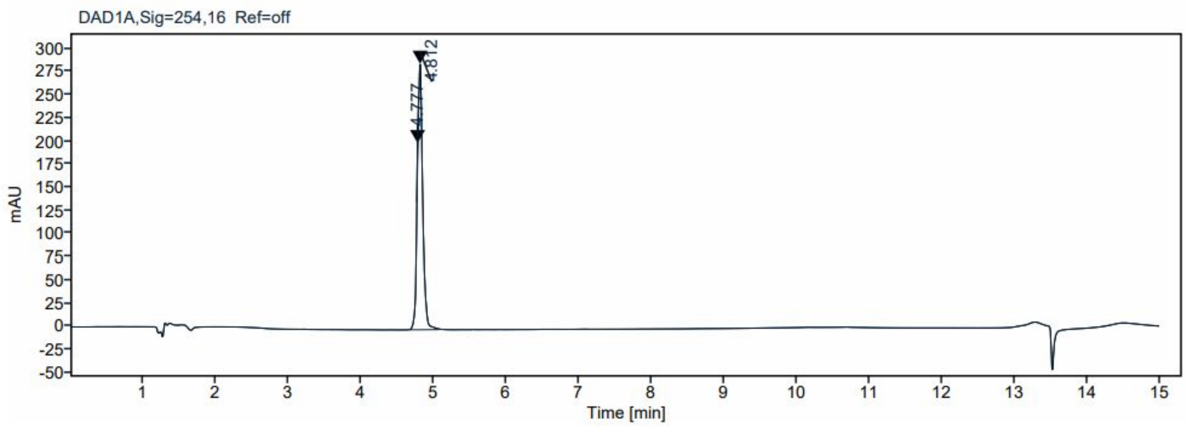

N-[2-(4-{4-Amino-1-methylpyrazolo[3,4-d]pyrimidin-3-yl}phenoxy)phenyl]prop-2-enamide (S8)

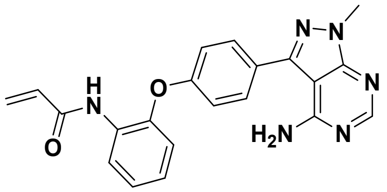

Acidic – 99.3%

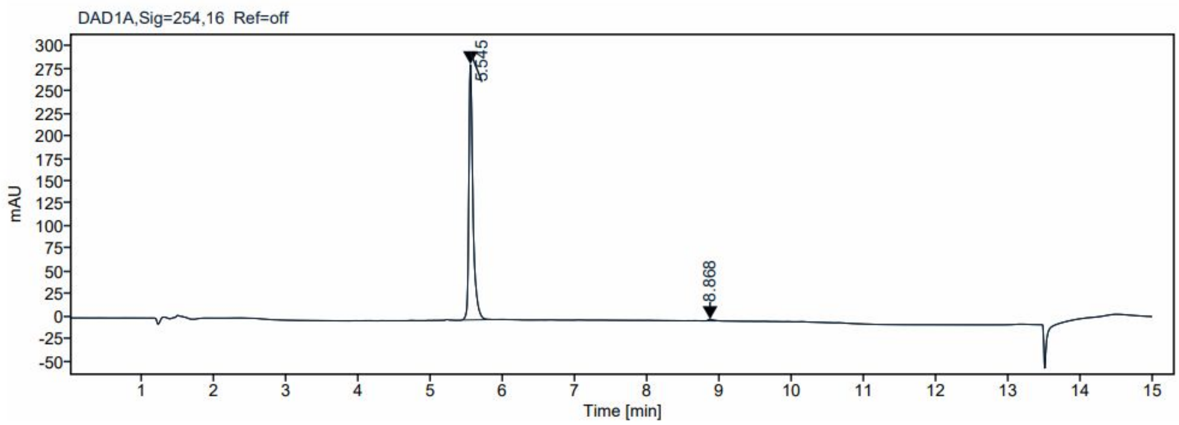

Basic – 98.3%

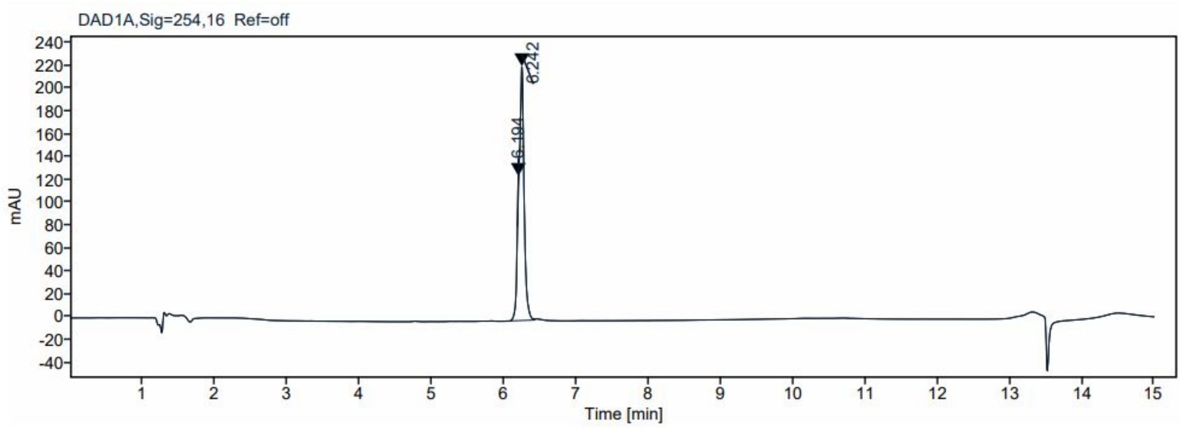

N-[2-(4-{4-Amino-1-methylpyrazolo[3,4-d]pyrimidin-3-yl}phenoxy)phenyl]prop-2-ynamide (S9)

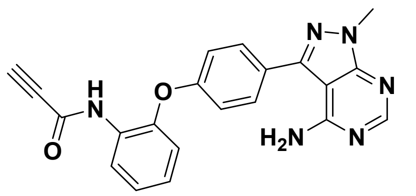

Acidic – 99.0%

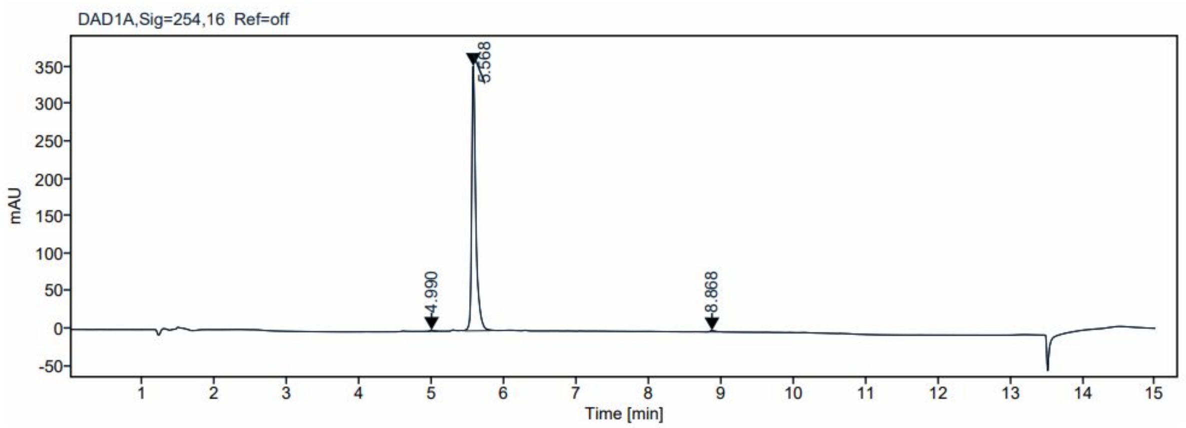

Basic – 99.4%

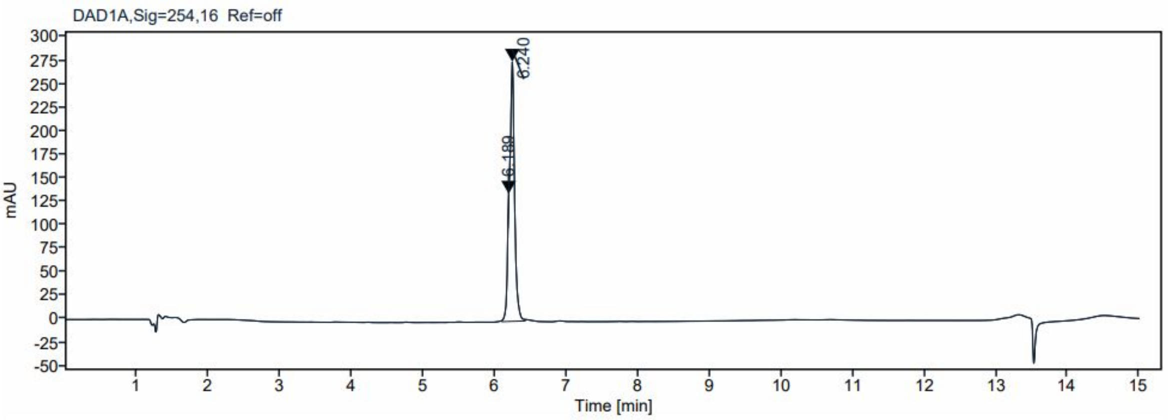

Scaffold C

N-(3-(2-((2-Methoxyphenyl)amino)-7-oxo-8-phenyl-7,8-dihydropyrido [2,3-d]pyrimidin-5-yl)benzyl)acrylamide (S4)

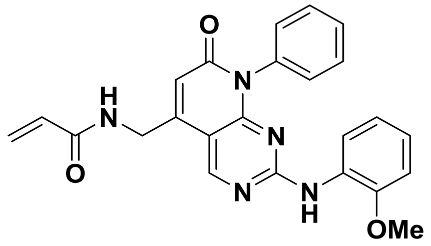

Acidic – 99.1%

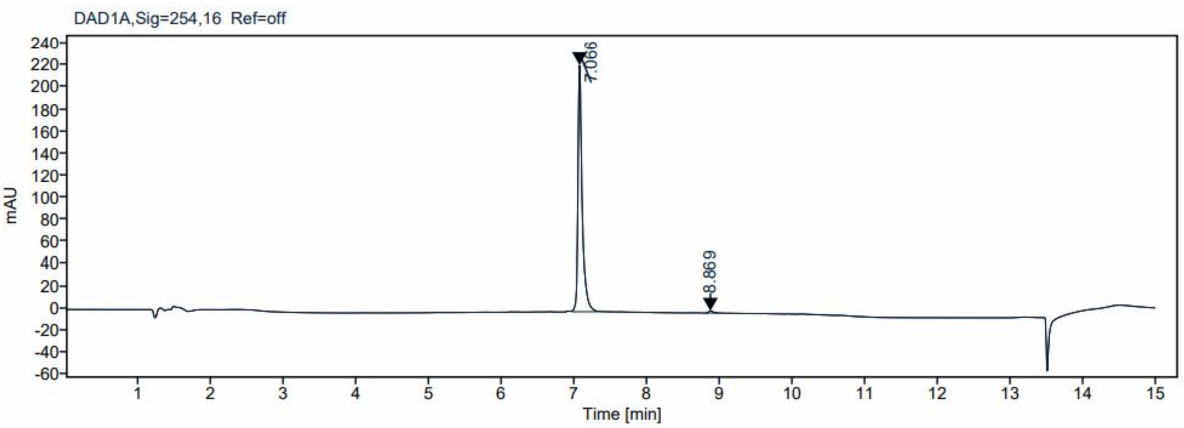

Basic – 98.1%

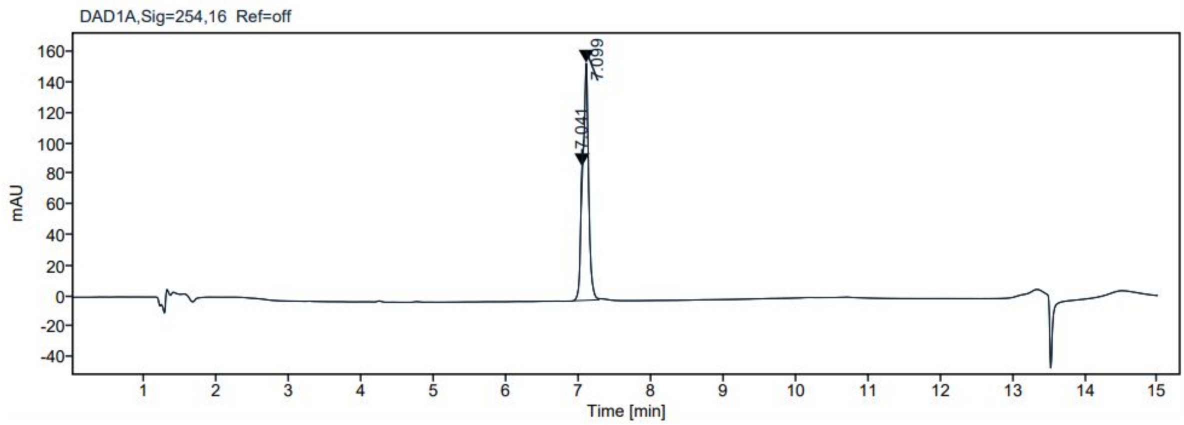

N-((2-((2-Methoxyphenyl)amino)-7-oxo-8-phenyl-7,8-dihydropyrido[2,3-d]pyrimidin-5-yl)methyl)propiolamide (S5)

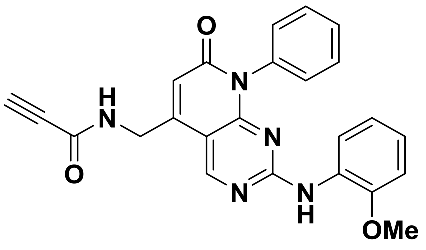

Acidic – 97.2%

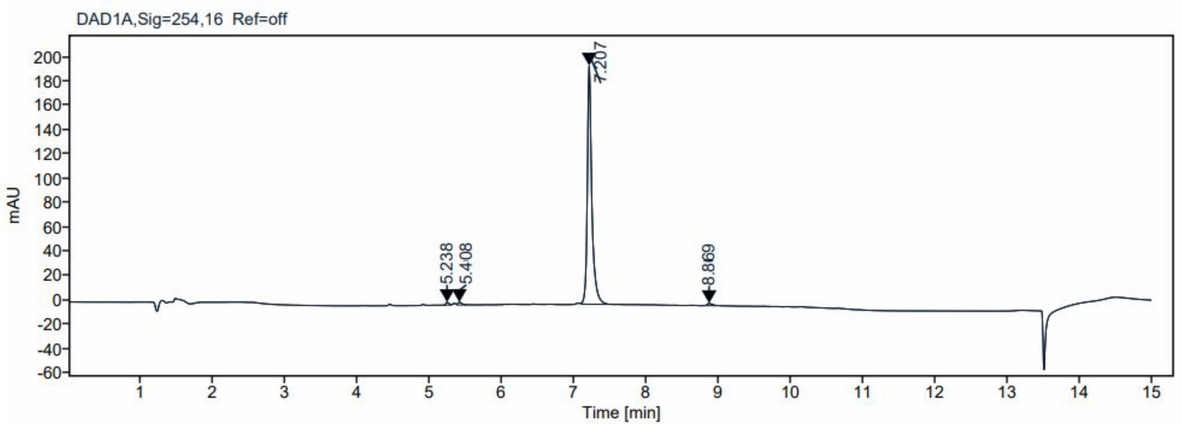

Basic – 98.1%

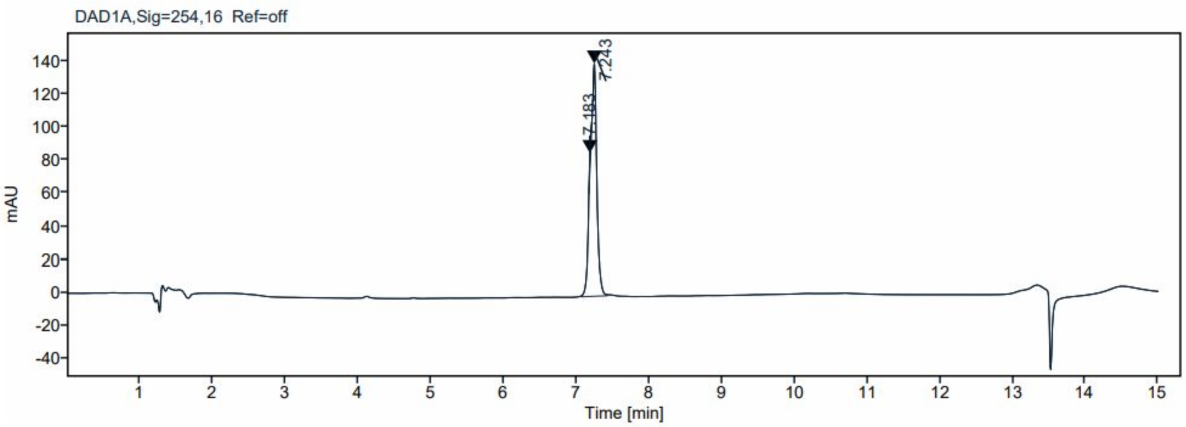

5-Ethynyl-2-[(2-methoxyphenyl)amino]-8-phenylpyrido[2,3-*d*]pyrimidin-7-one (3)

Acidic – 100%

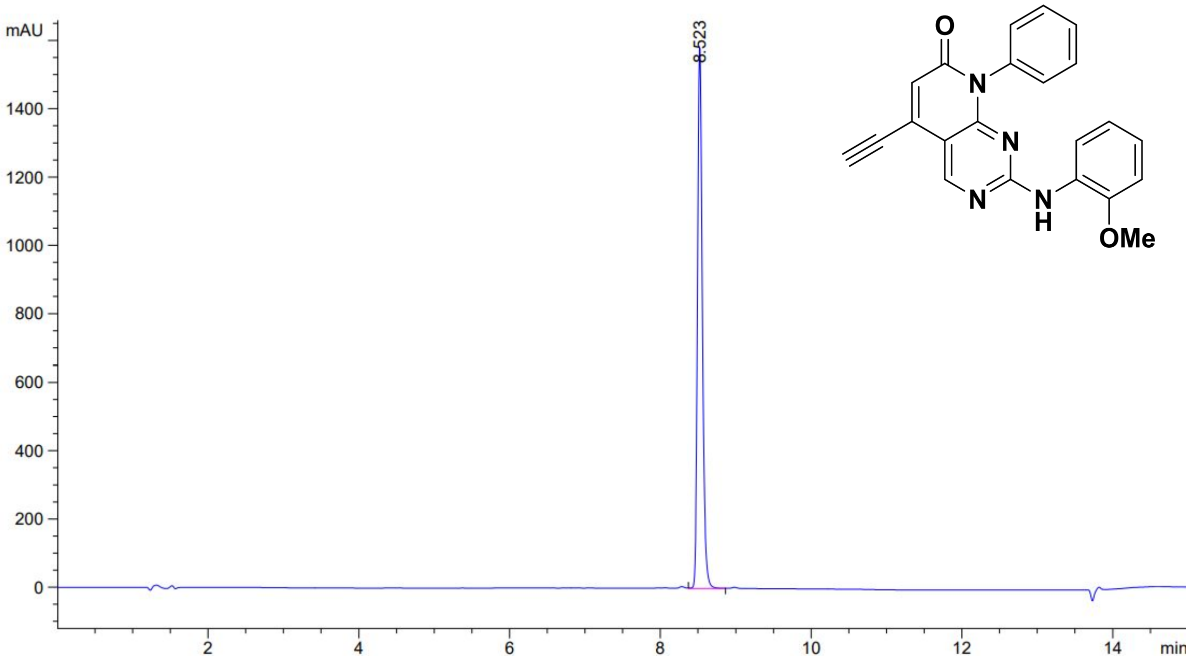

Basic – 98.8%

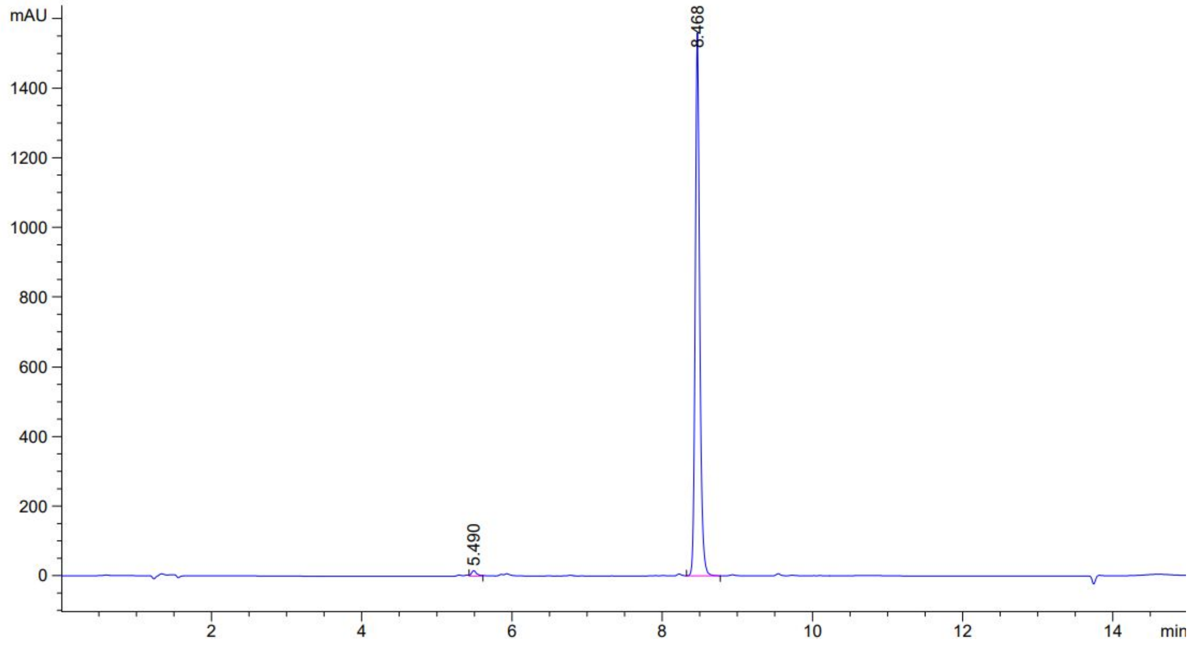

2-((2-methoxyphenyl)amino)-8-phenylpyrido[2,3-*d*]pyrimidin-7(8*H*)-one (S10)

Acidic – 97.7%

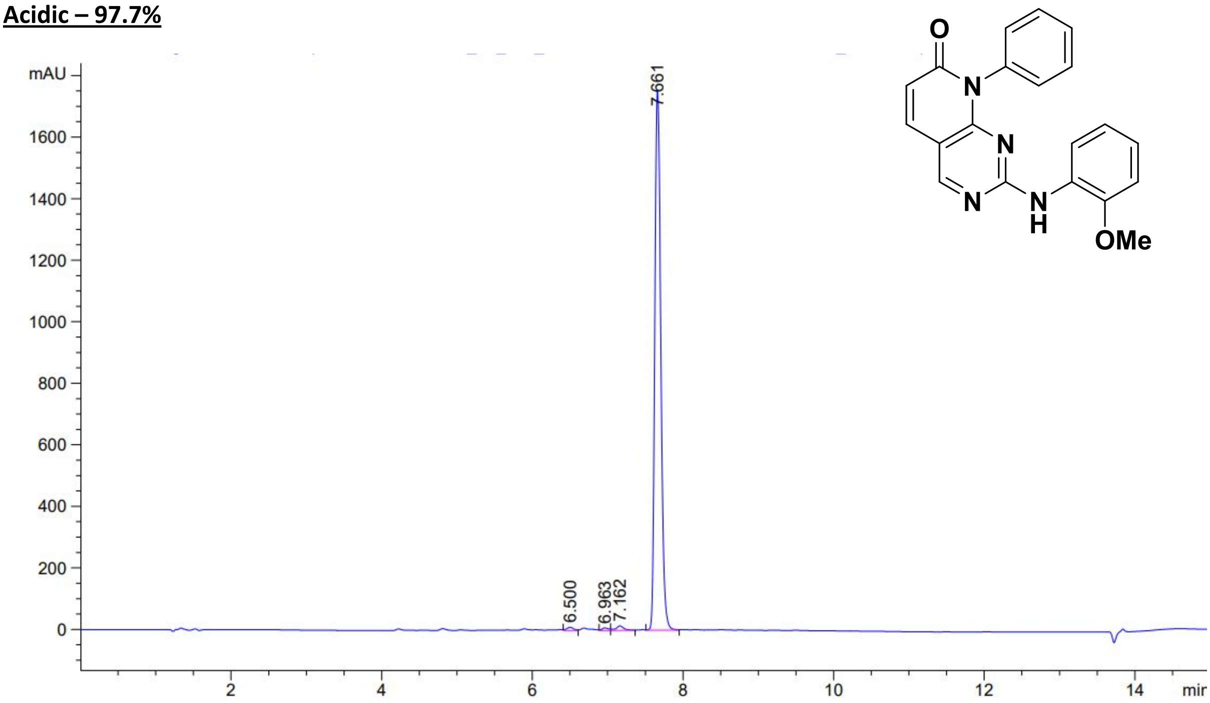

Basic – 96.6%

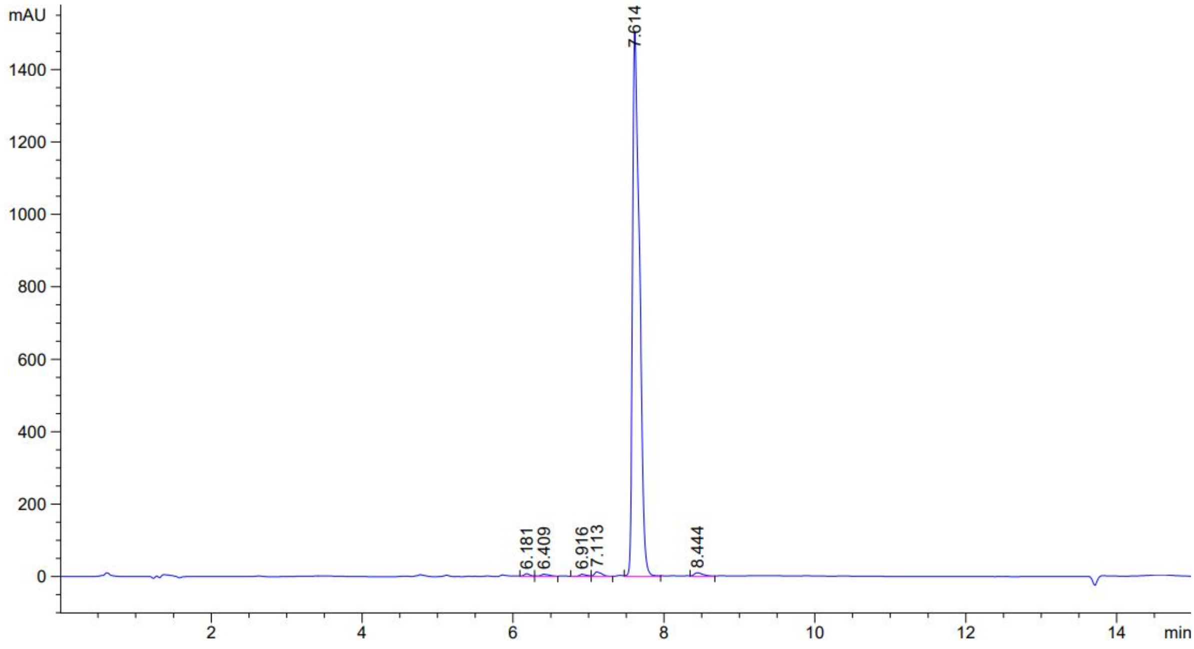

2-((2-Methoxyphenyl)amino)-8-phenyl-5-vinylpyrido[2,3-d]pyrimidin-7(8H)-one (S11)

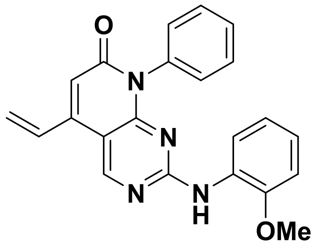

Acidic – 96.4%

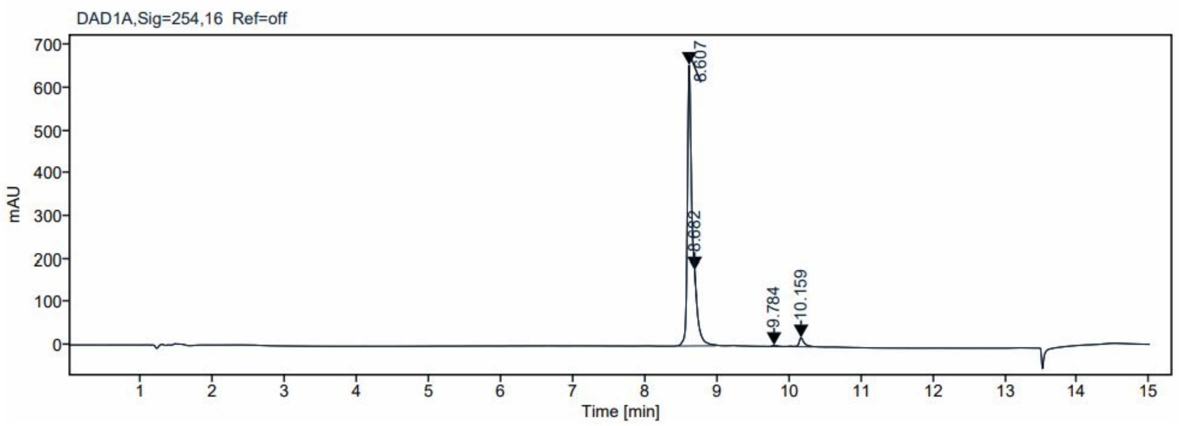

Basic – 95.0%

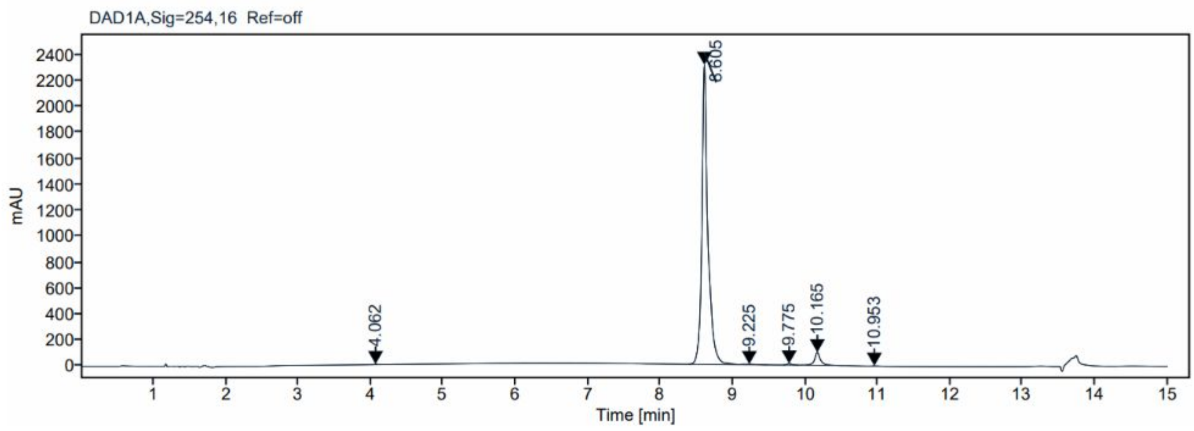

2-((2-Methoxyphenyl)amino)- 8-phenyl-5-(prop-1-yn-1-yl)pyrido[2,3-d]pyrimidin-7(8H)-one (S12)

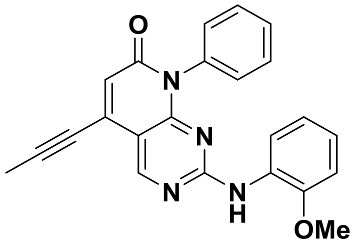

Acidic – 99.8%

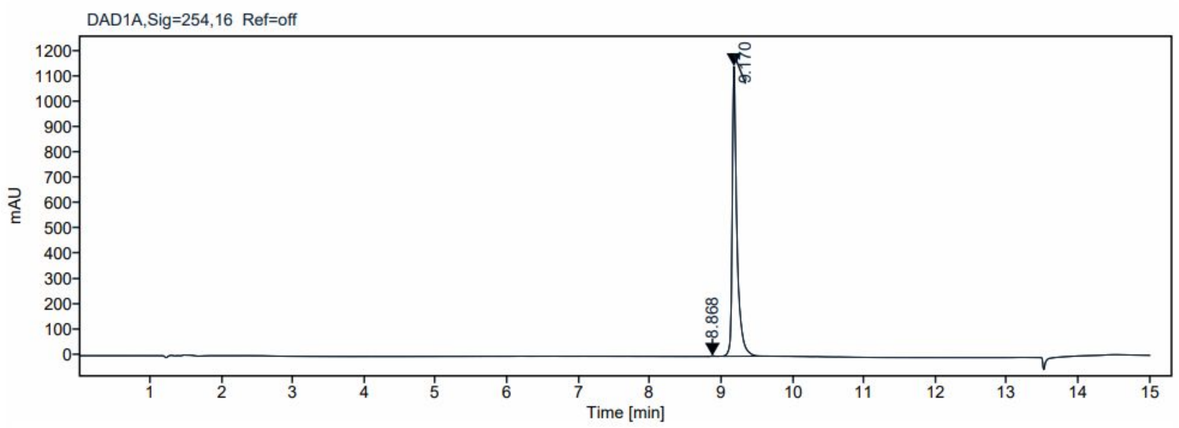

Basic – 97.5%

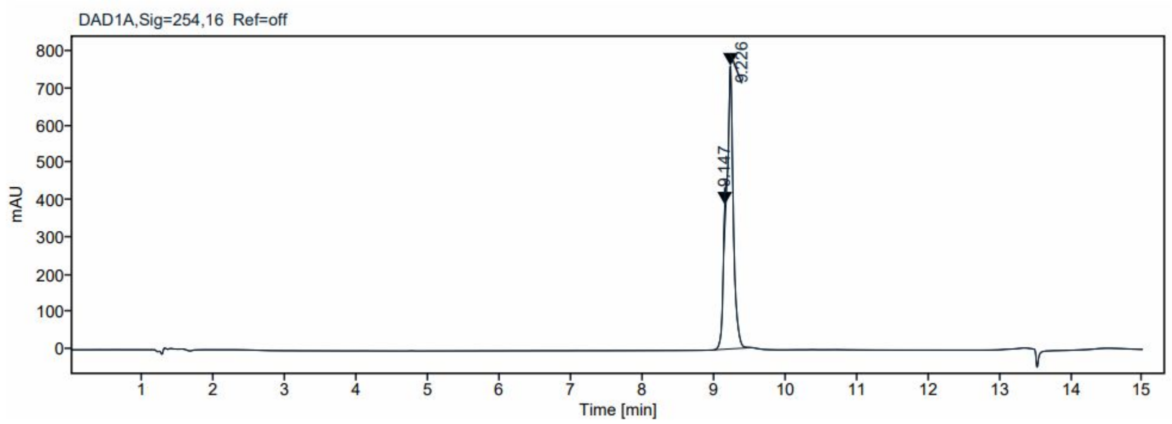

5-Ethynyl-2-((2-methoxyphenyl)amino)-8-phenylpyrido[2,3-*d*]pyrimidin-7(8*H*)-one (4)

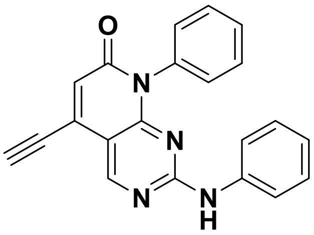

Acidic – 95.8%

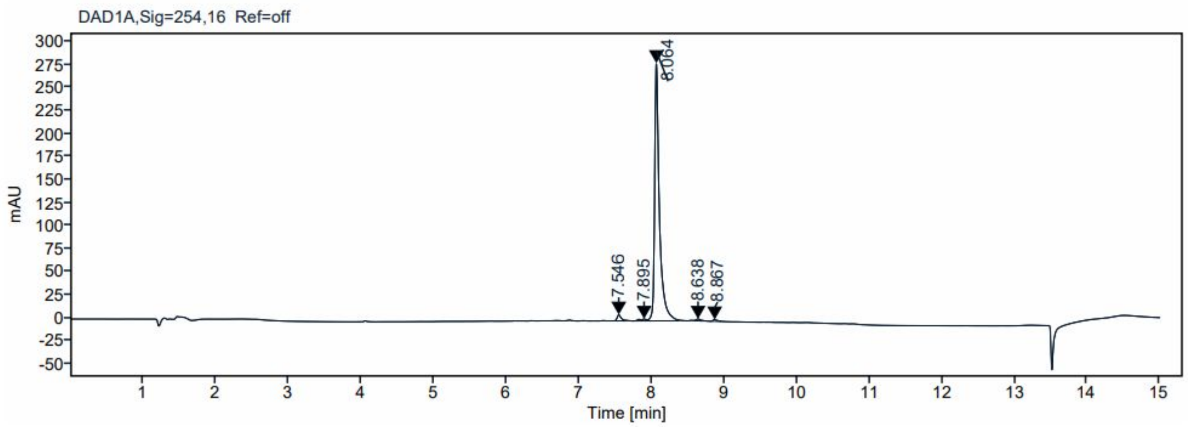

Basic – 95.8%

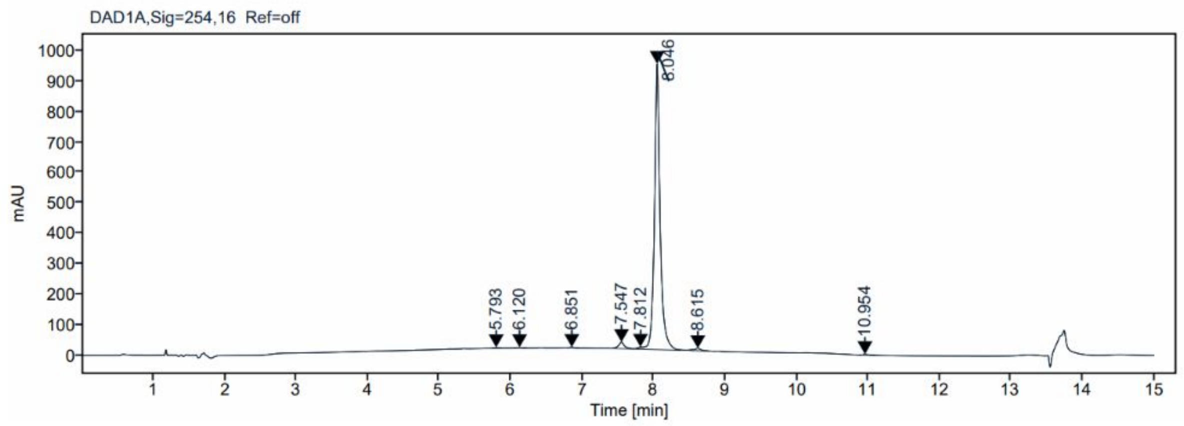

5-Ethynyl-2-((2-methoxy-4-morpholinophenyl)amino)-8-phenylpyrido[2,3-*d*]pyrimidin-7(8*H*)-one (5)

Acidic – 96.7%

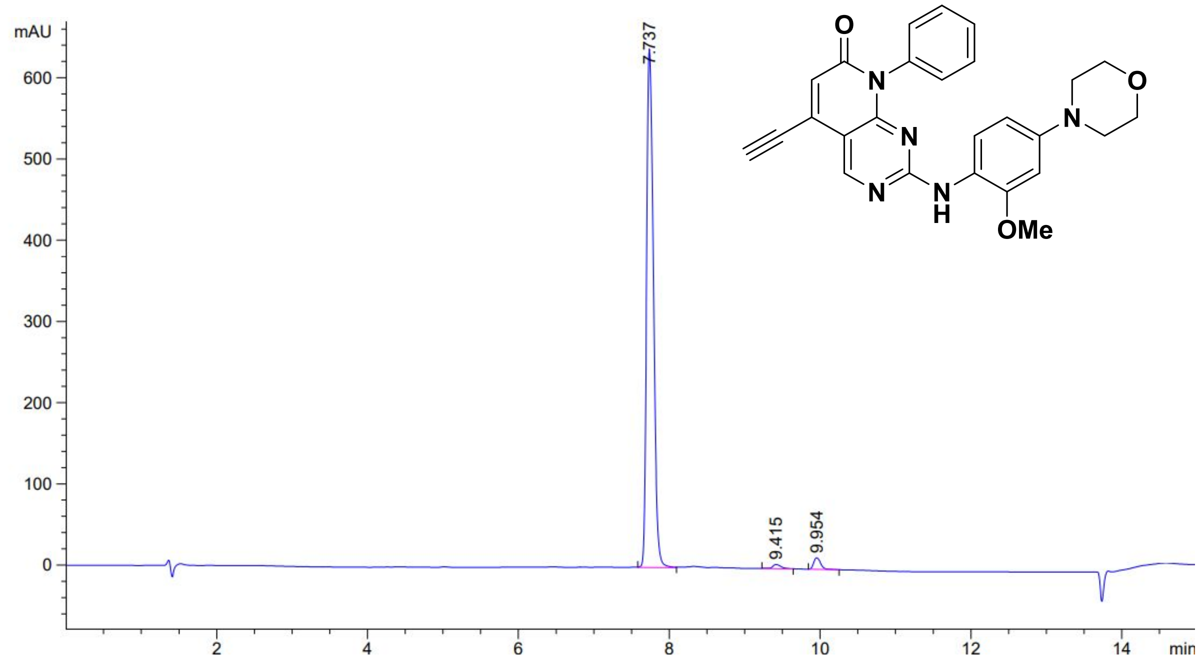

Basic – 78.5%

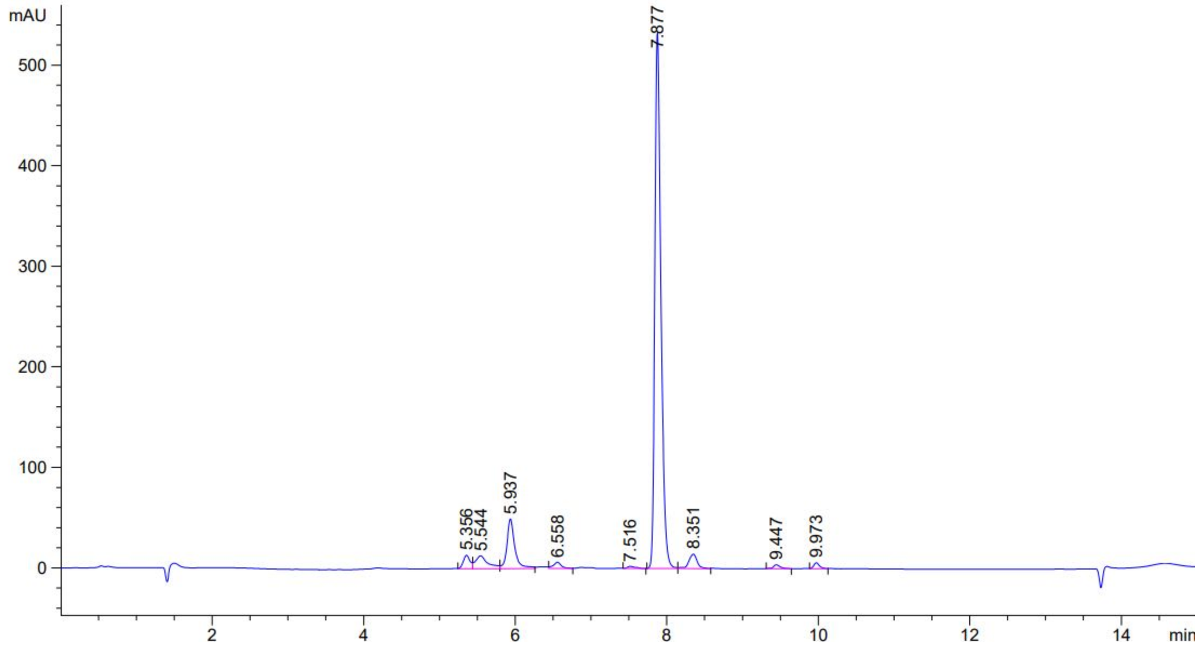

5-Ethynyl-2-((4-morpholinophenyl)amino)-8-phenylpyrido[2,3-*d*]pyrimidin-7(8*H*)-one (6)

Acidic – 98.7%

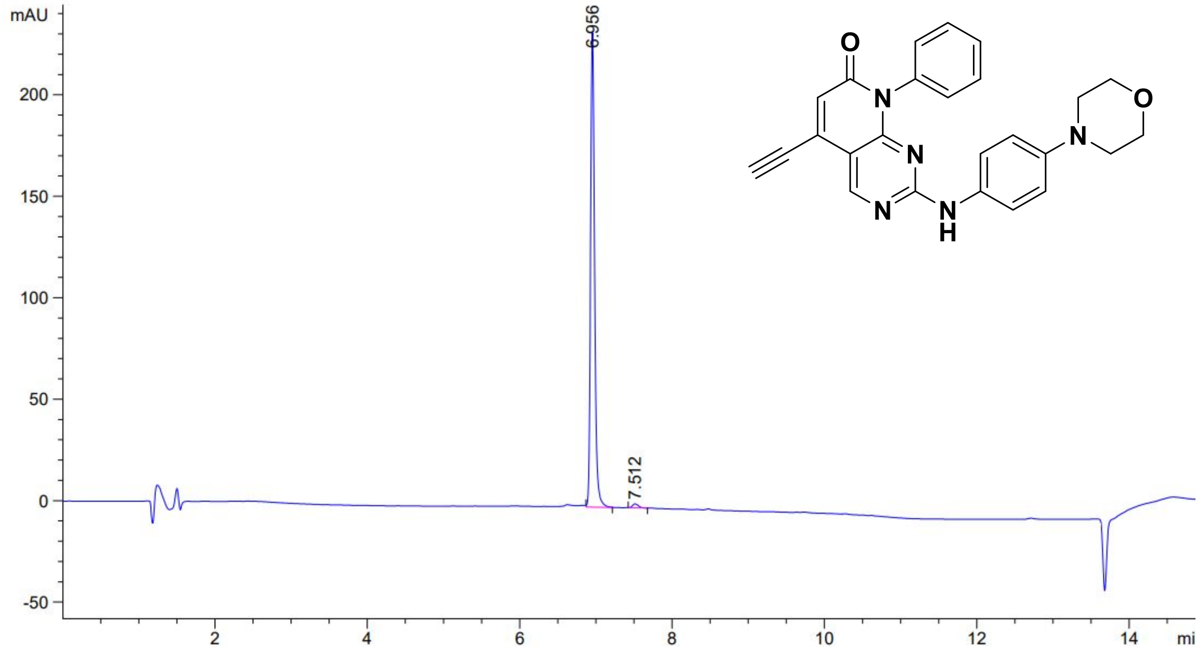

Basic – 98.4%

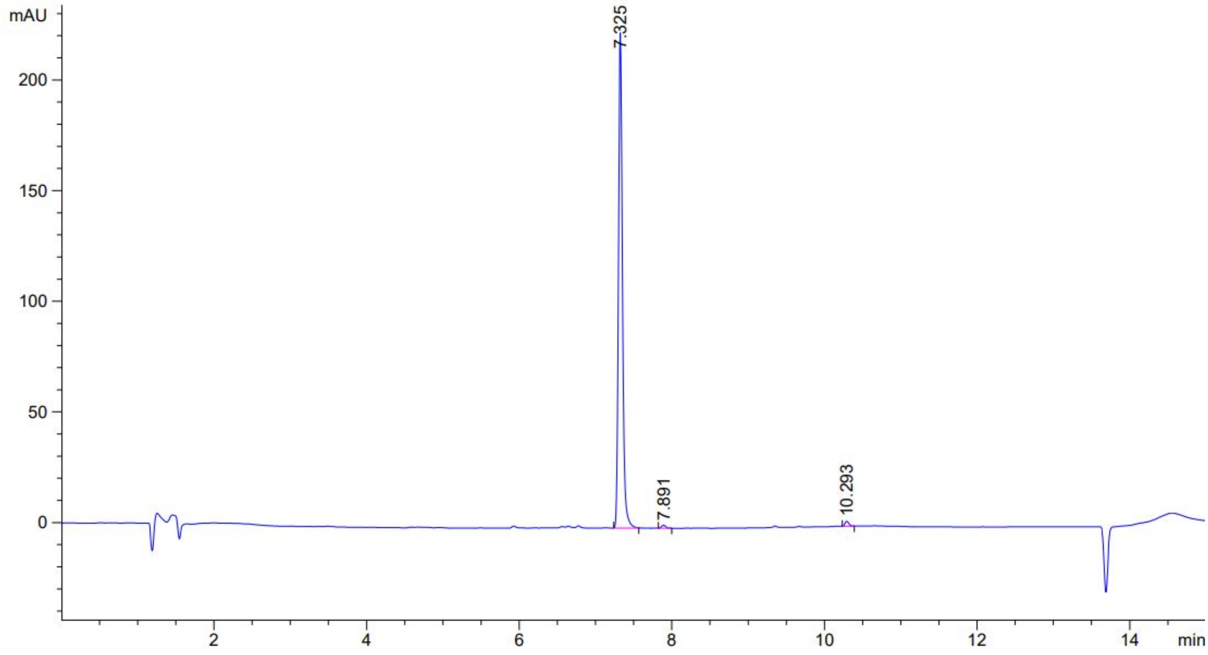

2-((4-((2-(Dimethylamino)ethyl)(methyl)amino)phenyl)amino)-5-ethynyl-8-phenylpyrido[2,3-*d*]pyrimidin-7(8*H*)-one (7)

Acidic - 95.8%

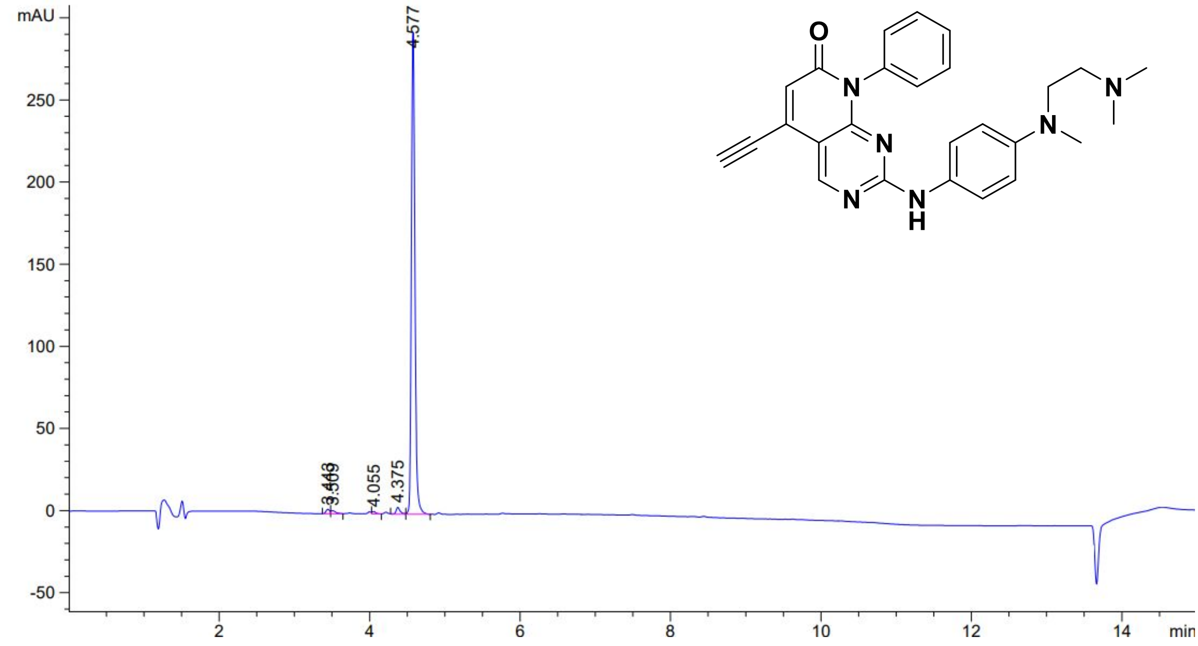

Basic – 95.8%

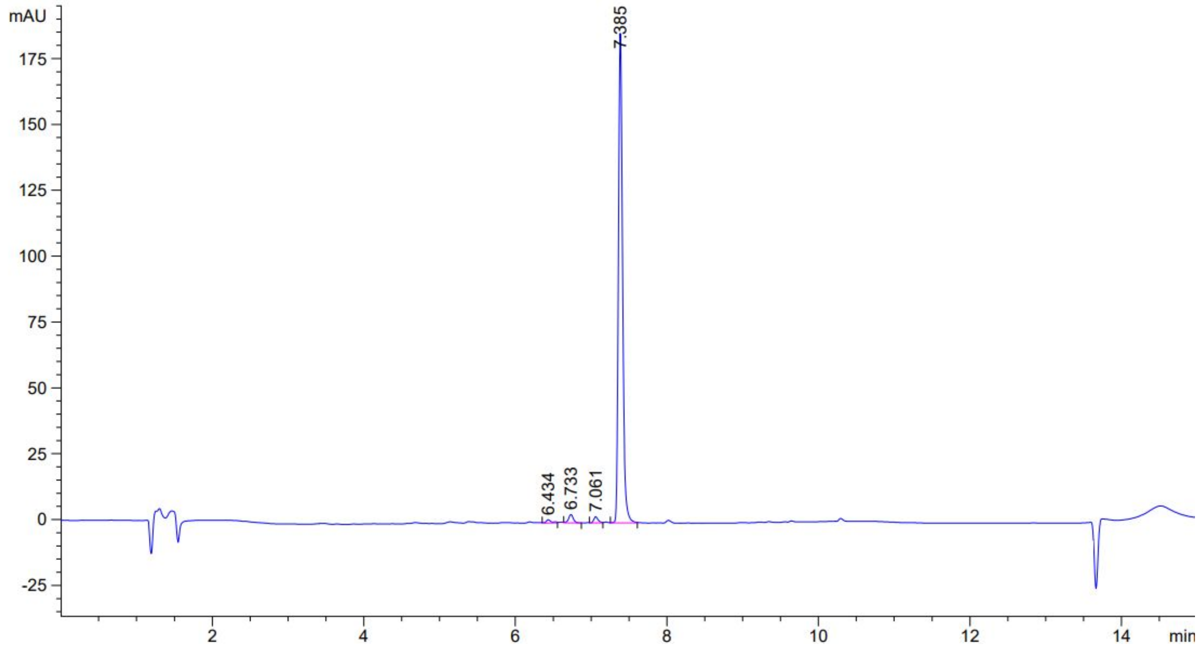

5-Ethynyl-2-((2-methoxy-4-(4-methylpiperazine-1-ylphenyl)amino)-8-phenylpyrido[2,3-*d*]pyrimidin-7(8*H*)-one (8)

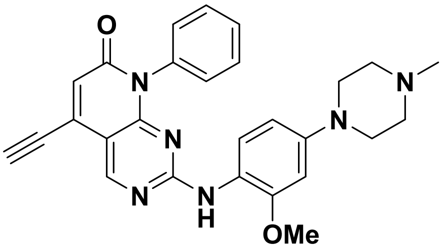

Acidic – 98.2%

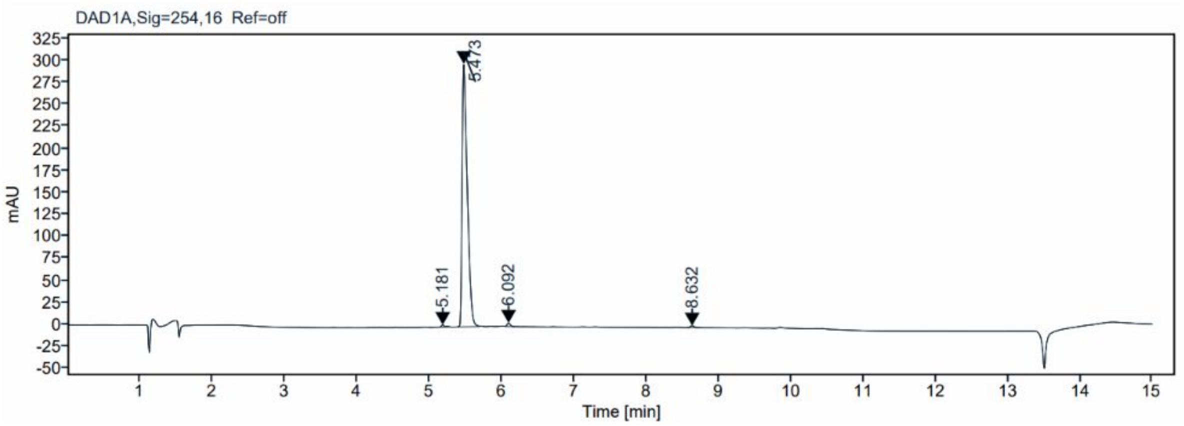

Basic – 73.7%

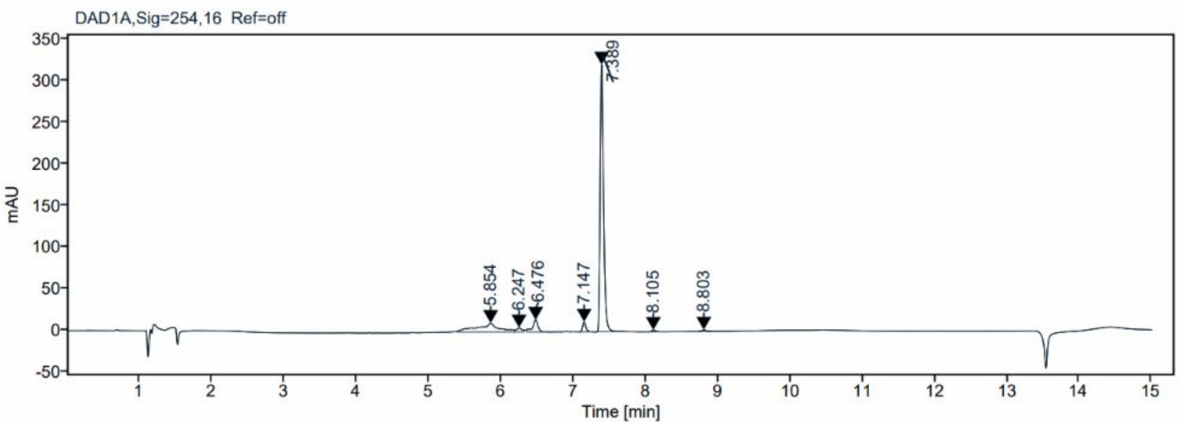

5-Ethynyl-2-((4-(4-methylpiperazin-1-yl)phenyl)amino)-8-phenylpyrido[2,3-d]pyrimidin-7(8H)-one (9)

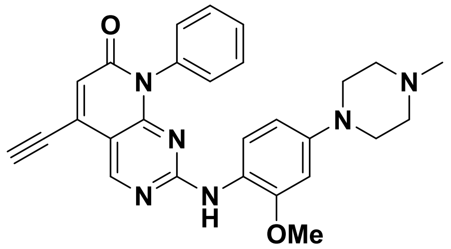

Acidic 96.4%

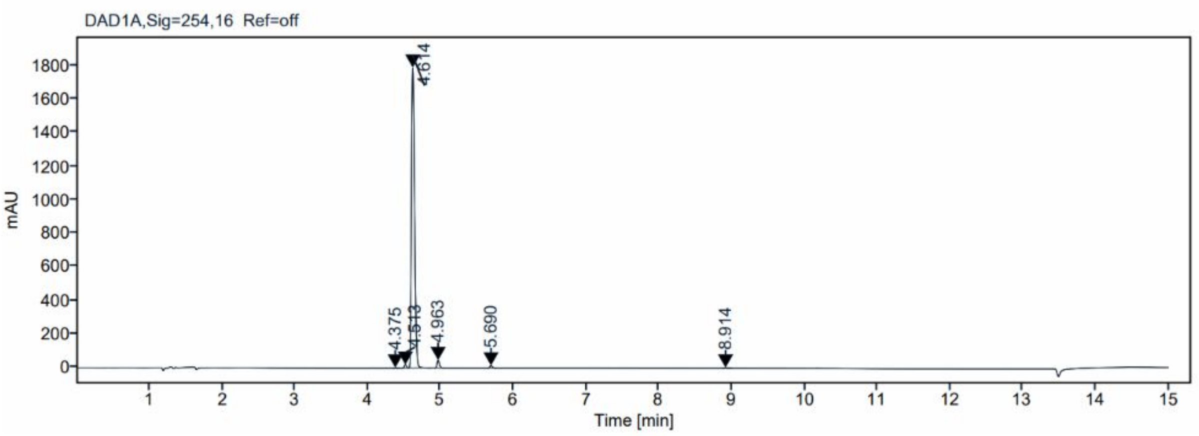

Basic 92.8%

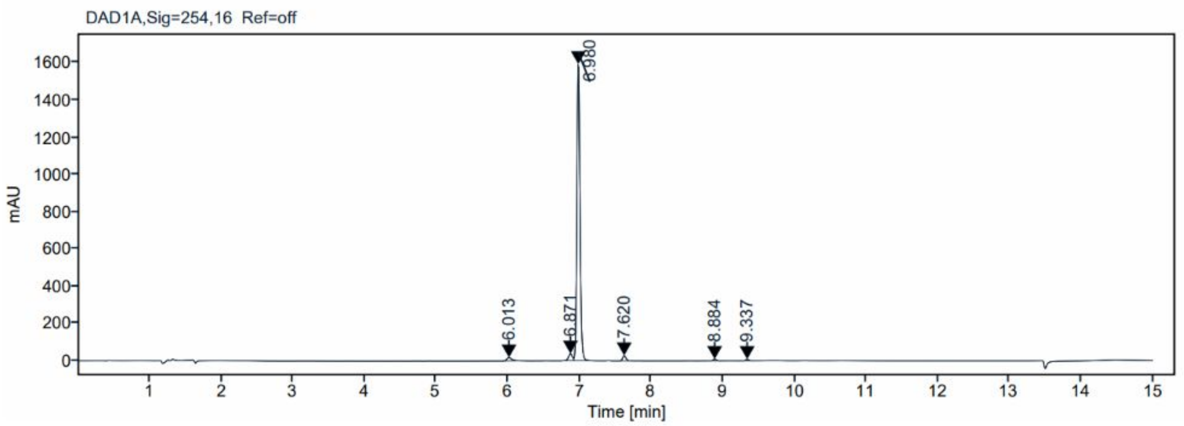

***N*-(4-((5-Ethynyl-7-oxo-8-phenyl-7,8-dihydropyrido[2,3-*d*]pyrimidin-2-yl)amino)-3-methoxyphenyl)-2-methoxy-*N*-methylacetamide (10)**

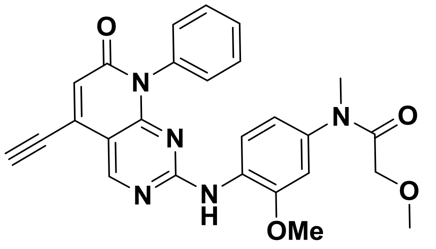

Acidic – 93.4%

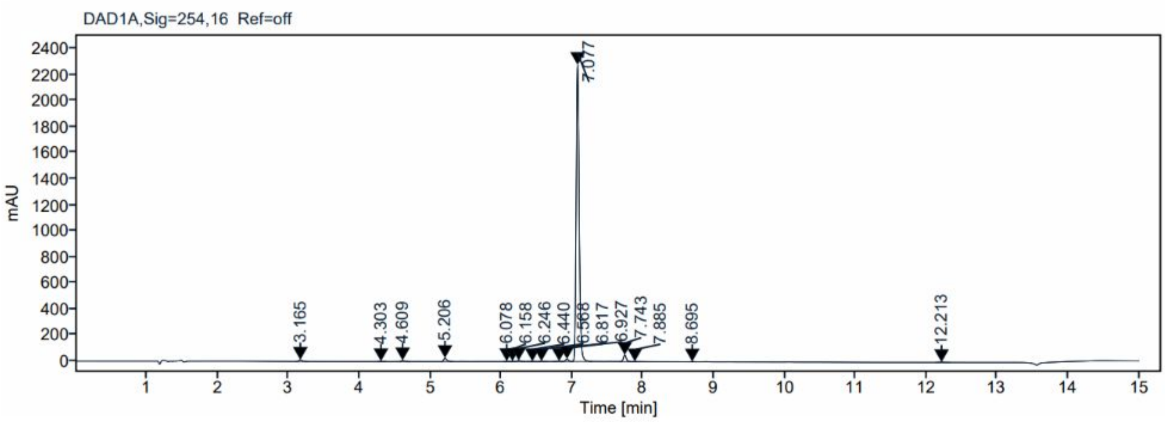

Basic – 93.7%

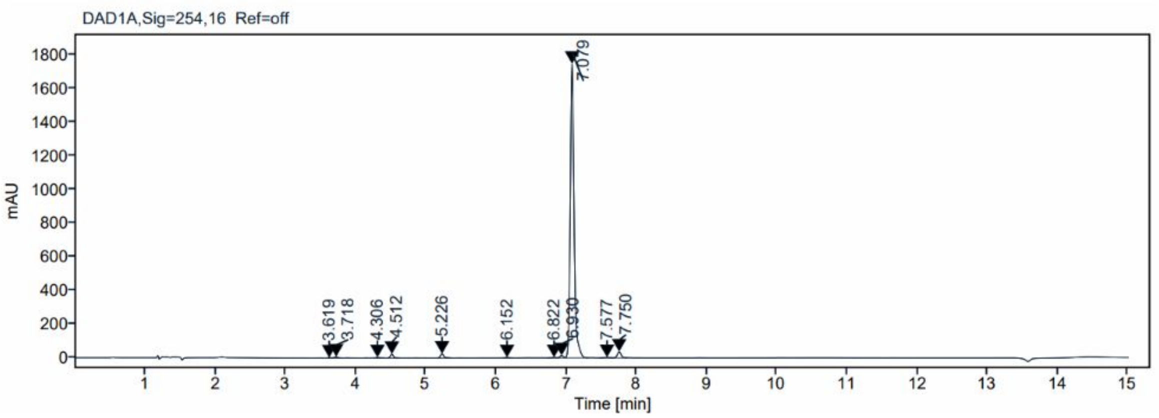

***N*-(4-((5-Ethynyl-7-oxo-8-phenyl-7,8-dihydropyrido[2,3-*d*]pyrimidin-2-yl)amino)-3-methoxyphenyl)-3-methoxy-*N*-methylpropanamide (11)**

Acidic – 100%

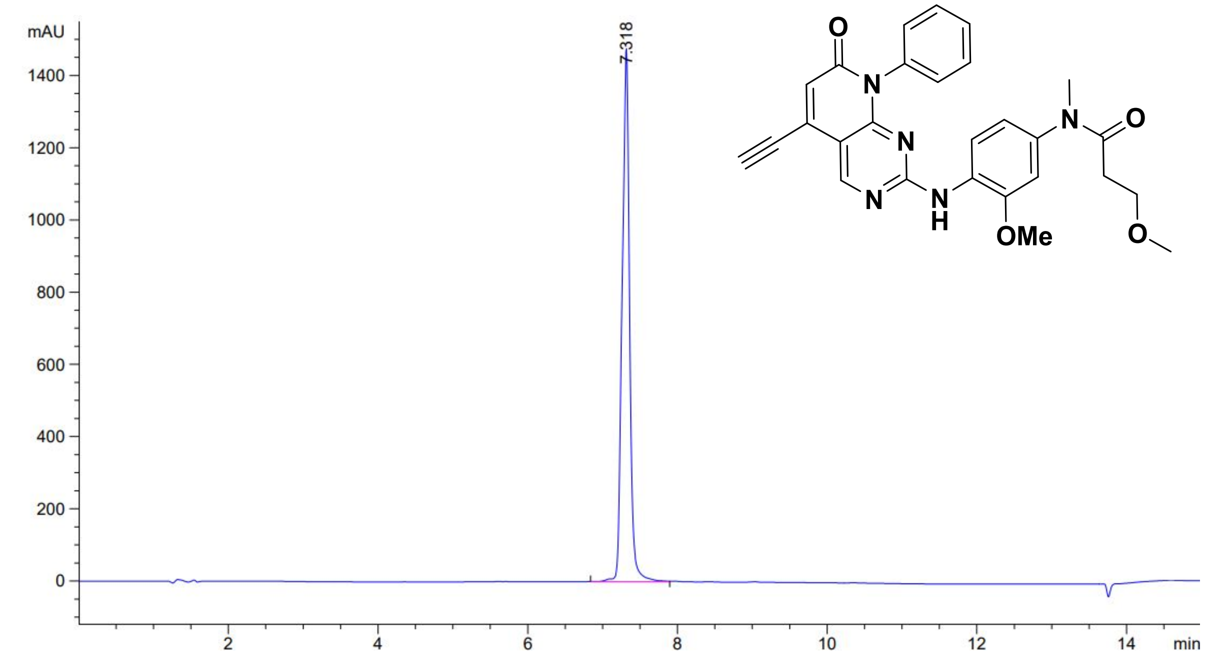

Basic - 100%

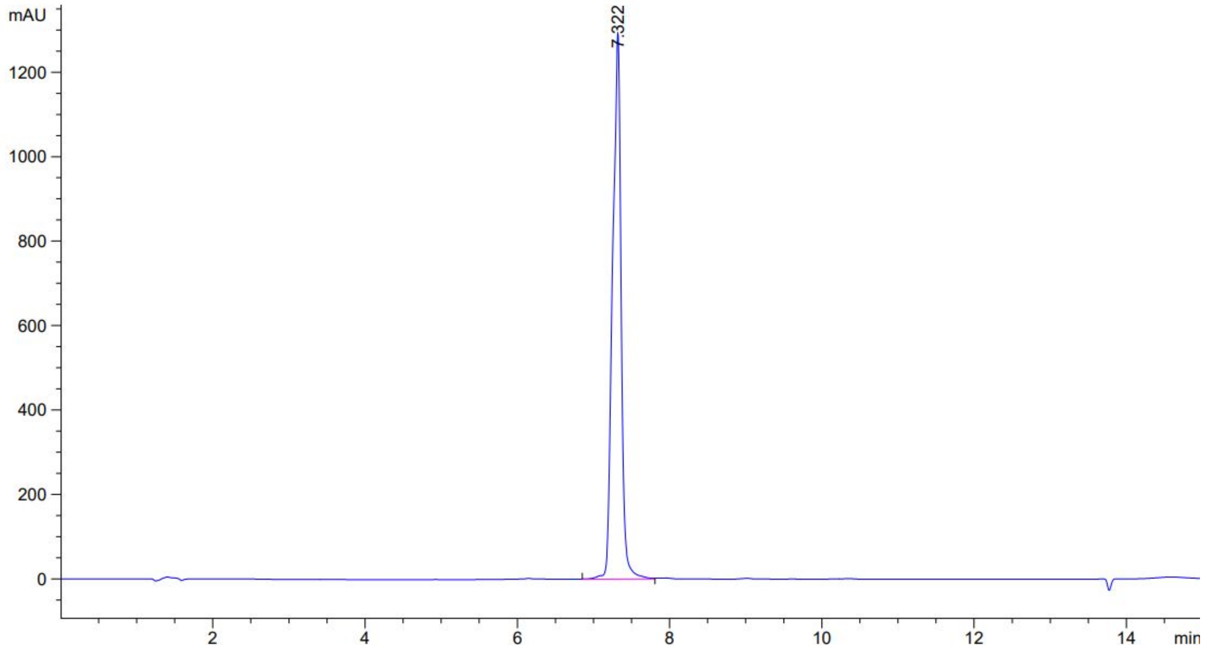

2-(Dimethylamino)-*N*-(4-((5-ethynyl-7-oxo-8-phenyl-7,8-dihydropyrido[2,3-*d*]pyrimidin-2-yl)amino)-3-methoxyphenyl)-*N*-methylacetamide (12)

Acidic – 100%

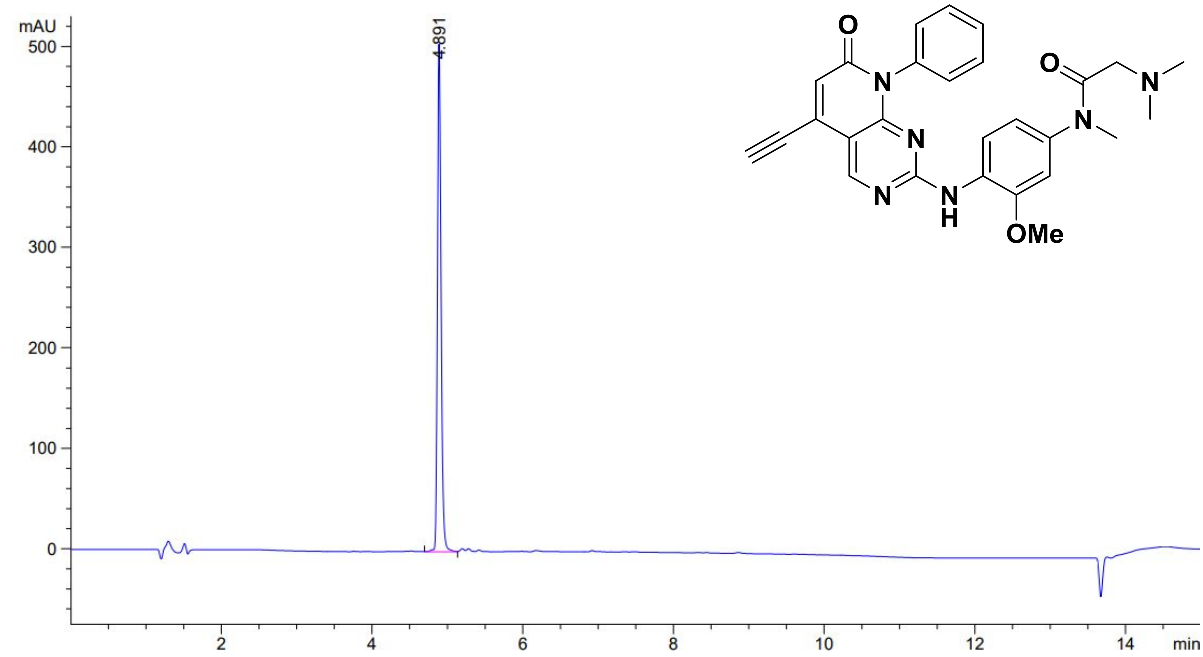

Basic – 100%

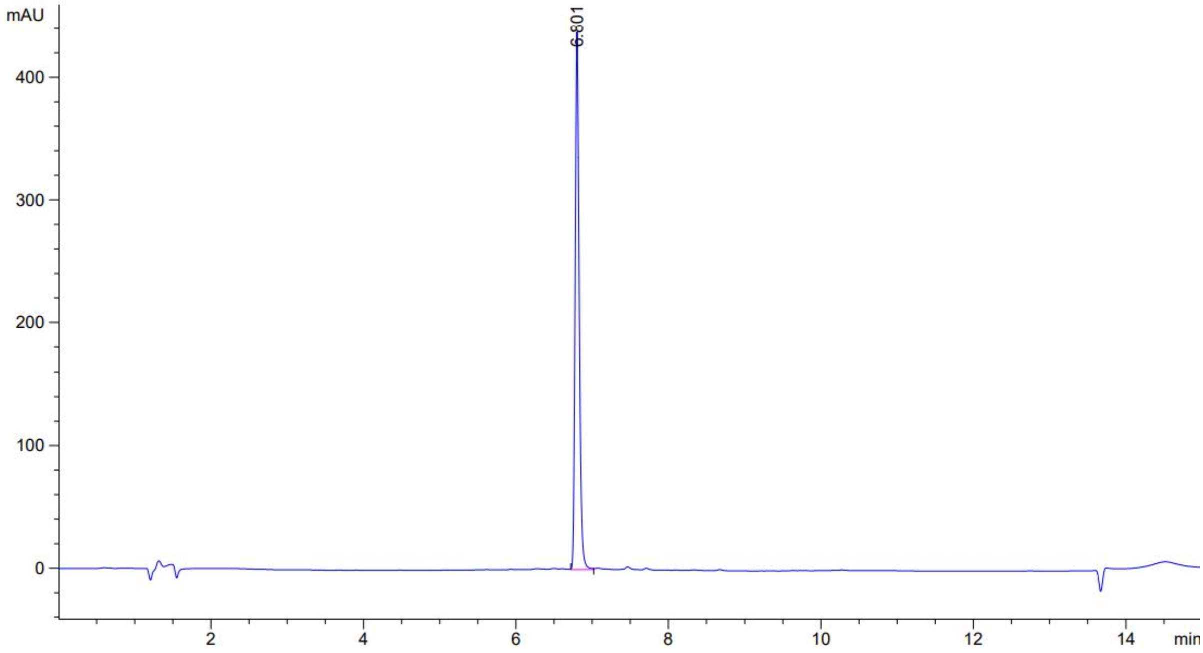

2-((4-((2-(dimethylamino)ethyl)(methyl)amino)phenyl)amino)-8-phenylpyrido[2,3-d]pyrimidin-7(8H)-one (S13)

Acidic – 97.9%

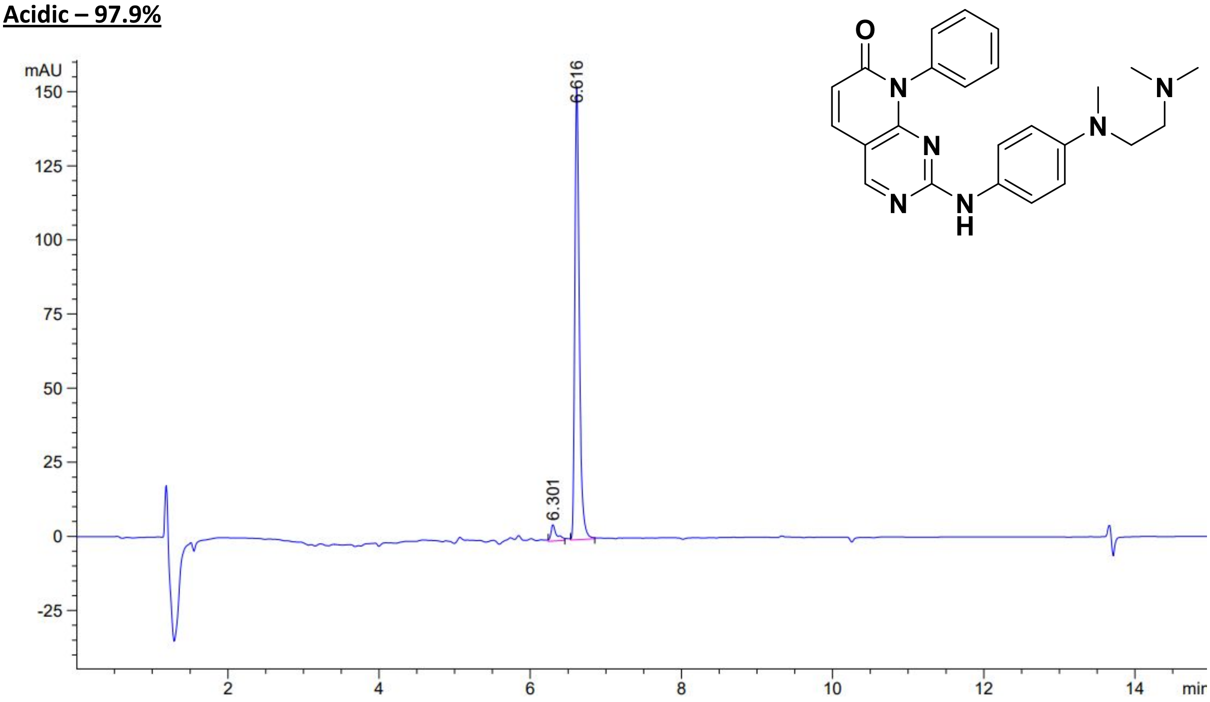

Basic – 95.4%

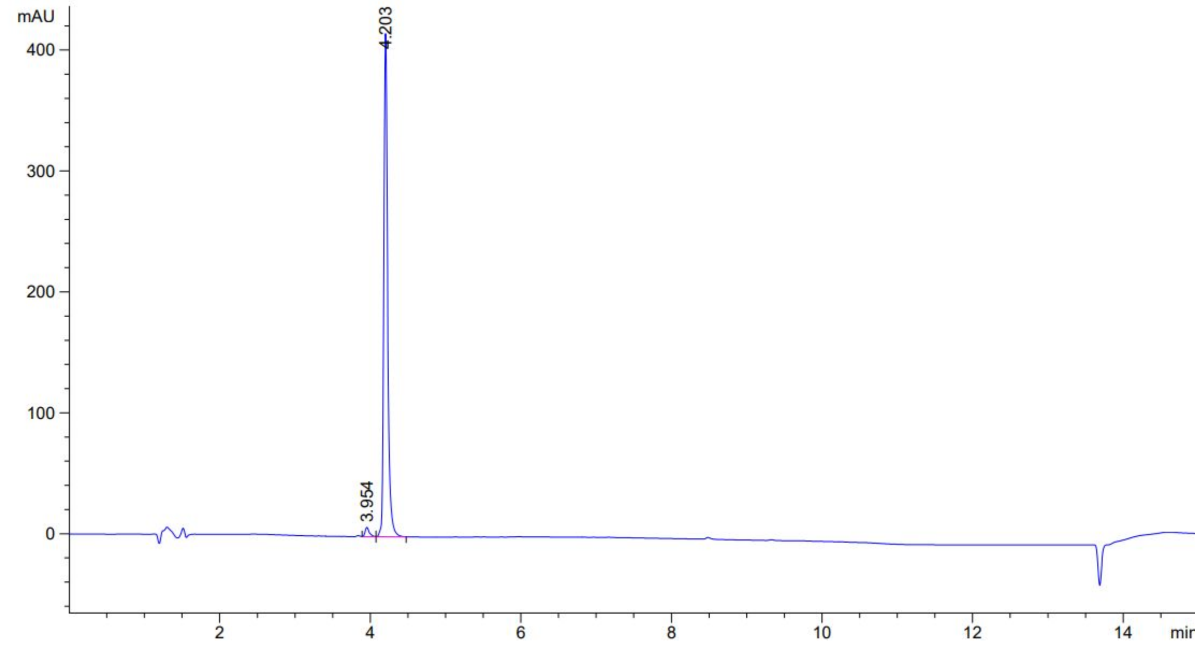

NMR Spectra

Scaffold A - 4-ethynyl-*N*-phenyl-6-(pyrazolo[1,5-a]pyridin-3-yl)pyrimidin-2-amine (2)

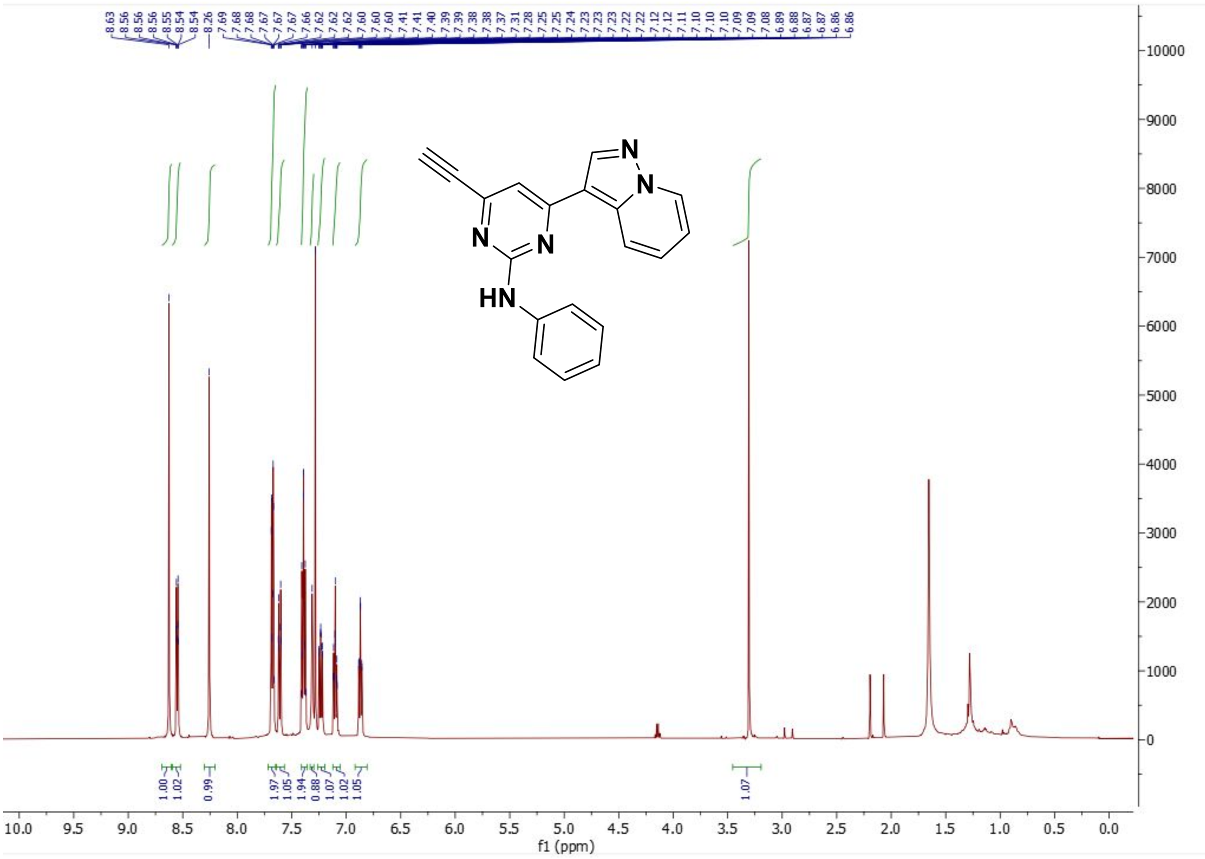

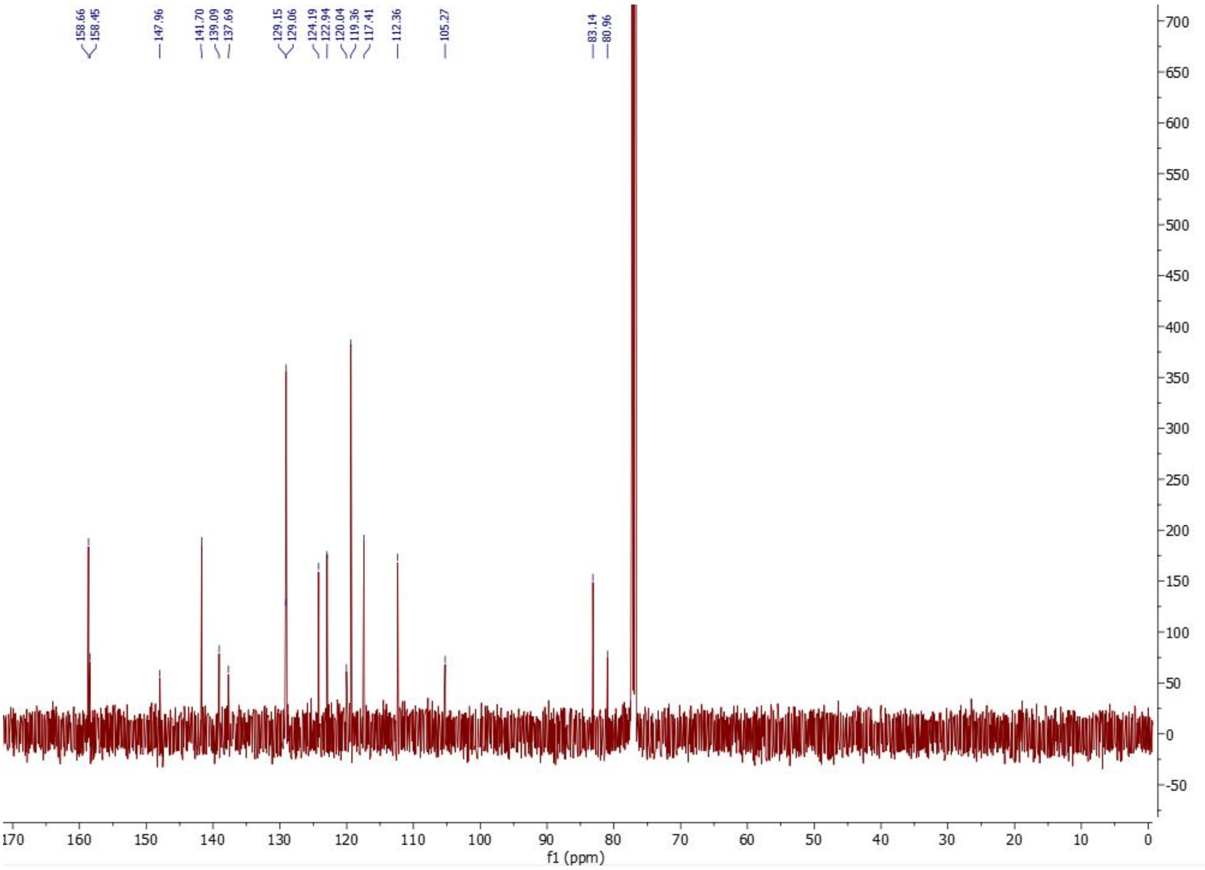

Scaffold B - N-(3-{4-Amino-1-methylpyrazolo[3,4-d]pyrimidin-3-yl}phenyl)prop-2-enamide (S1)

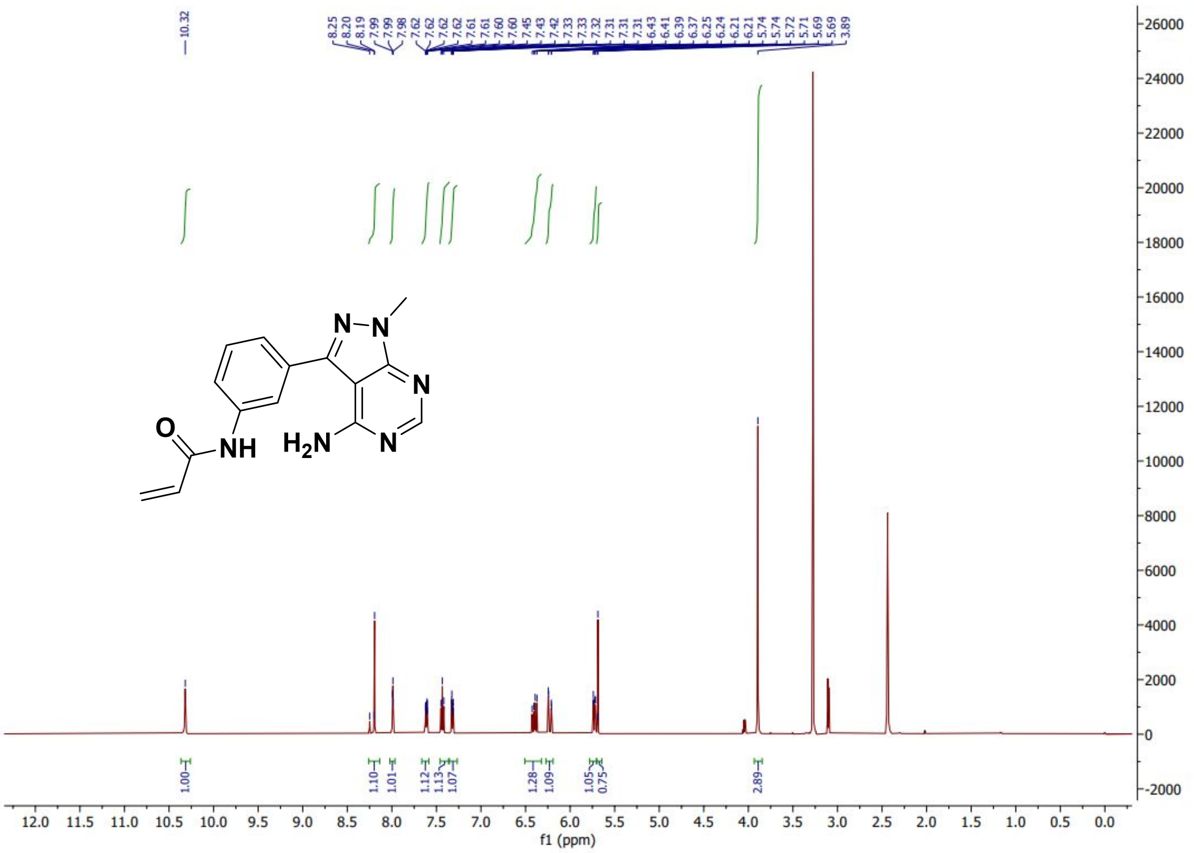

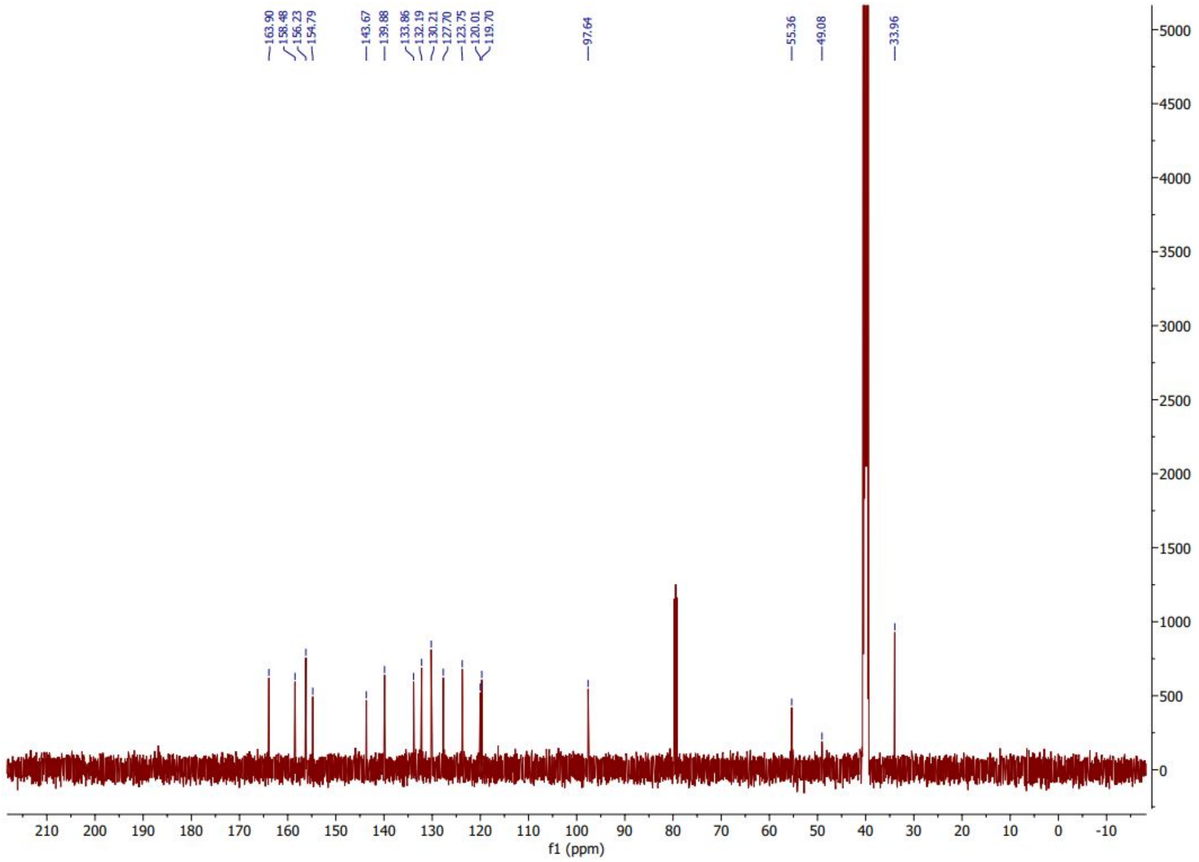

N-(3-{4-amino-1-methylpyrazolo[3,4-d]pyrimidin-3-yl}phenyl)prop-2-ynamide (S2)

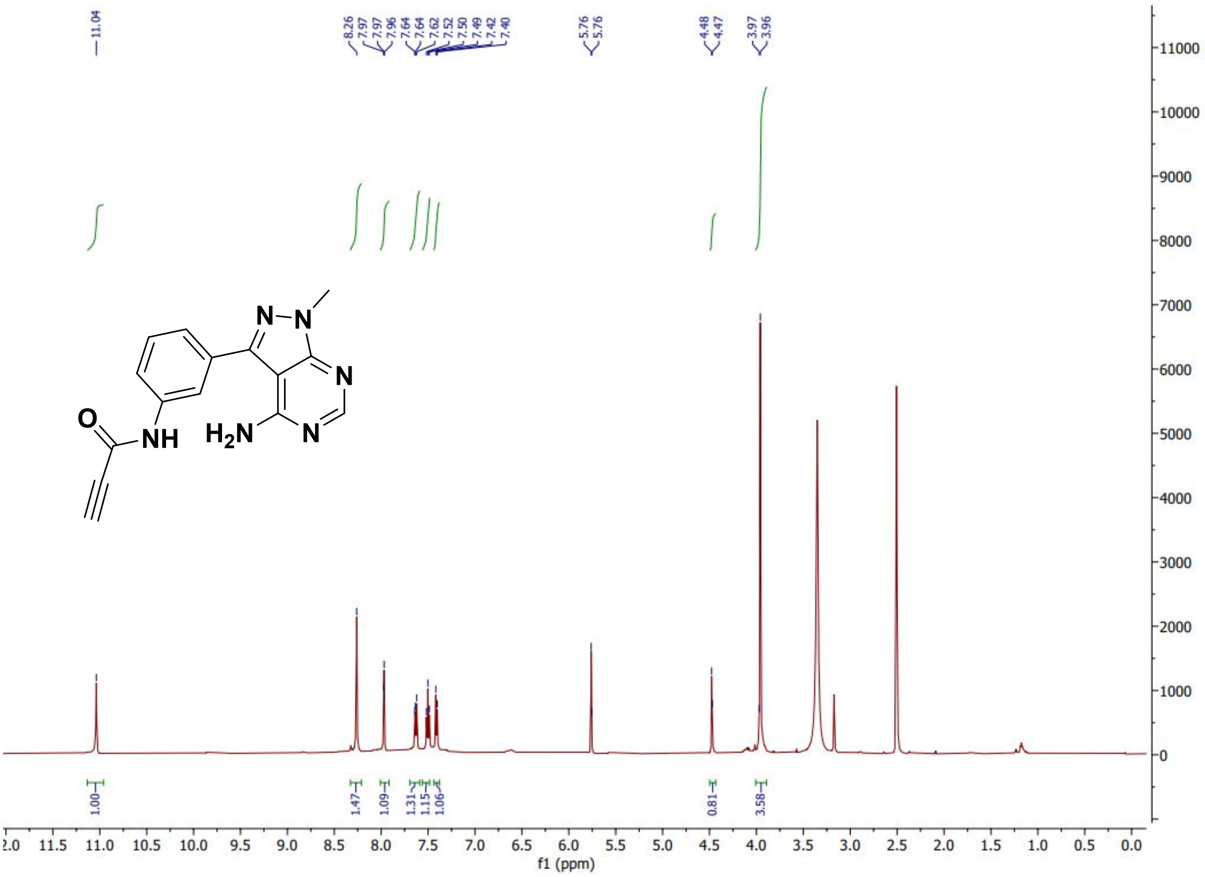

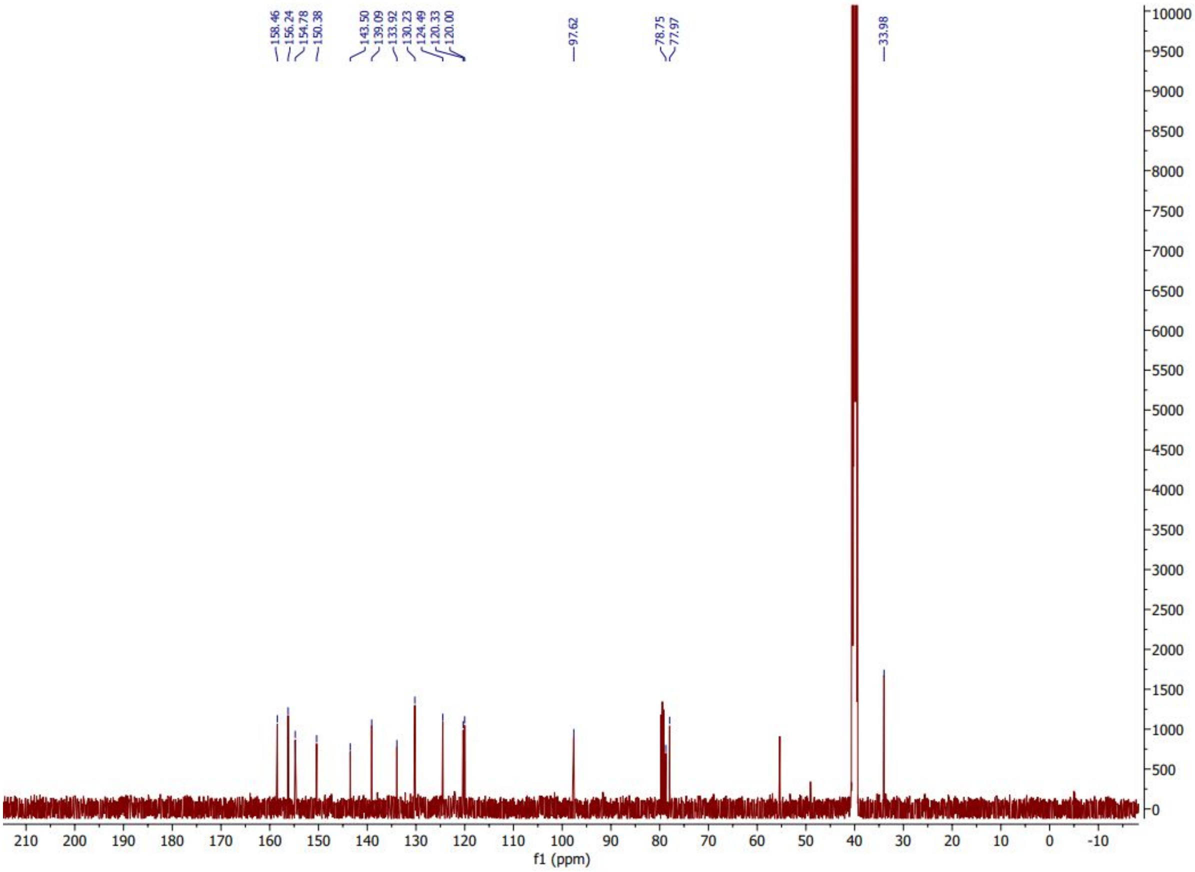

N-[(3-{4-Amino-1-methylpyrazolo[3,4-d]pyrimidin-3-yl}phenyl)methyl]prop-2-enamide (S3)

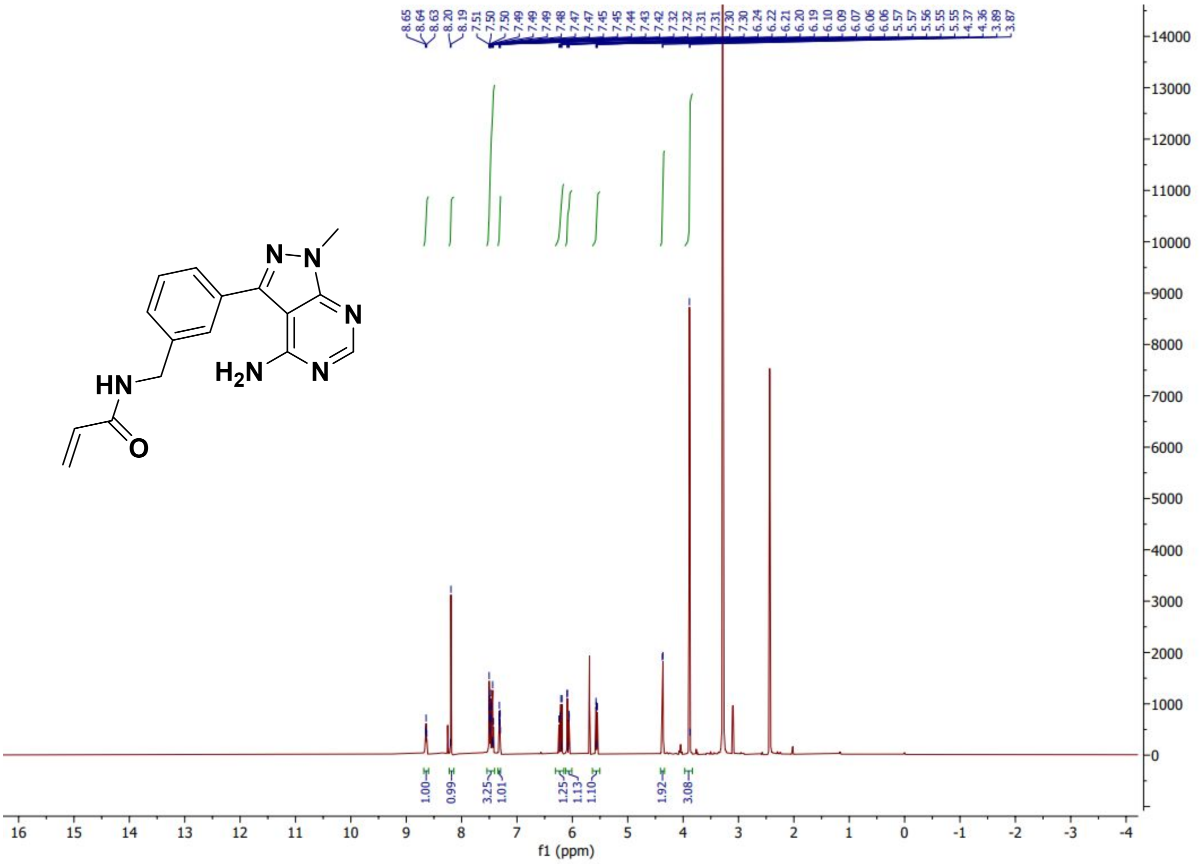

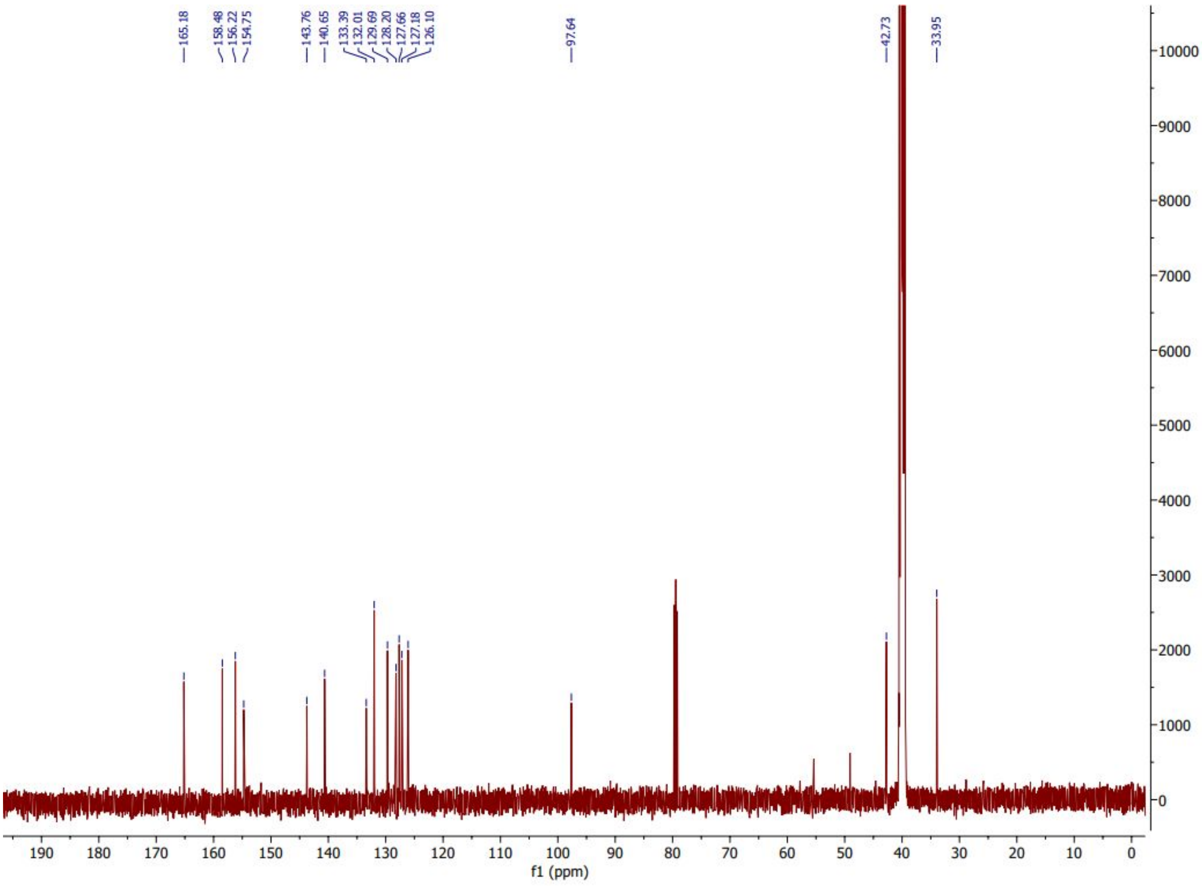

N-[2-(3-{4-Amino-1-methylpyrazolo[3,4-d]pyrimidin-3-yl}phenoxy)ethyl]prop-2-enamide (S6)

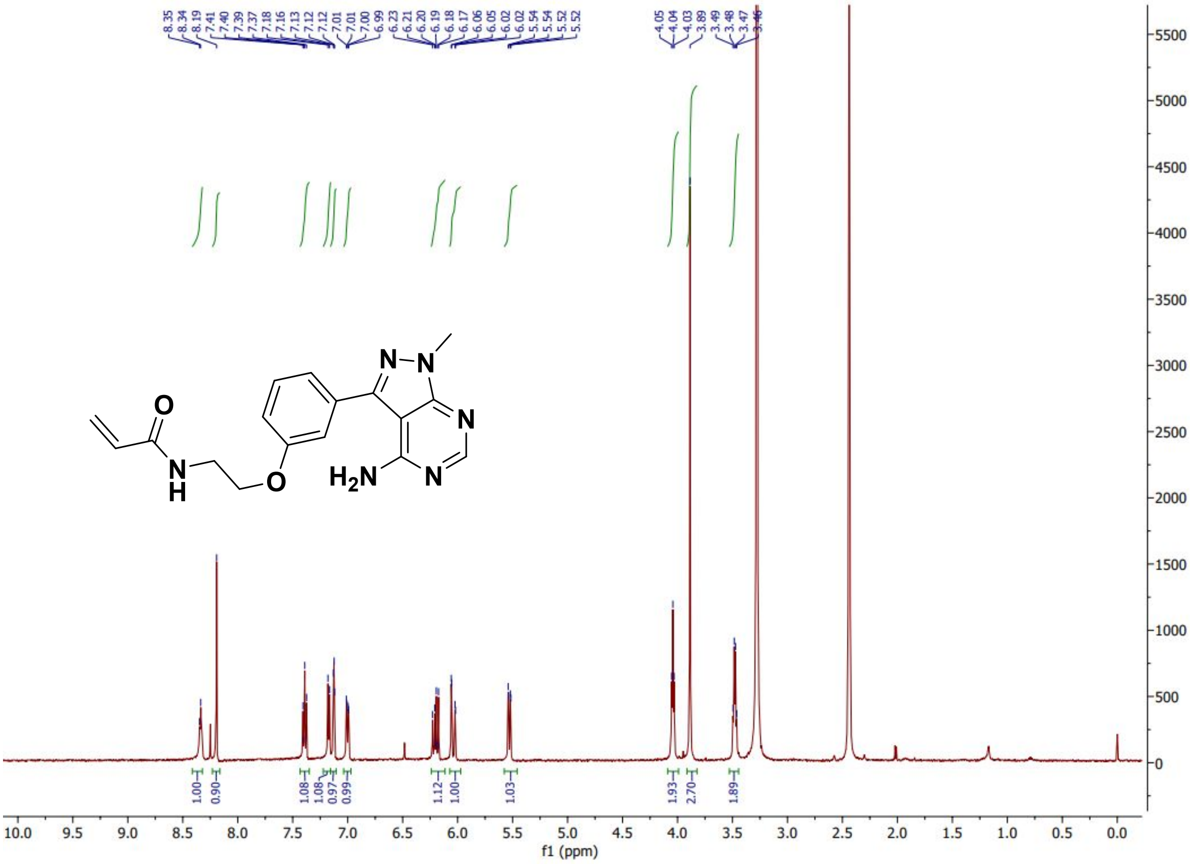

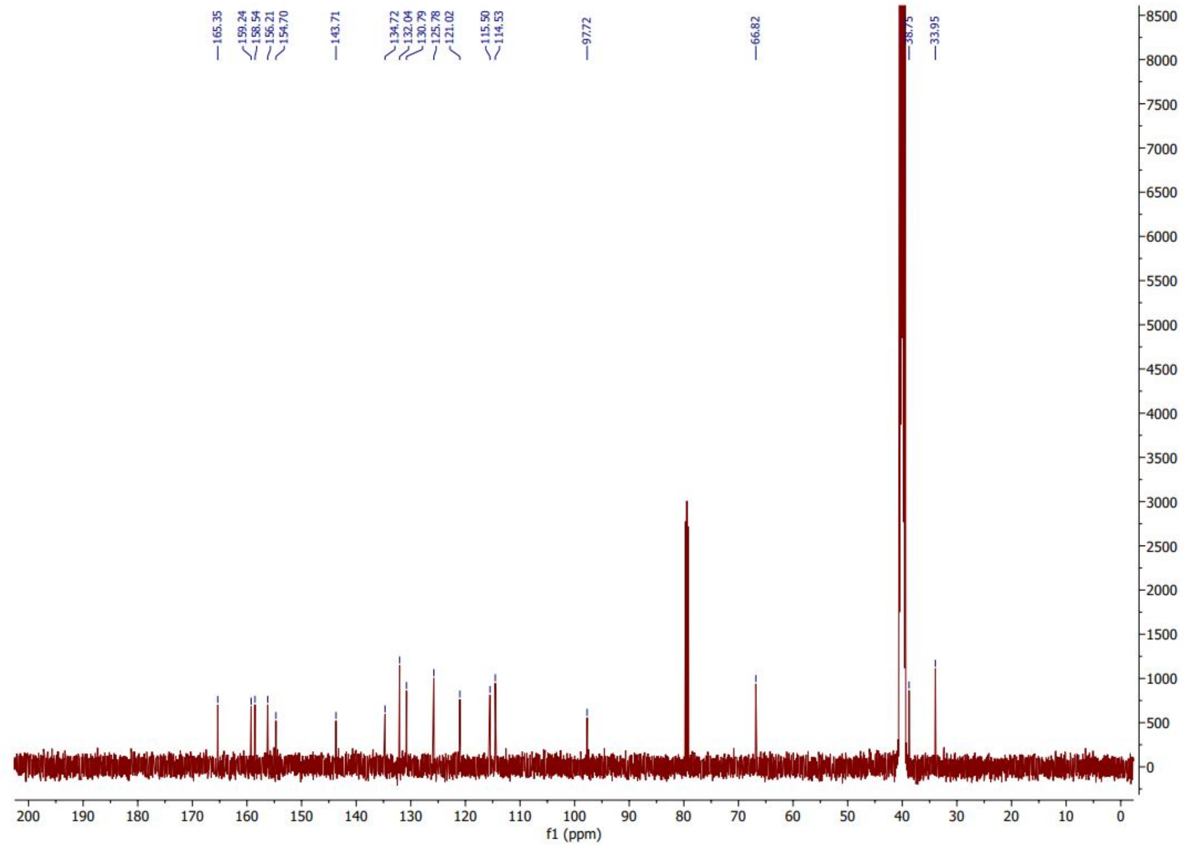

***N*-(2-(4-(4-amino-1-methyl-1H-pyrazolo[3,4-*d*]pyrimidin-3-yl)phenoxy)ethyl)acrylamide (S7)**

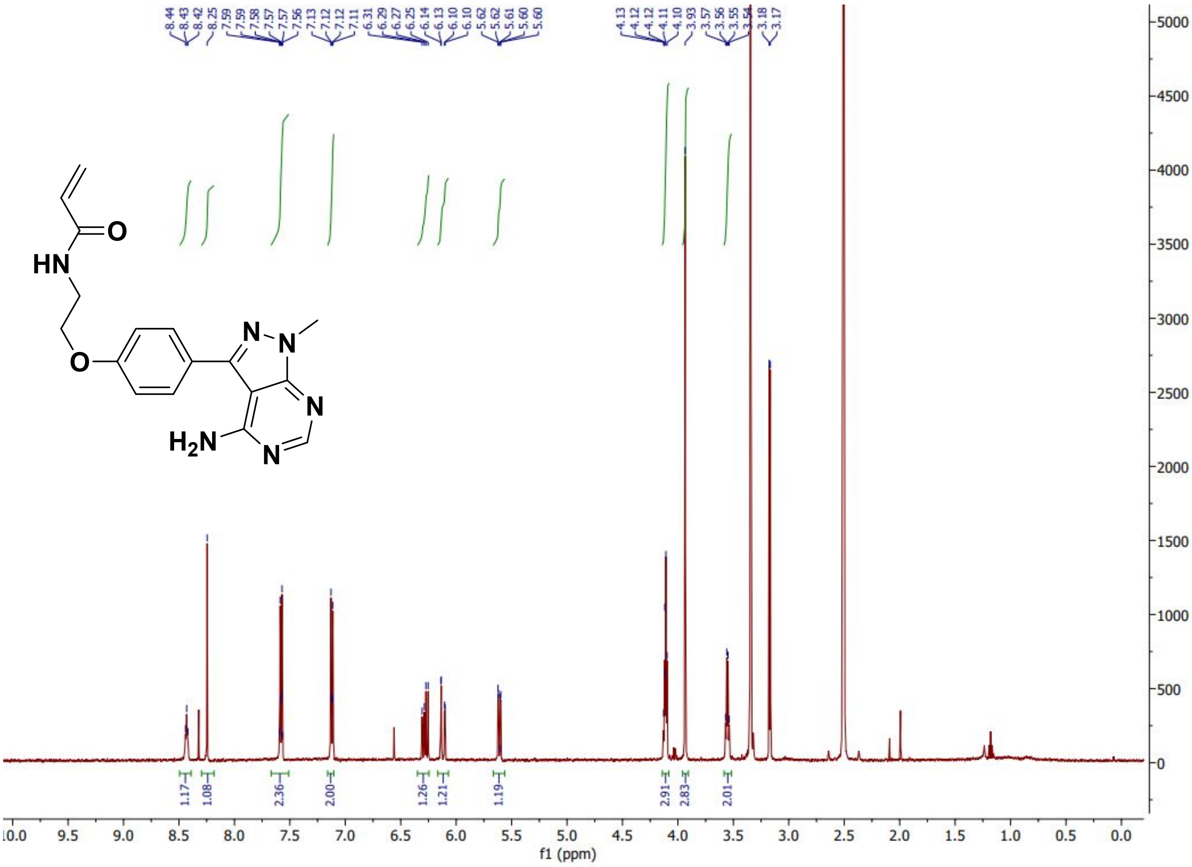

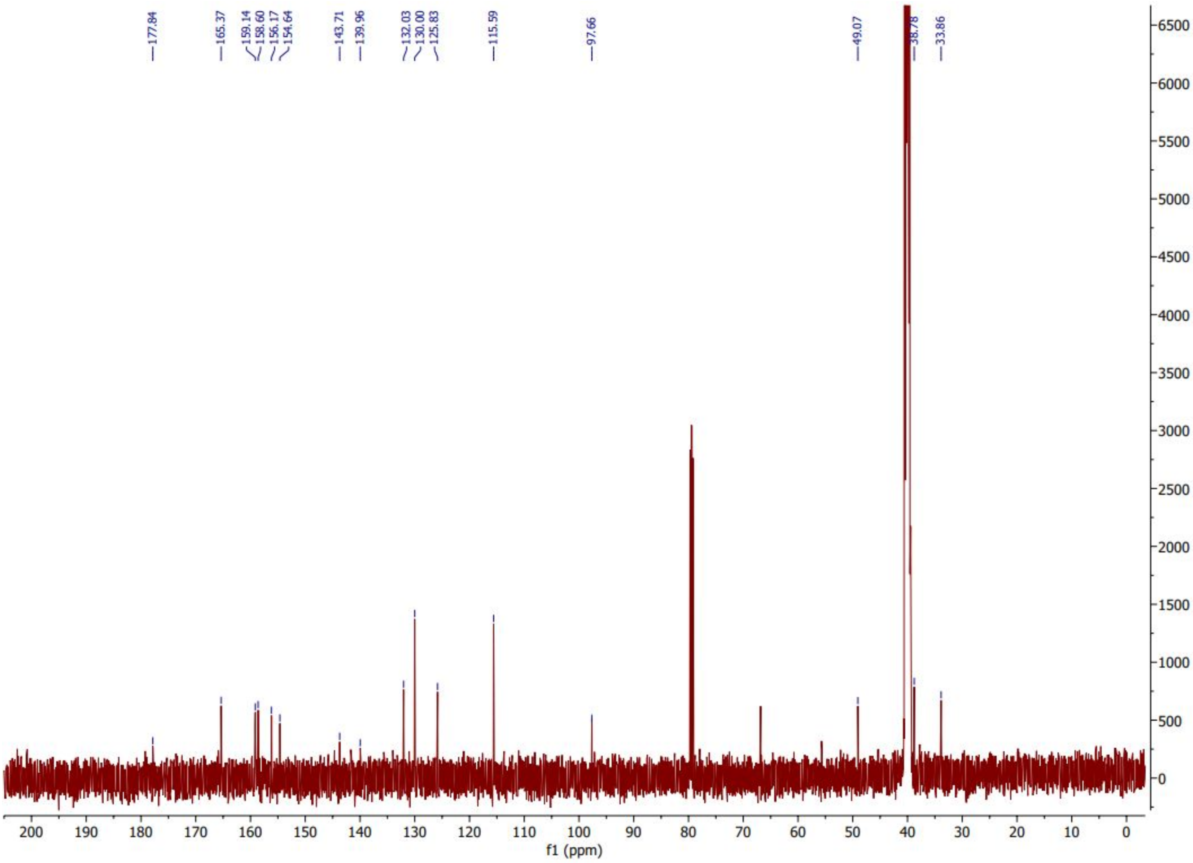

N-[2-(4-{4-Amino-1-methylpyrazolo[3,4-d]pyrimidin-3-yl}phenoxy)phenyl]prop-2-enamide (S8)

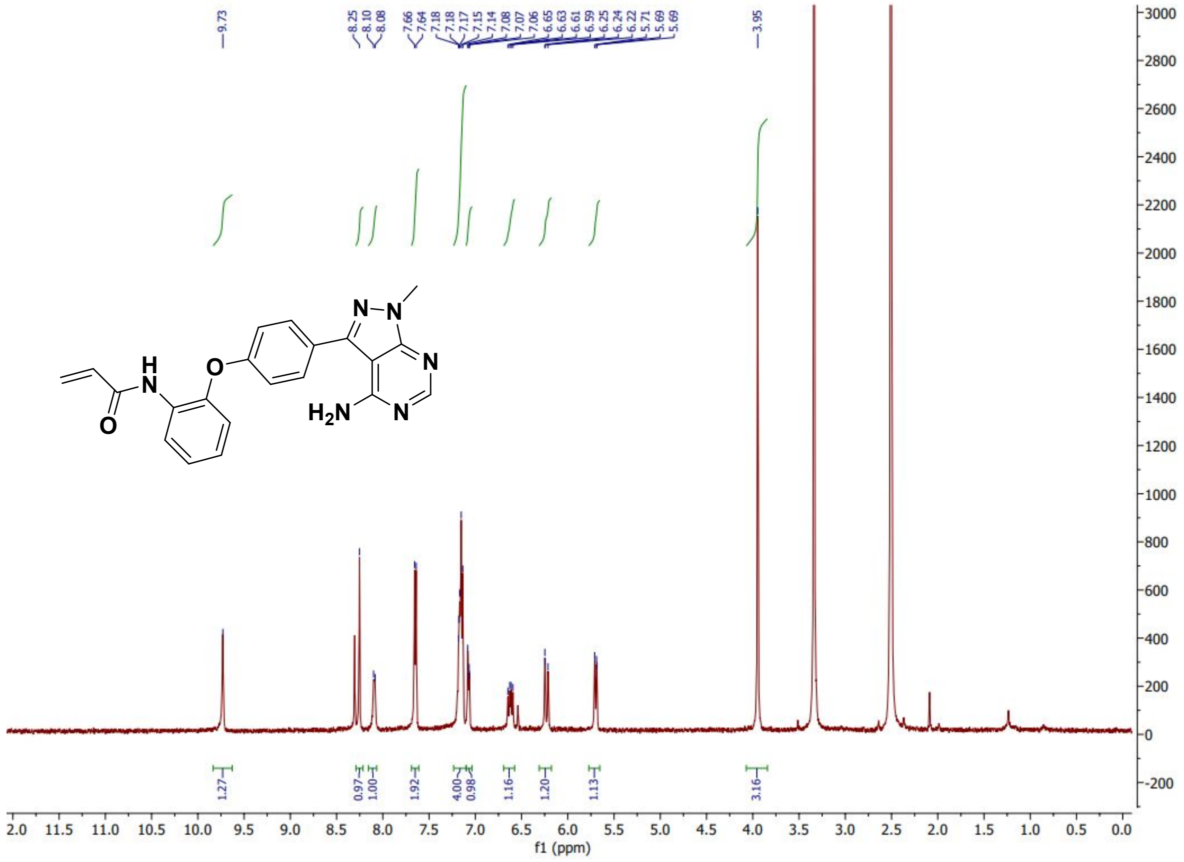

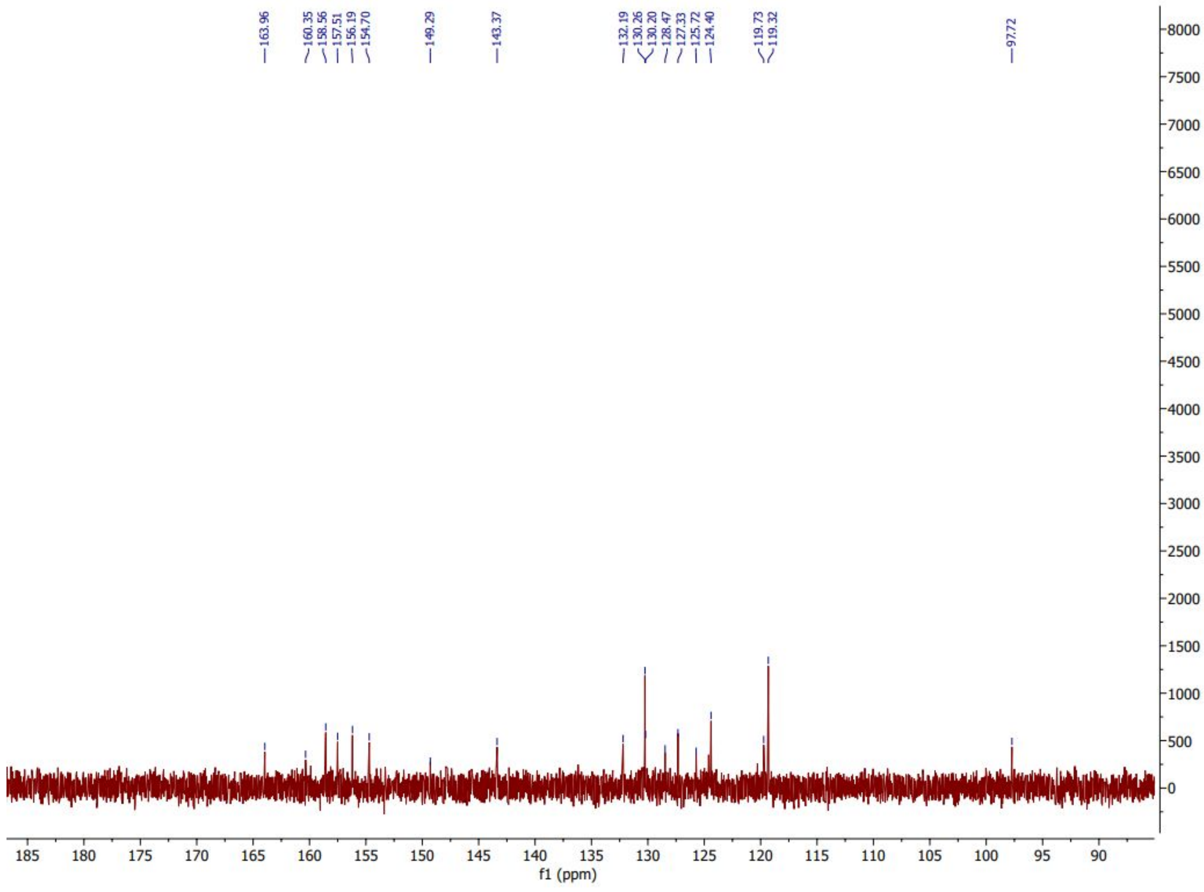

N-[2-(4-{4-Amino-1-methylpyrazolo[3,4-d]pyrimidin-3-yl}phenoxy)phenyl]prop-2-ynamide (S9)

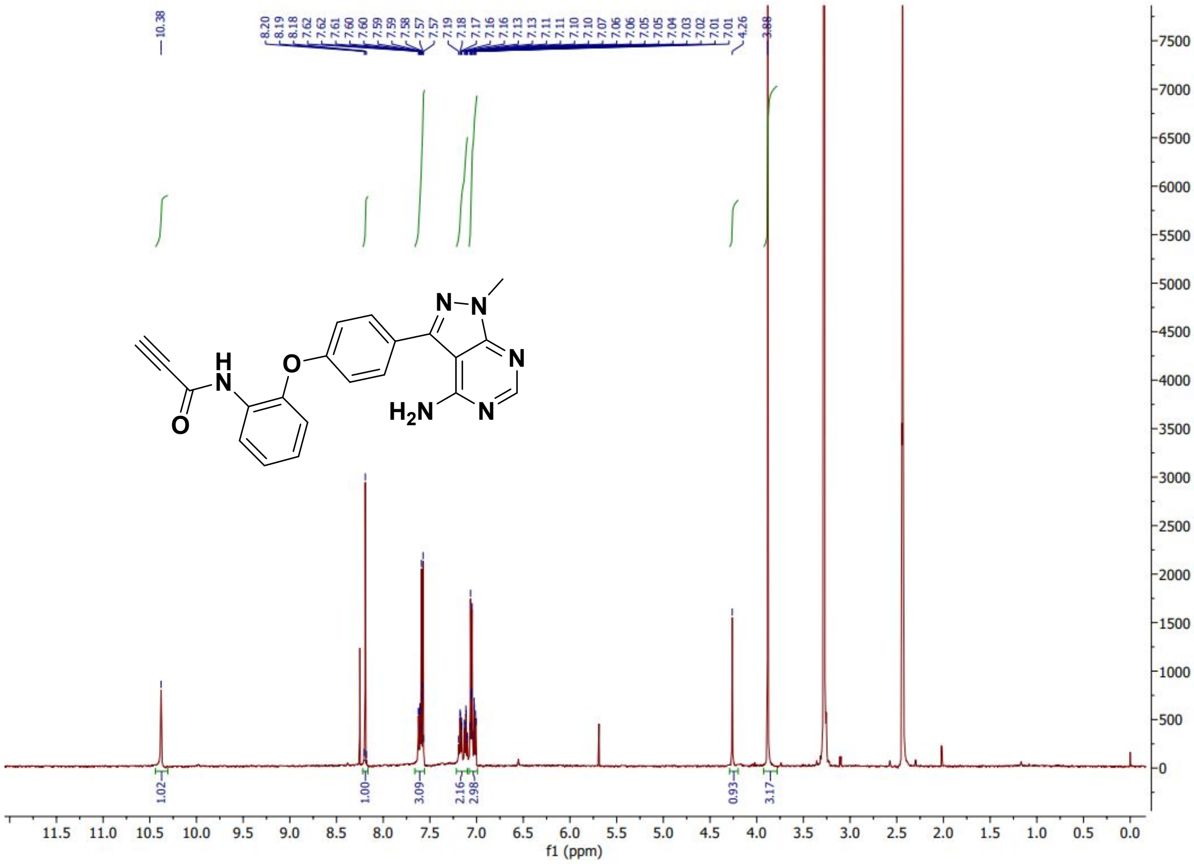

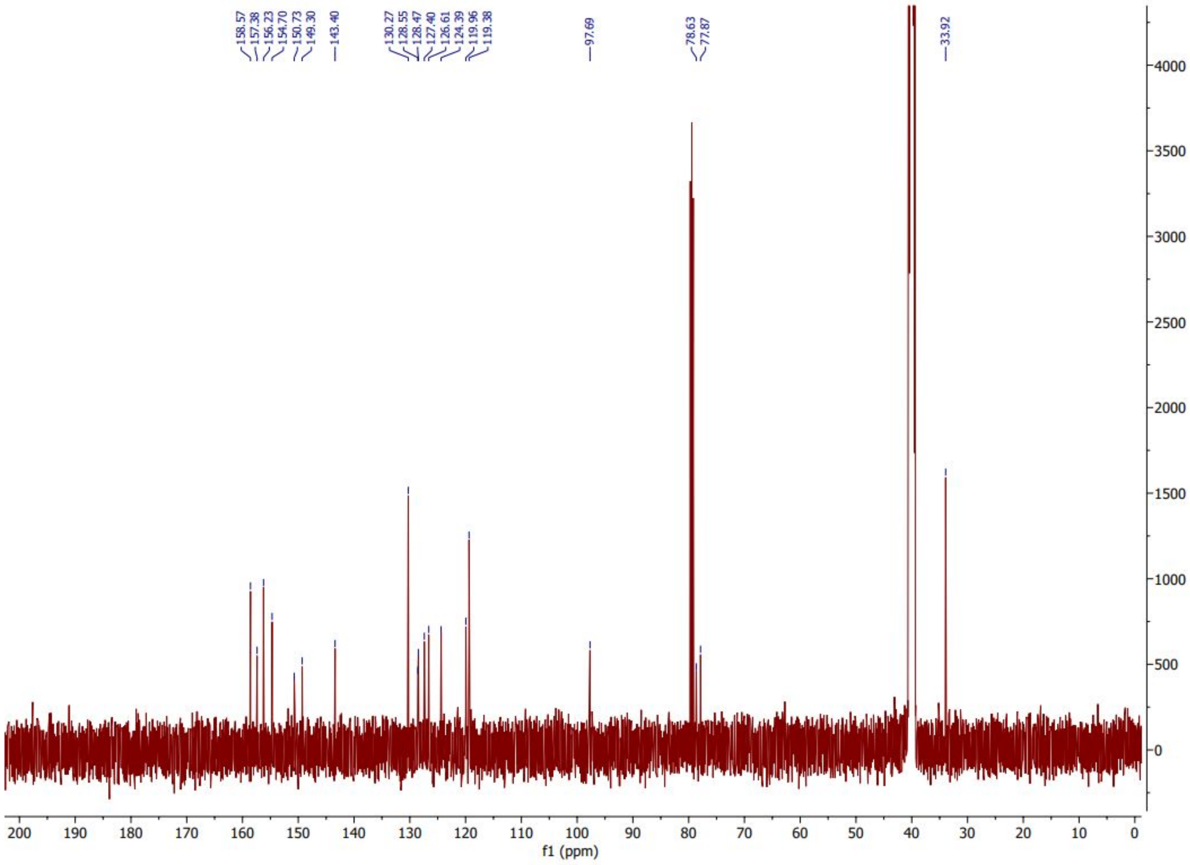

Scaffold C - N-(3-(2-((2-Methoxyphenyl)amino)-7-oxo-8-phenyl-7,8-dihydropyrido [2,3-d]pyrimidin-5-yl)benzyl)acrylamide (S4)

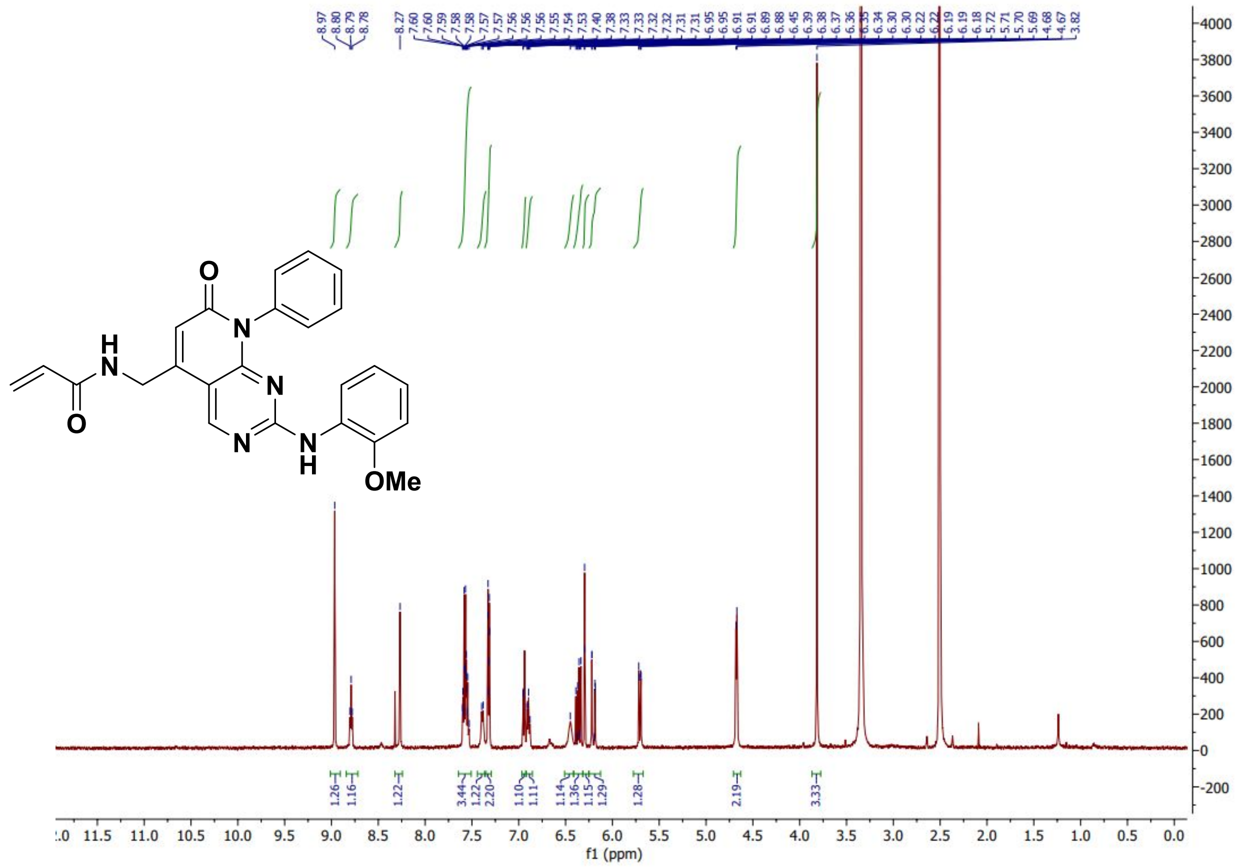

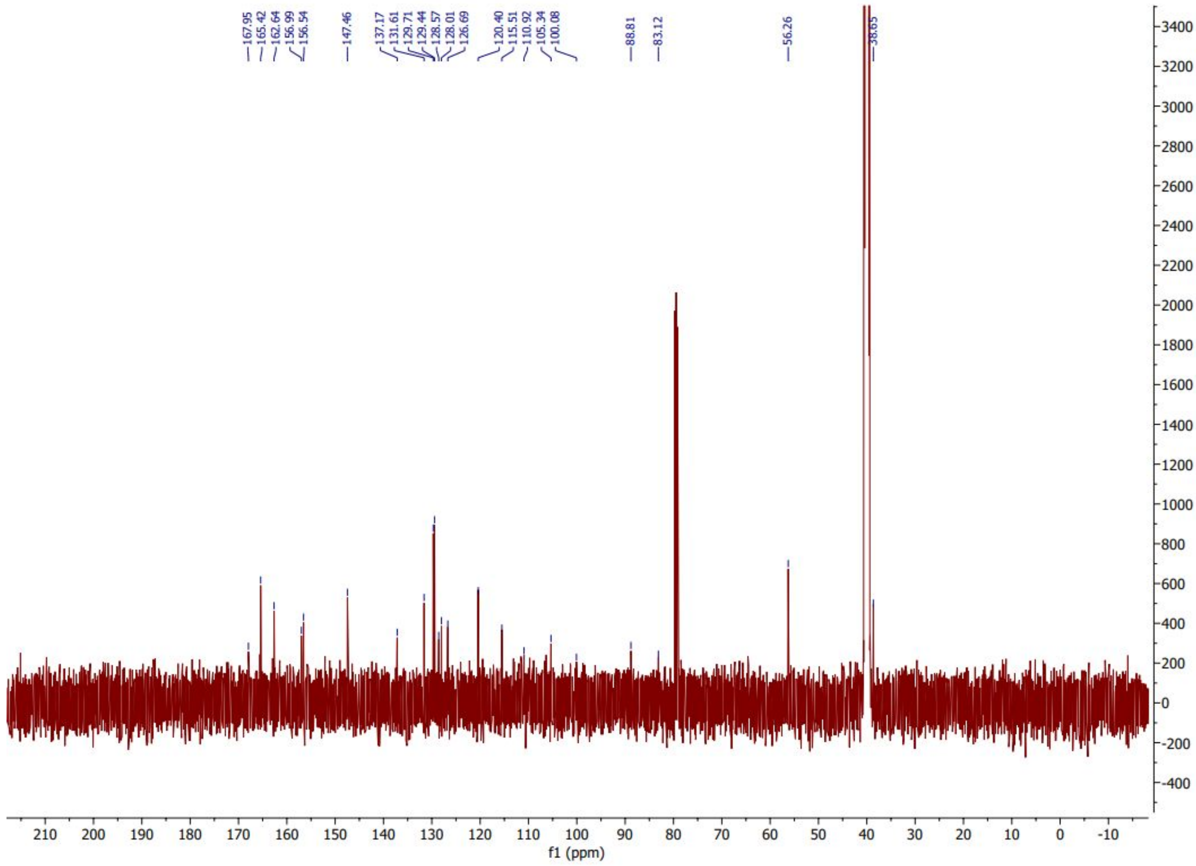

N-((2-((2-Methoxyphenyl)amino)-7-oxo-8-phenyl-7,8-dihydropyrido[2,3-d]pyrimidin-5-yl)methyl)propiolamide (S5)

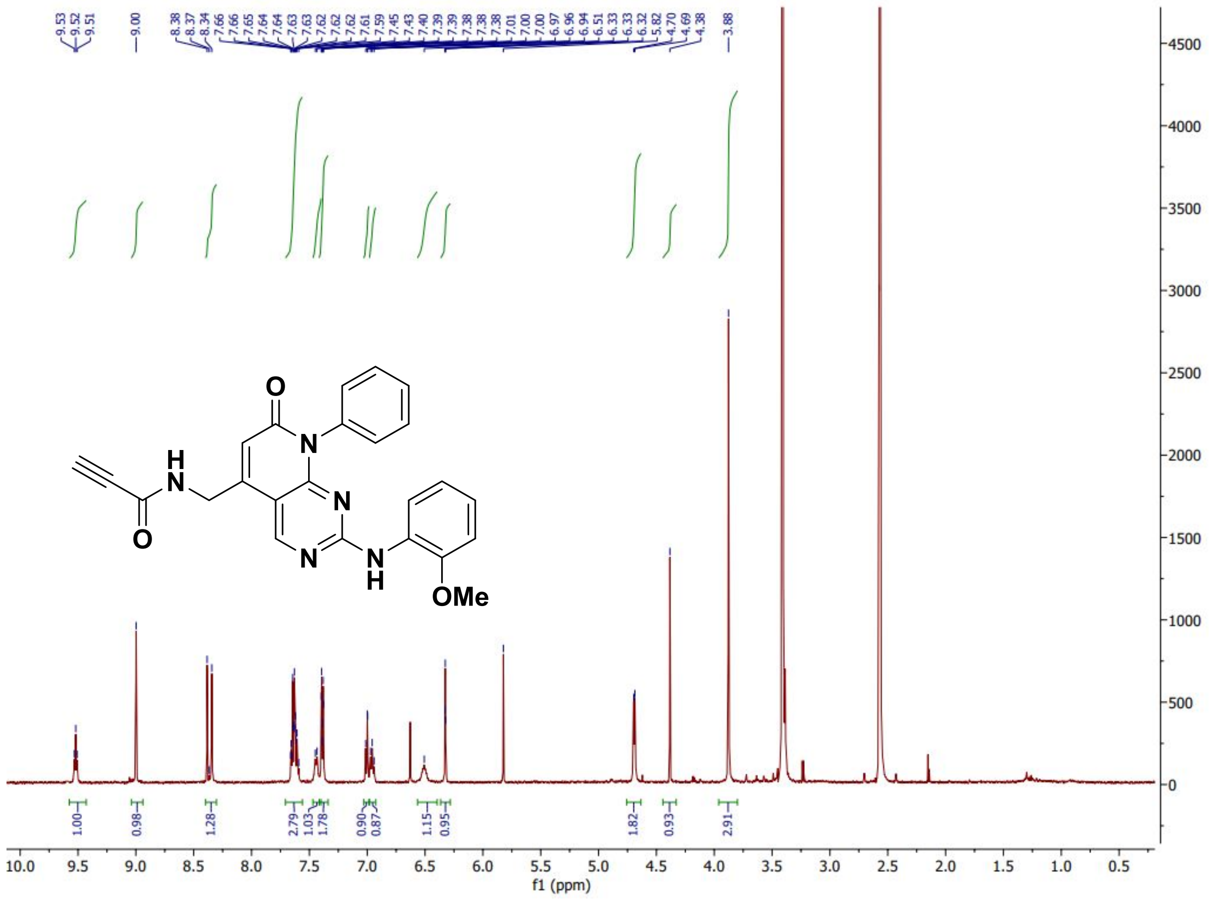

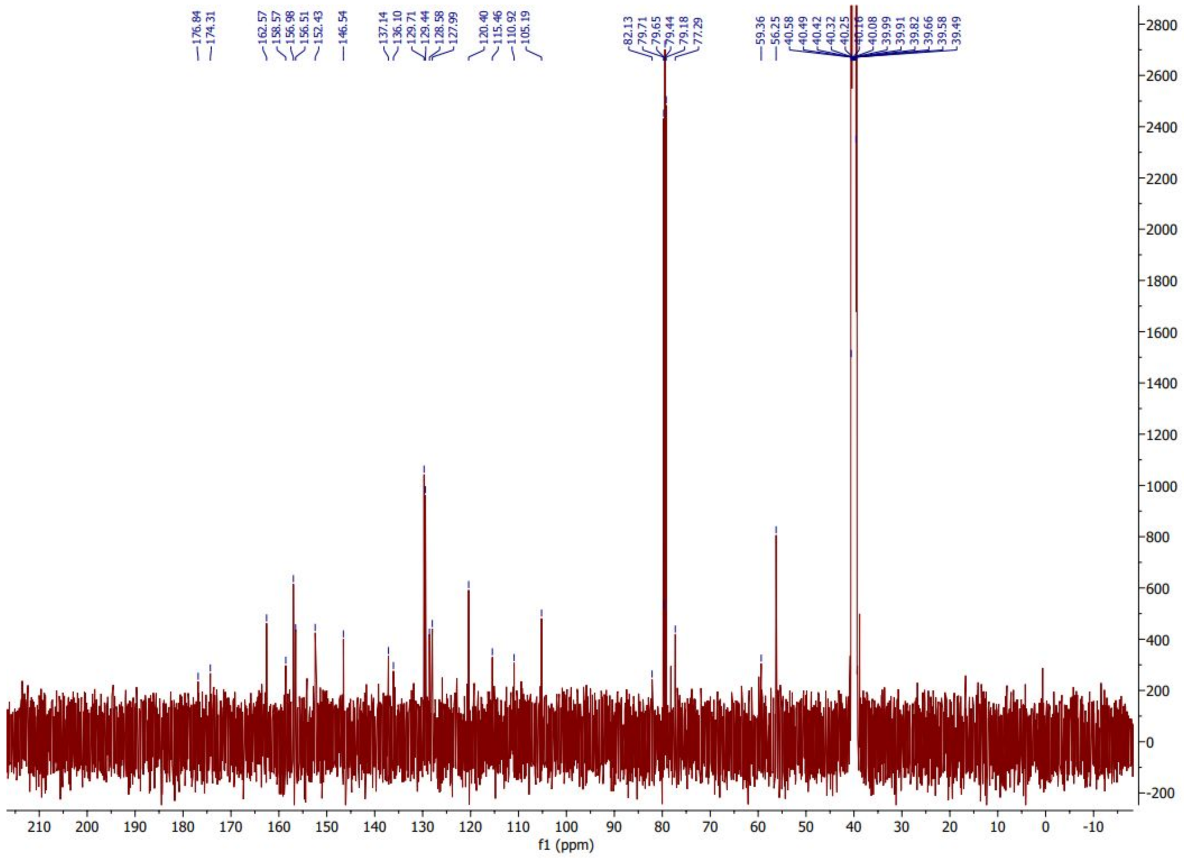

5-Ethynyl-2-[(2-methoxyphenyl)amino]-8-phenylpyrido[2,3-*d*]pyrimidin-7-one (3)

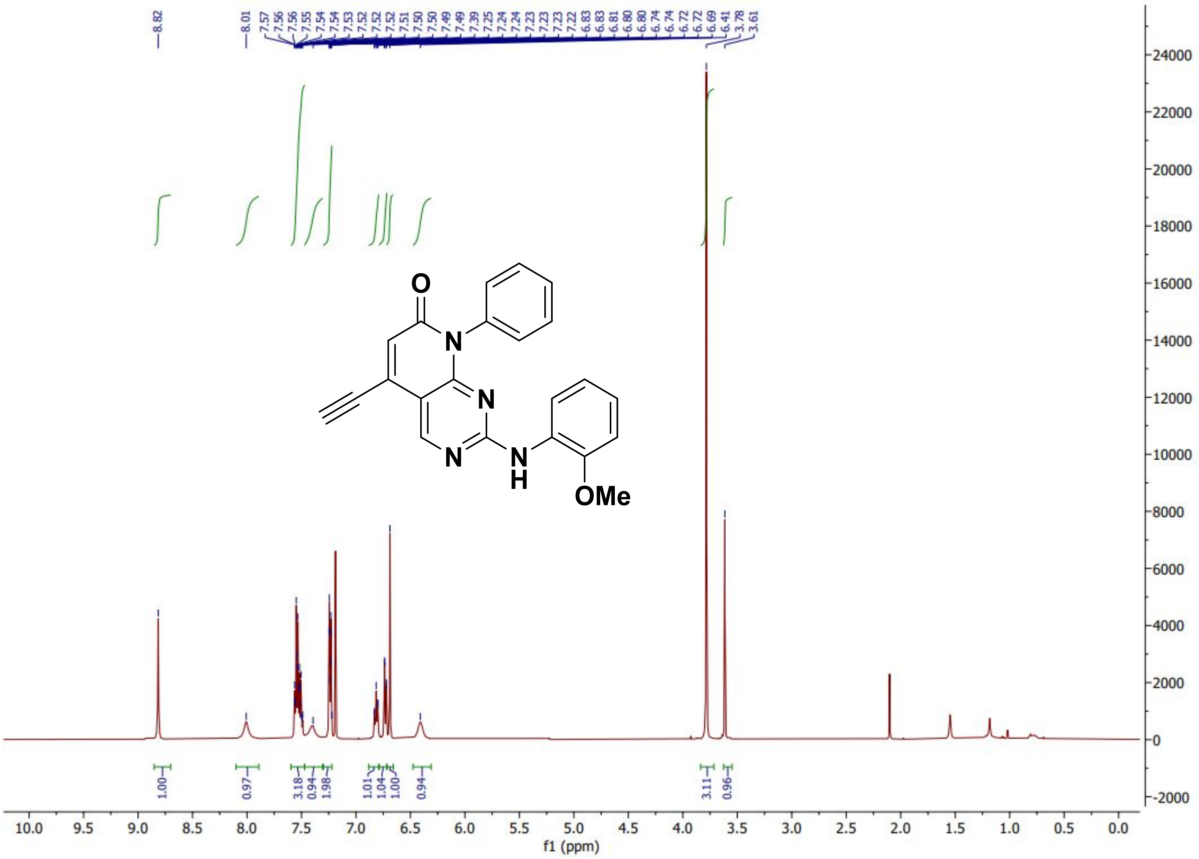

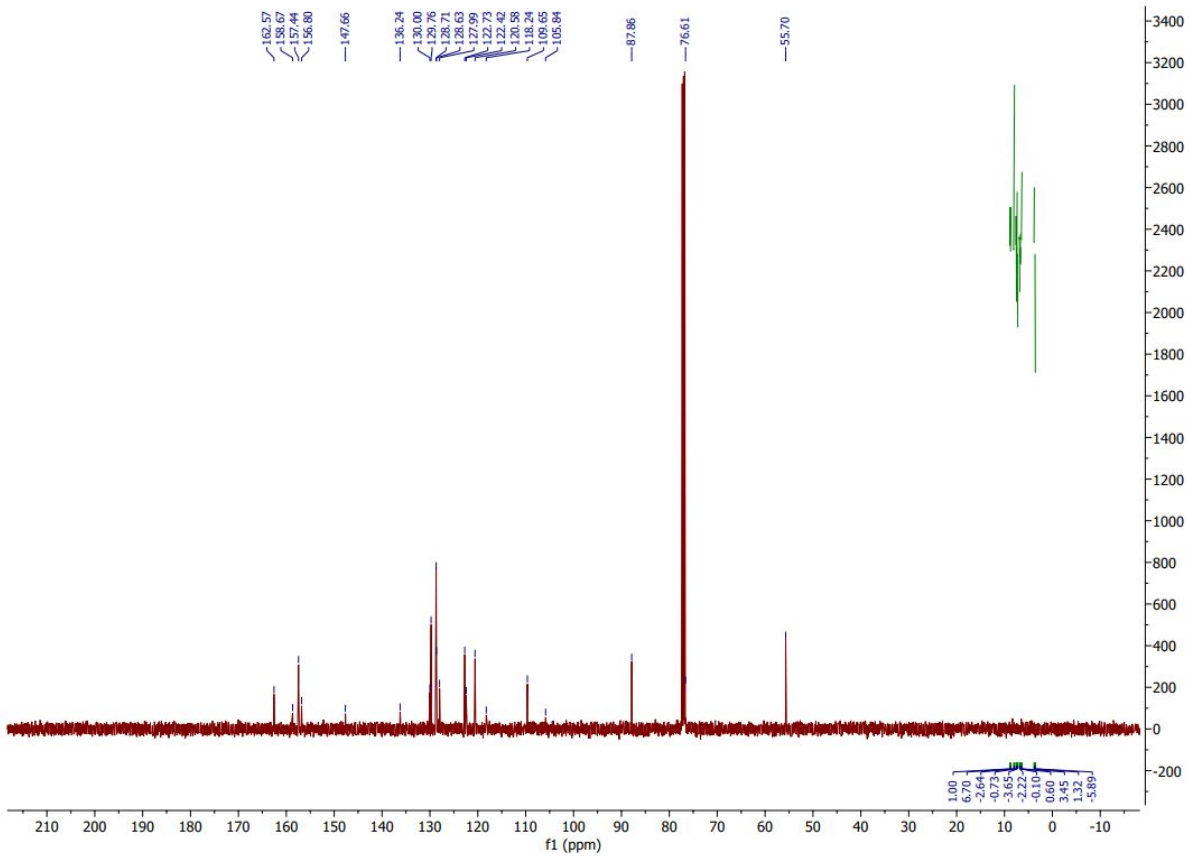

2-((2-methoxyphenyl)amino)-8-phenylpyrido[2,3-*d*]pyrimidin-7(8*H*)-one (S10)

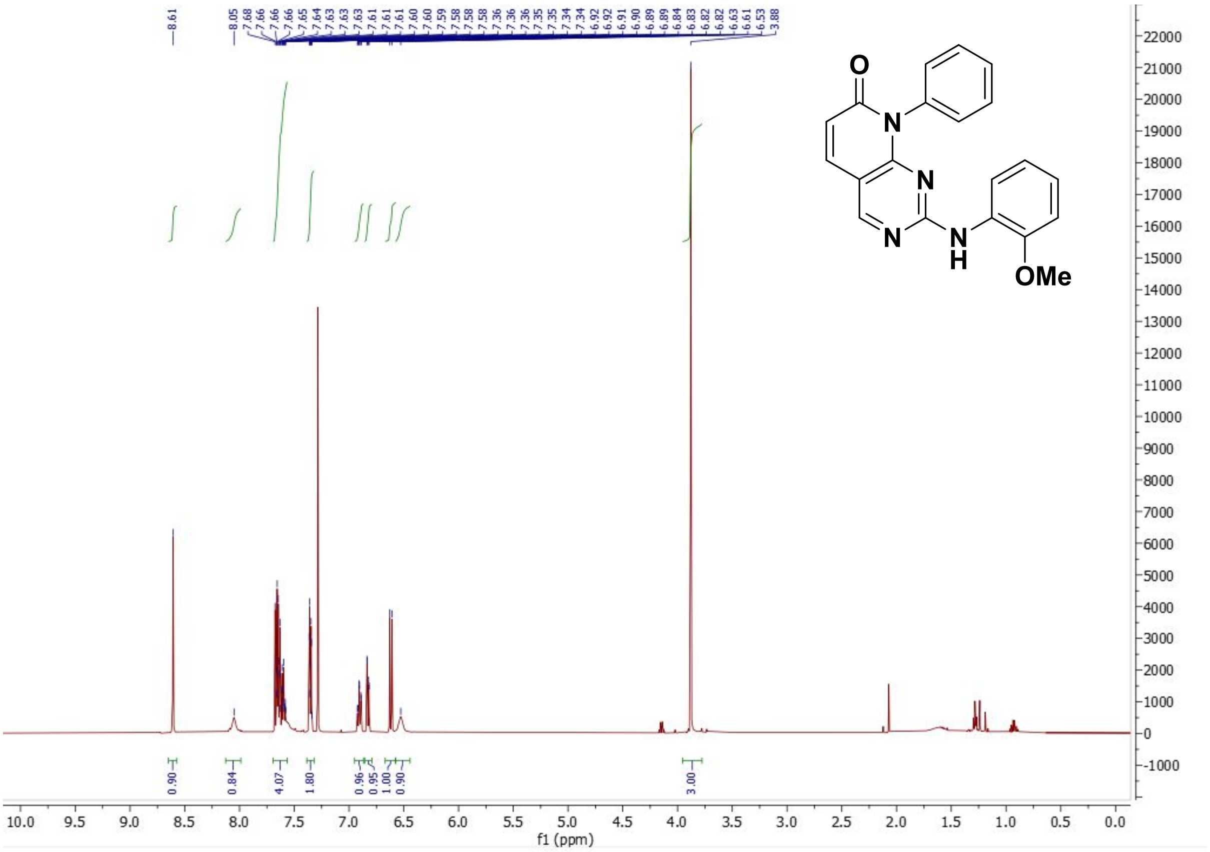

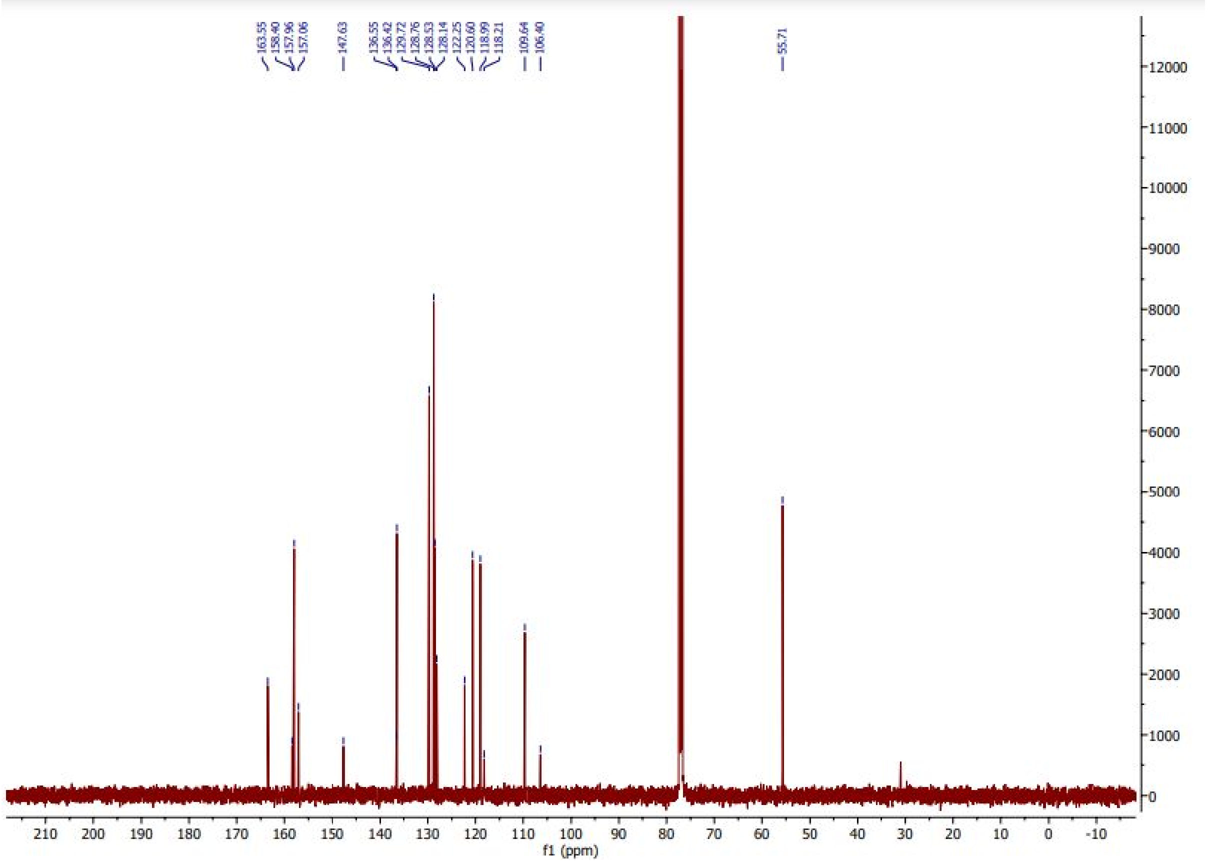

2-((2-Methoxyphenyl)amino)-8-phenyl-5-vinylpyrido[2,3-d]pyrimidin-7(8H)-one (S11)

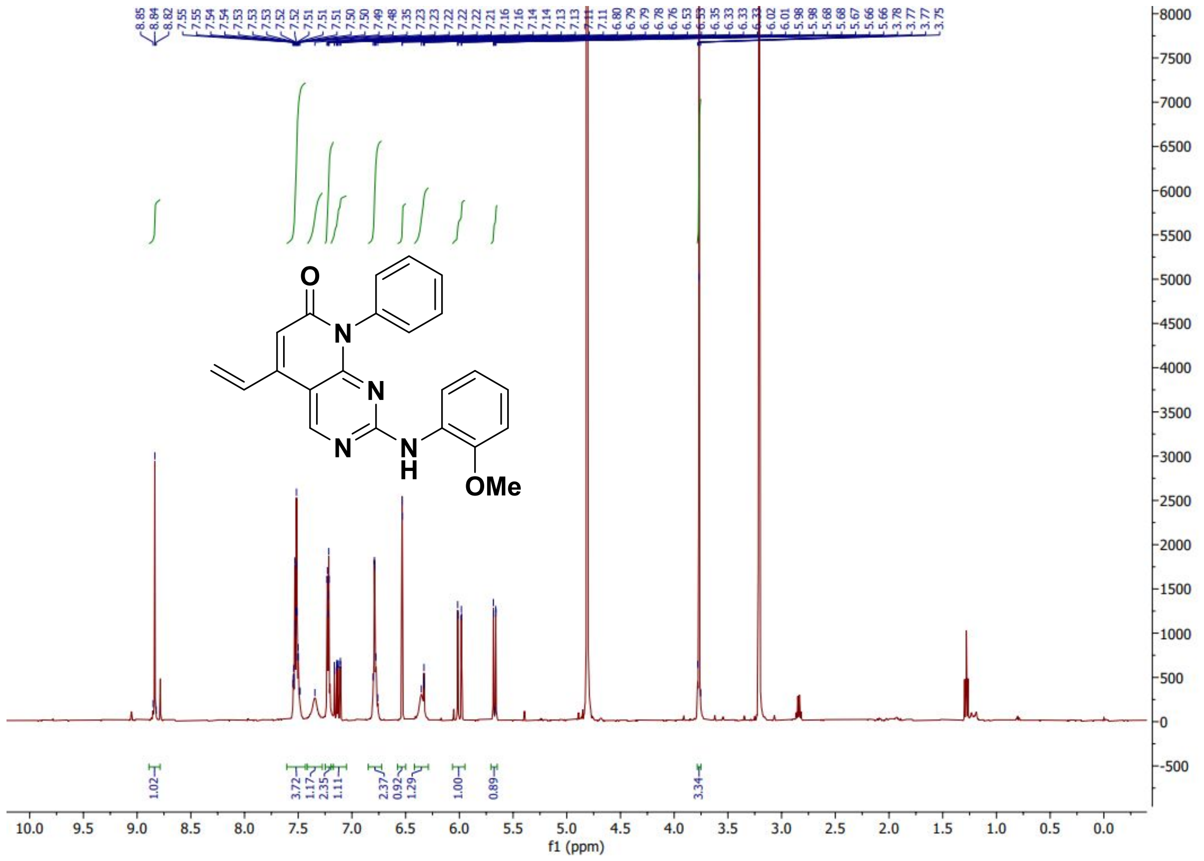

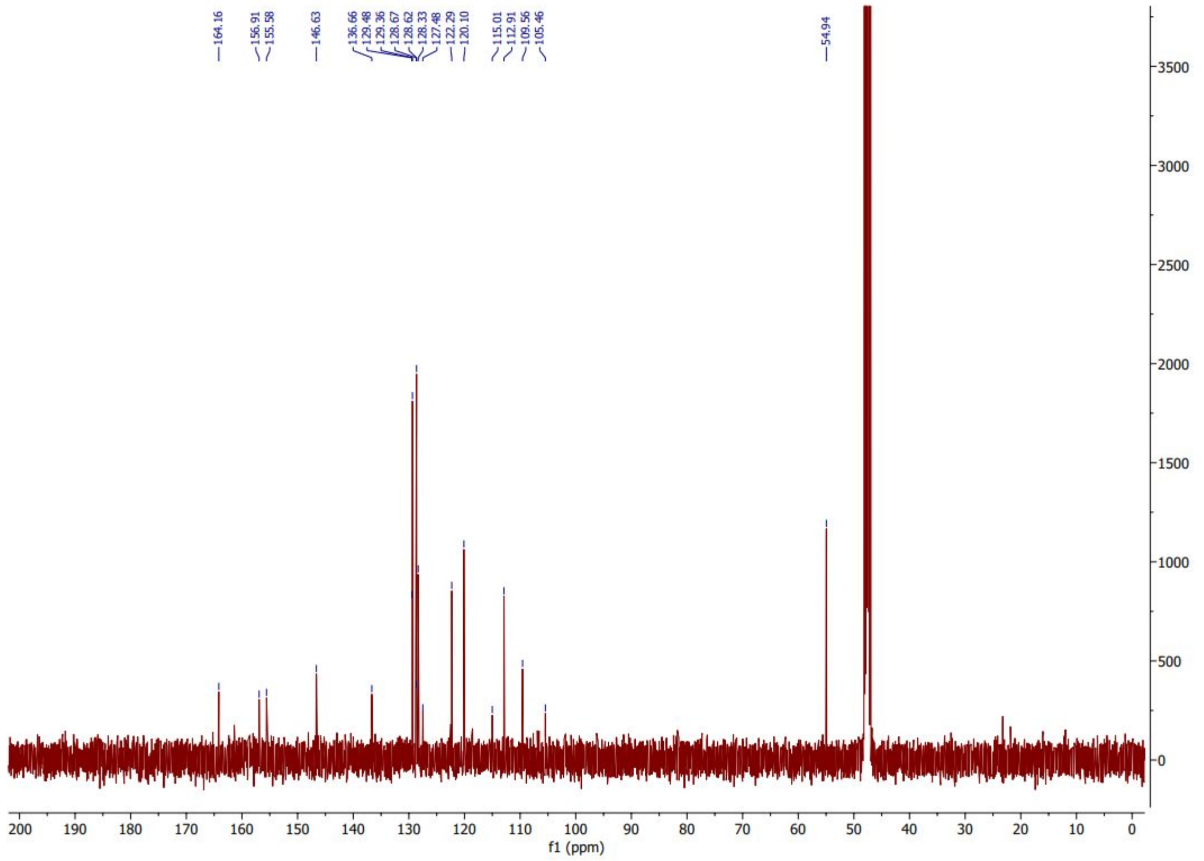

2-((2-Methoxyphenyl)amino)- 8-phenyl-5-(prop-1-yn-1-yl)pyrido[2,3-d]pyrimidin-7(8H)-one (S12)

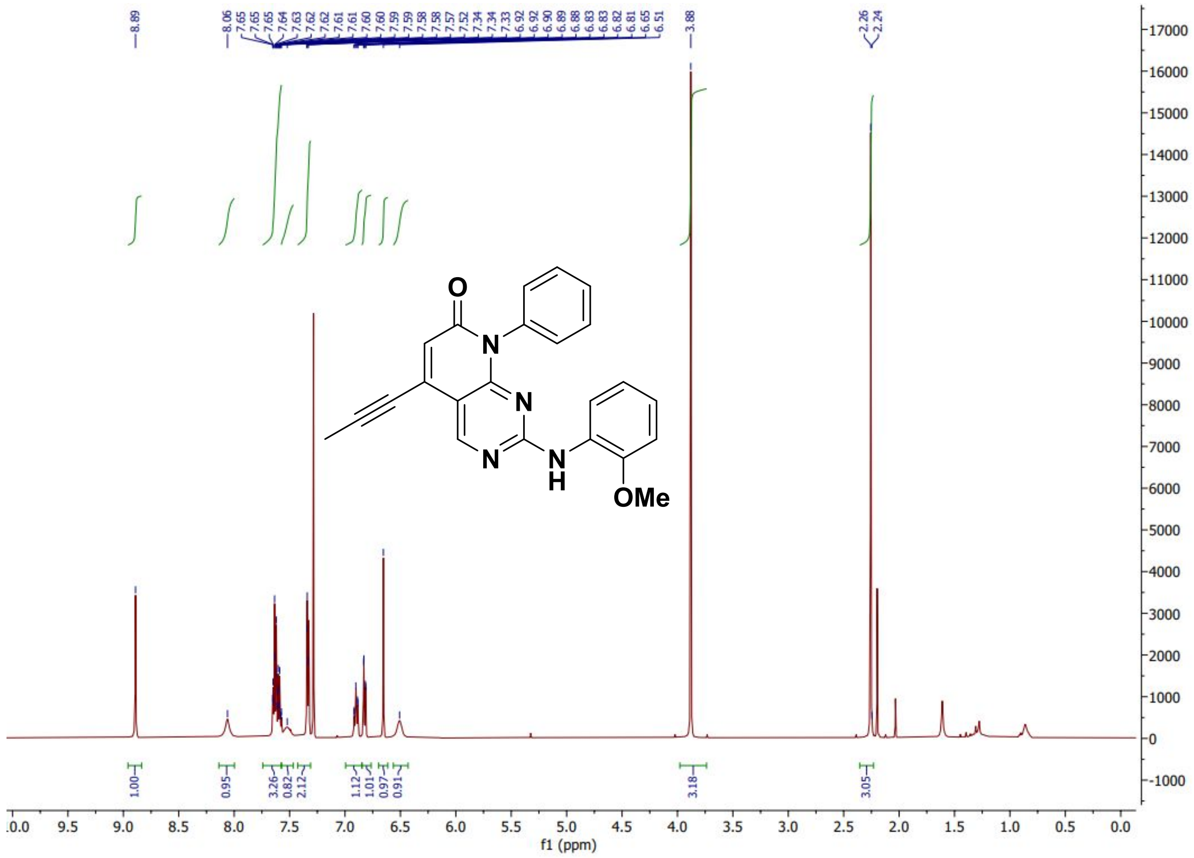

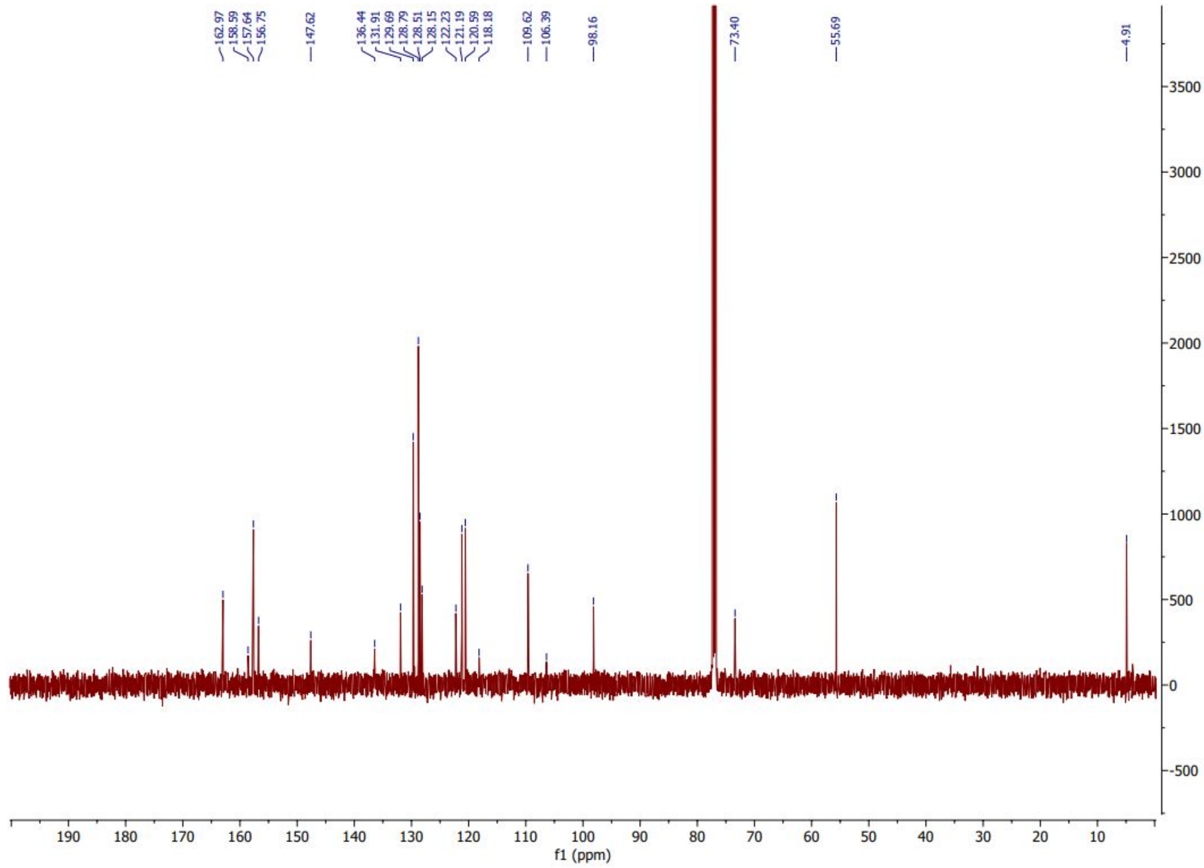

5-Ethynyl-2-((2-methoxyphenyl)amino)-8-phenylpyrido[2,3-*d*]pyrimidin-7(8*H*)-one (4)

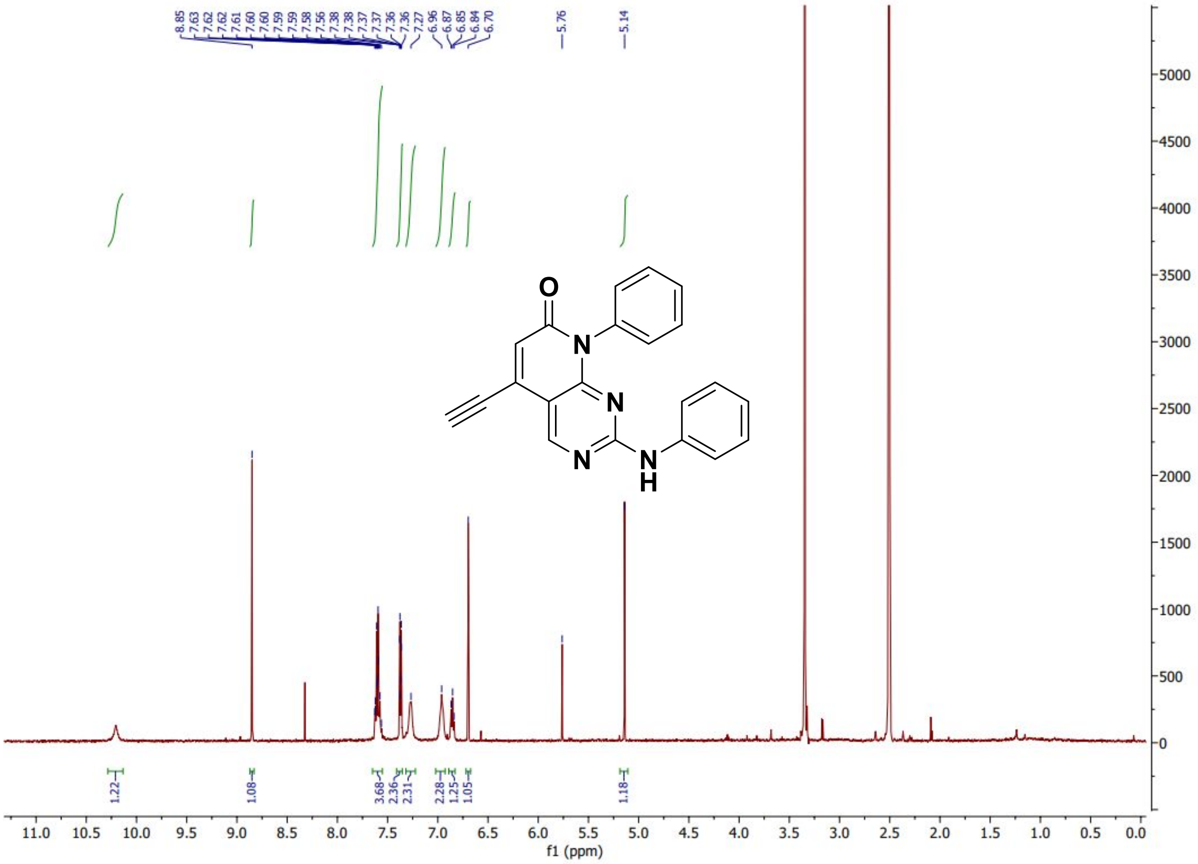

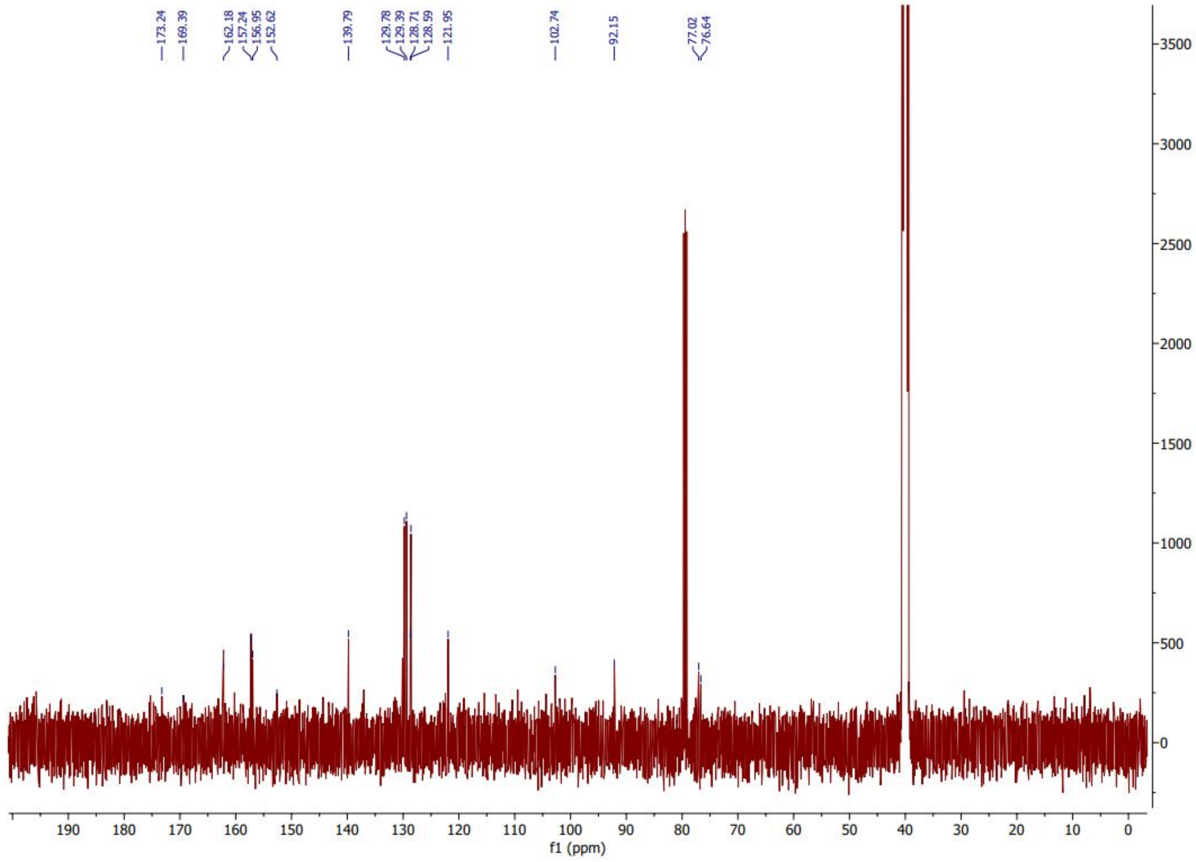

5-Ethynyl-2-((2-methoxy-4-morpholinophenyl)amino)-8-phenylpyrido[2,3-*d*]pyrimidin-7(8*H*)-one (5)

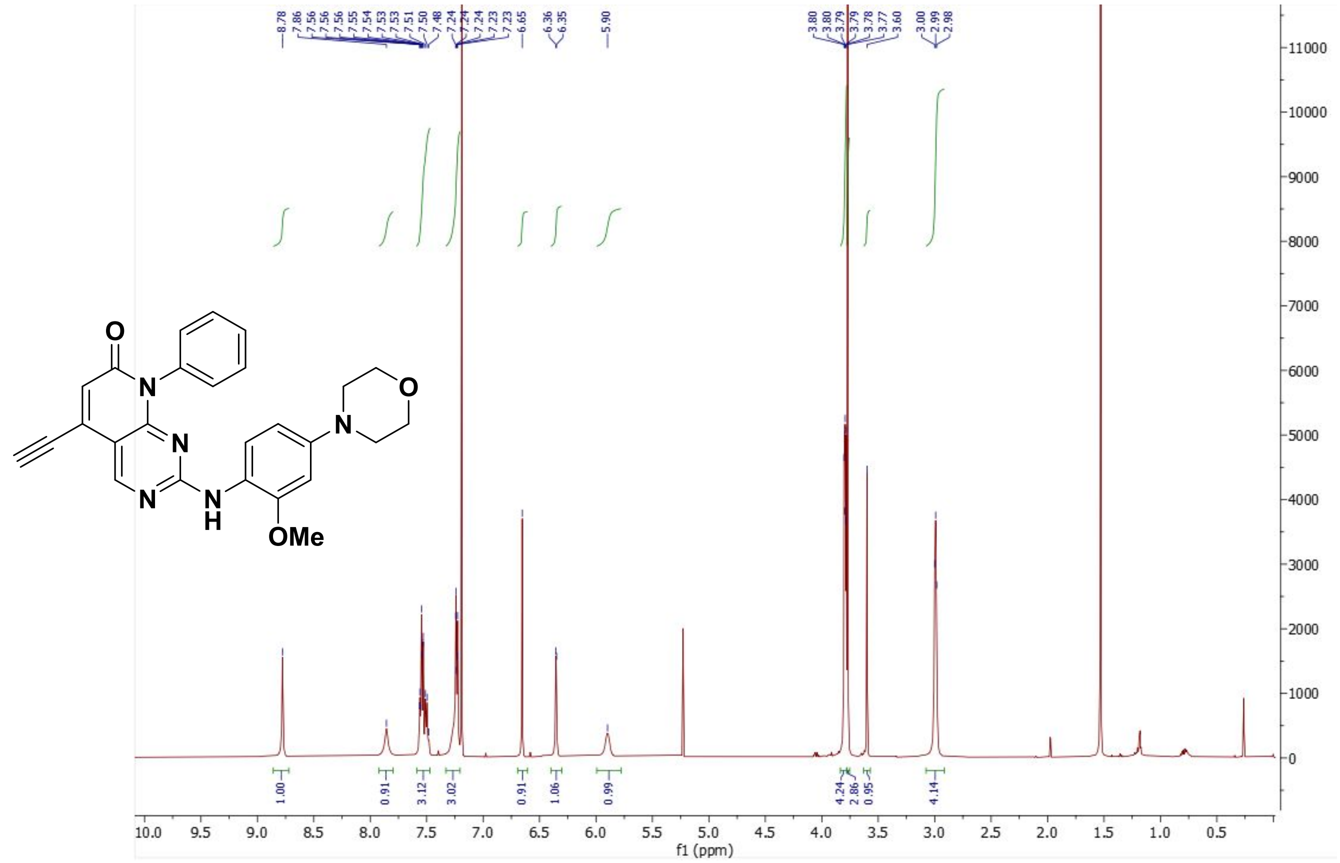

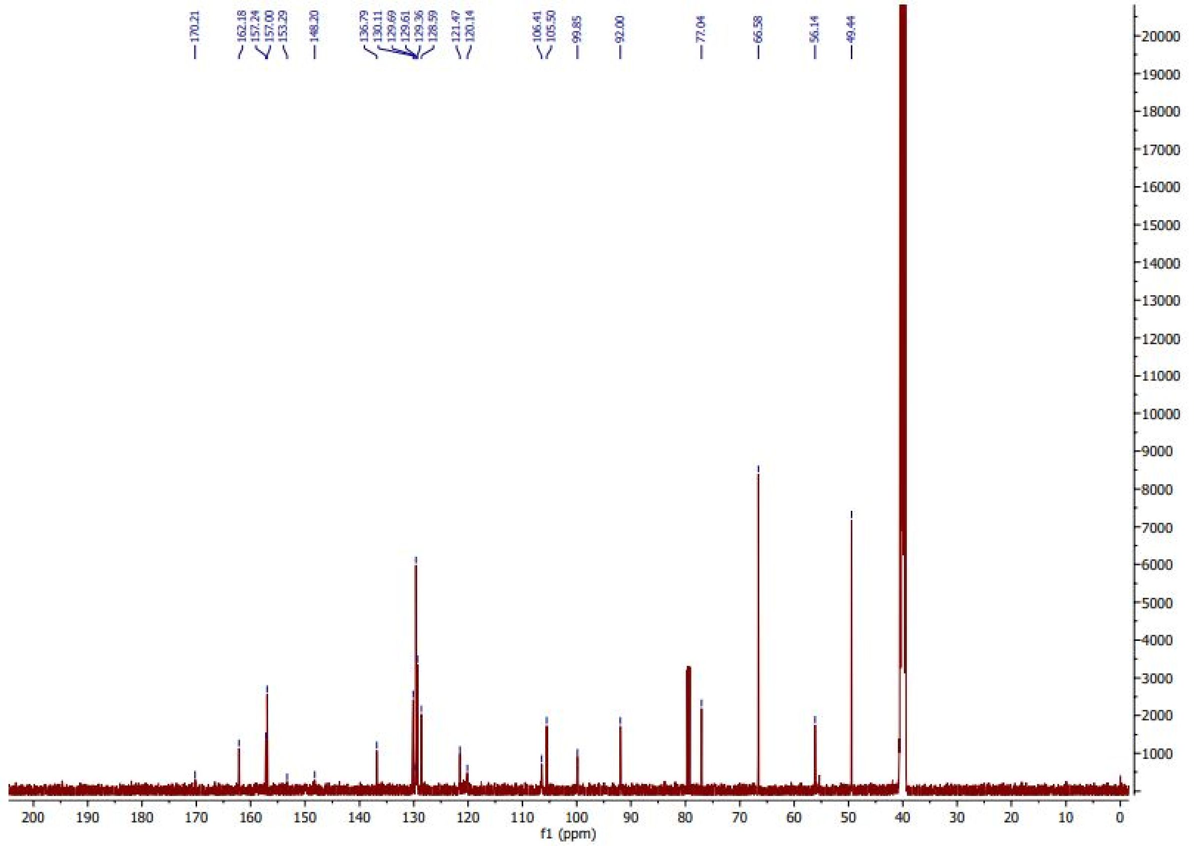

5-Ethynyl-2-((4-morpholinophenyl)amino)-8-phenylpyrido[2,3-*d*]pyrimidin-7(8*H*)-one (6)

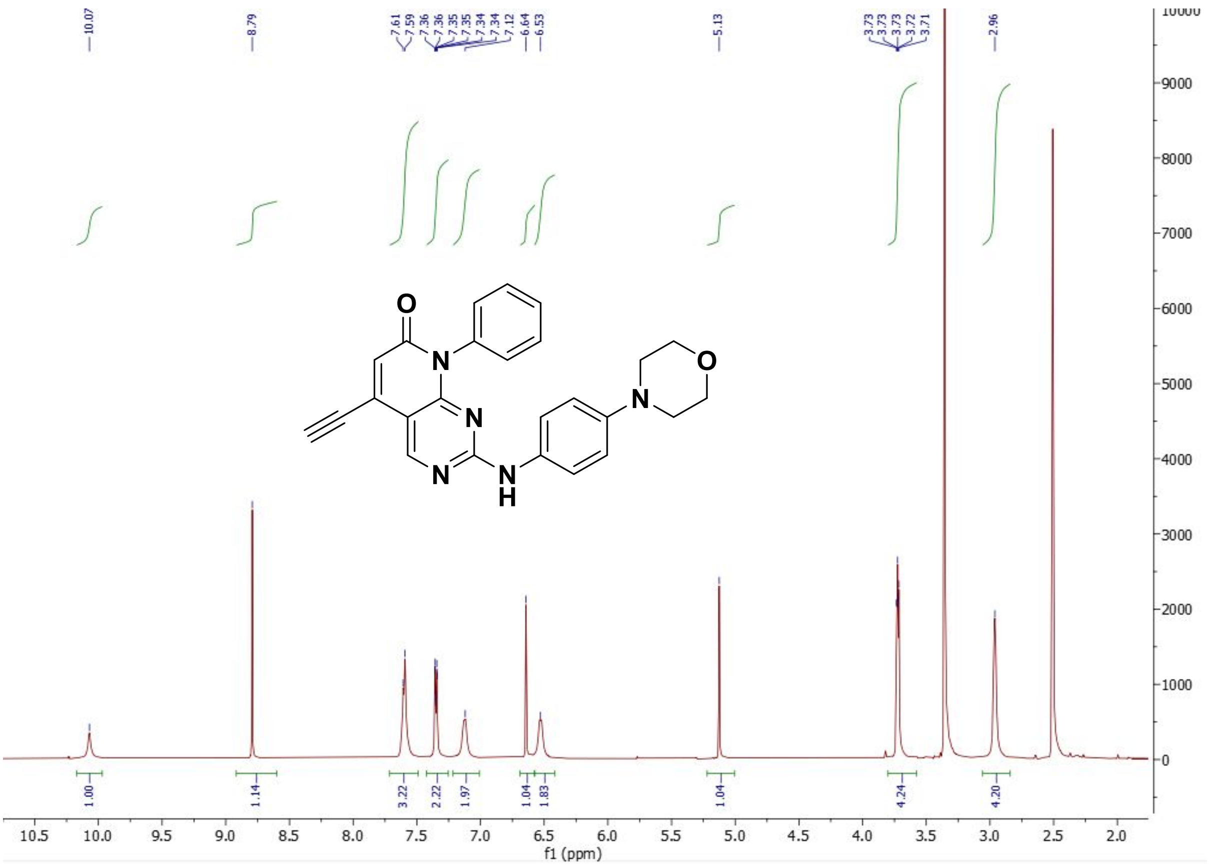

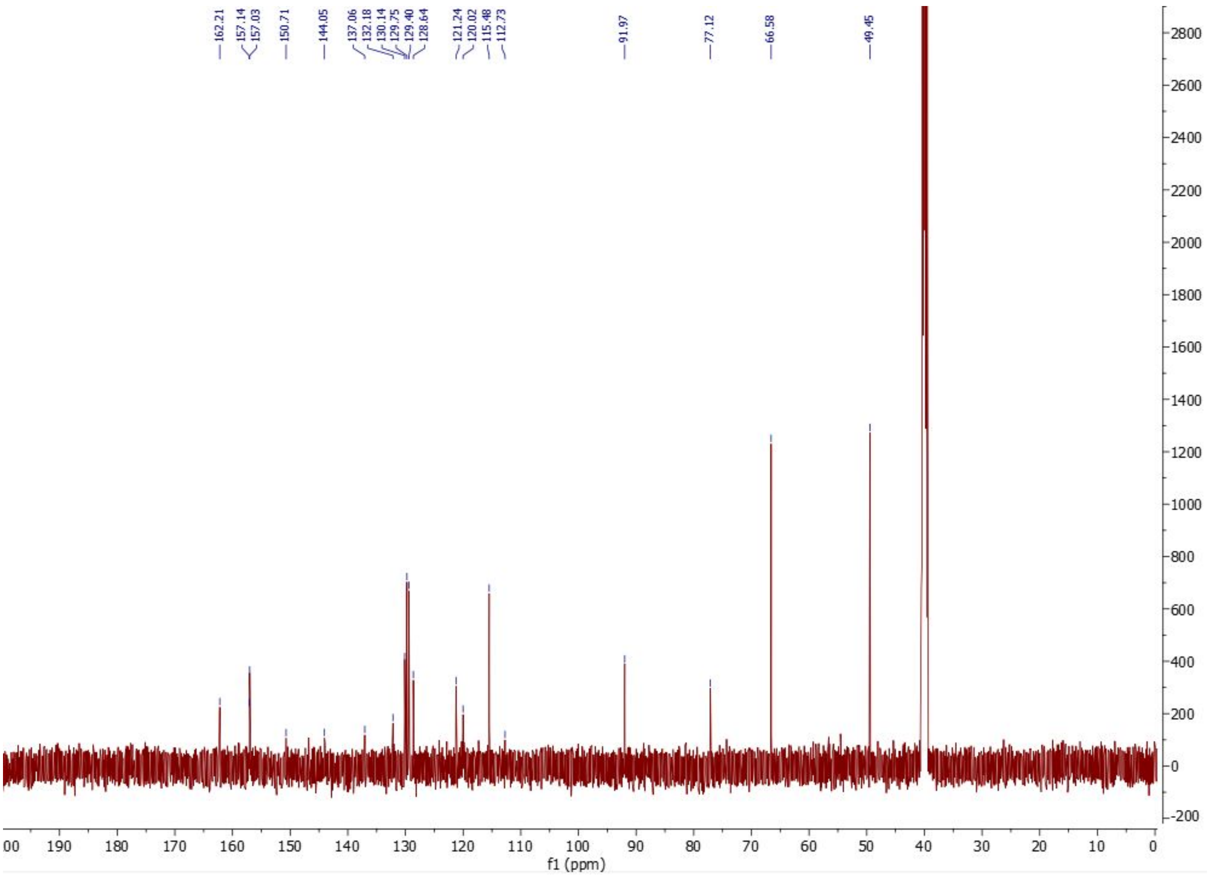

2-((4-((2-(Dimethylamino)ethyl)(methyl)amino)phenyl)amino)-5-ethynyl-8-phenylpyrido[2,3-*d*]pyrimidin-7(8*H*)-one (7)

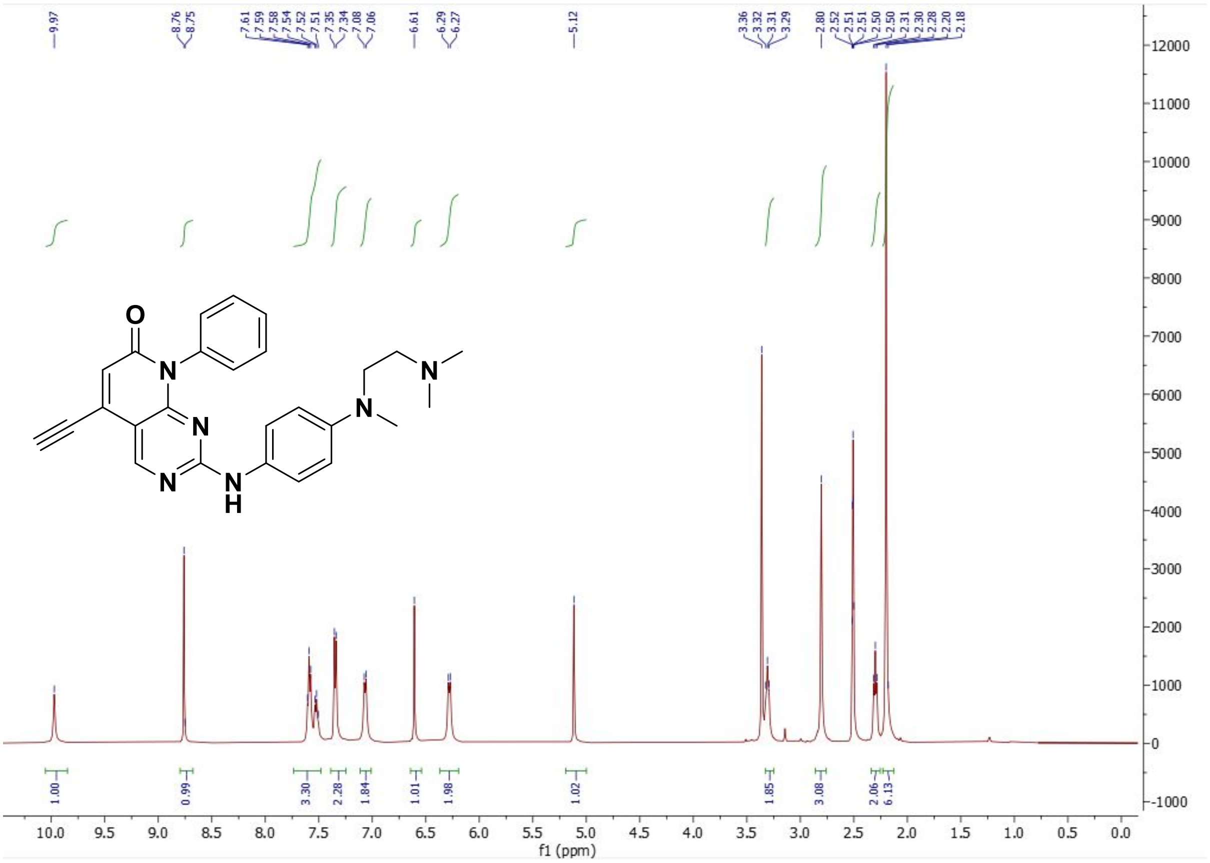

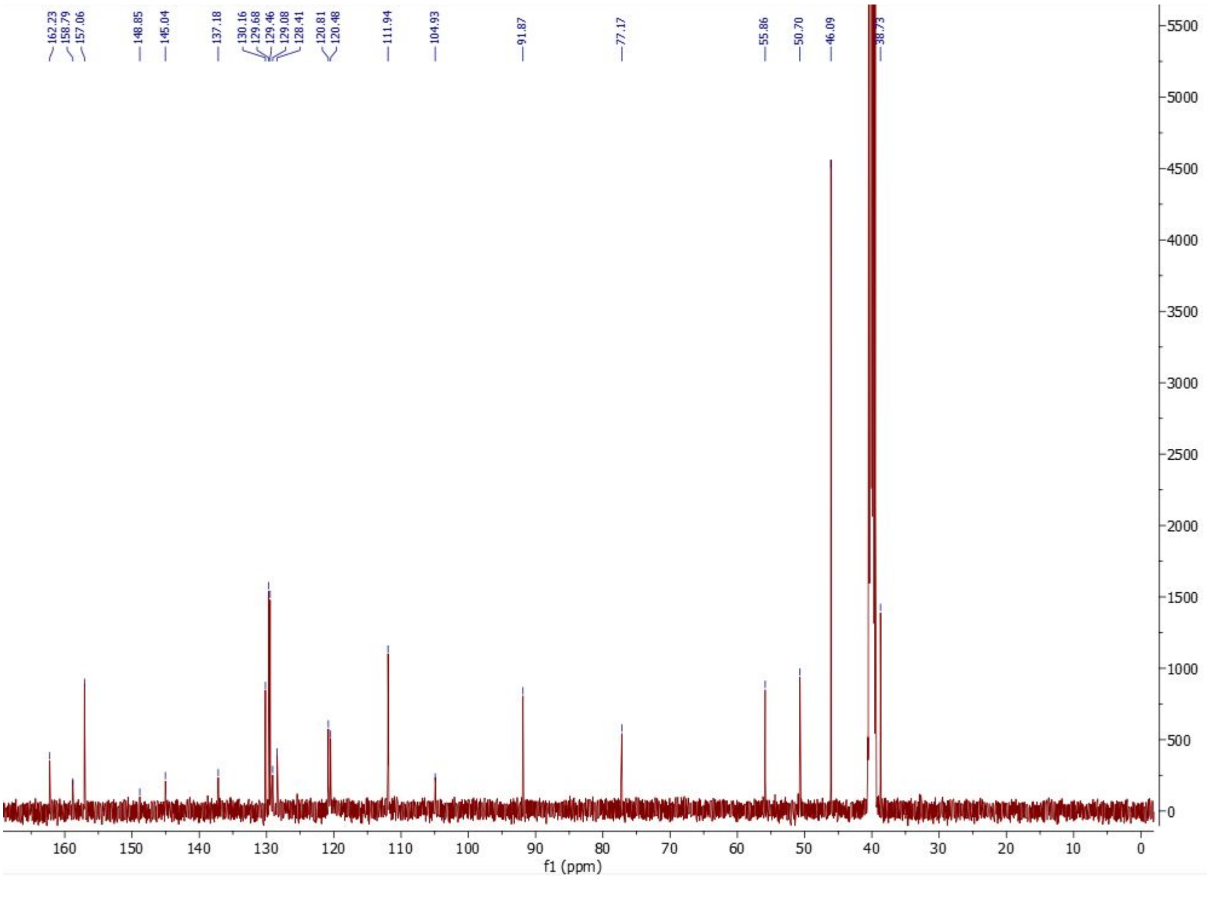

5-Ethynyl-2-((2-methoxy-4-(4-methylpiperazine-1-ylphenyl)amino)-8-phenylpyrido[2,3-*d*]pyrimidin-7(8*H*)-one (8)

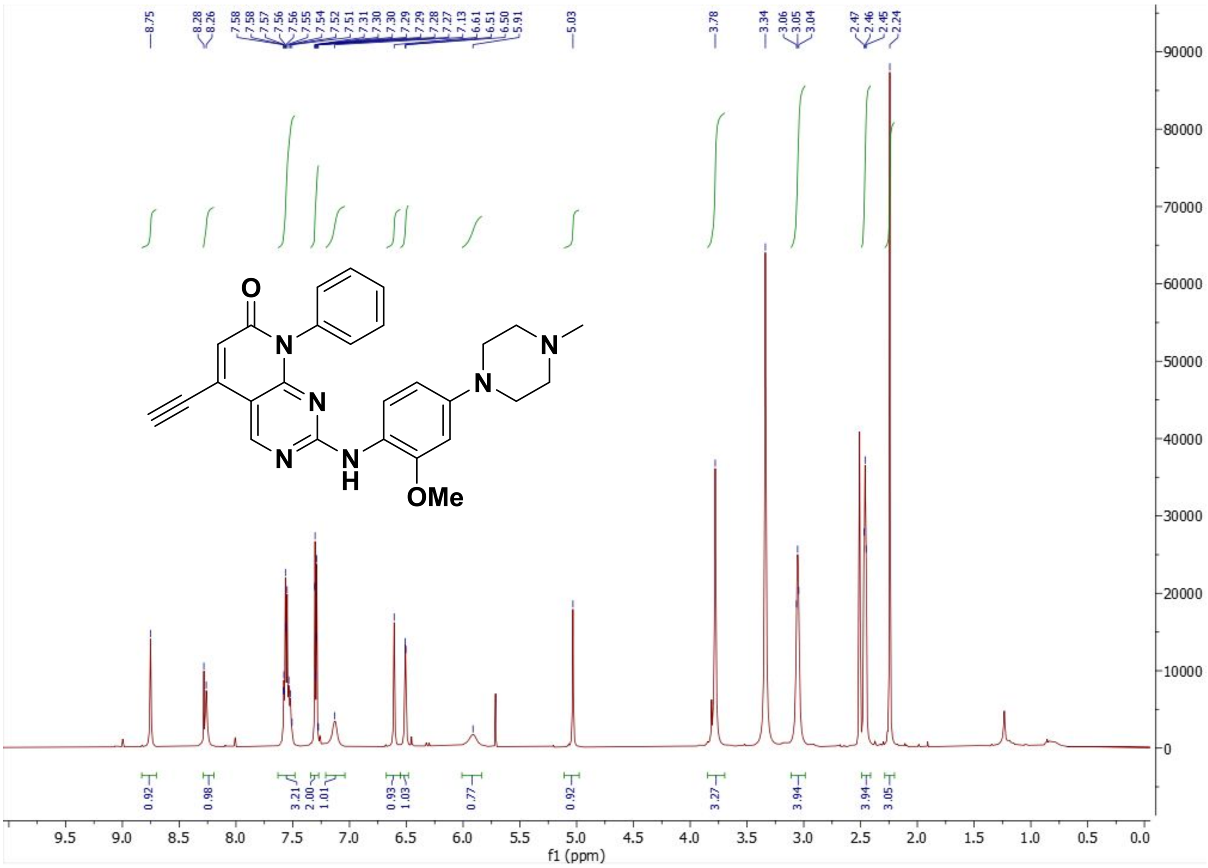

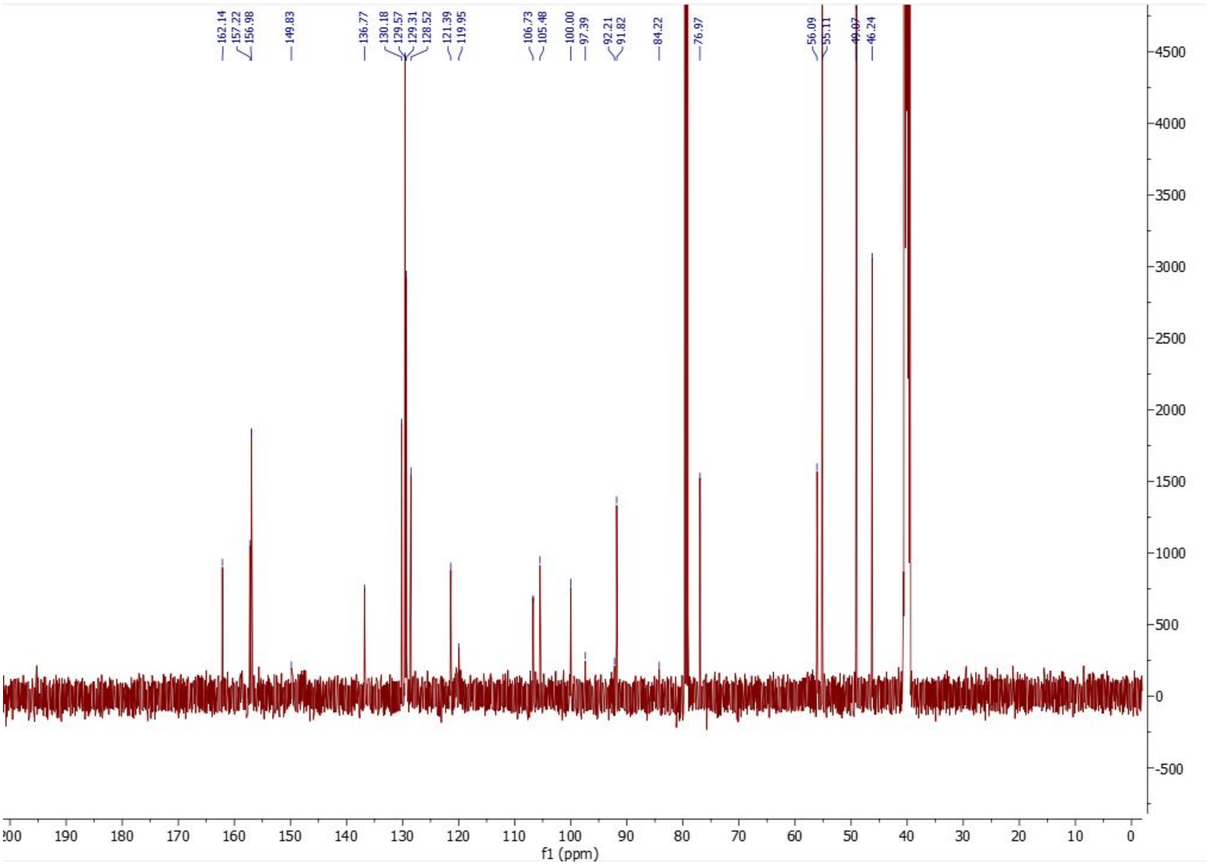

5-Ethynyl-2-((4-(4-methylpiperazin-1-yl)phenyl)amino)-8-phenylpyrido[2,3-d]pyrimidin-7(8H)-one (9)

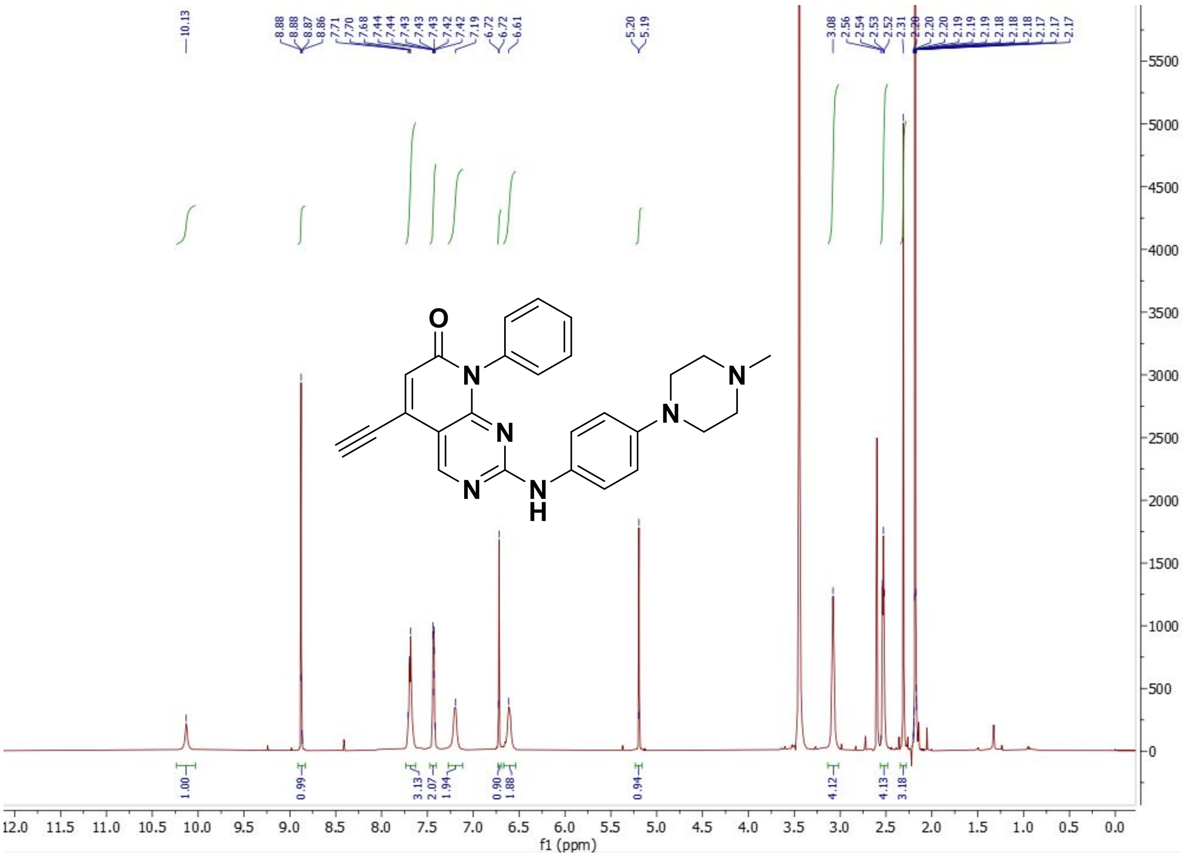

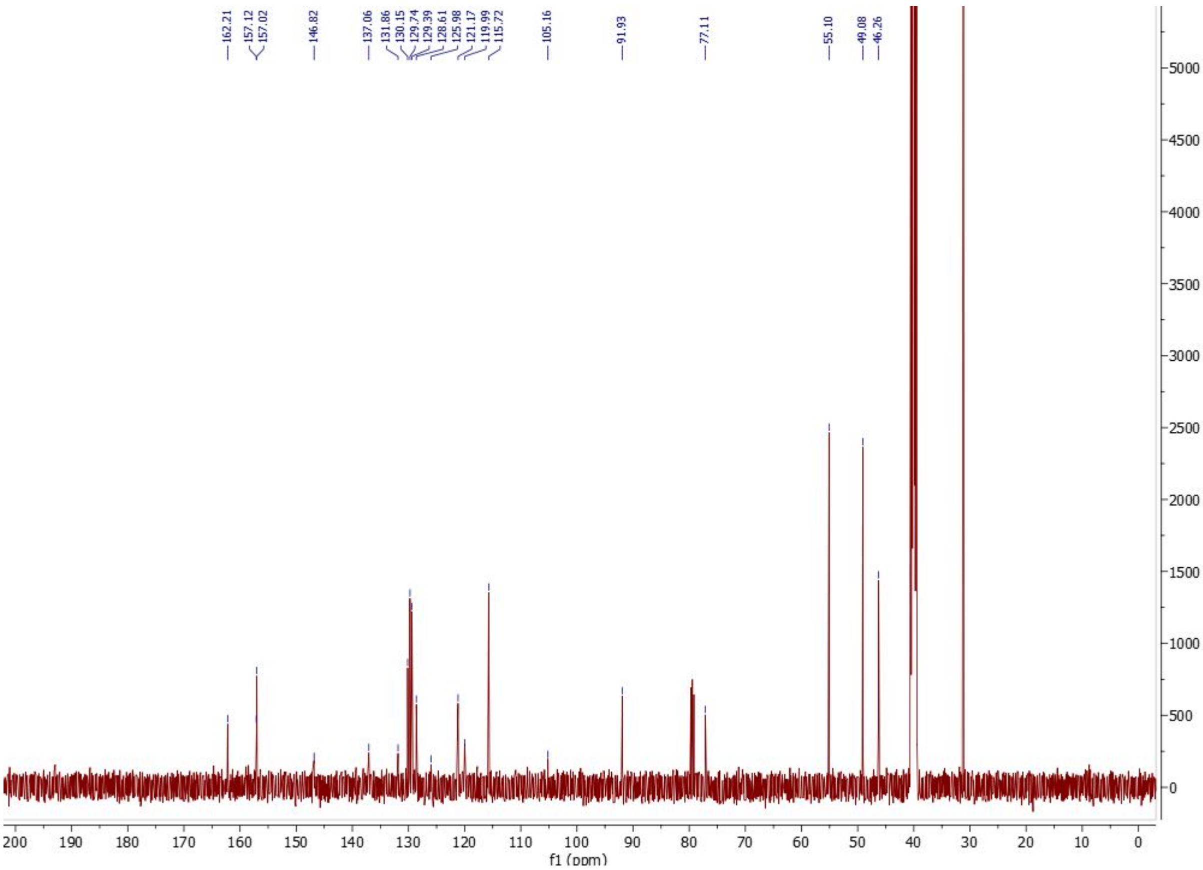

_N_-(4-((5-Ethynyl-7-oxo-8-phenyl-7,8-dihydropyrido[2,3-_d_]pyrimidin-2-yl)amino)-3-methoxyphenyl)-2-methoxy-_N_-methylacetamide (10)

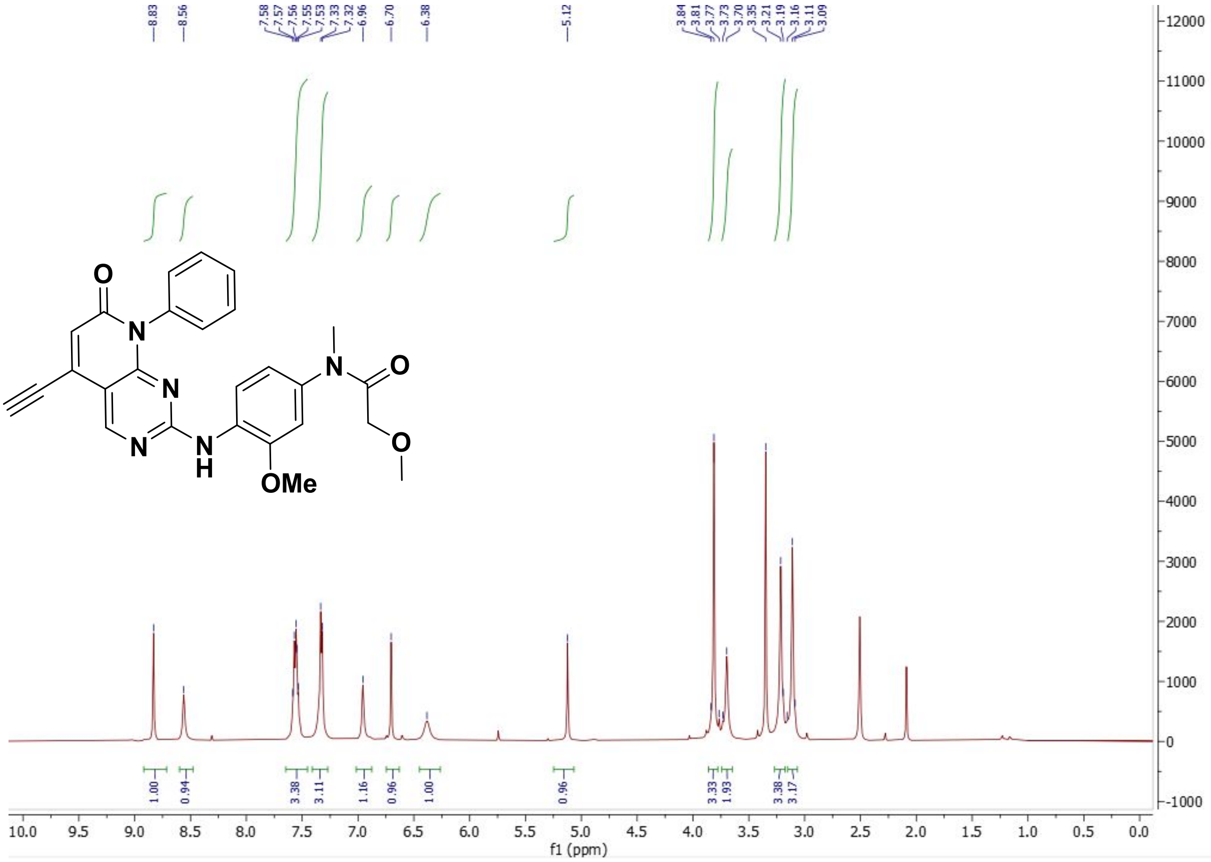

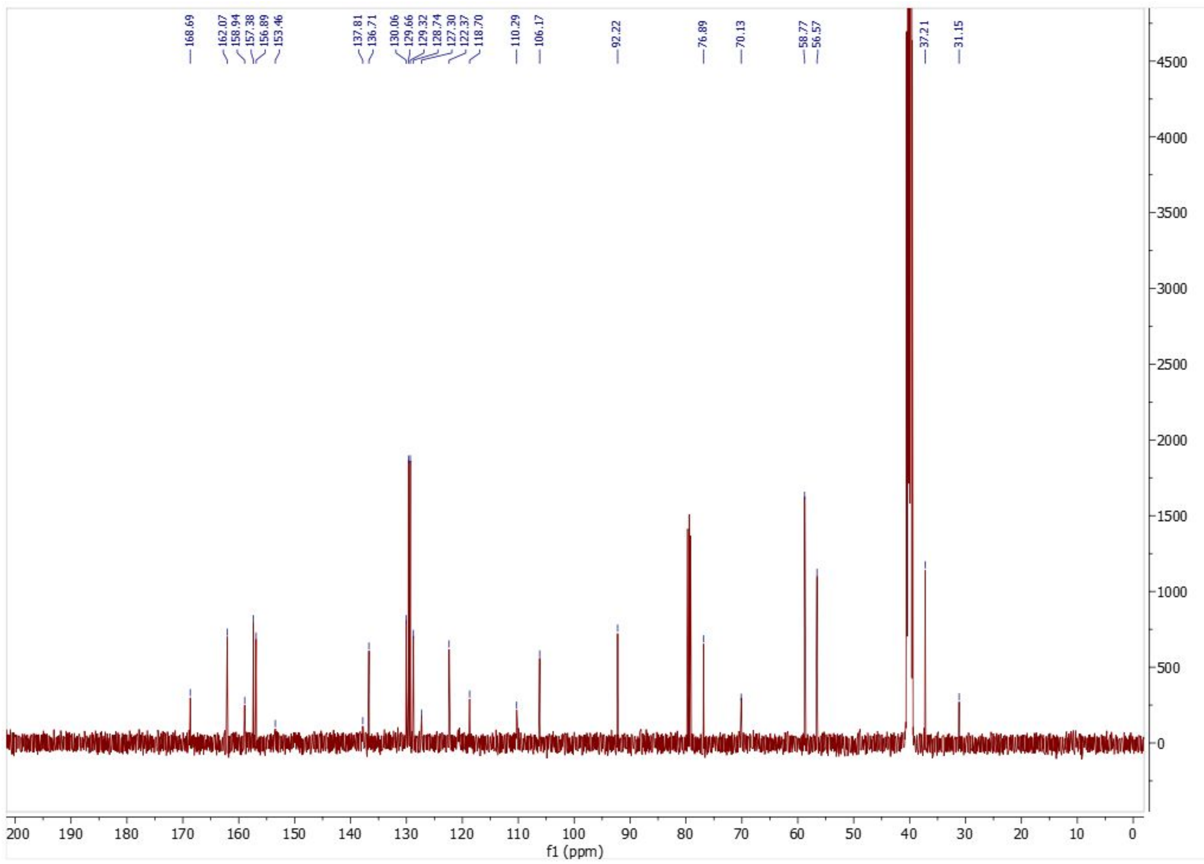

***N*-(4-((5-Ethynyl-7-oxo-8-phenyl-7,8-dihydropyrido[2,3-*d*]pyrimidin-2-yl)amino)-3-methoxyphenyl)-3-methoxy-*N*-methylpropanamide (11)**

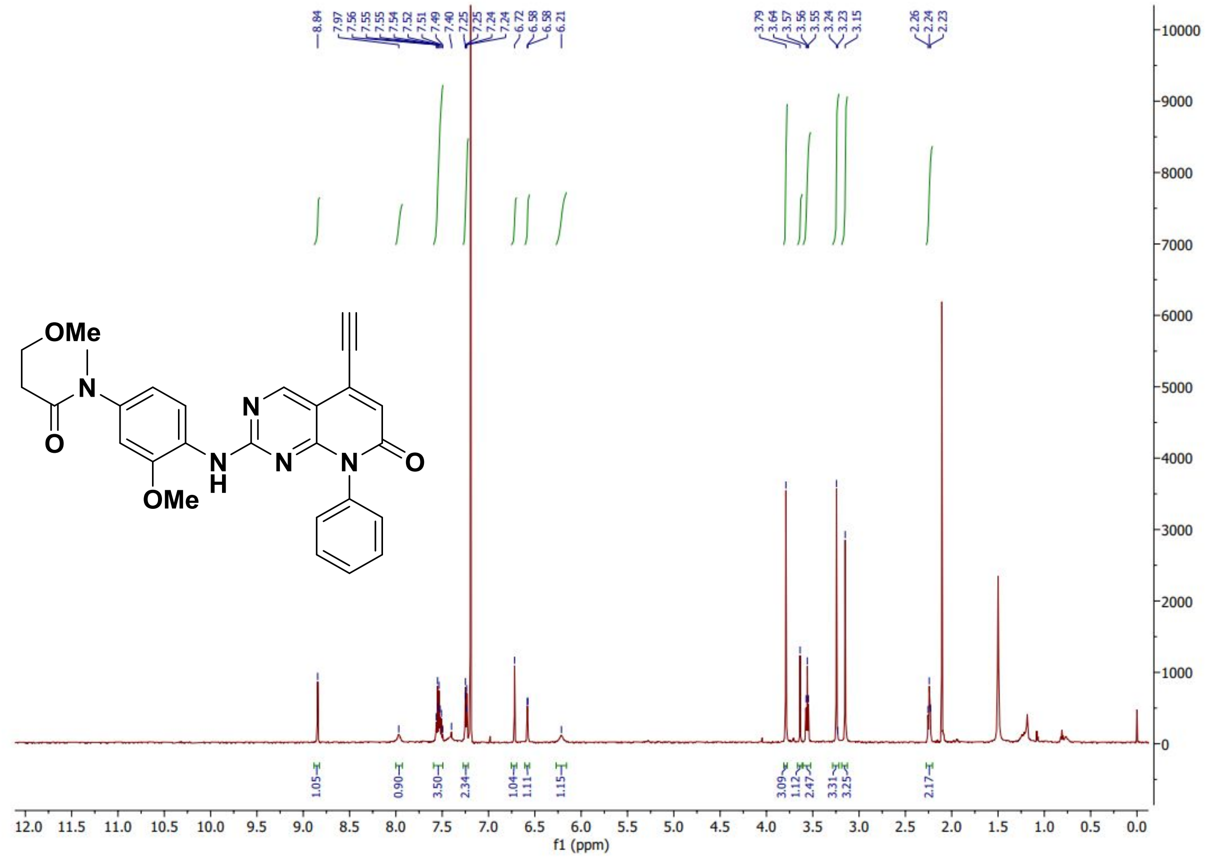

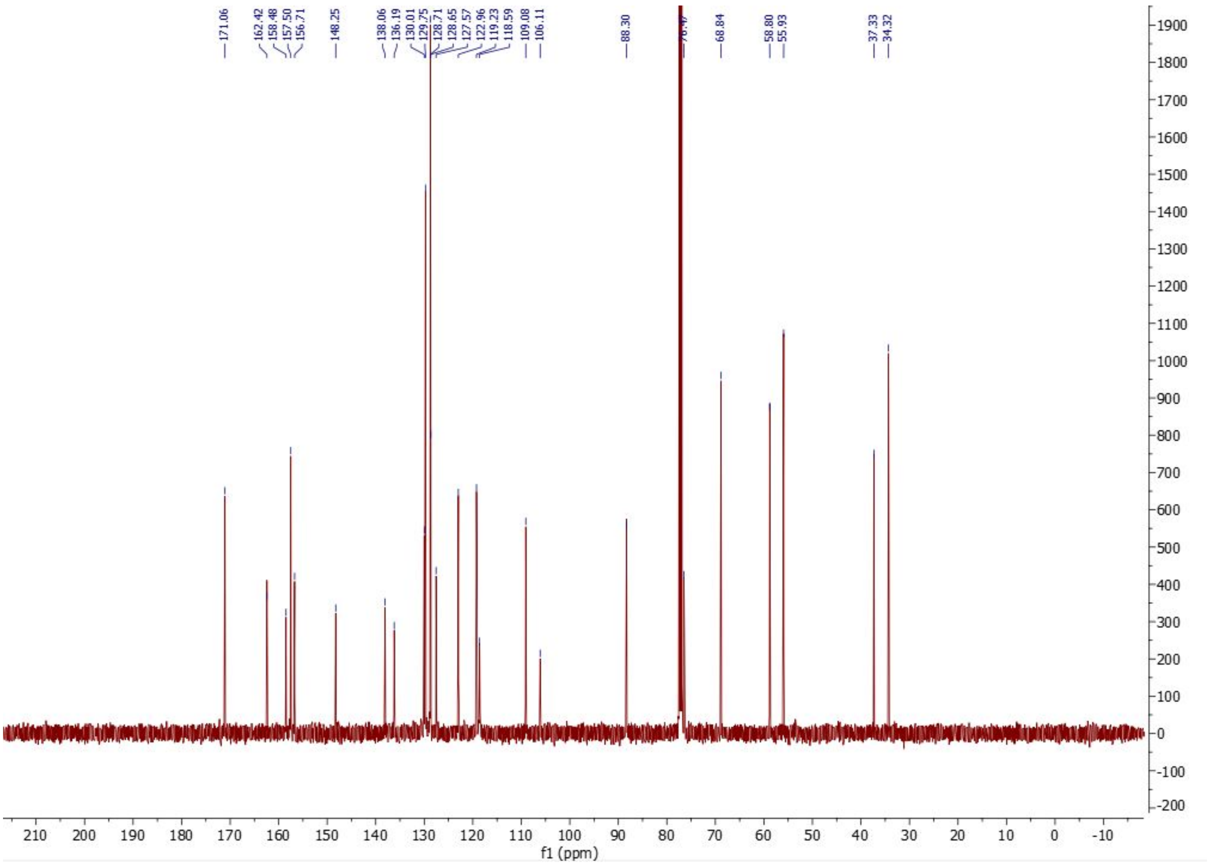

2-(Dimethylamino)-*N*-(4-((5-ethynyl-7-oxo-8-phenyl-7,8-dihydropyrido[2,3-*d*]pyrimidin-2-yl)amino)-3-methoxyphenyl)-*N*-methylacetamide (12)

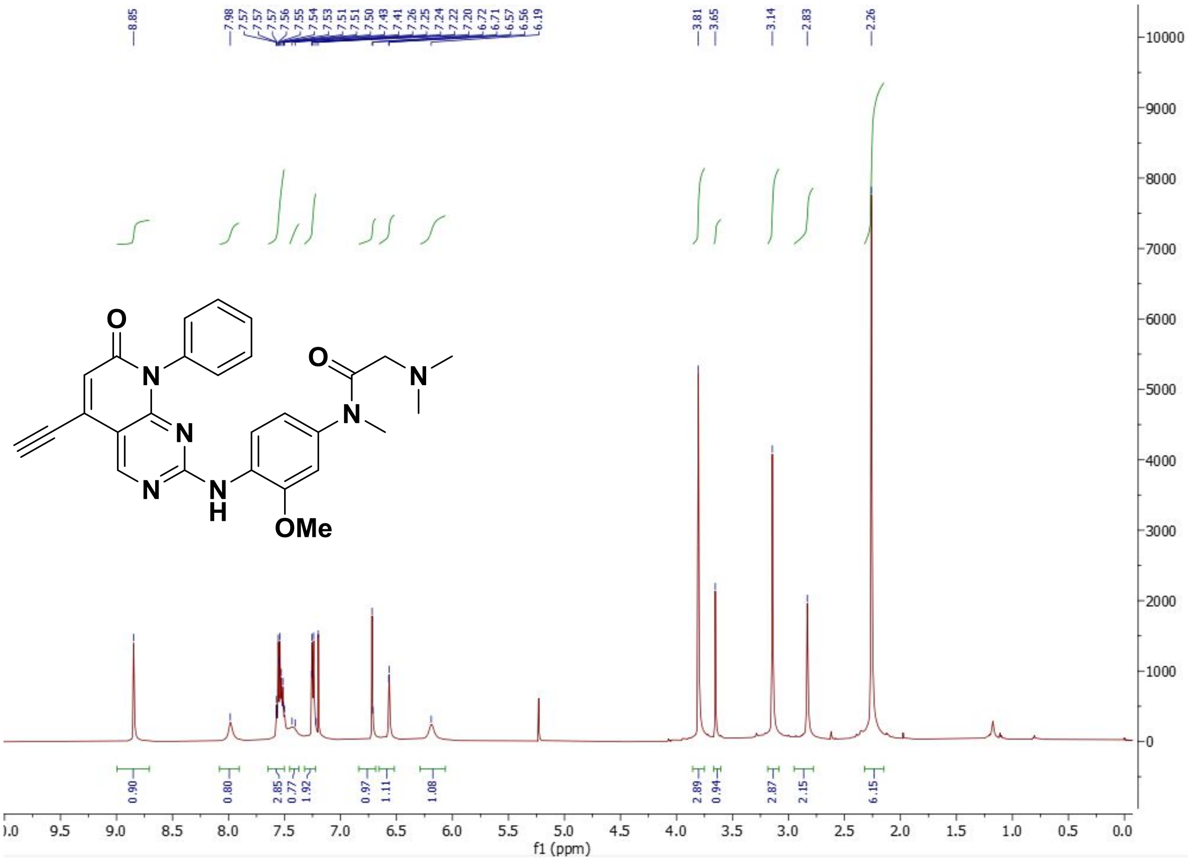

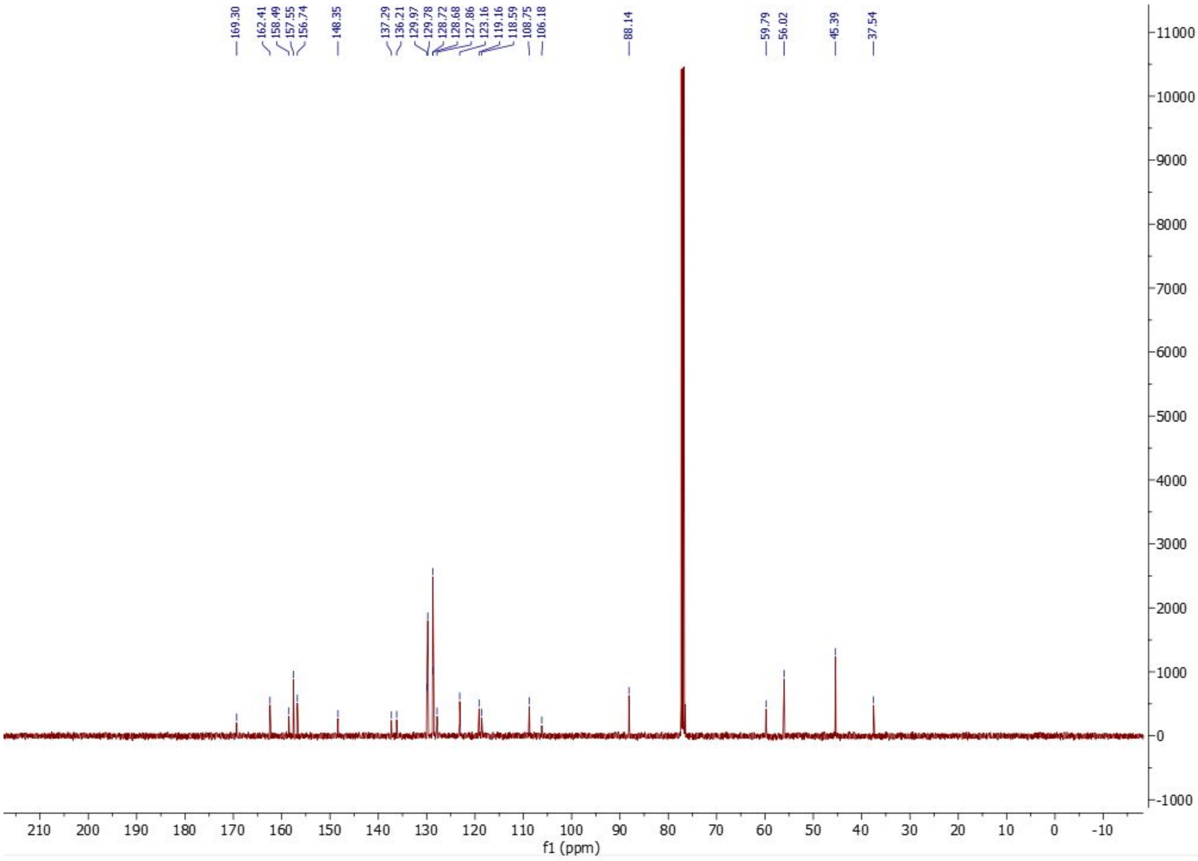

2-((4-((2-(dimethylamino)ethyl)(methyl)amino)phenyl)amino)-8-phenylpyrido[2,3-d]pyrimidin-7(8H)-one (S13)

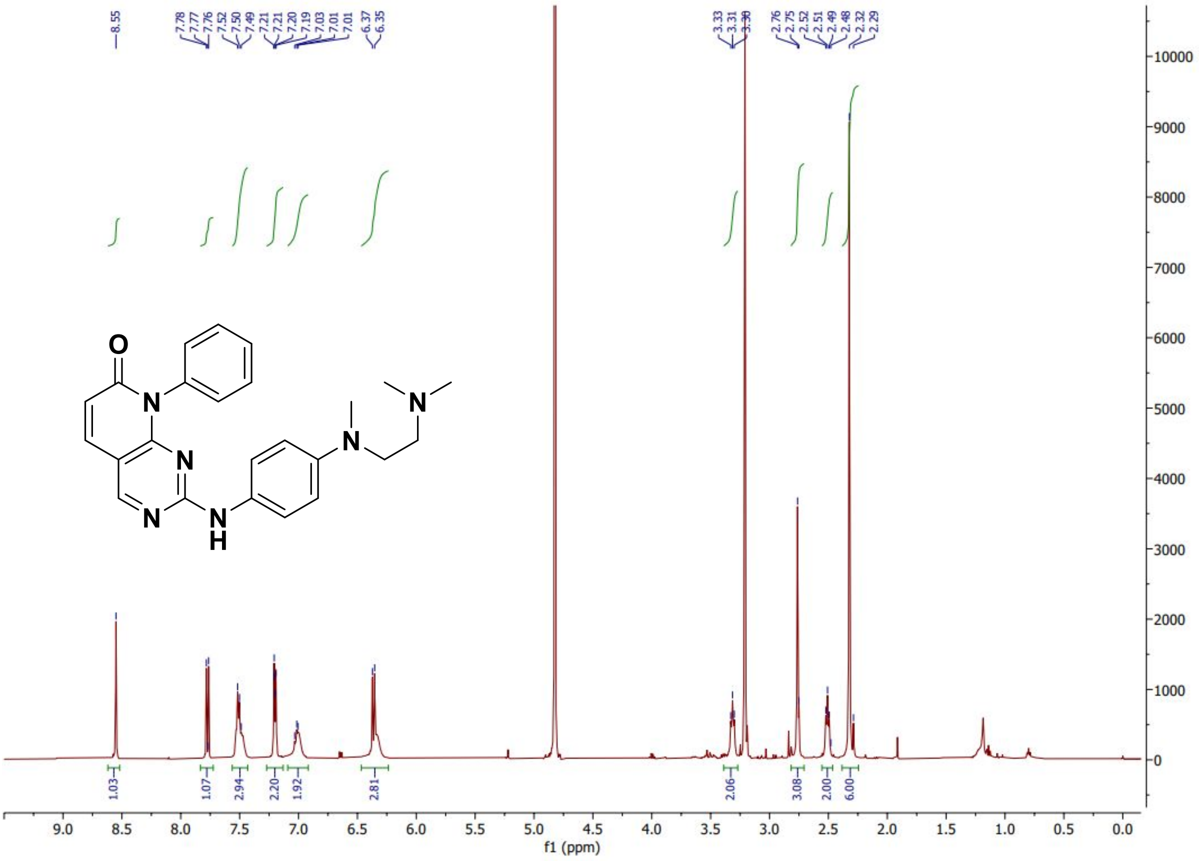

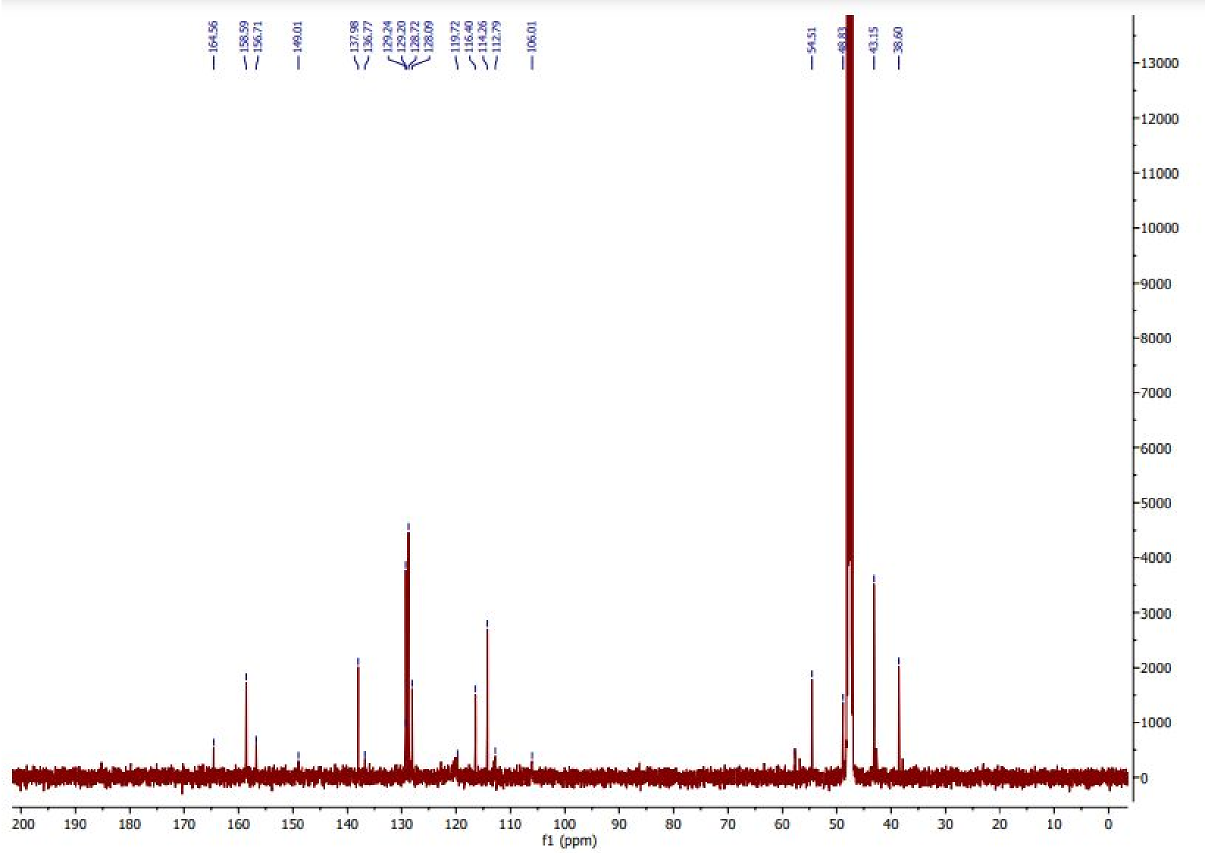

References

1) Yuza Y, Glatt KA, Jiang J, Greulich H, Minami Y, Woo MS, Shimamura T, Shapiro G, Lee JC, Ji H, Feng W, Chen TH, Yanagisawa H, Wong KK, Meyerson M., Allele-dependent variation in the relative cellular potency of distinct EGFR inhibitors. Cancer Biol Ther. 2007, 6, 5, 661-7, 10.4161/cbt.6.5.4003 PubMed 17495523
2) Greulich H, Chen TH, Feng W, Janne PA, Alvarez JV, Zappaterra M, Bulmer SE, Frank DA, Hahn WC, Sellers WR, Meyerson M., Oncogenic Transformation by Inhibitor-Sensitive and -Resistant EGFR Mutants, PLoS Med. 2005, 2, 11, e313, 10.1371/journal.pmed.0020313 PubMed 16187797
3) Naviaux RK, Costanzi E, Haas M, Verma IM., The pCL vector system: rapid production of helper-free, high-titer, recombinant retroviruses. J. Virol. 1996, 70, 8, 5701-5. PubMed 8764092
